# Hardware and software implementation of POCT1-A for integration of point of care testing in research

**DOI:** 10.1016/j.jpi.2022.100096

**Published:** 2022-05-21

**Authors:** Nathan T.P. Patel, Magan R. Lane, Timothy K. Williams, Lucas P. Neff

**Affiliations:** a Department of Surgery, Wake Forest Baptist Medical Center, Winston-Salem, NC, USA; b Department of Cardiothoracic Surgery, Wake Forest Baptist Medical Center, Winston-Salem, NC, USA; c Department of Vascular/Endovascular Surgery, Wake Forest Baptist Medical Center, Winston-Salem, NC, USA; d Department of Pediatric Surgery, Wake Forest Baptist Medical Center, Winston-Salem, NC, USA

**Keywords:** Point-of-care, Informatics, Automated, POCT1-a

## Abstract

Point of care testing (POCT) is increasingly utilized in clinical medicine. Small, portable testing devices can now deliver reliable and accurate diagnostic results during a patient encounter. With these increases in POCT, the issue of data and results management quickly emerges. Results need to be cataloged accurately and efficiently while the providers/support staff are simultaneously managing patient encounters. The integration of electronic medical records (EMR) as data repositories requires that point of care testing data imports automatically into the EMR. POCT1-A was developed as a standard communication language for POCT device manufacturers to streamline automatic data import integration. While all modern POCT devices are built with this connectivity, the systems that provide the integration layer are often proprietary and require a fee for service. In the research environment, there is not enough throughput to justify the practical investment in these data management architectures. Moreover, researcher needs are different and unique compared to data management systems for clinicians. To meet this need, we developed a novel hardware and software connectivity solution using commercially available components to automate data management from a point-of-care blood biochemical analyzer during a critical care study in the preclinical research environment.

## Introduction

Point of care testing (POCT) devices have become increasingly common in the clinical environment. These tools provide real-time decision support to clinicians, but this has simultaneously come with practical concerns over quality control, cost, connectivity, and maintenance.^1^ The connectivity, design, quality of, and management of POCT devices has advanced significantly since implementation of regulatory guidelines and accreditation standards.^2^ Specifically, device connectivity can reduce error rates of transcription and decrease the personnel time required to manage the data.^3^ Given the tremendous need and potential for integrated medical testing platforms, the market has become saturated with clinical grade data management systems that provide seamless integration into electronic healthcare records. These systems are proprietary, often levy a fee for service, and do not allow for advanced configurability. These characteristics of commercialization for a clinical market have left a capability gap for researchers who are using these devices in bench top or translational research environments.

Abbott has licensed the i-STAT Alinity (Abbott U.S., Chicago, Illinois, USA) to Zoetis US (previously through Abaxis, Parsippany, NJ, USA) and branded a next-generation i-STAT Alinity for veterinary medicine. Specifically the i-STAT alinity has the ability to perform arterial blood gas analysis (Cg4+) and electrolyte analysis (Chem8) with acceptable performance.^4^ These tools in the translational research realm provide a quick and reliable way to perform basic analysis in the management of animals or measurement for end points of care.

Our lab focuses on ischemia-reperfusion injury in a swine hemorrhagic shock model and resuscitative endovascular balloon occlusion of the aorta. As such, we utilize large numbers of point of care biomarker tests. i-STAT Alinity affords rapid biomarker analysis for swine receiving critical care in the face of profound hemodynamic instability and provides real-time actionable data for management decisions.^5^

As with most medical devices, the i-STAT Alinity can interface with healthcare records systems, but currently the only available interfacing options are provided through a proprietary system distributed by Zoetis US (Vetscan Fuse). To adapt a device for our unique data management needs, we have developed a hardware and software solution that implements the POCT1-A communication protocol using commonly available parts to download data from the i-STAT Alinity and upload to a database.

## Materials and methods

### Overview

A combined hardware/software platform was developed using open source software and commercially available components to build and deploy a connectivity solution for the i-STAT Alinity. The impetus for creating the platform was the need for logging of point of care testing results due to limited staff for managing the data and also for autonomous resuscitation protocols for standardizing injury management in swine.

**Fig. 1:**
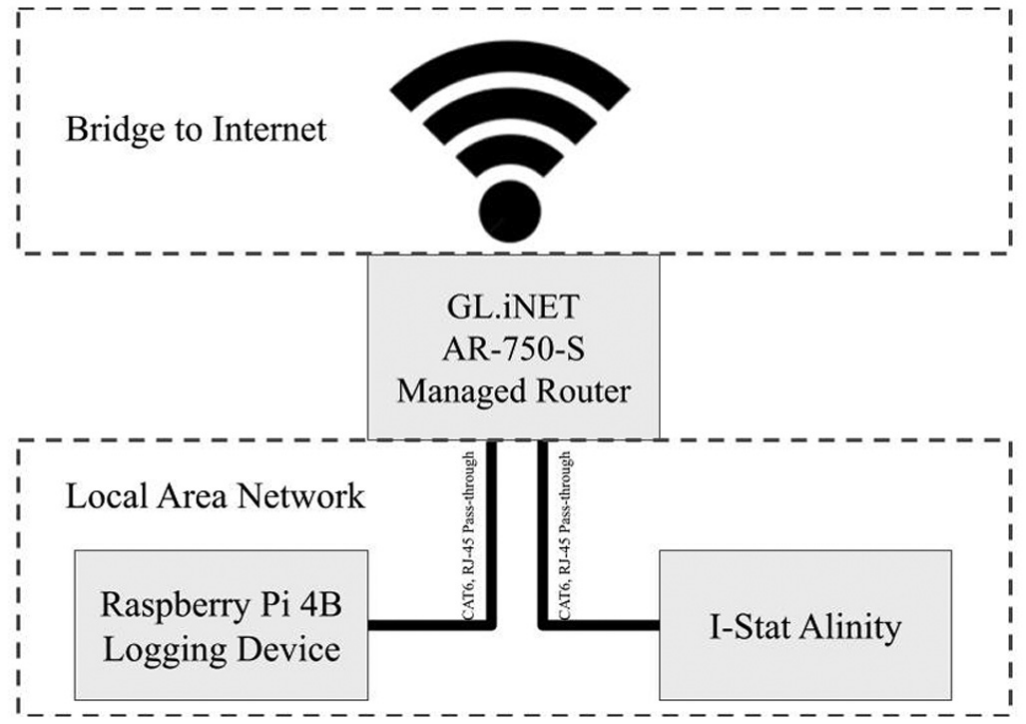
A schematic representation of the hardware design.

The hardware was developed using a Raspberry Pi 4B, 2 gigabytes (GB) of memory, 32 GB MicroSD card (Raspberry PI 4 Starter Kit, Cana Kit Corporation, Tualatin, Oregon, USA) which includes all the pieces for installing the base 32-bit operating system. To set up the computer, a universal serial bus (USB) mouse and USB keyboard along with an High-Definition Multimedia Interface (HDMI) compatible monitor are required. Once the operating system is installed, the Raspberry Pi can be configured to run headless (without monitor or keyboard/mouse) and use Secure shell Protocol (SSH) or Virtual Network Computing (VNC) to communicate with the Raspberry Pi. In addition, a travel router (GL-AR750-S, GL.iNet Bellevue, Washington, USA) is connected to manage a local area network (LAN) for inter-device communication (Fig. 1).

The software was developed using Python 3.7 (Python Software Foundation, Wilmington, Delaware, USA) on a standard distribution of Raspberry Pi 4B (Raspberry PI Foundation, Cambridge, United Kingdom) using preload NOOBS for installation of the operating system Raspbian (Raspberry PI Foundation, Cambridge, United Kingdom). The python packages required include Socket, Json, XML, and Adafruit Digitalio libraries which are downloaded via Pip3. The software can be downloaded and comments made at https://github.com/patnatha/poct1_a.

### Device setup

The i-STAT’s network must be configured using the Network Connectivity Utility Package (NCI) which runs on a standard Windows personal computer, using HyperText Markup Language (HTML) so it will open a web browser (like chrome) and create a text file that must be loaded onto a USB thumb drive (https://www.globalpointofcare.abbott/content/dam/poc/apoc/support/software/NCi-Installer.msi, supplemental file 1, supplemental file 2). This network configuration is to set the device to connect via ethernet port and use Dynamic Host Configuration Protocol (DHCP) for Internet Protocol (IP) address assignment (wireless configuration is not necessary). Software installation is accomplished by inserting the USB thumb drive with the text file in the root directory of the USB. At startup, the i-STAT Alinity which will recognize the USB and text files in the root directory. Using the i-STAT’s touch screen, configuring the network interface which can be reached from the device homepage on start-up and “install” the connectivity configuration. Now, when the i-STAT is docked into its base station, it will attempt to connect to the local area network (LAN) via the ethernet connection. Note, in the configuration settings the “Data Manager Address” and “Port” must be set so the i-STAT knows where to send the data. Set it to 192.168.8.216 on port 8080 for this demonstration.

### Hardware

**Fig. 2:**
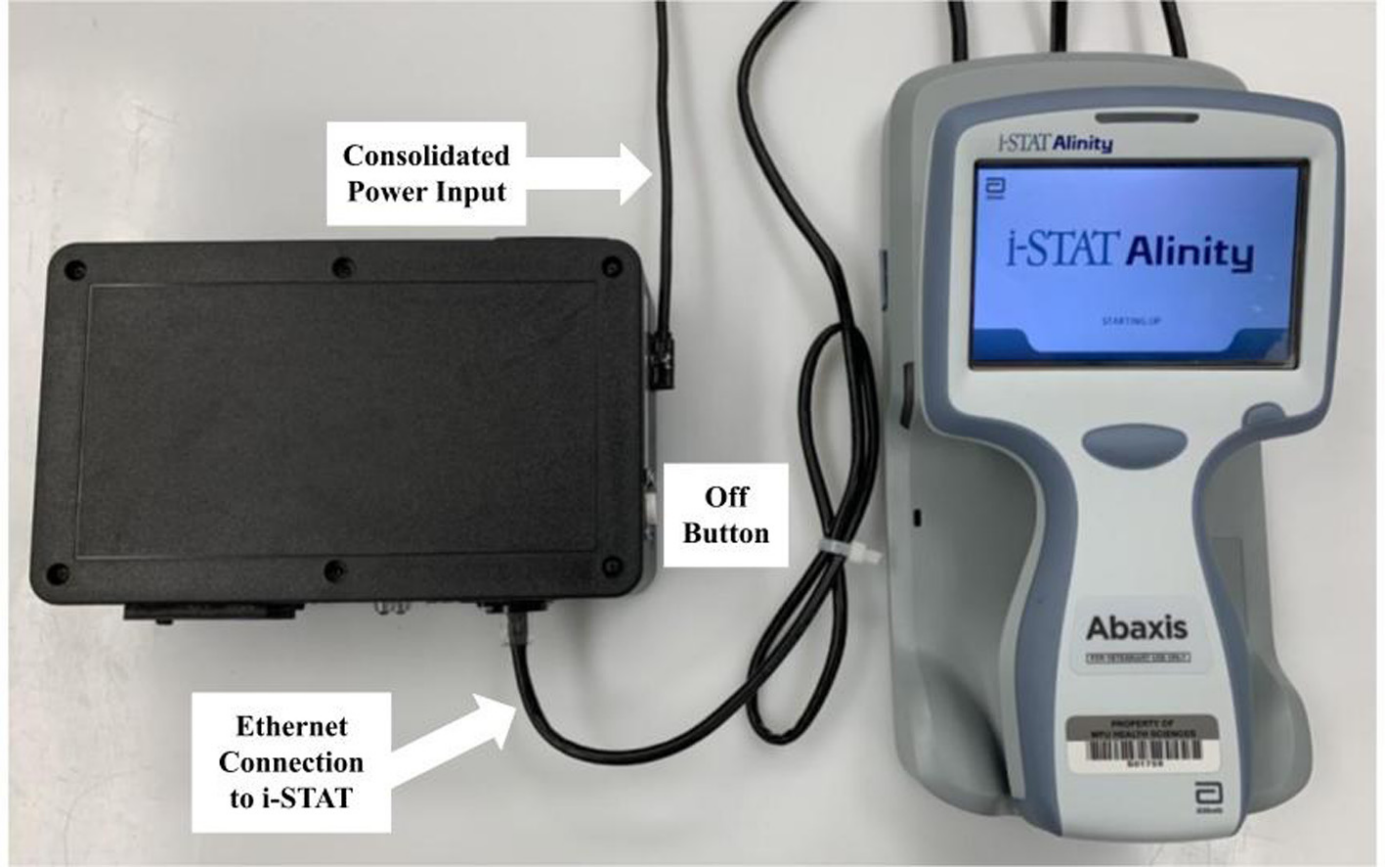
A picture of the logging device (black box on left) connected to the i-STAT Alinity (right) and ready to receive data from the peripheral device.

**Fig. 3:**
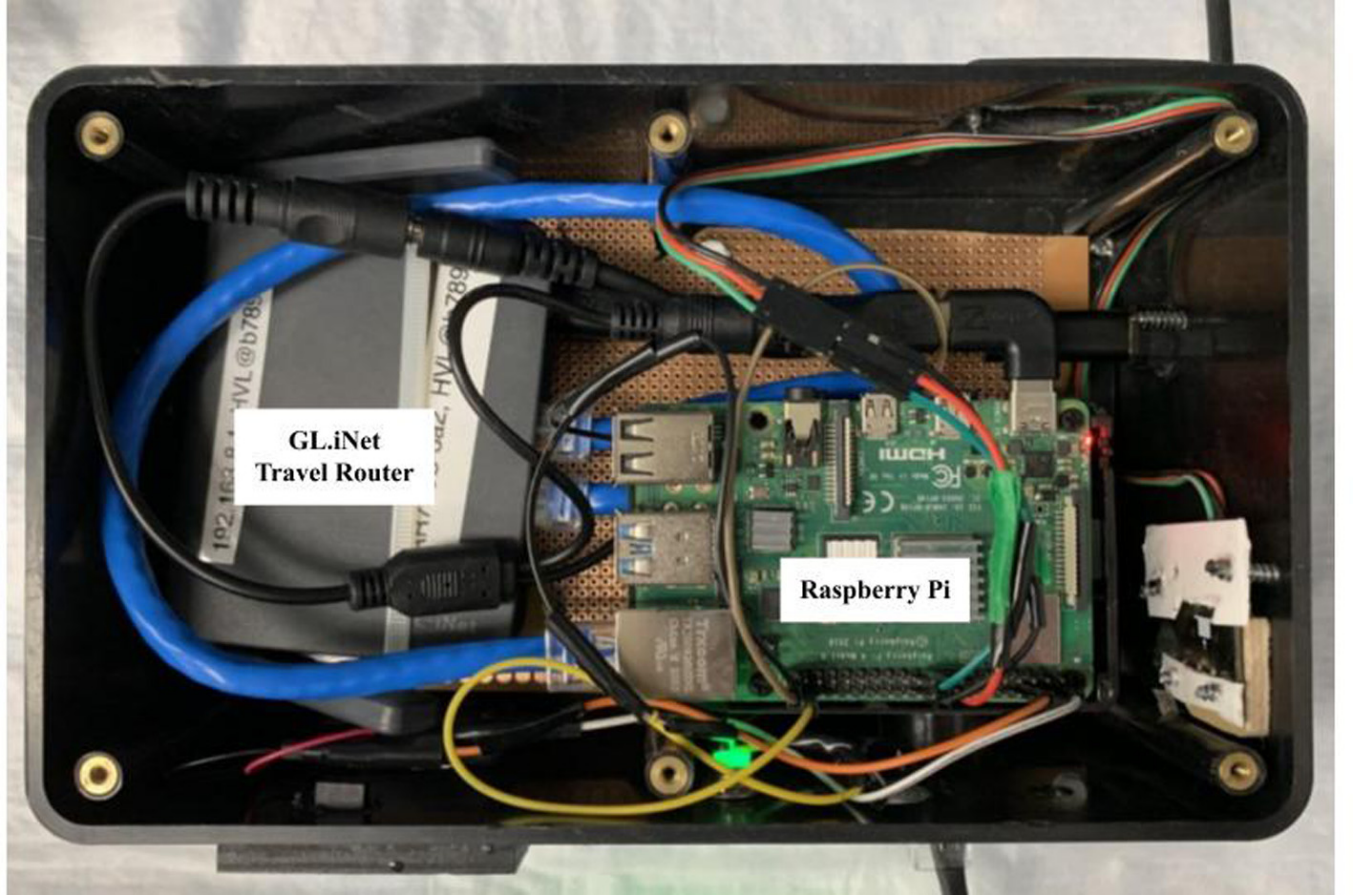
The device is mounted inside of a housing for protection in the clinical space. There is consolidated power for the Raspberry Pi (USB-C, 5 Volts) and the Gl.iNET travel router (USB-Micro, 5 Volts) at the top right. The devices are connected via a pass-through CAT6 cable and an external RJ-45 connector, at the bottom right. This particular device also has two LEDs, middle bottom which show the program running and when reading a new record. An on/off button is located on the side of the Raspberry Pi to provide a safe shutdown option.

A travel router is configured to run a DHCP with the Raspberry PI and i-STAT connected to the LAN via CAT6 RJ-45 pass-through cables. These connections are managed by the travel router and the travel router has an HTML interface (192.168.8.1 while connected to the network) for which a static IP address can be assigned to the Raspberry PI based on the media access control (MAC) address. Set the Raspberry PI to the static IP address of 192.168.8.216. This step ensures that the Raspberry PI is always located at the same IP address so the i-STAT has a reliable endpoint for sending the data. The ultimate aim is for the travel router to serve as a manager of the local area network and a bridge to the local wireless network for communication with an internet accessible database. Our laboratory has a hospital managed guest network through which the local area network is bridged to the world wide web. After designing and debugging, the entire device was mounted into a box and consolidated the power to a single input power source of 5.1 V at 3.5 A, providing ~17 W. The estimates of power consumption at heavy use for the Travel Router and Raspberry PI combined is 12 A. Additionally, a RJ-45 female jack, 2 lights for indications of device functioning, and an off button were mounted on the box to interface with the Raspberry PI (See Fig. 2, Fig. 3).

### Software

A Python 3.7 program was developed to listen on port 8080 using the socket library for incoming connections. When a connection is initiated by the i-STAT it starts a bi-directionl communication pipe for sending bytes of data which are decoded into Unicode characters and the structures of the unicode is Extensible Markup Language (XML). Using a Python-based XML parser, the results can be structured for searching. The communication protocol is based on POCT1-A. We used a publicly available description of the POCT1-A implementation for a Roche Cobas Liat point of care test for Influenza A/B, Respiratory Syncytial Virus, and Group A strep to develop the communication protocol (https://diagnostics.roche.com/content/dam/diagnostics/us/en/products/c/cobas-liat-support/cobas-liat-system-him-poct1-a_-sw-ver.-3.3_ver.-5.2.pdf, Supplemental File 3). There are numerous communication pathways available and many of them are not implemented or slightly different for the implementation of this i-STAT Alinity, but we focused on reading observations from the i-STAT. The bi-directional communication is able to let the logger know how many unsent records are in the queue. Then the logging device can loop through the queued records and parse each result and assign it to the respective column in the database. Then the record is posted to the remote database using an Hypertext Transfer Protocol Secure (HTTPS) POST command. If all this works successfully then the logger can report an acknowledgment command to the i-STAT Alinity which marks the record as sent and removes it from the queue. It will display on the user interface a “Successful Transmit” or a “Failed Transmit” notification.

### Database

Our institution (Wake Forest CTLI, Winston-Salem, North Carolina, USA) maintains an instance of the Research Electronic Data Capture (The REDCap Consortium, Vanderbilt, Nashville, Tenneseee, USA) database for research use. We obtained an account and were able to create a database which provides advanced programming interface (API) access using a text token. A main table for logging lab results metadata (including machine serial number, vender, cartridge information, and expiration date) and then additional tables are created for each cartridge result (CG4+ and CHEM8 for our use case) with each field as an observation (lactate, ph, sodium, potassium...etc.).

### Workflow

There are two possible workflow options. The first option is to press the transmitting records button on the right side of the screen after the results of the test return. This action will initiate a data push of the new result. Additionally, if the machine is left idle, then after a timeout, it will attempt to automatically shutdown. At shutdown, it will automatically send the remaining records in the queue. If the device is not in the dock when the result returns, then one can either set it in the dock and using the options travel to the transmit queue configuration or the next record result it will attempt to send the queued record and the current record. The i-STAT Alinity is designed to always look for an acknowledgement from the logging device and if it does not receive one, then it will present a notification to the user. This functionality is helpful because if the device is not sitting perfectly in the dock it will fail to make the connection to the local area network.

### Analysis

Before this logging device was developed, Cg4 and Chem8 results were analyzed during a swine ischemia-reperfusion injury. The results were printed to paper and then manually transcribed into an online database using an HTML form with minor error checking (forcing integers or floats). The labs were entered during the experiment by one of two technicians who also assisted with other aspects of veterinary management. Then after the experiment the results were spot quality controlled and checked. The technicians estimated that for a single experiment which included 16 Cg4 and 10 Chem8, it took about two hours for entry and quality/control. With 8 values per Cg4 and 11 values per Chem8, with 16 Cg4 and 10 Chem8 per study. This is 238 values per study which must be manually entered and if it takes about 2 hours of time to curate, the time is estimated to be 4 minutes per Cg4 and 5.5 minutes per Chem8.

For any given Cg4 result that were recorded by hand which included temperature corrected pH, temperature corrected partial pressure of carbon dioxide, temperature corrected partial pressure of oxygen, lactate, bicarbonate, base excess, and oxygen saturation. The values that were not recorded and available for download via the automated system included temperature of sample, actual measured pH, actual measured partial pressure of carbon dioxide, actual measured partial pressure of oxygen, and tension of carbon dioxide. This represented 61.5% of the available data that was not recorded by hand, this was chosen before experimentation due to personnel constraints and time required for entry of data during/after an experiment. All values from Chem8 were recorded.

## Results

During a study of critical care resuscitation, 11 swine underwent point of care testing. There were 1280 entries for Cg4 that were recorded by hand and the overall transcription error rate was 9.92% (n=127). On closer examination of the records, 9.6% of the errors (n=124) were due to transcribing the actual measured instead of the temperature corrected value (partial pressure of carbon dioxide, partial pressure of oxygen, and pH). This was likely due to the difference between the two technicians who were entering the values and was sporadic for each animal as they switched off entry throughout a given experiment. Additionally, there were 2 lactate values (0.1%) and 2 bicarbonate (0.1%) values that were not transcribed correctly.

There were 96 entries for Chem8 that were recorded by hand and the overall transcription error rate was 1.04% (n=1). The single transcription error was a Chloride that was recorded as 94 and the actual value was 95.

## Discussion

We have developed the first open source description and implementation of bi-directional communication with a clinical point of care device using the POCT1-A protocol for real-time logging of results to a database. This platform has the primary application for research environments that have significant limitations in personnel bandwidth that may also be engaged in multiple other aspects of clinical care. The present data management system also has the ability for advanced configurability to account for available computing resources. This platform could also add value in resource constrained lab environments that do not have the capital to maintain large enterprise level point of care data management software.

This logging device also enabled an increase in captured data (38.5%) that was otherwise being censored due to time constraints and anticipated lack of value. Transcription error rates were reduced by use of this system and the relevant transcription errors were measured at 0.2–1.0%. The biggest benefit was personal saving time, which was formerly several hours per study (a total of over 50-person hours for our most recent study which included 25 animals) and eliminated the need for training on how to interpret and transcribe the results.

A limitation in this design is the use of a travel router as it does increase the cost of the project (~$70) while not providing any additional functionality that the Raspberry Pi cannot already provide. We constructed a simplified version of the hardware to incorporate only the Raspberry PI using the onboard ethernet port with a direct connection to the i-STAT Alinity. This configuration can serve DHCP over ethernet and bridge to the onboard wifi chip for connection to the world wide web. This more advanced configuration requires a significant amount of modification of the onboard linux networking configurations – a project that requires in-depth knowledge of available packages. This current system can function almost entirely out of the box (standard configuration files) because it relies on usual network design. The travel router described here also has an intuitive HTML-based user interface and the ability to bridge the local area network to a cellular data signal. This feature could have a benefit in rural environments like sub-saharan Africa which has made the technologic leap to wireless communications. This setup also allows for ease of access to the Raspberry Pi via SSH and VNC for debugging, but we expected future versions to be slimmed down to a single Raspberry Pi plugged directly into the i-STAT Alinity.

A potential risk for deployment and maintenance of this system is stability over time. The i-STAT Alinity does require updates and will actually not allow the user to perform point of care tests unless the system has been updated. At any one of these required updates the manufacturer could alter the communication protocol or sever it entirely. There are incentives for the manufacturer to maintain backwards compatibility as revenue generating systems have been designed based on the current architecture. Also the POCT1-A communication protocol has been designed to be open-ended and allow for advanced configurability for a multitude of current and anticipated functionality. But overall this standard is an agreed upon concept, there are no accrediting bodies or inherent limitations on the manufacturer to maintain the current standard of communication. This should be considered when attempting to implement the tool described here-in and maintenance would require technical skill in python programming. But the current system does provide feedback through the i-STAT to alert the user of error with communication. The issue would arise immediately, providing feedback to the user prompting them to return to manual logging till the issue could be resolved.

In conclusion, this is an open source hardware/software solution for reliable point-of-care device connectivity in the veterinary translational research environment leveraging the POCT1-A communication standard.

## Author Contributions

All authors contributed to the literature search, study design, data analysis, data interpretation, and writing equally.

## Declaration of interests

The authors declare that they have no known competing financial interests or personal relationships that could have appeared to influence the work reported in this paper.

## Funding

Funding for this study was provided by the US Army Medical Research and Development Command. Award Number: W81XWH-18-0072.

## Disclosures

L.P.N. and T.K.W. are co-founders of Certus Critical Care, Incorporated. N.T.P.P. is a paid consultant for Certus Critical Care, Incorporated.

## Appendix A Supplementary data

Supplementary material 1 Image 1

Supplementary material 2 Image 2

Supplementary material 3 Image 3
