# Supplementary material for: Hardware and software implementation of POCT1-A for integration of point of care testing in research
Source: J Pathol Inform. 2022 May 21;13:100096. doi: 10.1016/j.jpi.2022.100096 (PMC9576979; doi:10.1016/j.jpi.2022.100096)
Supplement: Supplementary file 3 — Supplementary material 3 [file mmc3.pdf]

cobas® Liat® system

Host Interface Manual POCT1-A (DML)
Version 5.2
Software Version 3.3

Roche

cobas®
Life needs answers

OS-02605-01

COBAS, LIAT and LIFE NEEDS ANSWERS are trademarks of Roche.

©2015-2021 Roche

Roche Diagnostics Corporation
9115 Hague Rd
Indianapolis, IN 46256
USA
www.roche.com
dialog.roche.com

US Publication information

Edition notice

This publication is intended for operators of the cobas® Liat® System.

The contents of this document, including all graphics and photographs, are the property of Roche. No part of this document may be reproduced or transmitted in any form or by any means, electronic or mechanical, for any purpose, without the express written permission of Roche.

Every effort has been made to ensure that the information is correct at the time of publishing. Not all functionality described in this manual may be available to all users. Roche Diagnostics reserves the right to change this publication as necessary and without notice as part of ongoing product development. Such changes may not immediately be reflected in this document.

Screenshots

Any screenshots in this publication are added exclusively for the purpose of illustration. Configurable and variable data such as parameters, results, path names etc. visible therein must not be used for laboratory purposes.

Intended use

This document is intended for the US market only.

Caution: Federal law restricts this device to sale by or on the order of a physician.

Copyright

©2015-2021, Roche Diagnostics. All right reserved.

Trademarks

The following trademarks are acknowledged.

COBAS, LIAT and LIFE NEEDS ANSWERS are trademarks of Roche.

All other product names and trademarks are the property of their respective owners.

Distribution

Roche Diagnostics, Indianapolis, IN USA 46256

Feedback

This document was created by Roche Molecular Systems, Inc. and the Roche Diagnostics Engineering Operations department. Direct questions or concerns regarding the contents of this document:

Roche Diagnostics Corporation
Engineering Operations Department
9115 Hague Road
Indianapolis, IN 46256
USA

Document availability

This document is available on the Roche Diagnostics USA website at dialog.roche.com.

OS-00191-04

Due to the increasing complexity of laboratories and the increase in types of tests being run, it is critical to use unique host download codes for each test when mapping codes on your Laboratory Information System (LIS). It is also strongly recommended to use alpha or alphanumeric codes on your LIS. If unique LIS test codes are not used when mapping on the LIS, this could cause a test result from one test to be reported for a different test.

Required actions when using this host interface manual:

- ***Ensure that the LIS test codes mapped to your LIS are unique for each test.***
- ***Always identify the instrument source of the results on your LIS.***
- ***Please disregard any application code numbers in this host interface manual. Refer to the appropriate method sheet, package insert or application code numbers document for the most current application code number information.***

OS-00191-04

cobas® Liat® System

Host Interface Manual POCT1-A (DML)
Version 5.2
Software version 3.3

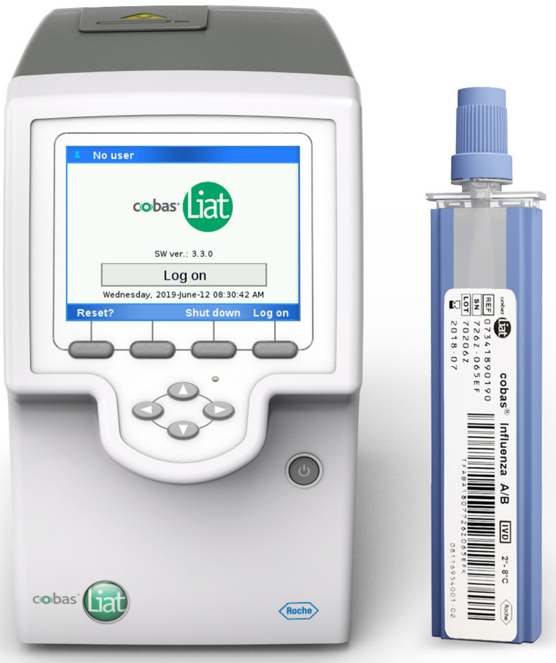

OS-02605-01

Publication information

Publication version	Software version	Revision date	Change description
1.0	2.1	December 2015	First release.
2.0	2.1.1	February 2016	Software update.<br>Branding, address, and intended use updates.
3.0	3.0	June 2016	Software update<br>Edition notice updated.<br>Approvals section updated.<br>Communication scenarios updated.
3.1	3.1	May 2017	New functionality:<br>• Operator lists<br>• Lot lists<br>• Enhanced error and event handling<br>• Observation (test) names updated so as to include the script used to process them, e.g.: Influenza A (FABA)<br>Updated user interface<br>Communication scenarios updated.<br>Sequence and other UML diagrams added.<br>Security enhancements, secure communications.
4.0	3.2	February 2018	New functionality:<br>• `GEN_CFG` object barcode attributes added.<br>Corrections:<br>• Access control object (ACC) object permission_level_cd element supports the string "Administrator" for administrator rights.<br>• Generic configuration object (GEN_CFGD) element: DateTime.TimeZone only supports certain strings. These must be exactly correct to prevent serious problems.
4.1	3.2	April 2020	New functionality:<br>• Assay SARS-CoV-2 (SCFA) added<br>• Removal of MRSA assay-related information

Revision history

Publication version	Software version	Revision date	Change description
5.0	3.3	Feb 2020	New functionality:<br>• Support of patient verification workflow<br>Patient verification (63)<br>Patient verification objects (86)<br>Patient verification request message (ROCHE.LIAT.PVI.R01) (119)<br>Patient verification response message (ROCHE.LIAT.PVR.R01) (120)<br>• Support of `note_txt` element in Acknowledgment object (ACK).<br>Acknowledgment object (ACK) (70)<br>• New section explaining the conversion of the manufacture lot number into the barcode lot number.<br>About lot number (104)<br>• Update of device configuration message<br>Device configuration message (DTV.ROCHE.LIAT.CFG) (116)<br>New settings added in the GEN_CFG object.<br>Corrections:<br>• Terminology of communication protocols<br>• Supported range for the Autolock time (1 to 1440 minutes)<br>• Connectivity item **Server**: fully qualified names are supported.<br>• Connectivity item **Data synchronization**: list of supported data topics has been updated.
5.1	3.3	July 2020	New functionality<br>• Assay SARS-CoV-2 (SCFA) added<br>• Examples for SARS-CoV-2 (SCFA) added
5.2	3.3	February 2021	• Added information about Ethernet configuration and speed<br>Corrections:<br>• Description of un-acknowledged messages in Observations (results)<br>• AutoReboot.Time formats in Generic configuration object (GEN_CFG)<br>• Link to example in Keep alive message (KPA.R01)<br>• Added related topics to Patient verification request message (ROCHE.LIAT.PVI.R01) and Patient verification response message (ROCHE.LIAT.PVR.R01)<br>• Removed spurious ACC.expiration_date in Operators topic<br>• Added description of message highlighting in Example message logs<br>• Removed extra EVT in Communication scenario 5 - Send a validated assay lot to a DMS<br>• Updated patient verification message example and table<br>• Added missing PRContent.Users tag to message examples<br>• Updated software version in message examples

Revision history

Edition notice

This publication is intended for operators of the **cobas**® Liat® Analyzer.

Every effort has been made to ensure that all the information contained in this publication is correct at the time of publishing. However, the manufacturer of this product may need to update the publication information as output of product surveillance activities, leading to a new version of this publication.

Where to find information

The **cobas**® Liat® System **User Guide** contains all information about the product, including the following:

- Safety
- Installation
- Routine operation
- Maintenance and calibration
- Troubleshooting information
- Configuration information
- Background information
- Approvals
- Contact addresses

The **cobas**® Liat® **Quick Start Guide** is intended as a reference during the **cobas**® Liat® Analyzer setup.

The **cobas**® Liat® **Cleaning Tool Guide** is intended as an instruction for using the **cobas**® Liat® Cleaning Tool with the **cobas**® Liat® Analyzer.

The **cobas**® Liat® **Advanced Tool Guide** is intended as a reference for performing various functions, including archiving data and syncing assay tube lots between **cobas**® Liat® Analyzers.

The **cobas**® Liat® System **Host Interface Manual HL7** contains all necessary information about the HL7 interface.

The **cobas**® Liat® System **Host Interface Manual POCT1-A (DML)** contains all necessary information about the DML interface.

The **cobas**® Liat® System **User Assistance** is the online help version of the **cobas**® Liat® System **User Guide**.

Privacy notice
When you use User Assistance online, viewing events (topics viewed and searches performed) and IP addresses are logged.
The data collected is for Roche internal use only and is never forwarded to third parties. It is anonymized, and after one year it is automatically deleted.
Viewing events are analyzed to improve User Assistance content and search functionality. IP addresses are used to classify regional behavior.

⚠ **General attention**

To avoid incorrect results, ensure that you are familiar with the instructions and safety information.

- Pay particular attention to all safety precautions.
- Always follow the instructions in this publication.
- Do not use the instrument in a way that is not described in this publication.
- Store all publications in a safe and easily retrievable place.

⚠ **Incident reporting**

- Inform your Roche representative and your local competent authority about any serious incidents which may occur when using this product.

Images

The screenshots and hardware images in this publication have been added exclusively for illustration purposes. Configurable and variable data in screenshots, such as tests, results, or path names visible therein must not be used for laboratory purposes.

Example communication messages

Any software version, build number and variable data included in the example communication messages are for illustration purposes only. They might not match what is actually transmitted by the analyzer or DMS.

Warranty

Any customer modification to the analyzer and the use of unauthorized assay tubes or accessories renders the warranty null and void.

Do not open the **cobas**® Liat® Analyzer, change a component, or install unauthorized software.

License information

cobas® Liat® software is protected by contract law, copyright law, and international treaties. **cobas**® Liat® System contains a user license between F. Hoffmann-La Roche Ltd. and a license holder, and only authorized users may access the software and use it. Unauthorized use and distribution may result in civil and criminal penalties.

Open-source and commercial software

cobas® Liat® System may include components or modules of commercial or open-source software. For further information on the intellectual property and other warnings, as well as licenses pertaining to the software programs included on the **cobas**® Liat® Analyzer, refer to the USB flash drive distribution of this **cobas**® Liat® Analyzer Software.

This open-source and commercial software and **cobas**® Liat® System as a whole can constitute a device regulated in accordance with applicable law. For more detailed information, refer to the corresponding user documentation and labeling.

Note that the respective authorization is no longer valid according to the corresponding legislation should any unauthorized changes be made to **cobas**® Liat® System.

Copyright

© 2014-2021, Roche Molecular Systems, Inc.

Trademarks

The following trademarks are acknowledged:

COBAS and LIAT are trademarks of Roche.

All other trademarks are the property of their respective owners.

Patents

See *http://www.roche-diagnostics.us/patents*

Support

If you have any further questions, contact your Roche representative with the system information collected as described in the Troubleshooting chapter in the **cobas**® Liat® System User Guide.

Feedback

Every effort has been made to ensure that this publication fulfills the intended use. All feedback on any aspect of this publication is welcome and is considered during updates. Contact your local Roche representative, should you have any such feedback.

In the U.S., call the following number: 1-800-800-5973 or contact Roche Technical Support at indianapolis_usa.liatsupport@roche.com.

Table of contents

Publication information 2
Table of contents 7
Intended use 9
Symbols and abbreviations 9
Supporting documents 11

About communication and workflows

1 About communication
About the analyzer functions 17
About the POCT1-A communication standard 18
Device interface 20
About connectivity 21
Device messaging layer 22
Communication initialization 25
Communication termination 28
Error handling 30

2 Supported workflows
Workflows 37
Observations (results) 41
Operator and lot lists 44
Device Configuration 53
Events 55
Patient verification 63

POCT1-A protocol

3 POCT1-A objects
Standard POCT1-A Objects 69
Custom objects 85

4 Message structure
Supported POCT1-A message structure 108
Custom **cobas**® Liat® messages 116

Communication examples

5 Communication examples
Communication start up topics 127
Lot topic 130
Observation topic 134
Operators topic 140
Device configuration directive 143
Communication ending 146
Keep alive message 147

6 Example message logs
FABA and SASA observation message log 151
FRTA observation message log 156
SCFA observation message log 159

7 Additional examples
Communication scenarios 165

Intended use

Refer to the **cobas**® Liat® System User Guide.

Symbols and abbreviations

Definitions

Even though this document is based on the POCT communication standard "POCT1-A2 - Approved Standard Second Edition" the common term "POCT1-A" is used in this document to name the POCT communication standard.

Throughout this document, the term "conversation" will be used when referring to an exchange of POCT1-A messages between the analyzer and a DML. The boundaries of a conversation are marked by an initiation message and its correspondent termination message. All messages between the initiation and the termination, plus the initiation/termination themselves will determine the length of a conversation.

Product names

Except where the context clearly indicates otherwise, the following product names and descriptors are used.

Product name	Descriptor
**cobas**® Liat® Analyzer	analyzer
**cobas**® Liat® Assay Tube	assay tube

Product names

Symbols used in the publication

Symbol	Explanation
•	List item
	Cross-reference to related topics containing further information.
	Tip. Extra information on correct use or useful hints.
	Extra information within a task
	Figure. Used in figure titles and cross-references to figures.
	Table. Used in table titles and cross-references to tables.
	Code example. Used in code titles and cross-references to codes.

Symbols used in the publication

Symbols used in diagrams

Symbol	Definition
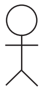	Actors
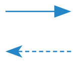	POCT1-A message
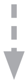	Timeline

Symbols used in diagrams

Abbreviations

The following abbreviations are used.

Abbreviation	Definition
**ADT**	Admission, Discharge and Transfer – a message type used in HL7 formatted communication
**CDR**	Clinical Data Repository
**CIC**	Connectivity Industry Consortium
**CIS**	Clinical Information System
**CLSI**	Clinical and Laboratory Standards Institute (formerly NCCLS)
**DAP**	Device and Access Point interface – specifies the POCT1-A interface between a POC device and an access point.
**DML**	Device Messaging Layer – specifies the complete POCT1-A messaging protocol (message types and message flow).
**DMS**	Data Management System (also called observation reviewer)
**EDI**	Electronic Data Interchange – term used in many industries to describe protocols to exchange data between enterprise-class information systems.
**EUI-64**	64-bit Extended Unique Identifier (for detailed information see http://standards.ieee.org/regauth/oui/tutorials/EUI64.html)
**HIS**	Hospital Information System – a comprehensive information system dealing with all aspects of information processing in a hospital. This encompasses human (and paper-based) information processing as well as data processing machines.
**HL7**	Health Level 7 - an organization that provides connectivity standards for the healthcare industry (see http://www.hl7.org/ for detailed information)
**IEEE**	Institute of Electrical and Electronics Engineers
**LAN**	Local Area Network – a computer network

Abbreviations used in the publication

Abbreviation	Definition
**LIS**	Laboratory Information System – a class of software which handles storing information generated by laboratory processes.
**NACK**	Negative acknowledgment
**ORI**	Observation Reporting Interface
**OSI**	Open Systems Interconnection
**POC**	Point of Care, the location or action of dealing directly with a patient
**POCC**	Point of Care Coordinator
**POCT**	Point of Care Testing – diagnostic testing performed near or at the patient care facility or bedside.
**QC**	Quality Control
**SW**	Software version
**TCP/IP**	Transmission Control Protocol/Internet Protocol. The standard internet communication protocol.
**UTF**	Unicode Transformation Format
**TLS**	Transport Layer Security
**XML**	Extensible Markup Language (see http://www.w3.org/XML/ for detailed information)

Abbreviations used in the publication

Supporting documents

This document makes references to or assumes familiarity with the information contained in the following documents:

External

POCT1-A2 "Point-of-Care Connectivity - Approved Standard Second Edition" standardized under CLSI Vol. 26 No. 28; refer to www.clsi.org.

Internal

cobas® Liat® System User Guide

About communication and workflows

1 About communication 15
2 Supported workflows 35

About communication

In this chapter

1

About the analyzer functions 17

About the POCT1-A communication standard 18

Device interface 20

About connectivity 21

Device messaging layer 22
- Messaging components (nomenclature) 22
- Message encoding 22
- Messaging profile 24

Communication initialization 25

Communication termination 28
- Initiated termination 28
- Abnormal termination 28

Error handling 30
- Application errors 30
- Protocol errors. 31
- Errors processing topics 31
- Application timeout 32
- Keep alive 33

About the analyzer functions

The analyzer and the associated disposable assay tubes are for in vitro diagnostic use. The analyzer identifies and/or measures the presence of genetic material in a biological sample. The analyzer automates all nucleic acid test (NAT) processes, including reagent preparation, target enrichment, inhibitor removal, nucleic acid extraction, amplification, real-time detection, and result interpretation in a rapid manner.

Overview

The assay tube uses a flexible tube as a sample processing vessel. It contains all assay reagents pre-packed in tube segments separated by seals. Multiple sample processing actuators in the analyzer compress the assay tube to selectively release reagents, move the sample from one segment to another, and control reaction conditions. A detection module monitors the reaction in real time, while an on-board computer analyzes the collected data and outputs an interpreted result.

In a typical assay, a sample is first mixed with an internal control and then with lysis reagents. Magnetic glass particles are incubated with the lysed sample for nucleic acid enrichment, and are then captured and washed to remove possible inhibitors. Subsequently, nucleic acid is eluted from the magnetic glass particles and transferred alternately between tube segments at different temperatures for rapid PCR amplification and real-time detection.

For more detailed information about the analyzer, refer to the **cobas**® Liat® System **User Guide**, chapter **About the analyzer**.

About the POCT1-A communication standard

The analyzer can connect to hosts using the POCT1-A communication standard.

Host communication, using the POCT1-A standard, supports reading and adjusting settings, loading of users and patients and reading of measurement results (observations). The POCT1-A defines a minimal set for compliance, and a framework for extensions, because not all analyzer-specific requirements are covered by the POCT1-A standard objects and messages.

Additional vendor-specific objects and messages are specified in the sections Custom objects (85) and Message structure (107).

The communication with the data management server is shown below (in POCT1-A compatible terminology showing the POCT1-A interfaces).

The POCT1-A standard describes 2 types of communication interfaces for the data exchange:

- *Device Interface:* controls the flow of information between POC devices and Observation Reviewers (DMS).
- *Observation Reporting Interface:* describes messaging between Observation Reviewers and Observation Recipients (Hospital or Laboratory Information System) based on HL7 version 2.5.1. messages. This interface is used to send test and QC results from the analyzer to the HIS or LIS.

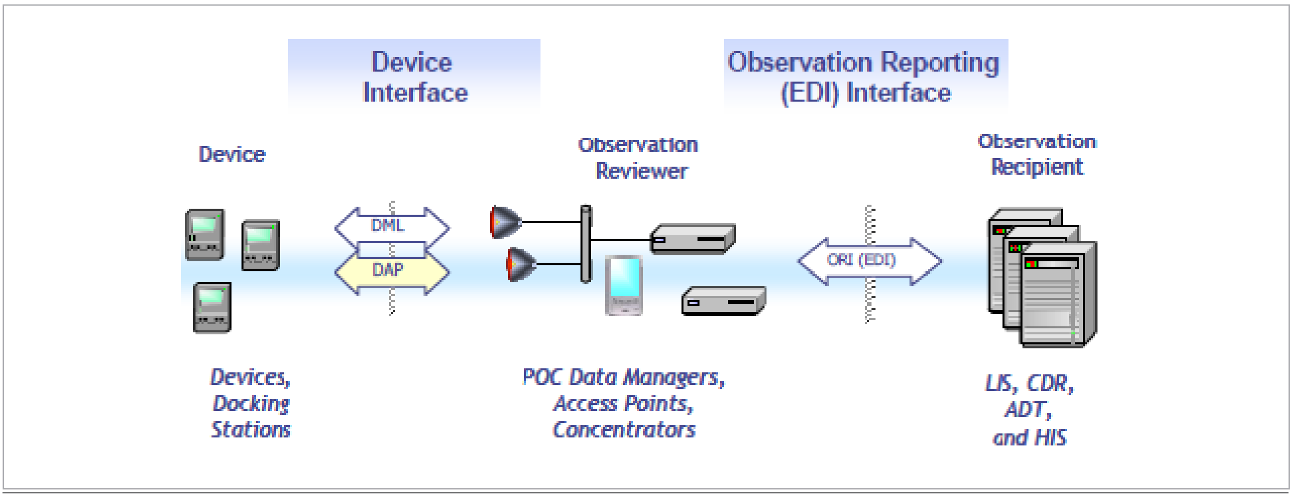

Overview of POCT1-A interfaces

This document defines the details of the Device Interface as shown in the figure above. The communication between the data management server (DMS) and any hospital information systems depicted through the Observation Reporting Interface or EDI is out of scope of this document.

The main objectives of making the analyzer POCT1-A compatible for communication with the DMS are:

- Bidirectional communication – allow the analyzer to send data to, and receive data from, an external DMS utilizing existing standards.
- QC and regulatory compliance – allow the analyzer to send QC data to the DMS.

Device interface

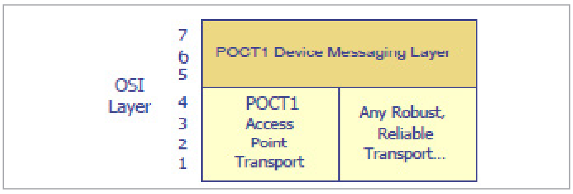

The Device Interface controls the communication between two parts of the deployed POC system – the POC device (device) and the POC Data Manager (DMS) – that need to be independent but are usually quite tightly coupled.

The Device Interface achieves the flexibility it needs as an interface while addressing the tight coupling that exists between the POC Device and the Observation Reviewer by defining its specification in two parts:

- Device Messaging Layer (DML) Specification – describes the structure, content, and flows of messages between a POC Device and an Observation Reviewer (OSI layer 5-7).
- Device and Access Point (DAP) Specification – defines a reliable, low-cost, and flexible means to communicate these messages (OSI layer 1-4). The analyzer DAP specification is detailed below.

Related topics

- The analyzer DML specifications is covered in Device messaging layer (22)

About connectivity

For connectivity related information, refer to the **cobas**® Liat® System **User Guide**, chapter **Connectivity**, which covers the following topics:

- How to connect the analyzer manually to the network
- How to connect the analyzer to the Roche remote service
- How to connect the analyzer to a host system
- Conceptual information about security, monitoring host connectivity, and data exchange with a DMS
- How to define and configure network resources
- How to back up results
- How to use the share lot folder
- How to configure the share lot function

Secure certificate validation

The analyzer uses TCP/IP based communication with the DMS through a wired LAN connection. The TCP/IP stack handles most of the lower-level communication protocol.

The analyzer can establish a secure connection with DMS hosts that support the Transport Layer Security (TLS) protocol version 1.2.

The secure communications server's certificate needed for establishing the secure TLS v1.2 connection shall be manually acknowledged on the analyzer by the Administrator. This acknowledgment just needs to be done once, prior to the first secure connection to the DMS. All upcoming secure connections will "remember" this first manual Acknowledgment, and will use the stored value to verify the identity of the DMS host.

Ethernet connection

The Ethernet connection does not need to be configured. The **cobas**® Liat® System is capable of communicating at 10/100 Mbps, at full or half duplex. The highest common speed between the connected devices is used.

Device messaging layer

This section introduces the Device Messaging Layer for the analyzer. This layer is responsible for high-level communication between the analyzer and the DMS. This high-level communication is described in terms of concepts defined by the POCT1-A standard.

Messaging components (nomenclature)

The messaging components for POCT1-A compatible communication are described in POCT1-A2 "Point-of-Care Connectivity - Approved Standard Second Edition" standardized under CLSI Vol. 26 No. 28; refer to www.clsi.org, appendix B, chap. 2.4.

The "SN" and "SV" attributes are used to specify the code set from which the value contained in the ‘V’ attribute is drawn.

- SN: The name of the registering authority for the code set
- SV: The version of the code set

If SN and SV attributes are transmitted, the value for the attribute "SN" is set to "ROCHE" and the attribute "SV" is set to "1.0".

If none of these coding system attributes (i.e. SN, SV) are specified, the code set is assumed to be the “POCT1-A” standard. In this case these attributes are not transmitted.

Message encoding

The analyzer uses XML for application-level message encoding. The XML encoding rules are those defined in the section on Data Types in the HL7 specification.

Both the DMS and the device are responsible for encoding (and decoding) data values according to the XML 1.0 (Fifth edition) specifications.

Character encoding used in the message is UTF-8 and is identified in the message XML header.

```
<?xml version="1.0" encoding="UTF-8"?>
```

If a received POCT message does not include the used character set encoding in the XML declaration, the default xml character set encoding UTF-8 is assumed.

Supported characters by the device

The supported characters that can be entered via the touch screen of the device are limited to ASCII characters without the following character: “`” (char 96 ASCII).

(space) ! # % & ( ) * + , - . _ / : ; < > ? ` ’
0 1 2 3 4 5 6 7 8 9
A B C D E F G H I J K L M N O P Q R S T U V W X Y Z
a b c d e f g h i j k l m n o p q r s t u v w x y z
Ä À Á Ã Å Â Ö Ô Ò Ó Ø Ü Ù Ú Ë Ê É È Ï Ì Í Î Æ Ç Ñ ß
ä à á ã å â ö ô ò ó ø ü ù ú ë ê è é ï ì í î æ ç ñ

Nevertheless, other characters received by a connected DMS can be displayed on the UI.

Non-supported characters

For any attribute received by the analyzer (e.g. user ID, user name, lot number etc.), the following characters are not supported:

- "
- '
- `

In addition, when receiving user IDs or sample IDs, the following characters are not supported:

- [Space] (ASCII: Space)
- |
- ^
- &
- \
- ~
- [CR] (ASCII: Carriage Return)
- [VT] (ASCII: Vertical Tabulator)
- [FS] (ASCII: Field Separator)

Messaging profile

The analyzer supports the Basic Profile as described in Appendix B, Section 4.1 of *POCT1-A2 Point-of-Care Connectivity - Approved Standard Second Edition*, standardized under CLSI Vol. 26 No. 28. The analyzer uses the *Synchronous Acknowledgment* connection profile – all conversations have to be synchronous.

The analyzer does not support Continuous Mode (described in Section 4.2 of the same appendix) or Asynchronous Observation Acknowledgments (described in Section 4.3 of the same appendix).

Communication initialization

Periodic communication

The analyzer periodically establishes a connection with the DMS, according to the value configured in the Connectivity interval setting:

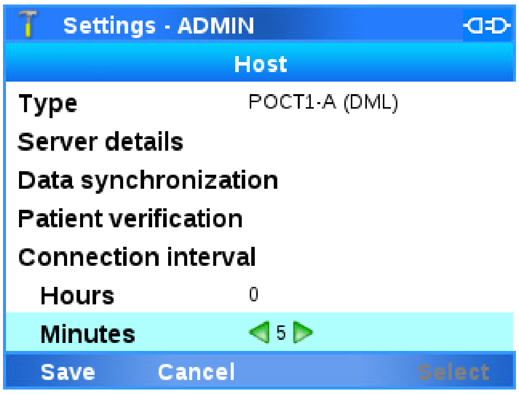

The interval allows a programmed periodic communication with the DMS that can be configured from 5 minutes to 24 hours. The interval starts counting as soon as the connection is established with the DMS. Most DMS “conversations” should fit on the lowest value for the interval (5 minutes), nevertheless, the true purpose of this programmed interval is to keep the DMS updated with the analyzer’s data as often as it is required by the POCC.

After a restart of the analyzer, a user must log on to enable connectivity of the analyzer. Afterwards, the analyzer can establish connections by itself without a user being logged on. Consider this when setting up the connection interval.

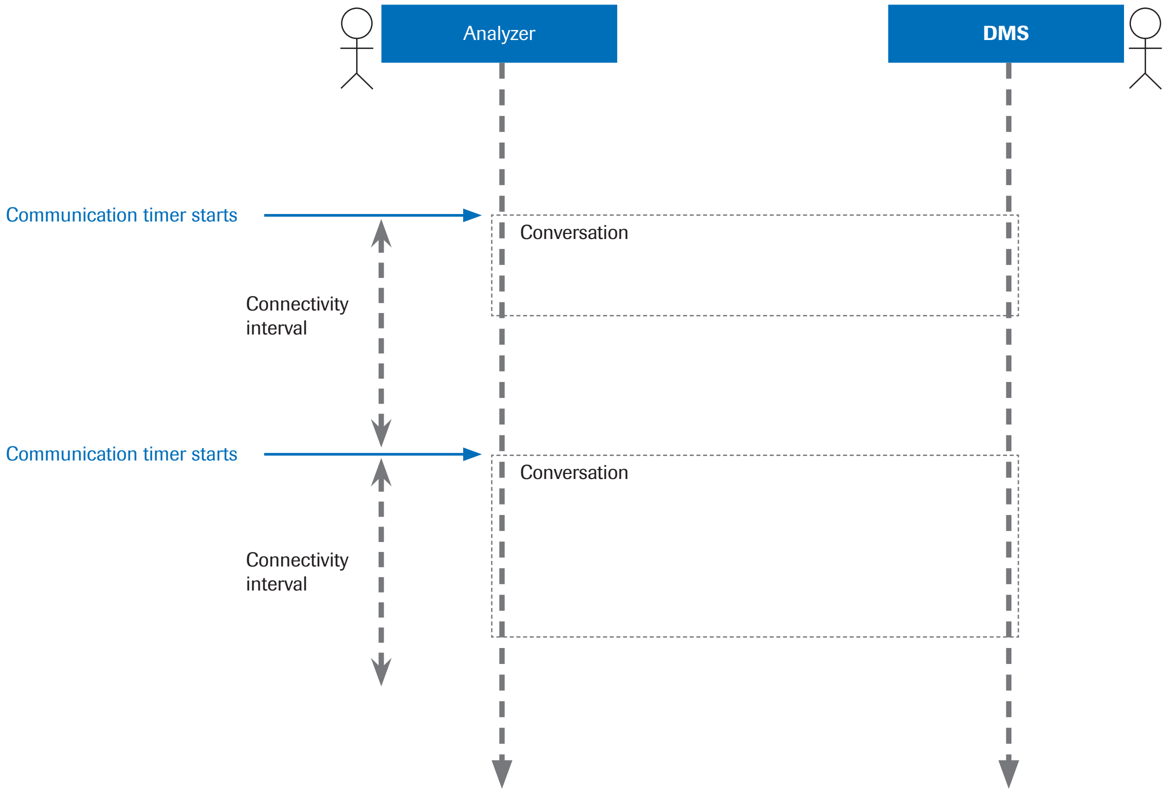

Periodic communication and connectivity interval

This periodic interval communication can be interrupted by a “forced communication”.

Forced communication

Besides the configured periodic communication, the analyzer forces a connection with the DMS when it enters or leaves the “Busy” state. The analyzer is in “Busy” state during the following processes:

- An assay run is being executed.
- A lot validation is being performed.
- Any of the import, export, or archive functionalities is triggered from the **Tools** menu.
- The software update process is being executed.
- A result is sent manually from the user interface.
- When a problem report (manually triggered or scheduled) is executed.

For detailed information on any of the above processes, refer to the **cobas**® Liat® System User Guide.

A “forced communication” is basically a connection that the analyzer establishes with the DMS host outside of the periodic intervals explained in the previous section, as it is depicted in the following figure.

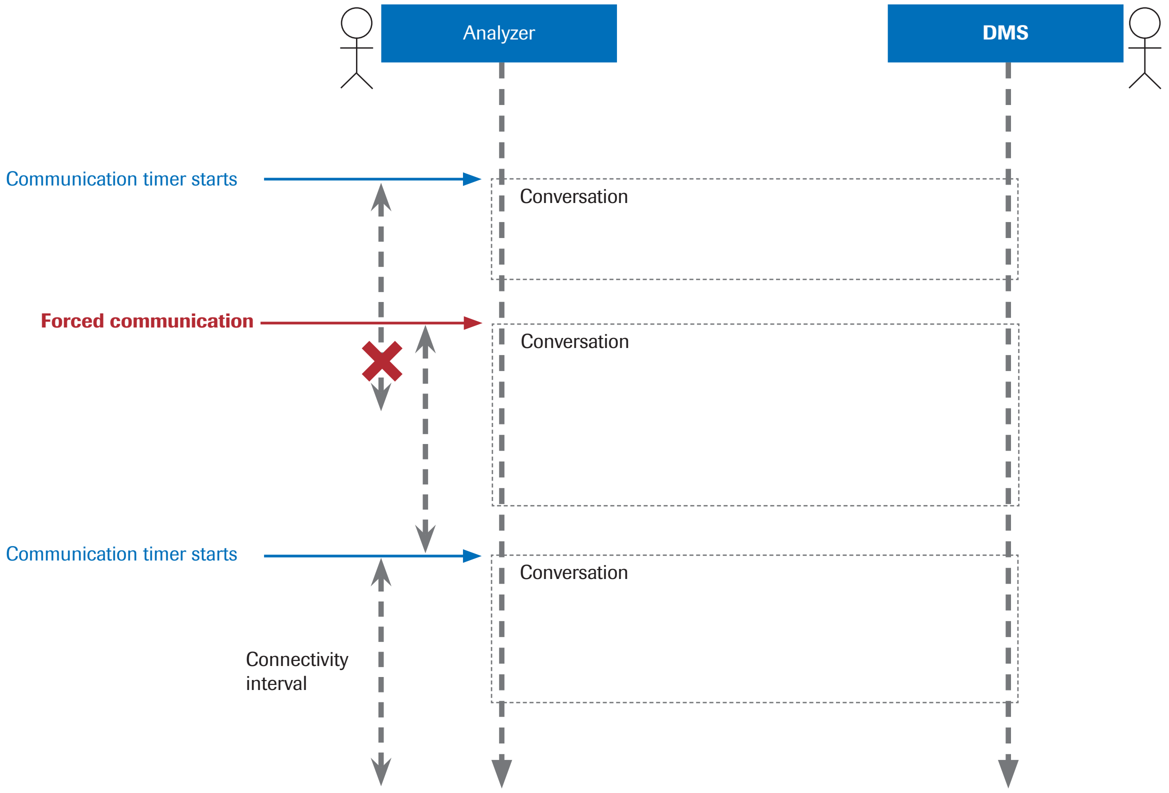

Communication initialization with forced communication

If one of the described user actions (entering/exiting the "Busy" state) starts (tries) a "forced communication" while there is an ongoing communication, the "forced communication" takes place as soon as the current one finishes.

For more information about when and how the communication is terminated, refer to the section Communication termination (28) in this manual.

Initialization flow

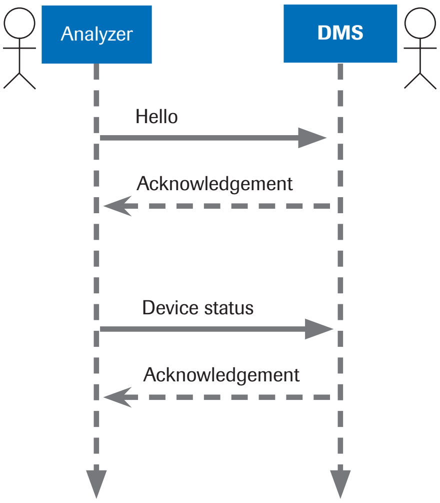

As soon as a connection is established between an analyzer and a DMS, the device starts a conversation by sending a Hello message and wait for an Acknowledgment message sent by the DMS. All established connections (sockets) are always started (opened) from the analyzer.

After receiving a valid Acknowledgment message in response to a Hello message, the analyzer sends the Device status and is waiting again for an Acknowledgment.

If the analyzer does not receive a positive Acknowledgment message in response to its Hello message or its Device status message, the device will immediately disconnect from the DMS by tearing down the lower-level link (without sending a Terminate message).

Communication termination

After a conversation has been established it can be terminated.

According to POCT1-A2 "Point-of-Care Connectivity - Approved Standard Second Edition" standardized under CLSI Vol. 26 No. 28; refer to www.clsi.org, Sect. 4.1.11 abnormal terminations and initiated terminations can be distinguished:

Initiated termination

An *initiated termination* can be initiated from each participant by sending a Terminate message. The recipient of a Terminate message must send an Acknowledgment message.

If the analyzer finished the Terminate topic, the device disconnects from the DMS by tearing down the lower-level link.

It is important that both a DMS as well as an analyzer are prepared to receive and process a Terminate message at any time during a conversation.

Preferred termination

The analyzer under normal circumstances expects to receive the Terminate message from the observation reviewer (the external DMS), as described in the external supporting document, Appendix B, Section 4.1.11.1.

Supporting documents (11)

If the analyzer does not receive a Terminate message after successfully sending the observations in a reasonable amount of time, it will initiate the conversation termination by itself. The same applies to any other topic supported by the analyzer.

Abnormal termination

Under normal circumstances, a conversation is always terminated by the DMS. However, there are situations in which the analyzer is required to terminate an ongoing conversation:

- Operator needs to perform patient tests
- Network connection is lost
- Abnormal program termination

Operator initiated termination

To support this scenario, the analyzer must be allowed to terminate a conversation at any time. It is important for the DMS to be prepared to handle such termination requests promptly; otherwise the analyzer is forced to terminate the lower level TCP/IP connection abruptly.

Network connection loss

Since the TCP/IP-based lower level transport is robust and reliable, network connection loss is detected by the lower level protocol, which informs the application layer. When the analyzer is informed by the lower level protocol of the loss of network connectivity during a conversation, it must clean up the connection and any local states in the context of the current conversation.

Abnormal analyzer program termination

The analyzer will send a protocol termination message if the Software ends abruptly.

There might be scenarios where the analyzer will not be able to send the protocol termination message. In this case it is up to the DMS to implement a Timeout strategy, as suggested by the POCT1-A specification.

Error handling

According to POCT1-A2 "Point-of-Care Connectivity - Approved Standard Second Edition" standardized under CLSI Vol. 26 No. 28; refer to www.clsi.org, Sect. 3.4 *application errors* and *protocol errors* are distinguished.

Application errors

From the communication perspective application errors are the set of errors that occur during the processing of messages.

If an error occurs on the devices side while processing a received valid message, the device returns an Application-error-acknowledgment message. In the message, it informs the DMS why the message has been rejected. At any time, the DMS can also request an error report by sending an Event message.

If an Acknowledgment reports an error condition ("error_detail_cd" attribute value = `AE`), it can include an element (attribute "note_txt") containing an error description. The receiving system may process, log, display, or discard the error description.

Note: The AE acknowledgment is also referred to as “negative acknowledgment”, or NACK.

Errors processing topics (31)

Acknowledgment object (ACK) (70)

When receiving an Application error-acknowledgment in reply to any other POCT message, the device returns an Escape message to finish the current topic.

Escape message (ESC.R01) (110)

Protocol errors

Protocol errors are faults that occur either in the delivery of messages or the receiver can't handle the message. Receiving wrong, unknown or unexpected messages or when the analyzer is in a state where it cannot handle the message (e.g. incompatible with current topic context) causes a protocol error.

All errors that occur in the lower level transport layer i.e. transport errors in the use of the TCP/IP stack are also translated as protocol errors.

Protocol errors are always answered by the analyzer with the Escape message. Table 30 in Appendix B of the POCT1-A Specification POCT1-A2 "Point-of-Care Connectivity - Approved Standard Second Edition" standardized under CLSI Vol. 26 No. 28; refer to www.clsi.org lists the different escape error codes.

Errors processing topics

Whenever the analyzer detects an error after receiving or processing a message/topic, it is stored locally. During the next communication with the DMS, the error is reported as an Event, and it is up to the DMS to request the analyzer Events and receive the error.

Events that represent a detected error while processing messages/topics are reported as "CO.[number]", where number is a 3 digit identifier.

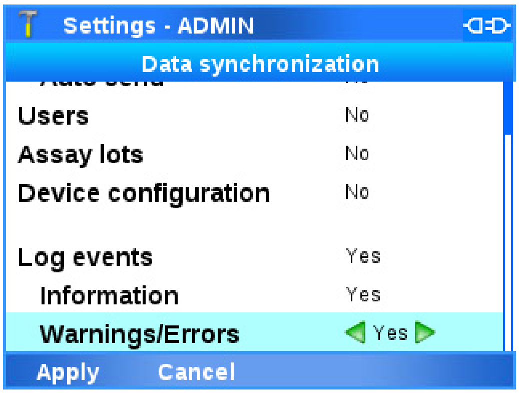

In order to store errors and send them as events to the connected DMS, the analyzer should be configured to do this, as in the screenshot.

Related topics

- Events (55)
- Device events (55)

Application timeout

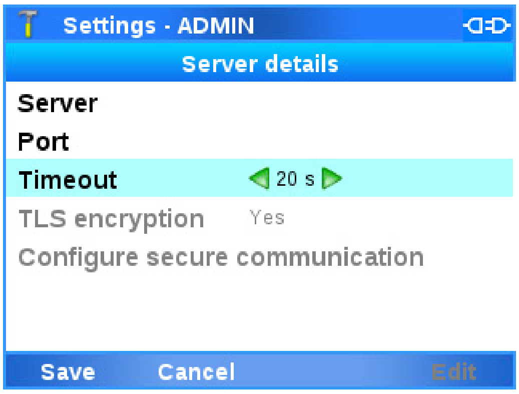

An application timeout occurs when one participant in a Conversation does not send an expected response within a predetermined period. An application timeout is distinct from lower-level timeouts that occur when the data link is broken.

Configure the application timeout in **Settings > Connections > Host > Server details**.

Supported range: 1 - 120 seconds

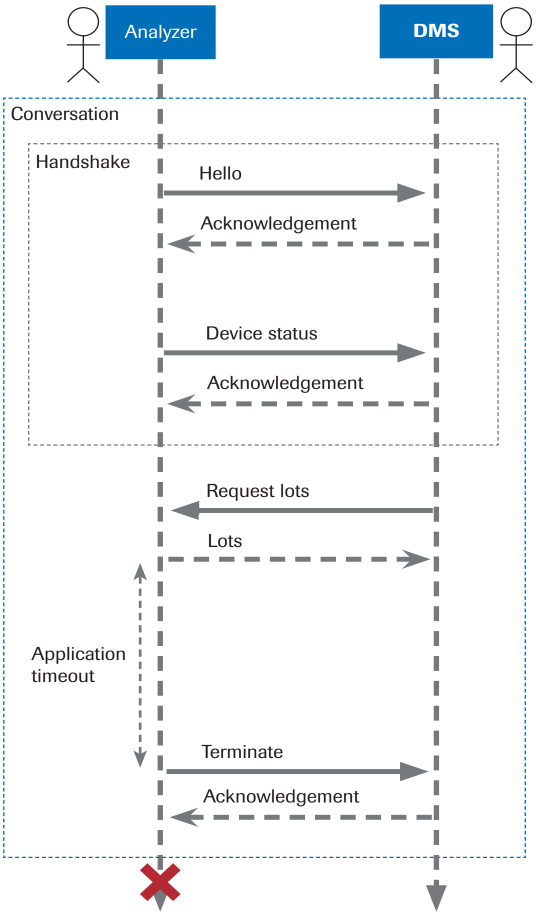

In this scenario, if the analyzer does not receive a final Acknowledgment message after sending the Terminate message, it closes the connection anyway after waiting for a short period of time.

Whenever the timeout is changed via a Device Configuration directive, any active connection with the DMS is reset, and on the next connection the new Timeout is considered.

Keep alive

The analyzer can process Keep alive messages from a connected DMS, but it does not send this kind of message.

The DMS sends a Keep alive message to maintain the connection if it has not received a message within a given period. The analyzer immediately replies with an Acknowledgment message.

Related topics

- Keep alive message (KPA.R01) (111)
- Example: Keep alive message (147)

Supported workflows

In this chapter 2

Workflows 37
Conversations and topics 38
Communication scenarios. 39
Observations (results) 41
Operator and lot lists 44
Update lists 45
Requesting information from the analyzer 46
Requesting operators. 47
About operators 48
Allowed number of users and protected users 49
Validation of operator data 49
Additional attributes of operators. 50
Allowed assays to be executed by a specific operator 51
Trigger for notifying operator changes 51
Lots. 51
Device Configuration 53
Events. 55
Patient verification 63

Workflows

NOTICE

Avoid large data transfers

A simultaneous transfer of large amounts of data to the analyzer can cause it to become inoperable.

- Split large amounts of data into smaller chunks.

Conversations and topics

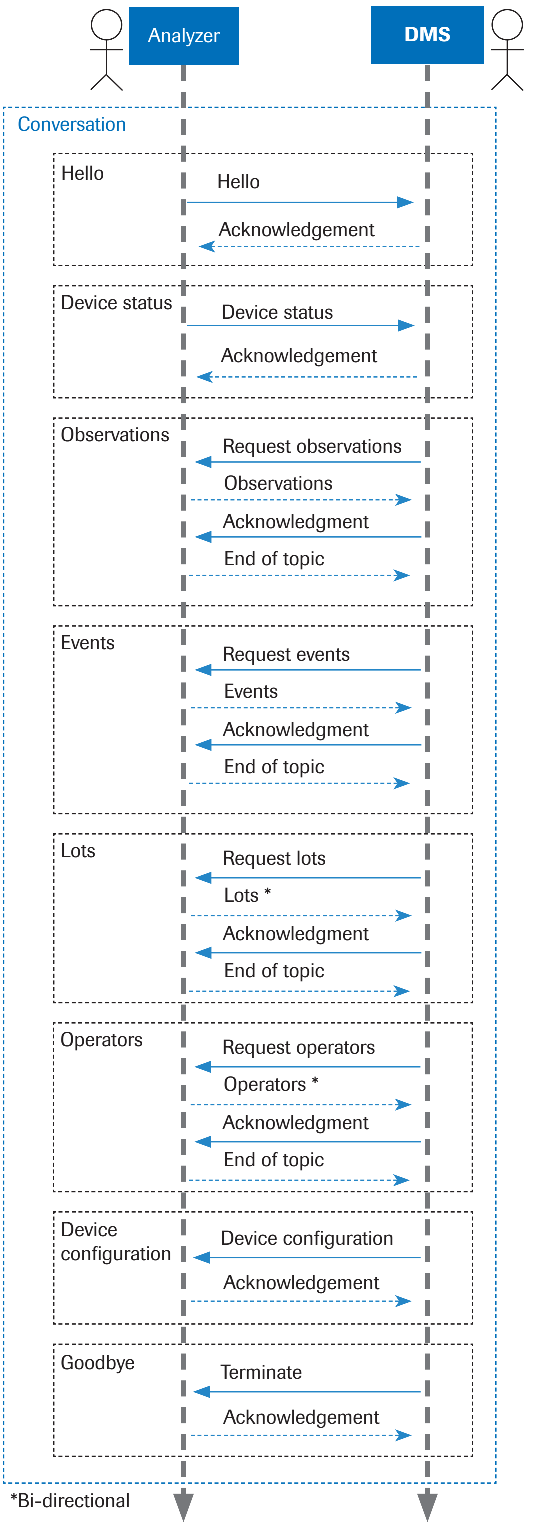

When the analyzer establishes a connection to a remote host (DMS) using the POCT1-A protocol, a “conversation” is started. The analyzer starts the conversation by sending a Hello and a Device status message. The conversation ends when either the DMS or analyzer sends a Terminate message.

Within a conversation, the DMS is able to send several requests to the analyzer:

- Test results (observations)
- QC results (observations)
- Events
- Lots
- Operators
- Patient verification

All the listed requests are standard POCT1-A messages, except the Lots and patient verification messages, which are custom messages for the **cobas**® Liat® Analyzer.

These topics are confirmed with an Acknowledgment message and are finished by an End of topic message. The analyzer can also receive a Device Configuration directive from the host. Directives just receive an “Acknowledgment” (and no “End of topic”).

This communication scenario depicts all the analyzer’s POCT1-A capabilities.

Communication scenarios

The following table lists all the communication scenarios (topics) that are currently supported by the analyzer.

Note: the topics *Operators (update)*, *Lots (update)*, and, *Device Configuration* only work if no user is logged on, or the instrument is in walk-by when the communication is started.

Name	Description	Communication flow	Input	Output
Results	The analyzer sends observations (test results or QC results) to the host:<br>• Automatically after measurement<br>• Or, triggered manually by the operator	Unidirectional: from analyzer to host	• Request from host<br>• Acknowledgment	Test results / observations
Operators	• The host sends a command to synchronize the analyzer's operators with the host.<br>The user must configure the analyzer to enable this.<br>This communication workflow is initiated only when no user is logged on, or the analyzer is in walk-by mode.<br>• The analyzer sends a command to synchronize the operators with the analyzer.	Bidirectional: from/to analyzer from/to host<br><br>New users cannot be added on the device if operators are shared over DMS.	Acknowledgment from host	Operators
Lots	• The host sends a command to synchronize the lots with the host.<br>The user must configure the analyzer to enable this.<br>This communication workflow is initiated only when no user is logged on, or the analyzer is in walk-by mode.<br>• The analyzer sends a command to synchronize the lots with the analyzer.	Bidirectional: from/to analyzer from/to host<br><br>Lots cannot be deleted on the device if lots are shared over DMS.	Acknowledgment from host	Validated Lots

Communication scenarios

Name	Description	Communication flow	Input	Output
Device Configuration	The host sends a command to synchronize the device configuration with the host.<br>The user must configure the analyzer to enable this.<br>This communication workflow is initiated only when no user is logged on, or the analyzer is in walk-by mode.	Unidirectional: from host to analyzer	Acknowledgment from host	Analyzer configuration
Positive Patient ID	The analyzer sends a Hello message to initiate communication with only two values in the device capabilities object	Bidirectional: from/to analyzer from/to host	• Request from host<br>• Acknowledgment	PHI information<br>• ID<br>• Name<br>• Gender
Events	The analyzer sends all relevant events. These events contain Information/Warning/Error messages for the POCC.	Unidirectional: from analyzer to host	• Request from host<br>• Acknowledgment	Device events

Communication scenarios

Observations (results)

The analyzer can generate positive, negative, invalid, indeterminate, or aborted overall results. When the overall result is negative or positive, the individual target results can be sent. When the overall result is aborted, indeterminate and invalid, the individual target results cannot be sent.

The following table shows the communication scenarios as they are currently supported by the analyzer.

Name	Description	Input	Output
Auto send results	The analyzer sends patient results (observations) to the DMS automatically after measurement.	Acknowledgment from DMS	Test results
Send results manually	The user selects patient results (observations) that are stored on the analyzer and triggers the transmission to the DMS.	Acknowledgment from DMS	Test results

Supported communication scenarios

Send results manually

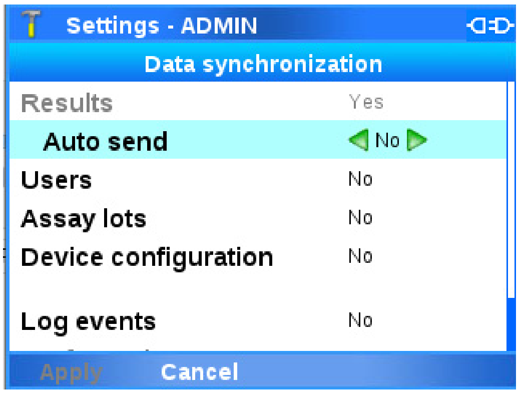

You can configure the analyzer to send results only manually to a connected DMS. In the **Host Settings > Connections > Host > Data Synchronization** screen, set the field **Auto Send** to “**No**”.

On the Results view screen there is an option called “Approval” which allows an operator to either release or reject the result. Rejected results cannot be sent to a DMS. On the other hand, the analyzer sends released results upon request (Results).

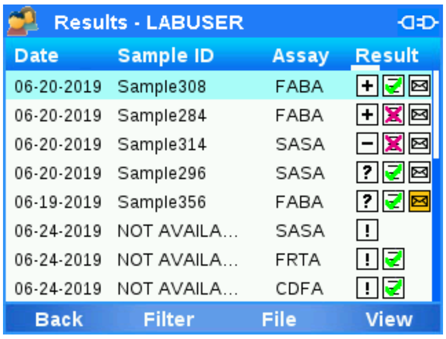

The icons on the right-hand column of the screen indicate the status of the result.

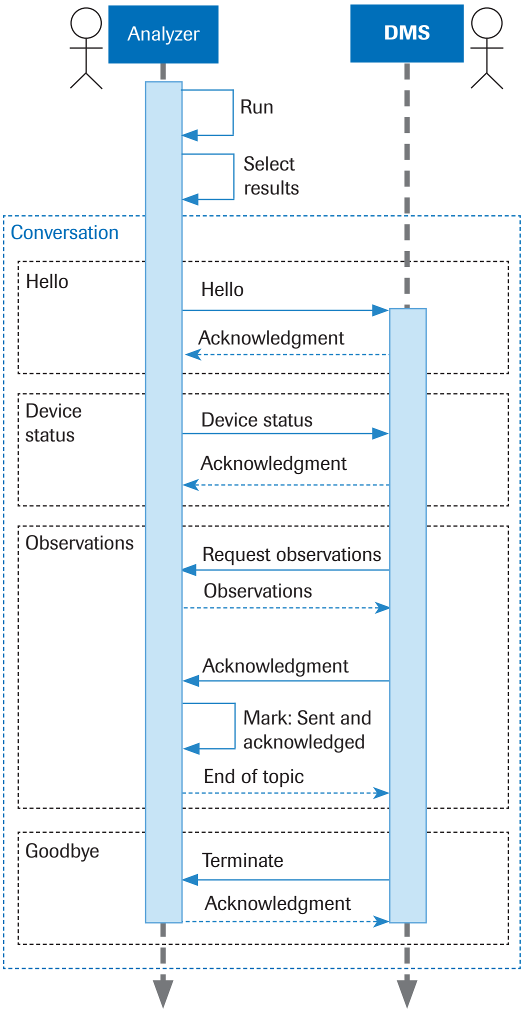

To send a result the operator selects a stored result on the analyzer to be sent to the host and the analyzer waits for a Request observation message, after which it sends the result itself. The host processes the received message, stores the result and sends back an acknowledgment. When the acknowledgment is successfully received by the analyzer, the result is marked by a white envelope indicating “sent to host and acknowledged”.

The acknowledgment is logically linked by the control ID from the Observation message (test result). When the host encounters an error, the acknowledgment contains information about the error condition. When the host does not send an acknowledgment, the analyzer times out. An orange envelope indicates "a message has been sent, but there is no further information about an acknowledgment”. This may be due to an error, or because the message was not accepted by the host, and the host did not send a proper response.

Acknowledgment object (ACK) (70)

Observation object (OBS) (75)

Request object (REQ) (80)

Message structure: Observation messages (OBS) (112)

Examples: Observation topic (134)

Auto send results

You can configure the analyzer to automatically send valid overall results (negative, positive) to the host, once the results are generated, without needing to manually release them.

Configure the **Auto send results** option in the **Host Settings > Connections > Host > Data Synchronization** screen.

Aborted, indeterminate, or invalid overall results are not sent to the host,

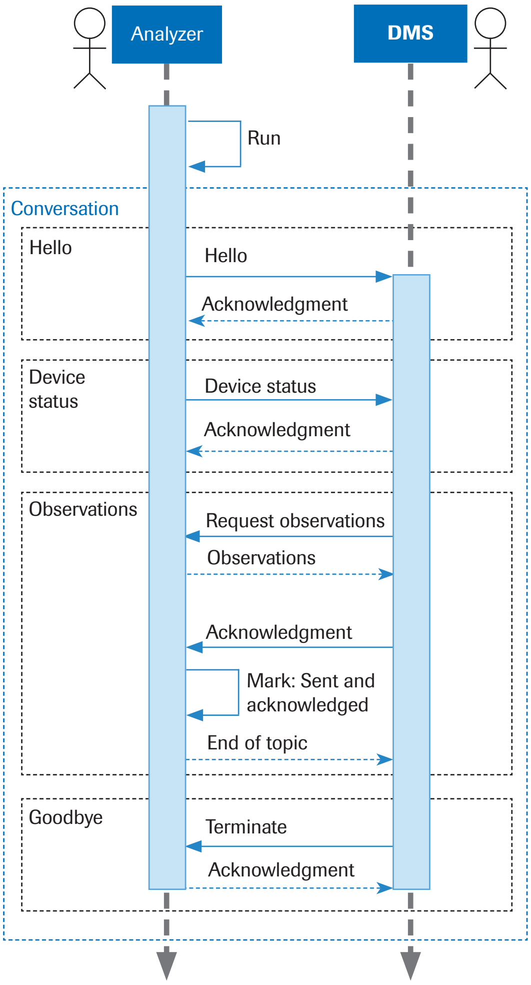

After the analyzer has completed an assay run, it automatically tries to send the result to the host, as follows.

1. The analyzer opens a POCT1-A 'conversation'. In the 'conversation startup' (Hello, Acknowledgment, Device status, Acknowledgment), specifically on the Device status, it is stated if there are new results and/or device events that have not been reported to the POCC.
2. After receiving an Acknowledgment message from the host, the analyzer sends a Device status message to indicate that a new observation (test result) is available. All stored results are reported in the Device status message.
3. If the analyzer receives a Request observation message from the host, it sends the completed result.
4. After the host has processed and stored the result, it sends an acknowledgment
5. After receiving the acknowledgment form the host, the analyzer marks the result by a white envelope indicating “sent to host and acknowledged”.

The acknowledgment is logically linked by the received message control id from the Observation message. When the host encounters an error, the acknowledgment contains information about the error condition. When the host does not send an acknowledgment, the analyzer times out. In either error condition, the analyzer marks the results as “Not accepted”.

If a result is successfully automatically sent, it cannot be sent manually later

Acknowledgment object (ACK) (70)

Observation object (OBS) (75)

Request object (REQ) (80)

Message structure: Observation messages (OBS) (112)

Examples: Observation topic (134)

Operator and lot lists

The analyzer allows the exchange of operators and lots.

- Operator refers to a user who can access and use the analyzer.
- Lot refers to a validated lot implying that the assay tubes have been validated by successfully passing a negative and positive QC run.
- For more information, refer to the **cobas**® Liat® System User Guide.

The Lots message is a custom POCT1-A topic and bidirectional: a POCC can send lots to the analyzer (DMS ➔ Analyzer) and request lots from the analyzer (DMS ← Analyzer).

The operator and lot topics are both POCT1-A update lists.

To be able to receive operators or lots from the DMS, the analyzer must be in "Stand-by" mode. The analyzer enters this mode when no user is logged on, or when the "walk-by" screen is active.

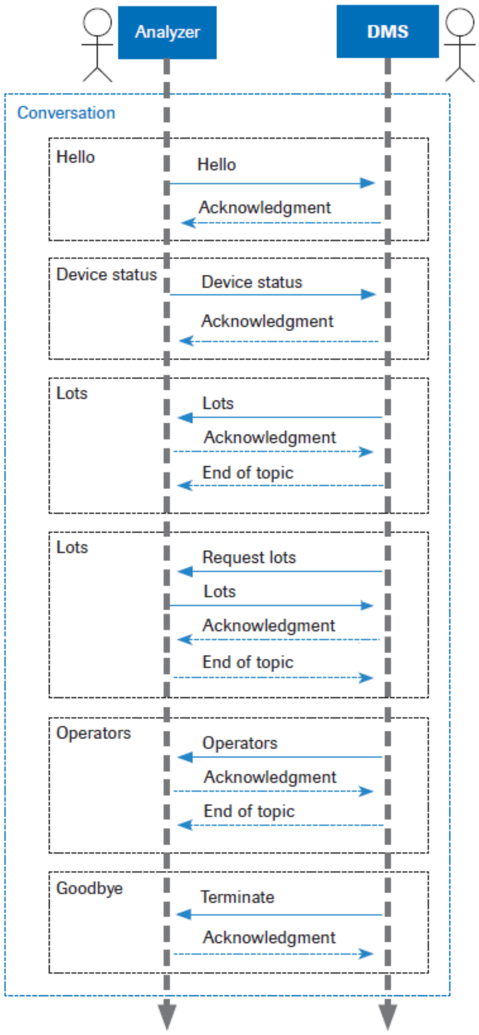

The DMS sends an Acknowledgment message to the analyzer when receiving the Update list message and the analyzer sends an End of topic message.

The example scenario shows a complete conversation that includes topics on "Lots and Operator Lots".

The DMS must send the Update List message and wait for an Acknowledgment message from the analyzer. The DMS must send an End of topic message after receiving the Acknowledgment in response to the message containing the Update List data.

If an analyzer receives an Update List message that it cannot parse or process, it shall respond with a Negative Acknowledge message and a triggered event indicating the error.

- Trigger events (61)
- Acknowledgment object (ACK) (70)
- Operator object (OPR) (78)
- Request object (REQ) (80)
- Message structure: Operator messages (OPL) (114)
- Message structure: Lot full list messages (ROCHE.LIAT.LOTS.R01) (118)
- Message structure: Lot partial list messages (ROCHE.LIAT.LOTS.R02) (119)
- Examples: Lot topic (130)
- Examples: Operators topic (140)

Update lists

There are 2 types of update lists messages:

Full (complete) list

The full update list message provides a complete (full) set of information. If the DMS sends a complete operator list to the analyzer, the analyzer replaces its current list with the one received from the DMS. All operators included in the list are able to access and use the analyzer. Local operators not included in the list received from the DMS are removed and can no longer access and use the analyzer.

Partial (incremental) list

The partial update list message provides a specific (partial) modification of existing information. For example, to update the operator list on the analyzer, the DMS can choose to send a partial list containing the changes since the last update of the analyzer. In a partial list, an operation (insert or delete) must be specified for a particular group of elements. In the following example, operators 11 and 12 are added to the analyzer, and operators 1, 2, and 3 are removed.

Operators

- Add
  - Operator_11, "John Smith"
  - Operator_12, "Jane Doe"
- Delete
  - Operator_1
  - Operator_2
  - Operator_3

To change an existing operator, do the following:

- Send a “Delete” operation.
- Send an “Add” operation with relevant information about the operator. In a partial list, the “Add” operation does not need to follow immediately the “Delete” operation.

For further understanding Update Lists refer to the POCT1-A2 "Point-of-Care Connectivity - Approved Standard Second Edition" standardized under CLSI Vol. 26 No. 28, section 4.1.7 “Operator and Patient Lists”, and section 4.2.5: "Update Lists".

Requesting information from the analyzer

You can request lots and/or operators from the analyzer. You can request full (complete) or partial (incremental) information.

Full data request

For a full data request, the “request_cd” field of the request object must contain the following information:

- `RRDL` for lots.
- `ROPL` for operators.

Partial data request

For a partial data request, the “request_cd” field of the request object must contain the following information:

- `RRDL_D` for lots.
- `ROPL_D` for operators.

The analyzer sends the lots/operators added since the last synchronization with the DMS.

Related topics

- Trigger for notifying operator changes (51)
- Trigger for notifying lot changes (52)
- Trigger events (61)

Requesting operators

If you configure operators to be managed remotely by a DMS, you can no longer manage operators locally.

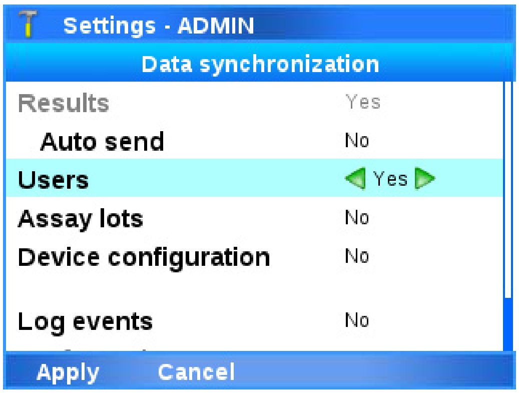

Operators are referred to as users on the user interface. If the **Users** option is set to “Yes”, operators are managed by the DMS.

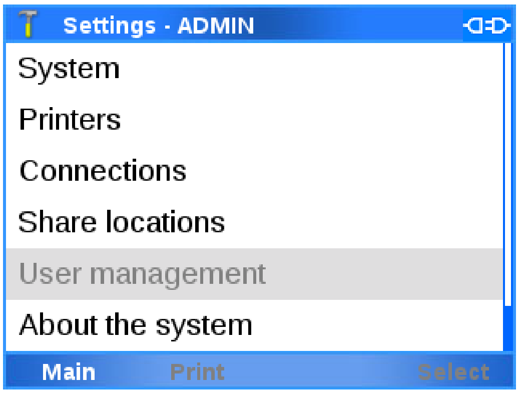

➔ Local user management is disabled.

About operators

All changes on operators must be sent as a full or partial list. No additional commands or messages are supported that insert or delete an operator on the analyzer.

Changes on a specific operator's attribute (like the password) are recommended to be sent via a partial list.

Preinstalled operators

The following operator types are preinstalled: ADMIN, SUPERVISOR, USER1, and USER2

- You cannot delete the ADMIN user via User Interface or DML.
- You cannot modify the role of the ADMIN user.
- You can delete or modify SUPERVISOR, USER1, and USER 2.

Ensure that operator information is provided in the correct format:

- User IDs and passwords defined on the DMS can only contain ASCII printable characters (33-126) except the following which are not supported:
  - Character 34: "
  - Character 39: '
  - Character 96: ` (grave character)
- Note that user names cannot contain special characters. The **cobas**® Liat® Analyzer interface only supports ASCII characters.
- User names are case-insensitive.
- The minimal password length is 4 characters.
- If the host sends a user name or password that uses special characters, the **cobas**® Liat® Analyzer ignores that user name's data.

NOTICE

One account, one user

Shared accounts are a security risk

- Do not allow users to share accounts.

Related topics

- Operator and lot lists (44)
- Additional attributes of operators (50)
- Access control object (ACC) (69)
- Operator object (OPR) (78)
- Reagent object (RGT) (79)
- Message structure: Operator messages (OPL) (114)
- Examples: Operators topic (140)

Allowed number of users and protected users

The analyzer supports up to 500 users to be stored at the same time. If this number is reached, additional operators will not be accepted by the device.

The following table lists the protected users (operator_id of the Operator object (OPR)) defined on the analyzer. These users follow special rules.

The following changes to protected users, received by a full or partial operator list, will not be accepted and will cause a rejection of the message:

- Deletion of the user
- Changes for the “LIAT.Locked” attribute (see Additional attributes of operators in related links)
- Changes for the “permission_level_cd” attribute (see the Access control object (ACC) in related links)

operator_id	Special rules
MANUF / SERVICE	• MANUF and SERVICE (case-insensitive) are protected user defined on each device. They can neither be added, changed nor deleted by a connected DMS system.<br>• DMS messages containing this users will be rejected by the device.<br>• MANUF and SERVICE are never contained in a full operator list requested by a connected DMS system.
ADMIN	• ADMIN (case-insensitive) is a protected user defined on each device.<br>• The ADMIN user can be synchronized with a connected DMS system and will be contained in a full operator list requested by the connected DMS system.

Protected users on the analyzer

Related topics

- Operator and lot lists (44)
- Additional attributes of operators (50)
- Access control object (ACC) (69)
- Message structure: Operator messages (OPL) (114)
- Examples: Operators topic (140)

Validation of operator data

Some fields of an Operator Object (OPR) or a referenced object are validated by the analyzer prior to accepting the message, meaning that the values of those specific fields get validated before the received operator list gets applied on the device. If one of these validations fails, the whole list is rejected by the device.

For additional information on validation rules, consider the details of the following objects:

Access control object (ACC) (69)

Operator object (OPR) (78)

If there was an error with any of the attributes sent from the DMS, this is reported back to the DMS with an Event message.

Event UM.010, see Events (55).

Additional attributes of operators

Operators exchanged with a full (or partial) list can have the following additional information attached as a Note object (NTE).

Attribute	Limited range of values	Comment
LIAT.Contact		Additional optional contact information of the operator
LIAT.Department		Additional information regarding the department of the operator
LIAT.ReadGeneralUserManual	"YES"/"NO"	Defines whether the operator has read the general user manual so that he can use the instrument or not.
LIAT.ChangePasswordOnNextLogin	"YES"/"NO"	Defines whether the operator is requested to change his password at next logon or not
LIAT.Locked	"YES"/"NO"	Indication whether the user is locked, meaning not allowed to log in.<br><br>Protected users cannot be locked. (MANUF, SERVICE, ADMIN)
LIAT.BadgeBarcode		Badge barcode that is used for the authentication of the user if the authentication mode uses the badge barcode.<br><br>LIAT.BadgeBarcode has to be unique across all defined operators.
LIAT.ReadAssayUserManuals	The assay names, or the universal_service_id values. See the link in the related topics below for possible values.	Defines a list of comma separated assay names whose manuals the operator has confirmed reading. Operators who attempt to execute an assay not in this list, receive a prompt instructing them to confirm reading the appropriate manual.

Additional attributes of operators

Related topics

- Values for universal_service_id (SV=1.0) (77)
- Note object (NTE) (83)

Allowed assays to be executed by a specific operator

On the Operators level it can be defined which assays the operator is allowed to execute. The method_cd elements of the Access control object (ACC) define the assays which the operator is allowed to execute.

For each allowed assay, the operator needs to confirm once that he has read the user manual for that specific assay. This information is persisted in a separate node LIAT.ReadAssayUserManuals, assigned to the operator object see Additional attributes of operators.

Related topics

- Additional attributes of operators (50)
- Access control object (ACC) (69)

Trigger for notifying operator changes

Whenever there are changes on operators that could be uploaded to the connected DMS system, the analyzer sends a DMS Event, informing about this change.

Event code	Severity	Event text
TR.002	N	Trigger notification for user data upload to DMS

DMS event trigger for user data

Whenever this device event is received by a connected DMS system, it is recommended to request a partial operator list. This list will contain the operator(s) that changed, along with all its attributes.

Lots

General

All changes on lots must be sent as a full or partial list. No additional commands or messages are supported in order to add or delete a lot on the analyzer.

Changes on a specific attribute of a lot, like the lot validation, are recommended to be sent by a partial list to the analyzer.

A successful exchange (sending / reception) between the analyzer and DMS is acknowledged with a positive Acknowledgment message.

If there was an error with any of the attributes sent from the DMS, this is reported back to the DMS with an Event message.

Event AM.014, see Events (55).

Validation of lot data

Some fields of a Lot Object (LOT) or a referenced object have limitations which are validated. The values of those specific fields get validated before the received lot list is accepted/stored by the analyzer.

If one of the validations fails, the whole list is rejected by the analyzer. Locally validated lots are automatically digitally signed by the analyzer before being sent to an external source (e.g.: DMS, archived lots etc). The signatures of lots received from a DMS are validated before they get applied on the analyzer. The whole list is rejected if the validation fails.

Trigger for notifying lot changes

Similar to the Operator's case, whenever there are new Lots that could be uploaded to the connected DMS system, the analyzer sends a DMS Event:

Event code	Severity	Event text
TR.001	N	Trigger notification for lot data upload to DMS

DMS event trigger for lot data

Whenever this device event is received by a connected DMS system, it is recommended to request a partial lot list. This list will contain the newly validated lots. It is also possible to request a full lot list. In this case, it is up to the DMS to determine the new lots from the received full list.

Related topics

- Operator and lot lists (44)
- Event object (EVT) (74)
- Request object (REQ) (80)
- Lot object (LOT) (103)
- Message structure: Lot full list messages (ROCHE.LIAT.LOTS.R01) (118)
- Message structure: Lot partial list messages (ROCHE.LIAT.LOTS.R02) (119)
- Examples: Lot topic (130)

Device Configuration

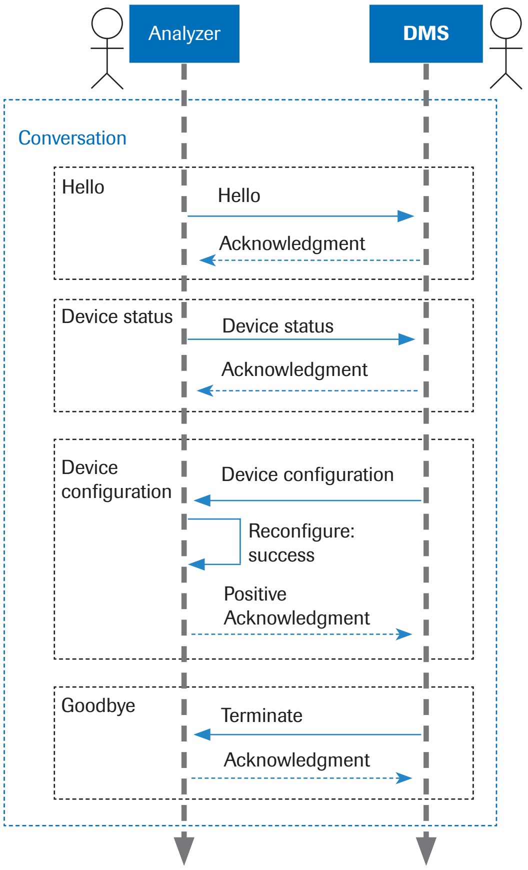

The Device Configuration is a custom **cobas**® Liat® directive designed to configure certain analyzer settings via DML.

To be able to receive a Device configuration message from the DMS, the analyzer must be in "Stand-by" mode, and the **Device configuration** option set to "Yes" (**Host Settings > Connections > Host > Data Synchronization > Device Configuration**). The analyzer enters the Standby mode when no user is logged on, or the "walk-by" screen is active.

The analyzer acknowledges the successful reception by returning a positive Acknowledgment message.

The connected DMS either sends all possible settings or a specific subset. The device then overwrites the local settings with the settings received from the DMS. An administrator can still change the local settings on the device. They are kept for the period configured in the connection interval after which they are overwritten by the device configuration directive received from the connected DMS.

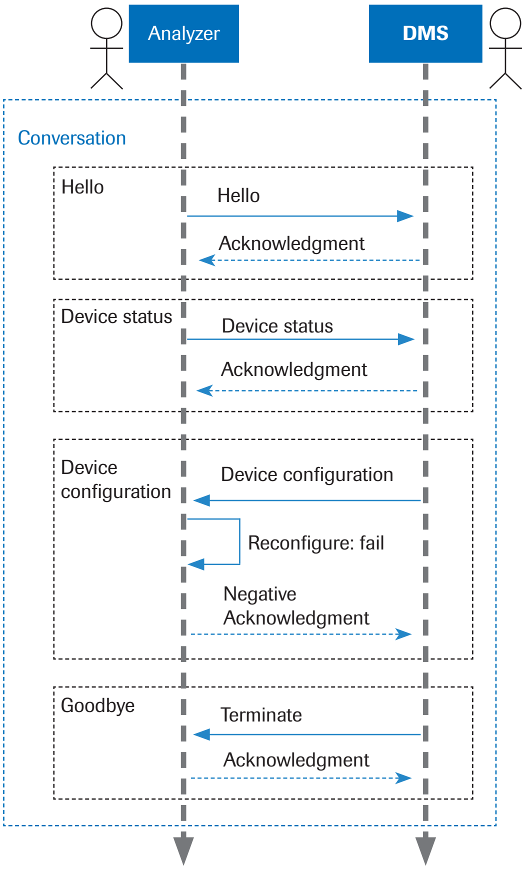

If there was an error with any of the attributes sent from the DMS, this is reported back to the DMS with an Event message:

Event code	Severity	Event text
CO.001	W	Error processing message received from DMS

DMS event to inform about errors

The DMS can detect an error in a "conversation" only by the presence of a Negative Acknowledgment" received from the analyzer.

The Negative Acknowledgment can further contain a "note_txt" attribute with a optional error description providing the reasons of the error.

Related topics

- Additional attributes of operators (50)
- Generic configuration object (GEN_CFG) (88)
- Message structure: Device configuration message (DTV.ROCHE.LIAT.CFG) (116)
- Examples: Device configuration directive (143)

Events

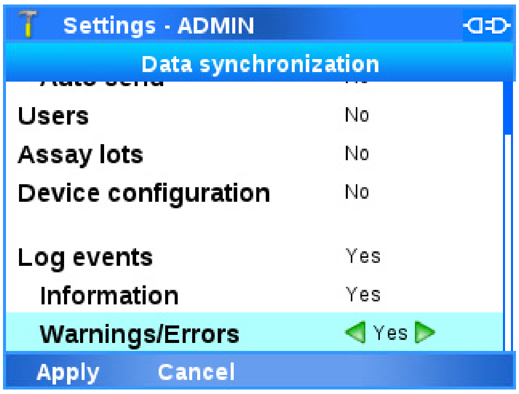

The events inform about the analyzer activity. These events are sent to the DMS when they are enabled (set to “Yes”) on the **Host Settings > Connections > Host > Data Synchronization** screen.

Event categories

Events are categorized either as “Information” or “Warnings/Errors” (Device events). However, the trigger events (local changes on operators and lots) are always sent to a connected DMS, no matter how this attribute is set.

The system stores a log of the events.

- Send and confirm events (acknowledgments) are not stored, but deleted immediately they have been confirmed.
- In order to save space, the analyzer stores at most only the most recent 1000 device events.
- Trigger events are stored separately.

An event log is deleted once the DMS has acknowledged receiving it.

Device events (55)

Trigger events (61)

Device events

The Event code consist of a two digit abbreviation for the function group it belongs to and a three digit number which is unique across the function group. Each device event has assigned one out of three possible severity levels:

Code	Value	Description
N	Note	Indicates information about the normal operation of the Device.
W(1)	Warning	Indicates that the Device has encountered a situation that may affect the normal operation of the Device.
C(2)	Critical	A critical event requires operator intervention to restore normal operation of this Device.<br>On the User interface, a critical event is displayed as “Error”.

Device event severity levels

(1) All received Warning Events should be analyzed by the POCC. Most Warnings either report a malformed/incomplete message (i.e. a POCT1-A Lot message without Lot ID), an alert (i.e. “Service is due in 30 days”) or any non-critical error.

(2) All received Critical Events should be immediately analyzed by the POCC, as the analyzer might not be operational anymore, and Roche service might need to be contacted.

The following table lists all the possible device events:

Function group	Event Code	Severity	Event message	Remarks
Startup System	SS.001	N	Device initialized successfully - no user logged on	
	SS.002	C	Error during initialization - not possible to run assays	
	SS.003	C	Registration period for Software version [SW version] has expired - not possible to run assays	
	SS.004	C	Registration period for Assay(s) [assay name(s)] has expired - assays cannot be used	
	SS.005	W	Software not registered - [nn] days left for registration	
	SS.006	W	Assay(s) [assay name(s)] expired - [nn] days left for registration	

Device events

Function group	Event Code	Severity	Event message	Remarks
Access Control	AC.001	N	User [user id] logged on with authentication mode [mode]	
	AC.002	N	User [user id] logged off	
	AC.003	N	User [user id] has confirmed the action [action]. The current authentication mode is [auth_mode]	ADMIN/SUPERVISOR confirms an assay installation
	AC.005	N	User [user id] has confirmed the action [action]. The current authentication mode is [auth_mode]	ADMIN/SUPERVISOR confirms a run abortion
	AC.007	N	Device unlocked by user [user id]	
	AC.008	N	Device locked automatically due to inactivity	
	AC.009	N	Device locked by user [user id]	
	AC.010	N	User [user id] logged off by user [user id]	
	AC.011	N	Unsuccessful login by User [user id]	
	AC.013	N	Unsuccessful login with an unknown badge	
	AC.014	N	Not possible to unlock User [user id] due to account changes by remote system.	
	AC.015	N	User [user id] has confirmed the action [action].	ADMIN/SUPERVISOR confirms a software update
	AC.016	N	Instrument has been reset	
	AC.017	N	User [MANUF] has confirmed the action [ImportLots Merge]. The current authentication mode is [User ID & Password]	
Run Assay	RA.001	N	Assay [assay name run id tube id] started by user [user id]	
	RA.002	N	Assay [assay name run id] finished successfully	
	RA.003	N	Assay [assay name run id] aborted by user [user id]	
	RA.004	W	Assay [assay name run id] aborted by system	If sample ID is entered manually.
	RA.008	N	Assay [assay name, run id] sample id entered manually started by user [user id]	If Sample ID is entered manually
	RA.009	W	Assay [assay name, run id, tube id] started without patient verification by user [user id]	Given ID could not be verified by the host
	RA.010	N	Assay [assay name, run id, tube id] started with ignoring the patient verification by user [user id]	Operator has canceled and/or ignored the patient verification and the result has been created without verification
Assay Management	AM.001	N	Lot(s) [Lot ID] validated by user [user id]	
	AM.002	N	Assay(s) [Assay name version] installed/updated from USB key by user [user id]	
	AM.003	N	Assay(s) [Assay name version] installed/updated from Axeda by user [user id]	
	AM.004	N	Assay(s) [Assay name version] installed/updated from Share Folder by user [user id]	
	AM.005	N	Assay(s) [Assay name version] installed/updated from FTP by user [user id]	
	AM.006	N	Assay(s) [Assay name version] activated by user [user id]	

Device events

Function group	Event Code	Severity	Event message	Remarks
	AM.007	N	Assay(s) [Assay nameversion] activated by system	
	AM.008	N	Assay(s) [Assay name] deleted by user [user id]	
	AM.009	N	Lot [Lot ID] deleted by user [user id]	
	AM.013	N	[Count] lots successfully synchronized with remote system	
	AM.014	W	Lot synchronization with remote system failed. [Count] successful [Count] unsuccessful	
	AM.016	N	Export Assay Lots started by user [user id]. The export mode is [export-mode]. Share lot is [use share lot]	
	AM.017	N	Export Assay Lots finished successfully by user [user id]	
	AM.018	N	Export Assay Lots aborted by system	
	AM.019	N	Export Assay Lots aborted by user [user id]	
	AM.020	N	Import Assay Lots started by user [user id]. The import mode is [import-mode]. Share lot is [use share lot]	
	AM.021	N	Import Assay Lots finished successfully by user [user id]	
	AM.022	N	Import Assay Lots aborted by system	
	AM.023	N	Archive Assay Lots started by user [user id]	
	AM.024	N	Archive Assay Lots finished successfully by user [user id]	
	AM.025	N	Archive Assay Lots aborted by system	
	AM.026	N	Archive Assay Lots aborted by user [user id]	
Result Management	RM.001	N	Result [result id] released by user [user id]	
	RM.002	N	Result [result id] was rejected by user [user id]	
	RM.003	N	Result [result id result state] sent to [host] by user [user id]	
	RM.004	N	Result [result id result state] sent to [host] by system	
	RM.007	N	Result [result id] was transferred into [media] by user [user id]	
	RM.008	N	Failed sending result [result_id] to host [host] by user [user_id]	
	RM.009	N	Failed sending result [result_id] to host [host] by system	
	RM.010	N	Archive Results started by user [user id]	
	RM.011	N	Archive Results finished successfully by user [user id]	
	RM.012	N	Archive Results aborted by system	
	RM.013	N	Archive Results aborted by user [user id]	
	RM.014	N	Archive and Delete Results started by user [user id]	
	RM.015	N	Archive and Delete Results finished successfully by user [user id]	
	RM.016	N	Archive and Delete Results aborted by user [user id]	
	RM.017	N	Archive and Delete Results aborted by system	

Device events

Function group	Event Code	Severity	Event message	Remarks
System Configuration	SC.003	N	’System' settings changed by user [user id]	
	SC.004	N	’Network ' settings changed by user [user id]	
	SC.005	N	Software Update started from USB key by user [user id]	
	SC.006	N	Software Update started from download by user [user id]	
	SC.007	N	Software Update from [version] to [version] finished successfully	
	SC.008	W	Software Update aborted by system. Reason: [Reason]	
	SC.009	N	Software [version] activated by [user id]	
	SC.010	N	Software [version] activated by system	
	SC.011	N	The received [value] value on the Device Configuration [DeviceConfiguration.Setting] attribute is out of range. The following value was assigned instead: [new value]	
	SC.013	N	‘Host’ settings changed by user [user id]	
	SC.014	N	‘Share Locations’ settings changed by user [user id]	
	SC.015	N	‘Remote Service' settings changed by user [user id]	
	SC.016	N	Export settings started by user [user id]	
	SC.017	N	Export settings finished successfully by user [user id]	
	SC.018	N	Export settings aborted by system	
	SC.019	N	Export settings aborted by user [user id]	
	SC.020	N	Import settings started by user [user id]	
	SC.021	N	Import settings finished successfully by user [user id]	
	SC.022	N	Import settings aborted by system	
	SC.023	N	‘Printers’ settings changed by user [user id]	
	SC.024	N	The following settings parameters are unknown and could not be applied: [List of ignored settings]	

Device events

Function group	Event Code	Severity	Event message	Remarks
Tools	TO.018	N	Display calibrated by user [user id]	
	TO.019	N	Archive Audit Trail started by user [user id]	
	TO.021	N	Archive Audit Trail aborted by user [user id]	
	TO.022	N	Archive Audit Trail aborted by system	
	TO.023	N	Archive and Delete Audit Trail started by user [user id]	
	TO.024	N	Archive and Delete Audit Trail finished successfully by user [user id]	
	TO.025	N	Archive and Delete Audit Trail aborted by system	
	TO.026	N	Archive and Delete Audit Trail aborted by user [user id]	
	TO.027	N	Problem Report creation started by user [user id]	
	TO.028	N	Manual Problem Report creation aborted by user [user id]	
	TO.029	N	Manual Problem Report creation aborted by system	
	TO.030	N	Manual Problem Report creation finished successfully by user [user id]	
	TO.031	N	Problem Report creation started by system	
	TO.032	N	Automatic Problem Report creation aborted by user [user id]	
	TO.033	N	Automatic Problem Report creation aborted by system	
	TO.034	N	Automatic Problem Report creation finished successfully by system	
	TO.035	N	Backup instrument started by user [user id]	
	TO.036	N	Backup instrument finished successfully by user [user id]	
	TO.037	N	Backup instrument aborted by system	
	TO.039	N	Restore instrument started by user [user id]	
	TO.040	N	Restore instrument finished successfully by user [user id]	
	TO.041	N	Restore instrument aborted by system	

Device events

Function group	Event Code	Severity	Event message	Remarks
User Management	UM.001	N	Password changed for user [user id] by user [user id]	
	UM.002	N	Access Badge assigned for user [user id] by user [user id]	
	UM.003	N	User [user id] locked by user [user id]	
	UM.004	N	User [user id] unlocked by user [user id]	
	UM.005	N	User [user id] added by user [user id]	
	UM.006	N	User [user id] modified by user [user id]	
	UM.007	N	User [user id] deleted by user [user id]	
	UM.008	N	Access Badge unassigned from user [user id] by user [user id]	
	UM.009	N	[Count] users successfully synchronized with remote system	
	UM.010	W	User synchronization with remote system failed. [Count] successful [Count] unsuccessful	
	UM.011	N	All users deleted by user [user id]	
	UM.013	N	Export users started by user [user id]	
	UM.014	N	Export users finished successfully by user [user id]	
	UM.015	N	Export users aborted by system	
	UM.016	N	Export users aborted by user [user id]	
	UM.017	N	Import users started by user [user id]	
	UM.018	N	Import users finished successfully by user [user id]	
	UM.019	N	Import users aborted by system	
System Management	SM.001	N	Auto-Calibration [Motor] done	
	SM.002	N	Auto calibration [Photometer] done	
	SM.005	N	The data encryption is initialized successfully	
	SM.006	N	Auto-reboot configured at [HH:mm] was executed at [HH:mm:ss]	
Internal Functions	IF.001	N	Assay [assay name] executed via service backdoor by user [user id]	
Trigger events	TR.001	N	Trigger notification for lot data upload to DMS	
	TR.002	N	Trigger notification for user data upload to DMS	
Connectivity events	CO.001	W	[Root cause error]	

Device events

Trigger events

The trigger events are notifications (for the connected DMS) reporting changes on operators or lots that are ready to be requested (from the connected DMS). The table below lists the 2 possible trigger events:

Event code	Severity	Event text
TR.001	N	Trigger notification for lot data upload to DMS
TR.002	N	Trigger notification for user data upload to DMS

DMS event trigger for lot data

These trigger events are always sent, even if the Events topic was disabled on the Data Synchronization screen.

Related topics

- Additional attributes of operators (50)
- Event object (EVT) (74)
- Message structure: Event message (EVS.R01) (109)

Patient verification

The patient verification workflow requests patient information from the DMS. The analyzer initiates the workflow by sending a Hello message containing the following 2 values in the device capabilities object (DCP): `ROCHE.LIAT.PVI` and `ROCHE.LIAT.PVR`.

Patient verification workflow

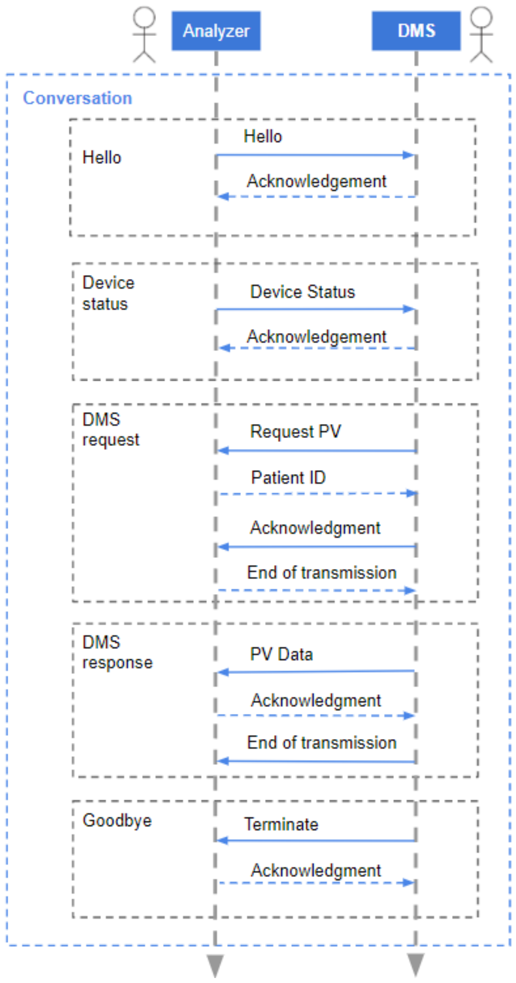

- To initiate the workflow, the analyzer sends a Hello message to the DMS with the device capabilities object values `ROCHE.LIAT.PVI` and `ROCHE.LIAT.PVR`.
- To request patient information from the analyzer, the DMS sends a request message (REQ.R01) with the value request_cd V= `RPVI` in the request object.
- The analyzer returns the patient information to the DMS by sending a patient verification request message (ROCHE.LIAT.PVI.R01) including a patient verification object containing type and value of the patient identifier.
- The DMS checks whether the requested patient identifier matches with a patient record in its database and returns a patient verification response message (ROCHE.LIAT.PVR.R01).
  - If the patient ID matches, the DMS returns a patient verification found object with status `T` (true) and a patient object with the patient demographics.
  - If the patient ID does not match, the DMS returns a patient verification found object with status `F` (false).

The patient verification workflow is a high priority workflow as the user actively waits for a reply. To minimize the waiting time, the DMS should request the patient verification message ROCHE.LIAT.PVI.R01 immediately. During a patient verification workflow, any other topic is rejected.

Unexpected response handling

Response received	Expected handling
The DMS initiates any other communication topic not supported by the workflow.	The communication topic is rejected.
Request for other data (including results) while in patient verification mode	The request is rejected.
The DMS sends patient verification information/request in a non-patient verification workflow.	The message is rejected.
An unexpected message is received during a patient verification workflow.	The message is rejected.

Unexpected response handling

Response received	Expected handling
Inconsistent data: ROCHE.LIAT.PVR.R01 reports patient not found, but contains data	The message is processed as a valid message.
Mandatory patient ID not received.	A NACK is sent and the communication is closed.<br>Application errors (30)<br>Acknowledgment object (ACK) (70)
More or less patient information as expected is received.	The message is processed as a valid message.
The DMS sends patient verification information before receiving the ROCHE.LIAT.PVI.R01 message from the analyzer.	The message is rejected.

Unexpected response handling

Related topics

- Patient verification identifier object (PVI) (87)
- Patient verification found object (PVF) (87)
- Patient verification request message (ROCHE.LIAT.PVI.R01) (119)
- Patient verification response message (ROCHE.LIAT.PVR.R01) (120)

POCT1-A protocol

3	POCT1-A objects	67
4	Message structure	107

POCT1-A objects

The **cobas**® Liat® System supports a subset of the standard POCT1-A objects, as well as some custom vendor defined objects.

3

In this chapter

Standard POCT1-A Objects 69
Access control object (ACC) 69
Acknowledgment object (ACK) 70
Control / Calibration object (CTC) 71
Device status object (DST) 72
End of topic object (EOT) 73
Escape object (ESC) 74
Event object (EVT) 74
Header object (HDR) 75
Observation object (OBS) 75
Order object (ORD) 77
Operator object (OPR) 78
Reagent object (RGT) 79
Service Object (SVC) 79
Request object (REQ) 80
Device object (DEV) 80
Device capabilities object (DCP) 81
Device static capabilities object (DSC) 82
Termination object (TRM) 83
Note object (NTE) 83
Update Action object (UPD) 84

Custom objects 85
Patient object (PT) 85
Patient verification objects 86
Patient verification identifier object (PVI) 87
Patient verification found object (PVF) 87
Generic configuration object (GEN_CFG) 88

Lot object (LOT) 103
About lot number 104

Standard POCT1-A Objects

Only a minimum subset of objects and attributes from the POCT1-A protocol are used by the analyzer and specified here. To get an overview about all POCT1-A standard objects and all their elements and attributes see POCT1-A2 "Point-of-Care Connectivity - Approved Standard Second Edition" standardized under CLSI Vol. 26 No. 28, appendix B, chap. 5.

Access control object (ACC)

The access control object is a component of the operator messages (OPL.R01, OPL.R02).

Definition

Element	DT	Attribute	Comment
method_cd	SET<CV>(1)	V<br>SN<br>SV	This value indicates an assay name that can be performed by the current operator.<br>The SN attribute is always "ROCHE".<br>The SV attribute defines the version of the element definition.
password	ED	V	This operator's password to access the Device.<br>The following validations for the operator_id are done by the **cobas**® Liat® Analyzer before applying the changes:<br>• The password needs to consist of ASCII alphanumeric characters, symbols and/or spaces. It is case-insensitive.<br>• The minimum length of a password is 4 characters<br>• The maximum length of a password is 20 characters
permission_level_cd	CV	V<br>SN<br>SV	Indicates what roles are authorized to execute the assay name in scope (the ones indicated on the method_cd attribute).<br>For the possible values, see Access Control Permission Level Values (permission_level_cd) (70)<br>The SN attribute is always "ROCHE".<br>The SV attribute defines the version of the element definition.

Access control object (ACC)

(1) The SET data type is used to communicate an unordered collection of related values. This type is represented as a repeating element of the given type.

The permission_level_cd element supports the following Access Control Permissions.

Value	Description
User	• Run authorized assays and view assay tube lots<br>• Change own password and badge barcode
Supervisor	As user, plus:<br>• Review results<br>• Manage users (with security level Supervisor or User)<br>• See up the analyzer (except network settings and data import)<br>• Manage assay tube lots<br>• Install assays
Administrator	As Supervisor, plus:<br>• Network configuration settings<br>• Manage all users<br>• Update assays and software<br>• Register assays and software

Access Control Permission Level Values (permission_level_cd)

Related topics

- Additional attributes of operators (50)
- Operator object (OPR) (78)
- Message structure: Operator messages (OPL) (114)
- Examples: Operators topic (140)

Acknowledgment object (ACK)

The acknowledgment object is a component of the acknowledgment message (ACK.R01).

Negative Acknowledgment

The Negative Acknowledgment can contain an optional error description in the “note_txt” attribute explaining why the analyzer rejected the message.

Examples

```
<ACK>
    <ACK.type_cd V="AA" />
    <ACK.ack_control_id V="549" />
</ACK>
```

Positive ACK (type_cd = AA)

```
<ACK>
    <ACK.type_cd V="AE" />
    <ACK.ack_control_id V="549" />
    <ACK.note_txt V="Update messages are only supported on Standby state."/>
</ACK>
```

Negative ACK (type_cd = AE)

Definition

Element	DT	Attribute	Comment
type_cd	CS	V	Type of the acknowledgment:<br>• "AA" – message received successfully.<br>• "AE" – an error occurred when processing the message.<br>Values acc. POCT1-A2 "Point-of-Care Connectivity - Approved Standard Second Edition" standardized under CLSI Vol. 26 No. 28, appendix B, Table 13.
ack_control_id	ST	V	The control ID of the message sent that this message is in Acknowledgment of
note_txt	ST	V	Text describing the error condition<br>Optional Element

Acknowledgment Object (ACK)

Related topics

- Error handling (30)
- Conversations and topics (38)
- Message structure: Acknowledgment message (ACK.R01) (108)
- Examples: Communication start up topics (127)

Control / Calibration object (CTC)

The Control / Calibration object is a component of the Observations message. It is a subelement of the SVC element and a parent element of the OBS element.

Example

```
<CTC>
    <CTC.name V="SASA control" />
    <CTC.lot_number V="67PZ" />
    <CTC.expiration_date V="2017-04-30T00:00:00+00:00" />
    <CTC.level_cd V="N" SN="ROCHE" SV="1.0" />
    <OBS>
        <OBS.observation_id V="Strep A (SASA)" SN="ROCHE" SV="1.0" />
        <OBS.qualitative_value V="Not Detected" SN="ROCHE" SV="1.0" />
        <OBS.method_cd V="M" />
    </OBS>
</CTC>
```

Definition

Element	DT	Attribute	Comment
name	ST	V	Name of assay + “control” (“FABA control”).

Control / Calibration Object

Element	DT	Attribute	Comment
lot_number	CS	V	Unique identifier of the QC lot.<br>About lot number (104)
expiration_date	TS	V	Date and time that the lot expires. This is the date of expiry of the QC lot.
level_cd	CV	V	For possible values of the attribute `V`, see the table below.<br>Attribute "V" values for Calibration control object level_cd (72)<br>The SN attribute is always "ROCHE".<br>The SV attribute defines the version of the element definition.

Control / Calibration Object

Code (attribute V)	Value	Comment
H	High	High Titer Positive Control
M	Medium	Medium Titer Positive Control
L	Low	Low Titer Positive Control
N	Negative	Negative Titer Negative Control

Attribute "V" values for Calibration control object level_cd

Related topics

- Observations (results) (41)
- About lot number (104)
- Observation object (OBS) (75)
- Message structure: Observation messages (OBS) (112)
- Examples: Observation topic (134)

Device status object (DST)

The device status object is a component of the device status message (DST.R01).

Example

```
<DST>
    <DST.status_dttm V="2017-04-13T12:39:25-04:00" />
    <DST.new_observations_qty V="1" />
    <DST.new_events_qty V="86" />
    <DST.condition_cd V="R" />
</DST>
```

Definition

Element	DT	Attribute	Comment
status_dttm	TS	V	Date and time when this status information was created.

Device Status Object (DST)

Element	DT	Attribute	Comment
new_observations_qty	INT	V	Number of unreported observations (all test results).
new_events_qty	INT	V	Number of unreported events.
condition_cd	CV	V<br>SN<br>SV	For supported values of the attribute `V`, see the table below.<br>Device Status condition_cd (DST.condition_cd.V) (73).<br>The SN attribute is always "ROCHE".<br>The SV attribute defines the version of the element definition.

Device Status Object (DST)

Code (attribute V)	Value	Comment
R	Ready	Device is ready to perform tests.
B	Busy	The device is in the process of running a test.
P	Partial Lock	The system reports a partial lock when one or more assay or the SW registration periods have expired, when an error was encountered during initialization or when the maximum Audit Trail threshold is reached.
S	Standby	The device is capable of running a new test once it has been awakened from this 'idle' mode. 'Idle' mode: no user logged on or walk-by screen is active.

Device Status condition_cd (DST.condition_cd.V)

Related topics

- Initialization flow (27)
- Conversations and topics (38)
- Message structure: Device status message (DST.R01) (108)
- Example: Communication start up topics (127)

End of topic object (EOT)

The end of topic object is a component of the end of topic message (EOT.R01).

Example

```
<EOT>
    <EOT.topic_cd V="OBS" />
</EOT>
```

Element	DT	Attribute	Comment
topic_cd	CV	V	Code denoting the identity of the topic.<br>For topics used by the analyzer, see the table below.

End of Topic Object (EOT)

Topic[1]	Topic Name	Description	Required for POCT1-A Compliance	Roche-specific
OBS	Observations	Device supports Observation Topic’s	Yes	No
EVS	Events	Device supports Event Topic’s	No	No
OPL	Operators	Device supports Operator Topic’s	No	No
ROCHE.LIAT.LOTS	Lots	Device supports Lot Topic’s	No	Yes
ROCHE.LIAT.PVI	Patient verification		No	Yes
RPVI	Patient verification		No	Yes

Topics supported by the analyzer

(1) This is the code to be set in the EOT.topic_cd field of the end of topic message

Related topics

- Conversations and topics (38)
- Additional attributes of operators (50)
- Example: Communication ending (146)

Escape object (ESC)

The escape object is a component of the escape message (ESC.R01).

Element	DT	Attribute	Comment
esc_control_id	ST	V	Message control ID from header of the message to which this escape is a response
detail_cd	CS	V	See POCT1-A2 "Point-of-Care Connectivity - Approved Standard Second Edition" standardized under CLSI Vol. 26 No. 28, appendix B, Table 30 for possible values.
note_txt	ST	V	Additional information about escape reason.<br>Optional Element.

Escape Object (ESC)

Related topics

- Message structure: Escape message (ESC.R01) (110)

Event object (EVT)

The Device event object is a component of the Device events message (EVS.R01).

Element	DT	Attribute	Comment
description	ST	V	Free text description of the event.
event_dttm	TS	V	Time at which the event occurred.
severity_cd	CS		Indication of operator intervention. See table below for supported values.<br>Supported values of severity_cd (75)

Event Object (EVT)

Code	Value	Descripttion
C	Critical	A critical event requires operator intervention to restore normal operation of this Device.
N	Note	Indicates information about the normal operation of the Device.
W	Warning	Indicates that the Device has encountered a situation that may affect the normal operation of the Device in the future.

Supported values of severity_cd

Related topics

- Message structure: Event message (EVS.R01) (109)

Header object (HDR)

The header object is a mandatory component of every message.

Example

```
<HDR>
    <HDR.control_id V="927"/>
    <HDR.version_id V="POCT1"/>
    <HDR.creation_dttm V="2019-08-12T08:37:04-04:00"/>
</HDR>
```

Definition

Element	DT	Attribute	Format	Comment
message_type	CV	V		A code made up of the message name and trigger value. Values for this field may be found in the descriptions of each message.
control_id	ST	V	Range: 1 - 65535	Identifies uniquely the message incrementing the counter by one on every new message. Reset on every new communication.
version_id	ST	V	-	Always "POCT1"
creation_dttm	TS	V	-	Date and time the message was created.

Header Object (HDR)

Related topics

- Conversations and topics (38)

Observation object (OBS)

The observation object is a component of the observation messages (OBS.R01 and OBS.R02).

It is a subelement of the PT or CTC element.

Examples

```
<OBS>
    <OBS.observation_id V="Influenza A (FABA)" SN="ROCHE" SV="1.0" />
    <OBS.qualitative_value V="Detected" SN="ROCHE" SV="1.0" />
```

```
    <OBS.method_cd V="M" />
</OBS>
<OBS>
    <OBS.observation_id V="Influenza B (FABA)" SN="ROCHE" SV="1.0" />
    <OBS.qualitative_value V="Detected" SN="ROCHE" SV="1.0" />
    <OBS.method_cd V="M" />
</OBS>
```

Definition

Element	DT	Attribute	Comment
observation_id	CE	V<br>SN<br>SV	The unique identifier of the result type. It consists of the result type itself, and the script name used for processing and detection (in parenthesis), e.g.: `Influenza A (FABA)`<br>See the tables below for possible values.<br>Observation ID's and script names used by the analyzer (SV=1.0) (76)<br>• The SN attribute is always "ROCHE".<br>• The SV attribute defines the version of the element definition.
qualitative_value	CV	V<br>SN<br>SV	The qualitative result value.<br>See table Values for qualitative_value (SV=1.0) (77) below for possible values.<br>• The SN attribute is always "ROCHE".<br>• The SV attribute defines the version of the element definition.
method_cd	CS	V	See POCT1-A2 "Point-of-Care Connectivity - Approved Standard Second Edition" standardized under CLSI Vol. 26 No. 28; refer to www.clsi.org, appendix B, Table 36.

Observation Object (OBS)

The **cobas**® Liat® System and associated assays are not commercially available in all markets. Please check with local Regulatory affiliate for regulatory status.

The observation ID consists of two parts, the assay, and then in brackets, the name of the script used for processing and detecting the result type.

OBS.observation_id: result type (script name)	Description
Cdiff (CDFA)	*Clostridium difficile* (*C. difficile*)
Influenza A (FABA)	Influenza assay run, type A
Influenza B (FABA)	Influenza assay run, type B
Influenza A (FRTA)	Influenza assay run, type A
Influenza B (FRTA)	Influenza assay run, type B
Influenza A (SCFA)	Influenza assay run, type A
Influenza B (SCFA)	Influenza assay run, type B
RSV (FRTA)	Respiratory syncytial virus
SARS-CoV-2 (SCFA)	Severe acute respiratory syndrome coronavirus 2
Strep A (SASA)	Strep assay run, type A (Group A *Streptococcus*)

Observation ID's and script names used by the analyzer (SV=1.0)

OBS.qualitative_value	Description
Detected	Test result was Positive for this observation
Not Detected	Test result was Negative for this observation
Indeterminate	If the overall result is “+” or “-”, certain assays (e.g. FABA and FRTA) send indeterminate target results to the DMS.

Values for qualitative_value (SV=1.0)

Related topics

- Observations (results) (41)
- Message structure: Observation messages (OBS) (112)
- Examples: Observation topic (134)

Order object (ORD)

The Order object is a component of the Observation message. It is a subelement of the PT element.

Example

```
<ORD>
    <ORD.universal_service_id V="Flu/RSV Assay" SN="ROCHE" SV="1.0" />
</ORD>
```

Definition

Element	DT	Attribute	Comment
universal_service_id	CE	V<br>SN<br>SV	Local identifier for the service provided by these observations.<br>See the table below for possible values.<br>Values for universal_service_id (SV=1.0) (77) .<br>• The SN attribute is always "ROCHE".<br>• The SV attribute defines the version of the element definition.

Order Object (ORD)

The **cobas**® Liat® System and associated assays are not commercially available in all markets. Please check with local Regulatory affiliate for regulatory status.

ORD.universal_service_id
Cdiff
Flu/RSV Assay
Influenza Assay
SARS-CoV-2/Flu Assay
Strep A Assay

Values for universal_service_id (SV=1.0)

Related topics

- Observations (results) (41)
- Observation object (OBS) (75)
- Message structure: Observation messages (OBS) (112)
- Examples: Observation topic (134)

Operator object (OPR)

The operator object is part of the following messages:

- OPL.R01/R01
- EVS.R01
- OBS.R01/R02 (sub-element of the SVC object)

Example

```
<OPR>
    <OPR.operator_id V="ADMIN" />
</OPR>
```

Definition

Element	DT	Attribute	Comment
operator_id	ST	V	The following validations for the operator_id are done by the analyzer before applying the changes:<br>• The operator_id has to be unique across all defined operators.<br>• The operator_id needs to consist of ASCII alphanumeric characters or symbols. Spaces are not allowed.<br>• The minimum length of an operator_id is 1 character.<br>• The maximum length of an operator_id is 20 characters.<br>• The operator_id has to be unique in the system. The uniqueness is defined case insensitive.
name	PN	V	The name of the operator.<br>The following validations for the name are done by the analyzer before applying the changes:<br>• The name can only consist of alphanumeric characters, symbols and spaces.<br>• The maximum length of the user name is 25 characters.<br>• The user name is not mandatory and can also be empty.

The operator object (OPR)

Related topics

- Initialization flow (27)
- Operator and lot lists (44)
- About operators (48)
- Additional attributes of operators (50)
- Message structure: Operator messages (OPL) (114)
- Examples: Operators topic (140)

Reagent object (RGT)

The Reagent object is a component of the Observation message (OBS.R01 and OBS.R02). It is a subelement of the SVC object.

Example

```
<RGT>
    <RGT.name V="FRTA"/>
    <RGT.lot_number V="FRTA^AAJ1^1.28"/>
    <RGT.expiration_date V="2042-09-30T00:00:00+00:00"/>
</RGT>
```

Definition

Element	DT	Attribute	Comment
name	ST	V	The manufacturer’s name for the reagent (e.g.,“Chem 7+”).
lot_number	CS	V	The lot number of the reagent used comprising three components:<br>• Vendor’s name of the reagent<br>• Lot code<br>• Internal version number
expiration_date	TS	V	The date after which the reagent should not be used.

Reagent Object (RGT)

Related topics

- Observations (results) (41)
- Observation object (OBS) (75)
- Message structure: Observation messages (OBS) (112)
- Examples: Observation topic (134)

Service Object (SVC)

The service object is a component of the observation messages (OBS.R01, OBS.R02). It is a parent of the PT, RGT, OBS, ORD, CTC and NTE objects.

Definition

Element	DT	Attribute	Comment
role_cd	CS	V	Type of test. See the table below for possible values
observation_dttm	TS	V	The time the observation (test) was performed.

Service Object (SVC)

role_cd	value	Description
LQC	Liquid QC	Observation(s) from a liquid quality control test
OBS	Observations	Patient test observation(s).

Supported values of the role_cd element

Related topics

- Observations (results) (41)
- Observation object (OBS) (75)
- Message structure: Observation messages (OBS) (112)
- Examples: Observation topic (134)

Request object (REQ)

The request object is a component of every request messages (REQ.R01).

Element	DT	Attribute	Comment
request_cd	CV	V	Code denoting the request. See POCT1-A2 "Point-of-Care Connectivity - Approved Standard Second Edition" standardized under CLSI Vol. 26 No. 28, appendix B, Table 14

Request Object (REQ)

Request(1)	Description	Required for POCT1-A Compliance	Roche-specific	Response Message(s)
ROBS	Request all unsent observations.	Yes	-	OBS.R02, OBS.R01
RDEV	Request all unsent events	No	-	EVS.R01
RRDL	Request complete lot list	No	Yes	ROCHE.LIAT.LOTS.R01
RRDL_D	Request incremental lot list	No	Yes	ROCHE.LIAT.LOTS.R02
ROPL	Request complete operator list	No	No	OPL_R01
ROPL_D	Request incremental operator list	No	No	OPL_R02
RPVI	Request patient verification object	No	Yes	ROCHE.LIAT.PVI.R01

Analyzer Requests

(1) This is the code for the request to be set in the request_cd field of the request object of the REQ.R01 message

Related topics

- Workflows (37)
- Message structure: Request message (REQ.R01) (115)

Device object (DEV)

The device object is a component of the hello message (HEL.R01). It is the parent of the DCP and DSC objects.

Example

```
<DEV>
    <DEV.device_id V="f8:dc:7a:06:27:0c"/>
    <DEV.vendor_id V="ROCHE"/>
    <DEV.serial_id V="M1-E-10063"/>
    <DEV.manufacturer_name V="Roche Molecular Diagnostics"/>
```

```
    <DEV.sw_version V="3.3.0.4027"/>
    <DEV.device_name V="cobasLiat"/>
    <DCP>
        <DCP.application_timeout V="70"/>
        <DCP.vendor_specific>ROCHE.LIAT.LOTS.R01;ROCHE.LIAT.LOTS.R02
        </DCP.vendor_specific>
    </DCP>
    <DSC>
        <DSC.connection_profile_cd V="SA"/>
        <DSC.topics_supported_cd V="D_EV"/>
        <DSC.max_message_sz V="614400"/>
    </DSC>
</DEV>
```

Definition

Element	DT	Attribute	Comment
device_id	ST	V	IEEE EUI-64 string-encoded Device identifier.
vendor_id	ST	V	Vendor-specific unique identifier.
serial_id	ST	V	Vendor-specific unique serial identifier.
manufacturer_name	ON	V	The manufacturer's corporate name.
sw_version	ST	V	The software version number(s) for the Device.
device_name	ST	V	A convenient name for the Device (cobasLiat)

Device object (DEV)

Related topics

- Initialization flow (27)
- Message structure: Hello message (HEL.R01) (110)
- Example: Communication start up topics (127)

Device capabilities object (DCP)

The device capabilities object is a component of the hello message (HEL.R01). It is a subelement of the DEV object.

Example

```
<DCP>
    <DCP.application_timeout V="120" />
    <DCP.vendor_specific>ROCHE.LIAT.LOTS.R01;ROCHE.LIAT.LOTS.R02</DCP.vendor_specific>
</DCP>
```

Definition

Element	DT	Attribute	Comment
application_timeout	REAL	V	Application-level timeout this device uses (specified in seconds).
vendor_specific	ED	ENC	Specifies the encoding of the data value. This field can be either "B64" or "TXT".<br>The analyzer always reports its capabilities as text "TXT"<br>Proprietary device Topic capabilities. See table below.

Device capabilities object (DCP)

Code	Description
ROCHE.LIAT.LOTS.R01	Full lot list
ROCHE.LIAT.LOTS.R02	Incremental lot list
DTV.ROCHE.LIAT.CFG	Device configuration message
ROCHE.LIAT.PVI<br>ROCHE.LIAT.PVR	Patient verification workflow

Supported values of vendor_specific element

Related topics

- Initialization flow (27)
- Message structure: Hello message (HEL.R01) (110)
- Example: Communication start up topics (127)

Device static capabilities object (DSC)

The device static capabilities object is a component of the hello message (HEL.R01). It is a subelement of the DEV object.

Example

```
<DSC>
    <DSC.connection_profile_cd V="SA" />
    <DSC.topics_supported_cd V="D_EV" />
    <DSC.max_message_sz V="614400" />
</DSC>
```

Definition

Element	DT	Attribute	Comment
connection_profile_cd	CS	V	CIC messaging profile the Device supports.
topics_supported_cd	SET(CV)	V	The message topics supported. See table below for the topics supported.
max_message_sz	INT	V	The maximum size message (in bytes) that the Device can handle.

Device static capabilities object (DSC)

Code	Topic	Description
OP_LST	Operator List	Device supports Operator List topic.
OP_LST_I	Incremental Operator List	Device supports Incremental Operator List topic.
D_EV	Device Events	Device supports Device Events topic.
DTV	Directives 3	Device supports Directives.
ROCHE.LIAT.<br>PVI	Patient verification request	Device supports patient verification request
ROCHE.LIAT.<br>PVR	Patient verification response	Device supports patient verification response

Supported values of topics_supported_cd element

Related topics

- Initialization flow (27)
- Message structure: Hello message (HEL.R01) (110)
- Example: Communication start up topics (127)

Termination object (TRM)

The terminate object is a component of the terminate messages (END.R01).

Example

```
<TRM>
    <TRM.reason_cd V="ABN" />
    <TRM.note_txt V="Timeout occurred." />
</TRM>
```

Element	DT	Attribute	Comment
reason_cd	CV	V	Values acc. POCT1-A2 "Point-of-Care Connectivity - Approved Standard Second Edition" standardized under CLSI Vol. 26 No. 28, appendix B, Table 54
note_txt	ST	V	Additional information about termination reason<br>Optional Element.

Terminate Object (END)

Related topics

- Communication termination (28)
- Message structure: Termination message (END.R01) (115)
- Example: Communication ending (146)

Note object (NTE)

The note object is a component of the Operator and Escape messages (OPL.R01,OPL.R02, ESC.R01).

The Note object is optional in the Terminate message (END.R01).

Examples

```
<NTE>
    <NTE.text V="LIAT.Use=For In vitro Diagnostic Use" />
</NTE>
<NTE>
    <NTE.text V="LIAT.Run=00016" />
</NTE>
<NTE>
    <NTE.text V="LIAT.Tube=023" />
</NTE>
<NTE>
    <NTE.text V="LIAT.Tube_id=TFRTA170769RC023W" />
</NTE>
<NTE>
    <NTE.text V="LIAT.Approver=ADMIN" />
</NTE>
<NTE>
    <NTE.text V="LIAT.Universal_service_id=Liat Flu/RSV Assay" />
</NTE>
<NTE>
    <NTE.text V="LIAT.Patient_verification_failure_ignored=Run has been performed without patient ve
    rification" />
```

</NTE>

Definition

Element	DT	Attribute	Comment
text	ST	V	A text string. The string's contents are dependent on the context in which the Note object is used.

Note object (NTE)

Related topics

- Operator and lot lists (44)
- About operators (48)
- Escape object (ESC) (74)
- Operator object (OPR) (78)
- Termination object (TRM) (83)
- Message structure: Operator messages (OPL) (114)
- Message structure: Escape message (ESC.R01) (110)

Update Action object (UPD)

The update action method is a component of the operator and lot messages (OPL.R02, ROCHE.LIAT.LOTS.R02).

Element	DT	Attribute	Comment
action_cd	CS	V	Operation to be performed on the item in scope. See the table below for possible values.

Update Action object (UPD)

Code	Value	Description
I	Insert	Insert the specified entries into the associated list.
D	Delete	Delete the specified entries from the associated list.

Supported values of the action_cd element

Related topics

- About operators (48)
- Lots (51)
- Operator object (OPR) (78)
- Lot object (LOT) (103)
- Message structure: Operator messages (OPL) (114)
- Message structure: Lot full list messages (ROCHE.LIAT.LOTS.R01) (118)
- Message structure: Lot partial list messages (ROCHE.LIAT.LOTS.R02) (119)
- Examples: Lot topic (130)
- Examples: Operators topic (140)

Custom objects

In this section

Patient object (PT) (85)
Patient verification objects (86)
Generic configuration object (GEN_CFG) (88)
Lot object (LOT) (103)
About lot number (104)

Patient object (PT)

Definition

Element	DT	Attribute	Comment
patient_id	ST	V	The unique identifier for the patient<br>• If the patient verification workflow is enabled, the received patient ID is sent in the result message (OBS.R01). The scanned patient identifier (sample ID, order ID, or visit ID) is thereby replaced by the ID (PT.patient_id) sent from the DMS to the Liat analyzer.<br>• If the patient verification workflow is disabled, the scanned or manually entered sample ID is sent in the result message (OBS.R01).
name		V	Patient name
birth_date		V	Format = YYYY-MM-DD
gender_cd		V	The analyzer supports the following values:<br>• `F` = female<br>• `M` = male<br>• `U` = unknown,<br>Any other value is interpreted as `U`.

Patient object (PT)

Related topics

- Observations (results) (41)
- Observation object (OBS) (75)
- Message structure: Observation messages (OBS) (112)
- Examples: Observation topic (134)

Patient verification objects

Patient verification identifier object (PVI)

The PVI object is part of the patient verification request message. It contains type and value of the requested patient identifier (patient ID, sample ID, order ID, or visit ID).

Example

```
<PVI>
    <PVI.verification_type_cd V="P” />
    <PVI.identifier V="1234"/>
</PVI>
```

Definition

Element	DT	Attribute	Required	Comment
verification_type_cd	CS	V	Y	Type of patient identifier to be sent.<br>Supported values:<br>• P = patient ID: requested identifier is patient ID<br>• S = sample ID: requested identifier is sample ID<br>• O = order ID: requested identifier is order ID<br>• V = visit ID: requested identifier is visit ID
identifier_id	CS	V	Y	Value of the patient identifier to be matched with the patient details in the DMS database

Patient verification identifier object (PVI)

Related topics

- Patient verification (63)
- Patient verification found object (PVF) (87)
- Patient verification request message (ROCHE.LIAT.PVI.R01) (119)
- Patient verification response message (ROCHE.LIAT.PVR.R01) (120)

Patient verification found object (PVF)

The patient verification found object is part of the response from the DMS to the patient verification request from the analyzer. It informs the analyzer whether the DMS was able to match the requested patient identifier with a patient record in its database.

The response will therefore contain the patient’s full name, gender and date of birth. The “status_cd” element indicates whether the DMS was able to match the record or not.

Example

```
<PVF status_cd V=”T” />
```

Definition

Element	DT	Attribute	Comment
status_cd	CS	V	Supported values:<br>• `T` = True: the requested patient identifier matches to a patient in the DMS database<br>• `F` =False: the requested patient identifier does not match to a patient in the DMS database

Patient verification found object (PVF)

Related topics

- Patient verification (63)
- Patient verification identifier object (PVI) (87)
- Patient verification request message (ROCHE.LIAT.PVI.R01) (119)
- Patient verification response message (ROCHE.LIAT.PVR.R01) (120)

Generic configuration object (GEN_CFG)

The Device configuration object is a component of the Device configuration custom directive.

Definition

Element	DT	Attribute	Comment
DateTime.sntp	CS	V	A boolean that determines if the analyzer uses the SNTP protocol to adjust time.
DateTime.Server	ST	V	The IP address or host name of the SNTP server
DateTime.TimeZone	CS	V	Time Zone. (Case-sensitive string)<br>For possible values, see table below: Supported values of DateTime.TimeZone element (99)
DateTime.DateFormat	CS	V	Date format of the analyzer<br>Supported values:<br>• dd.mm.yyyy<br>• dd/mm/yyyy<br>• dd-mm-yyyy<br>• mm-dd-yyyy<br>• yyyy-mm-dd
DateTime.TimeFormat	CS	V	Time format of the analyzer<br>Supported values:<br>• 12<br>• 24
Display.brightness	CS	V	The brightness of the screen. Values from 0 to 7.<br>When the value given is out of range the system sets the value to the maximum or minimum that can be established. Decimal values are not accepted.
EnableTilt.EnableCheckTilt	CS	V	A boolean that enables/disables the Tilt sensing

Generic configuration object

Element	DT	Attribute	Comment
Sound.sSoundInitialization	CS	V	The sound of the device when it initializes.<br>Supported values:<br>• Off<br>• Beep2<br>• Beep3<br>• Beep4<br>• BUSY<br>• BUZZER<br>• CRITICAL<br>• DEFAULT<br>• DTMF1<br>• EMPTY<br>• EXCLAM<br>• INFEND<br>• LATCHED<br>• ONLINE
Sound.sSoundBarcodeScan	CS	V	The sound of the device when a barcode is scanned.<br>Supported values:<br>• Off<br>• Beep2<br>• Beep3<br>• Beep4<br>• BUSY<br>• BUZZER<br>• CRITICAL<br>• DEFAULT<br>• DTMF1<br>• EMPTY<br>• EXCLAM<br>• INFEND<br>• LATCHED<br>• ONLINE
Sound.sSoundTubeInsert	CS	V	The sound of the device when a tube is inserted.<br>Supported values:<br>• Off<br>• Beep2<br>• Beep3<br>• Beep4<br>• BUSY<br>• BUZZER<br>• CRITICAL<br>• DEFAULT<br>• DTMF1<br>• EMPTY<br>• EXCLAM<br>• INFEND<br>• LATCHED<br>• ONLINE

Generic configuration object

Element	DT	Attribute	Comment
Sound.sSoundAssayFinish	CS	V	The sound of the device when an assay has finished or is aborted.<br>Supported values:<br>• Off<br>• Beep2<br>• Beep3<br>• Beep4<br>• BUSY<br>• BUZZER<br>• CRITICAL<br>• DEFAULT<br>• DTMF1<br>• EMPTY<br>• EXCLAM<br>• INFEND<br>• LATCHED<br>• ONLINE
Sound.sSoundTouchScreen	CS	V	Determines the volume of the sound generated by the device when the user touches the screen.<br>Supported values:<br>• Off<br>• Soft<br>• Loud
Sound.sSoundKeyClicks	CS	V	Determines the volume of the sound generated by the device when the user presses the HW keys.<br>Supported values:<br>• Off<br>• Soft<br>• Loud
Sound.SoundVolume	CS	V	The overall volume of the device. Values range from 0 to 5.<br>When the value given is out of range the system sets the value to the maximum or minimum that can be established. Decimal values are not accepted.
TubeInsertTime.iTubeInsertTime	CS	V	The time allowed to insert the assay tube to start the assay run. Values from 1 to 20 seconds.<br>When the value given is out of range the system sets the value to the maximum or minimum that can be established. Decimal values are not accepted.
Autolock.autolocktime	CS	V	The time at which the screen gets automatically locked. Values from 1 to 1440 minutes.<br>When the value given is out of range the system sets the value to the maximum or minimum that can be established. Decimal values are not accepted.
Authentication.authenticationType	CS	V	The type used to authenticate the user.<br>Supported values:<br>• User ID & Password<br>• Barcode<br>• Barcode & Password

Generic configuration object

Element	DT	Attribute	Comment
Printers.InkLaserName	ST	V	Printer name. It is set automatically when the printer is detected by the analyzer. It cannot be changed on the analyzer.<br>This property is mandatory for configuring a printer via DeviceConfiguration message.<br>If set, the property “Printers.InkLaserConnection” must also be set.<br>Printer settings (98)
Printers.InkLaserDescription	ST	V	Printer description. It is set automatically when the printer is detected by the analyzer. It cannot be changed on the analyzer.<br><br>This property is optional for configuring a printer via DeviceConfiguration message.
Printers.InkLaserLocation	ST	V	Printer location. It is set automatically when the printer is detected by the analyzer. It cannot be changed on the analyzer.<br>This property is optional for configuring a printer via DeviceConfiguration message.
Printers.InkLaserColorMode	CS	V	The color mode defines if the print output is in color or grayscale.<br>Supported values:<br>• grayscale (default)<br>• color
Printers.InkLaserConnection	ST	V	Network connection string of the printer. It is set automatically when the printer is detected by the analyzer. It cannot be changed on the analyzer.<br>This property is mandatory for configuring a printer via DeviceConfiguration message.<br>If set, the property “Printers.InkLaserName” must also be set.<br>Printer settings (98)
Printers.InkLaserUser	ST	V	Optional user name used to connect to the printer when requested by the printer configuration
Printers.InkLaserPassword	ST	V	Optional password used to connect to the printer when requested by the printer configuration.
Printers.ThermalName	ST	V	Printer name. It is set automatically when the printer is detected by the analyzer. It cannot be changed on the analyzer.<br>This property is mandatory for configuring a printer via DeviceConfiguration message.<br>If set, the property “Printers.ThermalConnection” must also be set.
Printers.ThermalDescription	ST	V	Printer description. It is set automatically when the printer is detected by the analyzer. It cannot be changed on the analyzer.<br>This property is optional for configuring a printer via DeviceConfiguration message.
Printers.ThermalLocation	ST	V	Printer location. It is set automatically when the printer is detected by the analyzer. It cannot be changed on the analyzer.<br>This property is optional for configuring a printer via DeviceConfiguration message.<br>Printer settings (98)

Generic configuration object

Element	DT	Attribute	Comment
Printers.ThermalColorMode	CS	V	The color mode defines if the print output is in color or grayscale.<br>Note: None of the analyzer's supported thermal printers can do it in color.<br>Supported value:<br>• grayscale
Printers.ThermalConnection	ST	V	Network connection string of the printer. It is set automatically when the printer is detected by the analyzer. It cannot be changed on the analyzer.<br>This property is mandatory for configuring a printer via DeviceConfiguration message.<br>If set, the property "Printers.ThermalName" must also be set.<br>Printer settings (98)
Printers.ThermalUser	ST	V	Optional user name used to connect to the printer when requested by the printer configuration
Printers.ThermalPassword	ST	V	Optional password used to connect to the printer when requested by the printer configuration.
Printers.ReportPrinting.SelectedPrinter	CS	V	This attribute defines if report printing is enabled or not. To support report printing, an inkjet/laser printer must be configured (either already configured on the analyzer, or on the same DeviceConfiguration message). Otherwise the attribute value must be *not configured*.<br>Supported values:<br>• not configured (default setting)<br>• inklaser
Printers.ResultPrinting.Autoprinting	CS	V	This attribute defines if a result is automatically printed after a run.<br>Supported values:<br>• true<br>• false
Printers.ResultPrinting.SelectedPrinter	CS	V	This attribute defines the printer on which a result is printed. At least one printer must be configured (either already configured on the analyzer, or on the same DeviceConfiguration message). Otherwise the attribute value must be *not configured*.<br>Supported values:<br>• not configured (default setting)<br>• inklaser<br>• thermal
Printers.ResultPrinting.Manualprinting	CS	V	This attribute defines the printer to be used for manual result printing. At least one printer must be configured (either already configured on the analyzer, or on the same DeviceConfiguration message).<br>By setting the value default, the current printer for results (Printers.ResultPrinting.SelectedPrinter) will be used.<br>If multiple printers are configured, the value custom allows the user to manually choose the printer for each print job.<br>Supported values:<br>• default<br>• custom<br>• inklaser<br>• thermal<br>Printer settings (98)
ShareLocations.NetworkShare1.Name	ST	V	This attribute defines the name of the first network share. This could be any name

Generic configuration object

Element	DT	Attribute	Comment
ShareLocations.NetworkShare1.ServerName	ST	V	This attribute defines the server of the first network share. This could be: IP, machine name or DNS name
ShareLocations.NetworkShare1.FolderPath	ST	V	This attribute defines the network path of the first network share. Combining the ServerName and the FolderPath one obtains the typical (Windows) network share:\\MyServer1\Path\One
ShareLocations.NetworkShare1.UserName	ST	V	This attribute defines the user name that can access the first network share
ShareLocations.NetworkShare1.Password	ST	V	This attribute defines the password for the indicated user name
ShareLocations.NetworkShare2.Name	ST	V	This attribute defines the name of the second network share. This could be any name
ShareLocations.NetworkShare2.ServerName	ST	V	This attribute defines the server of the second network share. This could be: IP, machine name or DNS name
ShareLocations.NetworkShare2.FolderPath	ST	V	This attribute defines the network path of the second network share. Combining the ServerName and the FolderPath one obtains the typical (Windows) network share:\\MyServer2\Path\Two
ShareLocations.NetworkShare2.UserName	ST	V	This attribute defines the user name that can access the second network share
ShareLocations.NetworkShare2.Password	ST	V	This attribute defines the password for the indicated user name
ShareLocations.NetworkShare3.Name	ST	V	This attribute defines the name of the third network share. This could be any name
ShareLocations.NetworkShare3.ServerName	ST	V	This attribute defines the server of the third network share. This could be: IP, machine name or DNS name
ShareLocations.NetworkShare3.FolderPath	ST	V	This attribute defines the network path of the third network share. Combining the ServerName and the FolderPath one obtains the typical (Windows) network share:\\MyServer3\Path\Three
ShareLocations.NetworkShare3.UserName	ST	V	This attribute defines the user name that can access the third network share
ShareLocations.NetworkShare3.Password	ST	V	This attribute defines the password for the indicated user name
ShareLocations.FTPShare1.Name	ST	V	This attribute defines the name of the first FTP share.
ShareLocations.FTPShare1.Type	ST	V	This attribute indicates whether to use regular or FTP secure.<br>Supported value:<br>• FTP
ShareLocations.FTPShare1.IP	ST	V	This attribute defines the server of the first FTP share. This could be: IP, machine name or DNS name
ShareLocations.FTPShare1.Port	ST	V	This attribute defines the port of the first FTP share
ShareLocations.FTPShare1.FolderPath	ST	V	This attribute defines the folder path of the first FTP share. Combining the IP and the FolderPath one obtains the typical FTP URL:ftp://ftpserver1/path/one
ShareLocations.FTPShare1.UserName	ST	V	This attribute defines the user name that can access the first FTP share
ShareLocations.FTPShare1.Password	ST	V	This attribute defines the password for the indicated user name
ShareLocations.FTPShare2.Name	ST	V	This attribute defines the name of the second FTP share. This could be any name

Generic configuration object

Element	DT	Attribute	Comment
ShareLocations.FTPShare2.Type	ST	V	This attribute indicates whether to use regular or FTP secure.<br>Supported value:<br>• FTP
ShareLocations.FTPShare2.IP	ST	V	This attribute defines the server of the second FTP share. This could be: IP, machine name or DNS name
ShareLocations.FTPShare2.Port	ST	V	This attribute defines the port of the second FTP share
ShareLocations.FTPShare2.FolderPath	ST	V	This attribute defines the folder path of the second FTP share. Combining the IP and the FolderPath one obtains the typical FTP URL:ftp://ftpserver2/path/two
ShareLocations.FTPShare2.UserName	ST	V	This attribute defines the user name that can access the second FTP share
ShareLocations.FTPShare2.Password	ST	V	This attribute defines the password for the indicated user name
ShareLocations.FTPShare3.Name	ST	V	This attribute defines the name of the third FTP share. This could be any name
ShareLocations.FTPShare3.Type	ST	V	This attribute indicates whether to use regular or FTP secure.<br>Supported value:<br>• FTP
ShareLocations.FTPShare3.IP	ST	V	This attribute defines the server of the third FTP share. This could be: IP, machine name or DNS name
ShareLocations.FTPShare3.Port	ST	V	This attribute defines the port of the third FTP share
ShareLocations.FTPShare3.FolderPath	ST	V	This attribute defines the folder path of the third FTP share. Combining the IP and the FolderPath one obtains the typical FTP URL:ftp://ftpserver2/path/two
ShareLocations.FTPShare3.UserName	ST	V	This attribute defines the user name that can access the third FTP share
ShareLocations.FTPShare3.Password	ST	V	This attribute defines the password for the indicated user name
Connectivity.Timeout	CS	V	The time at which the system will disconnect from the DMS after the last message. Values from 5 to 120 seconds, in increments of 5<br><br>When the value given is out of range the system sets the value to the maximum or minimum that can be established. When the set value is not a multiple of 5, the system rounds it to the closest one. Decimal values are not accepted.
Connectivity.DMLAutosend	CS	V	A boolean which determines if the Auto send option is enabled/disabled
Connectivity.DataSynchronizationUsers	CS	V	A boolean that determines if users are synchronized with DMS
Connectivity.DataSynchronizationAssayLots	CS	V	A boolean that determines if lots are synchronized with DMS
Connectivity.DataSynchronizationLogEvents	CS	V	A boolean that determines if events are synchronized with DMS
Connectivity.DataSynchronizationInformation	CS	V	A boolean that determines if information events are synchronized with DMS
Connectivity.DataSynchronizationErrors	CS	V	A boolean that determines if errors and warning events are synchronized with DMS

Generic configuration object

Element	DT	Attribute	Comment
Connectivity.ConnectionInterval	CS	V	Time interval (in minutes) before the analyzer connects to the DMS after the last message. Values range from 5 to 1440 with increments of 5.
BarcodeITF.Enabled	CS	V	A boolean that determines if the analyzer can read ITF (interleaved 2 of 5) barcodes.
BarcodeITF.Checksum	CS	V	A boolean that determines if analyzer expects the ITF barcodes to have a checksum.
BarcodeITF.FixLength	ST	V	A string that determines if the ITF barcode is a fixed length.<br>Supported values.<br>• `"One discrete length"` (Default)<br>• `"Disabled"` (i.e. ITF barcodes of any length can be read. Note that ITF barcode lengths should always be an even number.)
BarcodeITF.BarcodeLength	CS	V	Integer, that sets the length of the ITF barcode. (Including checksum digit.)
BarcodeCodabar.Enabled	CS	V	A boolean that determines if the analyzer can read Codabar encoded barcodes.
BarcodeCodabar.TransmitStartStopChar	CS	V	A boolean that determines if the Liat barcode scanner, upon reading a Codabar, sends the full barcode to the application, or just the barcode string without the Start/End chars.
BarcodeCode39.Enabled	CS	V	A boolean that determines if the analyzer can read Code 39 barcodes.
BarcodeCode39.Checksum	CS	V	A boolean that determines if analyzer expects the Code 39 barcodes to have a checksum.
BarcodeCode93.Enabled	CS	V	A boolean that determines if the analyzer can read Code 93 barcodes.
BarcodeEAN8.Enabled	CS	V	A boolean that determines if the analyzer can read EAN-8 barcodes.
BarcodeEAN13.Enabled	CS	V	A boolean that determines if the analyzer can read EAN-13 barcodes.
BarcodeGS1Databar14.Enabled	CS	V	A boolean that determines if the analyzer can read GS1 Databar 14-digit barcodes.
Languages.Language	CS	V	Languages supported by the analyzer<br>Supported values:<br>• en-US<br>• de-DE<br>• fr-FR<br>• it-IT<br>• es-ES<br>• da-DK<br>• nl-NL<br>• hu-HU<br>• nb-NO<br>• pl-PL<br>• pt-PT<br>• sv-SE<br>• cs-CZ
PV.Verification	CS	V	A string that determines if the patient verification is performed or not.<br>Supported values:<br>• prior run<br>• no verification

Generic configuration object

Element	DT	Attribute	Comment
PV.VerificationType[(1)]	CS	V	Identifies the type of identifier (patient ID, sample ID, order ID, visit ID) to be sent to the DMS.<br>Supported values:<br>• `P` = patient ID<br>• `S` = sample ID<br>• `O` = order ID<br>• `V` = visit ID
PV.PatientMismatch[(1)]	CS	V	Determines whether a run can be performed when the requested patient identifier does not match with a patient in the database of the DMS.<br>Supported values:<br>• run allowed<br>• run not allowed
PV.DisplayedData[(1)]	CS	V	String that determines the level of detail required for the patient verification record response.<br>Supported values:<br>• `none` = no data<br>• `partial` = name<br>• `verbose` = name, sex, data of birth
PV.ManualConfirmation[(1)]	CS	V	Determines if confirmation by the user is required when the response from the DMS is received.<br>Supported values:<br>• not required<br>• required
RemoteService.HTTPproxy	CS	V	A boolean that determines if the analyzer can connect to a HTTP proxy
RemoteService.Server	ST	V	The IP address or host name of the RemoteService server If RemoteService.HTTPproxy is false, then Server must not be informed.
RemoteService.Port	CS	V	Port used If RemoteService.HTTPproxy is false, then Port must not be informed.
RemoteService.Authentication	CS	V	A boolean that determines if the analyzer must authenticate when connecting to a HTTP proxy
RemoteService.UserName	ST	V	User name identifierIf RemoteService.Authentication is false, then UserName must not be informed.
RemoteService.Password	ST	V	User passwordIf RemoteService.Authentication is false, then Password must not be informed.
AutoReboot.Time	ST	V	Indicates at what time the Liat will perform its daily reboot.<br>• Format: HH:mm<br>• Hour (HH) range: 00-23<br>• Minutes (mm) range: 00-55: Only multiples of 5 are allowed {00, 05, 10, ..., 50, 55}.<br>Default value: `3:00 (3 AM)`
Machine.sMachineName	ST	V	Name of the machine.<br>This name could be used as the host name to access the Liat via network (TCP/IP)<br>Ensure that this attribute is unique for every analyzer in your network!
PRContent.Communicationlog	ST	V	Indicates whether the communication traces (logs) are included or not inside the problem report.<br>Supported values:<br>• `included`<br>• `excluded` (default value)

Generic configuration object

Element	DT	Attribute	Comment
PRContent.DataRange	ST	V	Indicates that the date range of result related data is included in the problem report.<br>Supported values:<br>• `last24hours`<br>• `last7days`<br>• `last30days` (default value)<br>• `all`
PRContent.Runlog	ST	V	Indicates whether the run assay raw data (logs) are included or not inside the problem report.<br>Supported values:<br>• `included`<br>• `excluded` (default value)
PRContent.Users	ST	V	Indicates whether the sample results are included or not inside the Problem Report.<br>Supported values:<br>• `included`<br>• `excluded` (default value)
PRContent.Sampleresults	ST	V	Indicates whether the sample results are included or not inside the problem report.<br>Supported values:<br>• `included`<br>• `excluded` (default value)
PRContent.SampleID	ST	V	Indicates whether the run assay sample IDs are included or not inside the problem report.<br>Supported values:<br>• `included`<br>• `excluded` (default value)<br>The value “included” can only be used if the PRContent.Sampleresults element is also set to “included”.
PRSchedule.Creation	ST	V	Indicates if Problem Reports can be scheduled (automatic) or not (on demand).<br>Supported values:<br>• `on demand` (default value)<br>• `automatic`<br>If set to “automated”, the PRSchedule element must contain a value.
PRSchedule.Frequency	ST	V	Indicates how often problem reports are automatically generated.<br>Supported values:<br>• `daily`<br>• `weekly`<br>• `monthly` (default value)<br>Dependencies:<br>• If set to “weekly”, the PRSchedule.DayOfWeek element must contain a value.<br>• If set to “monthly”, PRSchedule.DayOfMonth must contain a value.<br>• If this element is used (populated by any supported value), the PRSchedule.Time and PRSchedule.Destination elements must contain a value.

Generic configuration object

Element	DT	Attribute	Comment
PRSchedule.DayOfWeek	ST	V	Indicates how often weekly problem reports are automatically generated.<br>Supported values:<br>• `sunday` (default value)<br>• `monday`<br>• `tuesday`<br>• `wednesday`<br>• `thursday`<br>• `friday`<br>• `saturday`
PRSchedule.DayOfMonth	ST	V	Indicates how often monthly problem reports are automatically generated.<br>Supported values:<br>• `firstdayofmonth` (default value)<br>• `lastdayofmonth`
PRSchedule.Time	ST	V	Indicates at what time the problem reports are automatically generated.<br>• Format: HH<br>• Hour (HH) range: 0-23.<br>Default value: `16 (4 PM)`
PRSchedule.Destination	ST	V	Indicates where the automatically generated problem reports are saved.<br>Supported values:<br>• `remoteservicesystem` (default value)<br>• `networkshare1`<br>• `networkshare2`<br>• `networkshare3`<br>• `ftpshare1`<br>• `ftpshare2`<br>• `ftpshare3`<br>**Note:** all networkshare# and ftpshare# correspond to the ShareLocations.NetworkShare# and ShareLocations.FTPShare# defined before in this table

Generic configuration object

(1) Note: If PV.Verification is set to "no verification", this element must not be informed.

Printer settings

Network printers connections configured via DMS (Printers.InkLaserConnection and/or Printers.ThermalConnection) must specify one of the supported printing network protocols:

- "http://"
- "https://"
- "ipp://"
- "lpd://"
- "ipps://"
- "ipp14://"
- "socket://"
- "smb://"
- "dnssd://"

Note: USB printers directly connected to the analyzer via USB port cannot be configured via DMS. These printers can be configured locally on the analyzer, via the printer detection workflow.

All printer settings can be set individually. In other words, it is possible that a DeviceConfiguration message just contains a single Printer setting. The only exception for this rule are the *Printer.[type]Name* and *Printer.[type]Connection* (where “type” is “Thermal” or “InkLaser”) settings: if one of these two is part of the message, the other should be too, otherwise the configuration is rejected.

Cross-referenced printer settings should make reference either to an already configured (analyzer) printer, or to a printer that is configured on the same DeviceConfiguration message. For example, if an instrument does not have an “inklaser” printer configured, and it receives a DeviceConfiguration message with just the following setting:

- Printers.ResultPrinting.Manualprinting = “inklaser”

The message is rejected. Note that if on the same message there is an inklaser configured (*Printers.InkLaserName* and *Printers.InkLaserConnection* are set), the message is accepted.

NOTICE

Incorrect DateTime.TimeZone string causes serious problems

An unsupported string can prevent updates. It can require reinstallation of the software to fix it.

- Make sure the string passed to DateTime.TimeZone is exactly correct, including correct upper and lower case.

Time zone names are case-sensitive.

DateTime.TimeZone	Description (analyzer user interface value)
(UTC+14:00) Kiritimati Island;Line Islands Standard Time	(UTC+14:00) Kiritimati Island
(UTC+13:00) Samoa;Samoa Standard Time	(UTC+13:00) Samoa
(UTC+13:00) Nuku'alofa;Tonga Standard Time	(UTC+13:00) Nuku’alofa
(UTC+12:00) Fiji;Fiji Standard Time	(UTC+12:00) Fiji
(UTC+12:00) Auckland, Wellington;New Zealand Standard Time	(UTC+12:00) Auckland, Wellington
(UTC+12:00) Anadyr, Petropavlovsk-Kamchatsky;Russia Time Zone 11 Standard Time	(UTC+12:00) Anadyr, Petropavlovsk-Kamchatsky
(UTC+12:00) Coordinated Universal Time +12;UTC+12	(UTC+12) Coordinated Universal Time +12
(UTC+11:00) Solomon Is., New Caledonia;Central Pacific Standard Time	(UTC+11:00) Solomon Is., New Caledonia
(UTC+11:00) Sakhalin;Sakhalin Standard Time	(UTC+11:00) Sakhalin
(UTC+11:00) Chokurdakh;Russia Time Zone 10 Standard Time	(UTC+11:00) Chokurdakh
(UTC+10:00) Vladivostok;Vladivostok Standard Time	(UTC+10:00) Vladivostok
(UTC+11:00) Magadan;Magadan Standard Time	(UTC+10:00) Magadan

Supported values of DateTime.TimeZone element

DateTime.TimeZone	Description (analyzer user interface value)
(UTC+10:00) Hobart;Tasmania Standard Time	(UTC+10:00) Hobart
(UTC+10:00) Guam, Port Moresby;West Pacific Standard Time	(UTC+10:00) Guam, Port Moresby
(UTC+10:00) Canberra, Melbourne, Sydney;AUS Eastern Standard Time	(UTC+10:00) Canberra, Melbourne, Sydney
(UTC+10:00) Brisbane;E. Australia Standard Time	(UTC+10:00) Brisbane
UTC+09:30) Darwin;AUS Central Standard Time	(UTC+09:30) Darwin
(UTC+09:30) Adelaide;Cen. Australia Standard Time	(UTC+09:30) Adelaide
(UTC+09:00) Chita;Transbaikal Standard Time	(UTC+09:00) Chita
(UTC+09:00) Yakutsk;Yakutsk Standard Time	(UTC+09:00) Yakutsk
(UTC+09:00) Seoul;Korea Standard Time	(UTC+09:00) Seoul
(UTC+09:00) Osaka, Sapporo, Tokyo;Tokyo Standard Time	(UTC+09:00) Osaka, Sapporo, Tokyo
(UTC+08:30) Pyongyang;North Korea Standard Time	(UTC+08:30) Pyongyang
(UTC+08:00) Ulaanbaatar;Ulaanbaatar Standard Time	(UTC+08:00) Ulaanbaatar
(UTC+08:00) Taipei;Taipei Standard Time	(UTC+08:00) Taipei
(UTC+08:00) Perth;W. Australia Standard Time	(UTC+08:00) Perth
(UTC+08:00) Kuala Lumpur, Singapore;Singapore Standard Time	(UTC+08:00) Kuala Lumpur, Singapore
(UTC+08:00) Irkutsk;North Asia East Standard Time	(UTC+08:00) Irkutsk
(UTC+08:00) Beijing, Chongqing, Hong Kong, Urumqi;China Standard Time	(UTC+08:00) Beijing, Chongqing, Hong Kong, Urumqi
(UTC+07:00) Krasnoyarsk;North Asia Standard Time	(UTC+07:00) Krasnoyarsk
(UTC+07:00) Barnaul, Gorno-Altaysk;Altai Standard Time	(UTC+07:00) Barnaul, Gorno-Altaysk
(UTC+07:00) Bangkok, Hanoi, Jakarta;SE Asia Standard Time	(UTC+07:00) Bangkok, Hanoi, Jakarta
(UTC+06:30) Yangon (Rangoon);Myanmar Standard Time	(UTC+06:30) Yangon (Rangoon)
(UTC+06:00) Novosibirsk;N. Central Asia Standard Time	(UTC+06:00) Novosibirsk
(UTC+06:00) Dhaka;Bangladesh Standard Time	(UTC+06:00) Dhaka
(UTC+06:00) Astana;Central Asia Standard Time	(UTC+06:00) Astana
(UTC+05:45) Kathmandu;Nepal Standard Time	(UTC+05:45) Kathmandu
(UTC+05:30) Sri Jayawardenepura;Sri Lanka Standard Time	(UTC+05:30) Sri Jayawardenepura
(UTC+05:30) Chennai, Kolkata, Mumbai, New Delhi;India Standard Time	(UTC+05:30) Chennai, Kolkata, Mumbai, New Delhi
(UTC+05:00) Islamabad, Karachi;Pakistan Standard Time	(UTC+05:00) Islamabad, Karachi
(UTC+05:00) Ekaterinburg;Ekaterinburg Standard Time	(UTC+05:00) Ekaterinburg
(UTC+05:00) Ashgabat, Tashkent;West Asia Standard Time	(UTC+05:00) Ashgabat, Tashkent
(UTC+04:30) Kabul;Afghanistan Standard Time	(UTC+04:30) Kabul
(UTC+04:00) Yerevan;Caucasus Standard Time	(UTC+04:00) Yerevan
(UTC+04:00) Tbilisi;Georgian Standard Time	(UTC+04:00) Tbilisi
(UTC+04:00) Port Louis;Mauritius Standard Time	(UTC+04:00) Port Louis
(UTC+04:00) Izhevsk, Samara;Russia Time Zone 3 Standard Time	(UTC+04:00) Izhevsk, Samara
(UTC+04:00) Baku;Azerbaijan Standard Time	(UTC+04:00) Baku
(UTC+04:00) Astrakhan, Ulyanovsk;Astrakhan Standard Time	(UTC+04:00) Astrakhan, Ulyanovsk
(UTC+04:00) Abu Dhabi, Muscat;Arabian Standard Time	(UTC+04:00) Abu Dhabi, Muscat
(UTC+03:30) Tehran;Iran Standard Time	(UTC+03:30) Tehran
(UTC+03:00) Nairobi;E. Africa Standard Time	(UTC+03:00) Nairobi
(UTC+03:00) Moscow, St. Petersburg, Volgograd;Russian Standard Time	(UTC+03:00) Moscow, St. Petersburg, Volgograd
(UTC+03:00) Minsk;Belarus Standard Time	(UTC+03:00) Minsk
(UTC+03:00) Kuwait, Riyadh;Arab Standard Time	(UTC+03:00) Kuwait, Riyadh
(UTC+03:00) Baghdad;Arabic Standard Time	(UTC+03:00) Baghdad

Supported values of DateTime.TimeZone element

DateTime.TimeZone	Description (analyzer user interface value)
(UTC+02:00) Tripoli;Libya Standard Time	(UTC+02:00) Tripoli
(UTC+02:00) Kaliningrad;Kaliningrad Standard Time	(UTC+02:00) Kaliningrad
(UTC+02:00) Jerusalem;Israel Standard Time	(UTC+02:00) Jerusalem
(UTC+02:00) Istanbul;Turkey Standard Time	(UTC+02:00) Istanbul
(UTC+02:00) Helsinki, Kyiv, Riga, Sofia, Tallinn, Vilnius;FLE Standard Time	(UTC+02:00) Helsinki, Kyiv, Riga, Sofia, Tallinn, Vilnius
(UTC+02:00) Harare, Pretoria;South Africa Standard Time	(UTC+02:00) Harare, Pretoria
(UTC+02:00) E. Europe;E. Europe Standard Time	(UTC+02:00) E. Europe
(UTC+02:00) Damascus;Syria Standard Time	(UTC+02:00) Damascus
(UTC+02:00) Cairo;Egypt Standard Time	(UTC+02:00) Cairo
(UTC+02:00) Beirut;Middle East Standard Time	(UTC+02:00) Beirut
(UTC+02:00) Athens, Bucharest;GTB Standard Time	(UTC+02:00) Athens, Bucharest
(UTC+02:00) Amman;Jordan Standard Time	(UTC+02:00) Amman
(UTC+01:00) Windhoek;Namibia Standard Time	(UTC+01:00) Windhoek
(UTC+01:00) West Central Africa;W. Central Africa Standard Time	(UTC+01:00) West Central Africa
(UTC+01:00) Sarajevo, Skopje, Warsaw, Zagreb;Central European Standard Time	(UTC+01:00) Sarajevo, Skopje, Warsaw, Zagreb
(UTC+01:00) Brussels, Copenhagen, Madrid, Paris;Romance Standard Time	(UTC+01:00) Brussels, Copenhagen, Madrid, Paris
(UTC+01:00) Belgrade, Bratislava, Budapest, Ljubljana, Prague;Central Europe Standard Time	(UTC+01:00) Belgrade, Bratislava, Budapest, Ljubljana, Prague
(UTC+01:00) Amsterdam, Berlin, Bern, Rome, Stockholm, Vienna;W. Europe Standard Time	(UTC+01:00) Amsterdam, Berlin, Bern, Rome, Stockholm, Vienna
(UTC) Monrovia, Reykjavik;Greenwich Standard Time	(UTC) Monrovia, Reykjavik
(UTC) Dublin, Edinburgh, Lisbon, London;GMT Standard Time	(UTC) Dublin, Edinburgh, Lisbon, London
(UTC) Coordinated Universal Time;UTC	(UTC) Coordinated Universal Time
(UTC) Casablanca;Morocco Standard Time	(UTC) Casablanca
(UTC-01:00) Azores;Azores Standard Time	(UTC-01:00) Azores
(UTC-01:00) Cabo Verde Is.;Cape Verde Standard Time	(UTC-01:00) Cabo Verde Is.
(UTC-02:00) Coordinated Universal Time -02;UTC-2	(UTC-02:00) Coordinated Universal Time -02
(UTC-03:00) Brasilia;E. South America Standard Time	(UTC-03:00) Brasilia
(UTC-03:00) Buenos Aires;Argentina Standard Time	(UTC-03:00) Buenos Aires
(UTC-03:00) Cayenne, Fortaleza;SA Eastern Standard Time	(UTC-03:00) Cayenne, Fortaleza
(UTC-03:00) Greenland;Greenland Standard Time	(UTC-03:00) Greenland
(UTC-03:00) Montevideo;Montevideo Standard Time	(UTC-03:00) Montevideo
(UTC-03:00) Salvador;Bahia Standard Time	(UTC-03:00) Salvador
(UTC-03:30) Newfoundland;Newfoundland Standard Time	(UTC-03:30) Newfoundland
(UTC-04:00) Asuncion;Paraguay Standard Time	(UTC-04:00) Asuncion
(UTC-04:00) Atlantic Time (Canada);Atlantic Standard Time	(UTC-04:00) Atlantic Time (Canada)
(UTC-04:00) Cuiaba;Central Brazilian Standard Time	(UTC-04:00) Cuiaba
(UTC-04:00) Georgetown, La Paz, Manaus, San Juan;SA Western Standard Time	(UTC-04:00) Georgetown, La Paz, Manaus, San Juan
(UTC-04:00) Santiago;Pacific SA Standard Time	(UTC-04:00) Santiago
(UTC-04:00) Caracas;Venezuela Standard Time	(UTC-04:30) Caracas
(UTC-05:00) Bogota, Lima, Quito, Rio Branco;SA Pacific Standard Time	(UTC-05:00) Bogota, Lima, Quito, Rio Branco
(UTC-05:00) Chetumal;Eastern Standard Time (Mexico)	(UTC-05:00) Chetumal

Supported values of DateTime.TimeZone element

DateTime.TimeZone	Description (analyzer user interface value)
(UTC-05:00) Eastern Time (US & Canada);Eastern Standard Time	(UTC-05:00) Eastern Time (US & Canada)
(UTC-05:00) Haiti;Haiti Standard Time	(UTC-05:00) Haiti
(UTC-05:00) Indiana (East);US Eastern Standard Time	(UTC-05:00) Indiana (East)
(UTC-06:00) Central America;Central America Standard Time	(UTC-06:00) Central America
(UTC-06:00) Central Time (US & Canada);Central Standard Time	(UTC-06:00) Central Time (US & Canada
(UTC-06:00) Easter Island;Easter Island Standard Time	(UTC-06:00) Easter Island
(UTC-06:00) Guadalajara, Mexico City, Monterrey;Central Standard Time (Mexico)	(UTC-06:00) Guadalajara, Mexico City, Monterrey
(UTC-06:00) Saskatchewan;Canada Central Standard Time	(UTC-06:00) Saskatchewan
(UTC-07:00) Arizona;US Mountain Standard Time	(UTC-07:00) Arizona
(UTC-07:00) Chihuahua, La Paz, Mazatlan;Mountain Standard Time (Mexico)	(UTC-07:00) Chihuahua, La Paz, Mazatlan
(UTC-07:00) Mountain Time (US & Canada);Mountain Standard Time	(UTC-07:00) Mountain Time (US & Canada)
(UTC-08:00) Baja California;Pacific Standard Time (Mexico)	(UTC-08:00) Baja California
(UTC-08:00) Pacific Time (US & Canada);Pacific Standard Time	(UTC-08:00) Pacific Time (US & Canada)
(UTC-09:00) Alaska;Alaskan Standard Time	(UTC-09:00) Alaska
(UTC-10:00) Hawaii;Hawaiian Standard Time	(UTC-10:00) Hawaii
(UTC-11:00) Coordinated Universal Time -11;UTC-11	(UTC-11:00) Coordinated Universal Time -11
(UTC-12:00) International Date Line West;Dateline Standard Time	(UTC-12:00) International Date Line West

Supported values of DateTime.TimeZone element

Related topics

- Device Configuration (53)
- Message structure: Device configuration message (DTV.ROCHE.LIAT.CFG) (116)
- Examples: Device configuration directive (143)

Lot object (LOT)

The Lot object is a component of the custom Lot topic (ROCHE.LIAT.LOTS.R01 and ROCHE.LIAT.LOTS.R02).

Element	DT	Attribute	Comment
lot_id	ST	V	Identifies uniquely the validated assay tube lot.<br>Before applying a lot received by the connected DMS, the analyzer validates syntactically the lot_id. It checks that the value is composed by:<br>• Assay name<br>• Barcode lot number (4 digit code) or manufacturer lot number (6 digit code)<br>About lot number (104)<br>• Minimum compatible assay version<br>Examples:<br>• `SASA^79EX^1.26`<br>• `FABA^90101R^1.33`<br>Unknown lot ids are ignored.
lot_insert_id	ST	V	The barcode used to start the lot validation.
parameters	ST	V	Lot specific parameters. These are Roche-internal parameters used by the run assay script.<br>The maximum length of a parameter is 11 characters.
assay	ST	V	Name of the assay to which the validated lot belongs.
expiration_date	TS		Expiration date of the assay tube lot.<br>Time and time zone information of the datetime field are set tozero, and are ignored. Only the date is used.<br>Ex: `V= "2018-10-30T00:00:00+00:00"`<br>This field reflects the expiration date of the lot.
lot_number	ST	V	Barcode lot number (4 digit code) or manufacturer lot number (6 digit code)<br>About lot number (104)
minimum_compatible_version	ST	V	The minimum compatible assay version of a lot received from a DMS is compared to the one specified for the installed assay in order to decide whether a received lot can be used with the locally installed assay version.
validation_dttm	TS	V	Date and time the lot was validated.<br>This field reflects the precise date/time/timezone when the lot was validated on an analyzer.
data	ST	V	Contains the encoded digital signature of the validated lot.

Lot object (LOT)

Related topics

- Lots (51)
- Message structure: Lot full list messages (ROCHE.LIAT.LOTS.R01) (118)
- Message structure: Lot partial list messages (ROCHE.LIAT.LOTS.R02) (119)
- Example: Lot topic (130)

About lot number

The lot number consists of the encoded manufacture date of the lot and a 1-letter identifier (lot code).

Lot manufacture date encoding

The lot manufacturing date can be encoded by a 6-digit code (manufacturer lot number), or by 4-digit code (barcode lot number).

On the user interface, the 6-digit code is displayed. For internal storage in the database, and in POCT1-A messages for the exchange of lot information with a connected DMS, the 4-digit code is used for lots created with a software version lower than 3.3.0. For new lots created on the analyzer with software version 3.3.0, the 6-digit code is used.

With software version 3.3.0, lots with 4-digit code are usable and accepted by the analyzer.

If a customer uses analyzers with different software versions, you should validate lots on an analyzer using a software version lower than 3.3.0.

Manufacture lot number (6-digit code)	Barcode lot number (4-digit code)
Format: `YMMDDI`<br>• `Y` = last digit of manufacture year<br>• `MM` = month of manufacture date<br>• `DD` = day of manufacture date<br>• `I` = lot identifier: 1-letter identifier (A to Z)	Format: `YMDI`<br>• `Y` = 1-digit abbreviation for manufacture year<br>Abbreviations Y (104)<br>• `M` = 1-digit abbreviation for month of manufacture date<br>Abbreviations for M (105)<br>• `D` = 1-digit abbreviation for day of manufacture date<br>Abbreviations for D (106)<br>• `I` = lot identifier: 1-letter identifier (A to Z)

Manufacture lot number and barcode lot number

Examples

The following tables provides 2 examples of the 6-digit and 4-digit encoding of the lot number.

Lot number (uncoded)	6-digit code	4-digit code
06-MAY-2017, lot A	`70506A`	`756A`
02-JAN-2018, lot Z	`80102Z`	`A12Z`

Encoding of lot manufacture date

Encoding tables

Y (1st digit)	Manufacture year
8	2008
9	2009
0	2010
1	2011
2	2012
3	2013

Abbreviations Y

Y (1st digit)	Manufacture year
4	2014
5	2015
6	2016
7	2017
A	2018
B	2019
C	2020
D	2021
E	2022
F	2023
G	2024
H	2025
I	2026
J	2027
K	2028
L	2029
M	2030
N	2031
O	2032
P	2033
Q	2034
R	2035
S	2036
T	2037
U	2038
V	2039
W	2040
X	2041
Y	2042
Z	2043

Abbreviations Y

M (2nd digit)	Manufacture month
1	January
2	February
3	March
4	April
5	May
6	June
7	July
8	August
9	September
0	October
A	November
B	December

Abbreviations for M

D (3rd digit)	Manufacture day
1	1
2	2
3	3
4	4
5	5
6	6
7	7
8	8
9	9
0	10
A	11
B	12
C	13
D	14
E	15
F	16
G	17
H	18
I	19
J	20
K	21
L	22
M	23
N	24
O	25
P	26
Q	27
R	28
S	29
T	30
U	31

Abbreviations for D

Message structure

The **cobas**® Liat® System supports several standard POCT1-A message types, as well as some custom extensions to the POCT1-A protocol.

In this chapter

4

Supported POCT1-A message structure 108
Acknowledgment message (ACK.R01) 108
Device status message (DST.R01) 108
End of topic message (EOT.R01). 109
Event message (EVS.R01) 109
Escape message (ESC.R01). 110
Hello message (HEL.R01) 110
Keep alive message (KPA.R01). 111
Observation messages (OBS) 112
Operator messages (OPL) 114
Request message (REQ.R01) 115
Termination message (END.R01) 115

Custom **cobas**® Liat® messages 116
Device configuration message (DTV.ROCHE.LIAT.CFG) 116
Lot full list messages (ROCHE.LIAT.LOTS.R01) . . . 118
Lot partial list messages (ROCHE.LIAT.LOTS.R02). 119
Patient verification request message (ROCHE.LIAT.PVI.R01) 119
Patient verification response message (ROCHE.LIAT.PVR.R01). 120

Supported POCT1-A message structure

See POCT1-A2 "Point-of-Care Connectivity - Approved Standard Second Edition" standardized under CLSI Vol. 26 No. 28, appendix B, chap. 6.1 for the notation conventions that apply to the message structure figures.

To get an overview of all POCT1-A standard messages and their elements, see POCT1-A2 "Point-of-Care Connectivity - Approved Standard Second Edition" standardized under CLSI Vol. 26 No. 28, appendix B, chap. 6.

Acknowledgment message (ACK.R01)

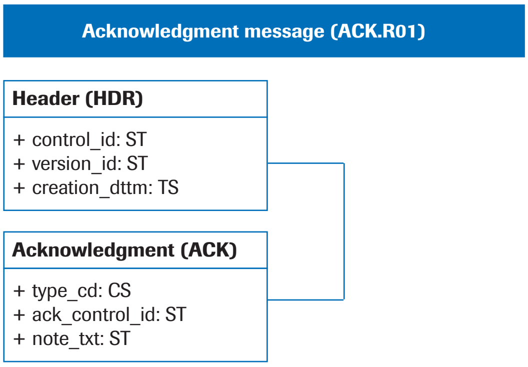

In the "ACK.ack_control_id" attributed, the device always sends the last known/valid "HDR.control_id".

Related topics

- Conversations and topics (38)
- Acknowledgment object (ACK) (70)
- Header object (HDR) (75)

Device status message (DST.R01)

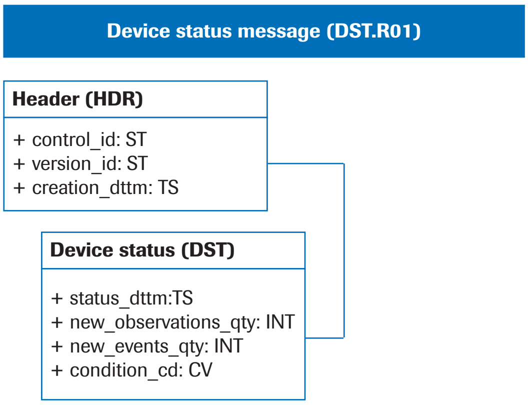

Related topics

- Conversations and topics (38)
- Acknowledgment object (ACK) (70)
- Device status object (DST) (72)
- Examples: Communication start up topics (127)

End of topic message (EOT.R01)

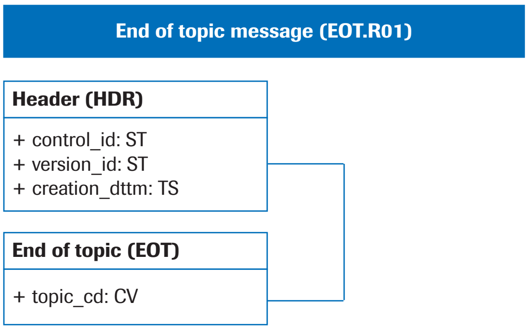

The End of topic message is used by the analyzer to indicate the end of a sequence of related messages to the DMS. This ability is needed to transfer large amounts of data on a topic such that the data is distributed into smaller, related messages when sending to the DMS.

Related topics

- Conversations and topics (38)
- End of topic object (EOT) (73)
- Header object (HDR) (75)

Event message (EVS.R01)

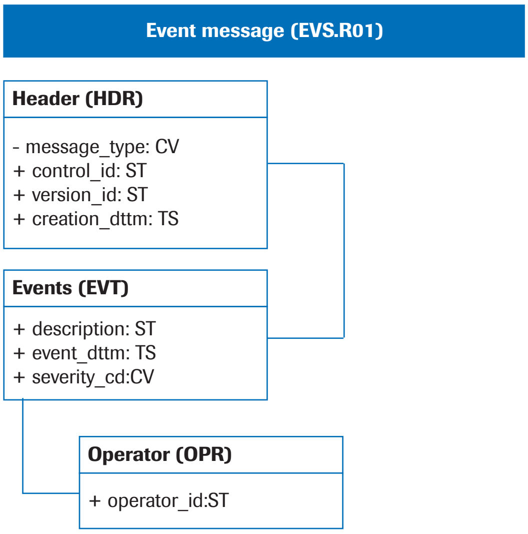

Related topics

- Events (55)
- Event object (EVT) (74)
- Header object (HDR) (75)
- Operator object (OPR) (78)

Escape message (ESC.R01)

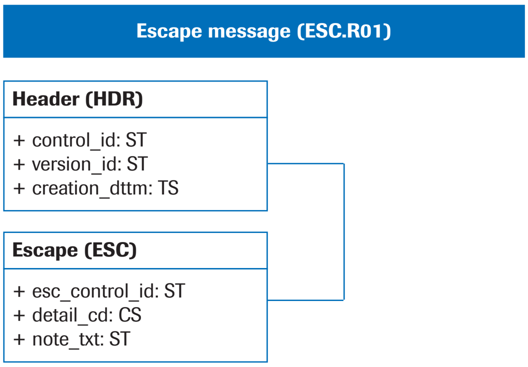

The analyzer or DMS uses the Escape message to interrupt the current conversation topic.

A topic of a conversation may be ended prematurely by the device or the DMS by used of the Escape message. When the Escape message is sent, the receiver must terminate the topic activity, and move on to the next topic of the conversation.

Related topics

- Header object (HDR) (75)
- Escape object (ESC) (74)

Hello message (HEL.R01)

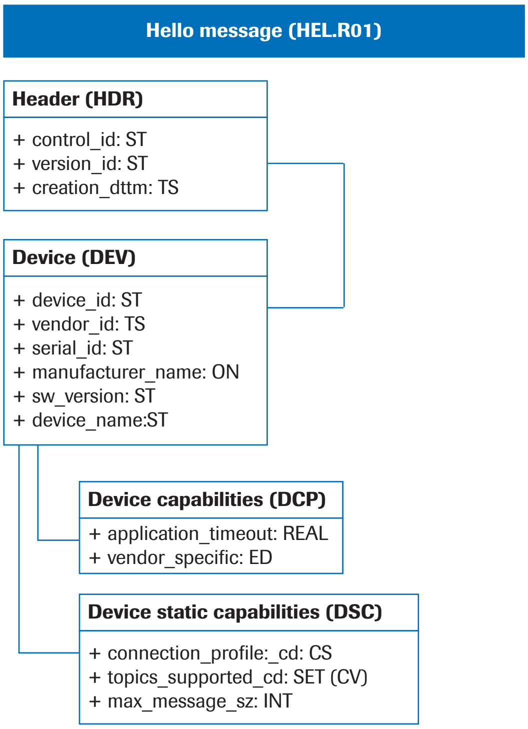

The Hello message is sent by the analyzer to indicate to the DMS that it wants to start a conversation.

Related topics

- Initialization flow (27)
- Communication initialization (25)
- Header object (HDR) (75)
- Device object (DEV) (80)
- Device capabilities object (DCP) (81)
- Device static capabilities object (DSC) (82)
- Examples: Communication start up topics (127)

Keep alive message (KPA.R01)

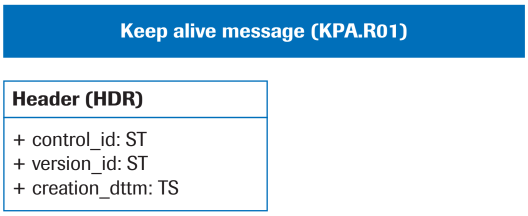

Related topics

- Keep alive (33)
- Header object (HDR) (75)
- Example: Keep alive message (147)

Observation messages (OBS)

The need for uploading of unsent results is indicated by the Device status message at the beginning of every communication (DST.new_observations_qty).

Observation messages are sent one at a time.

Patient-related Observation Message (OBS.R01)

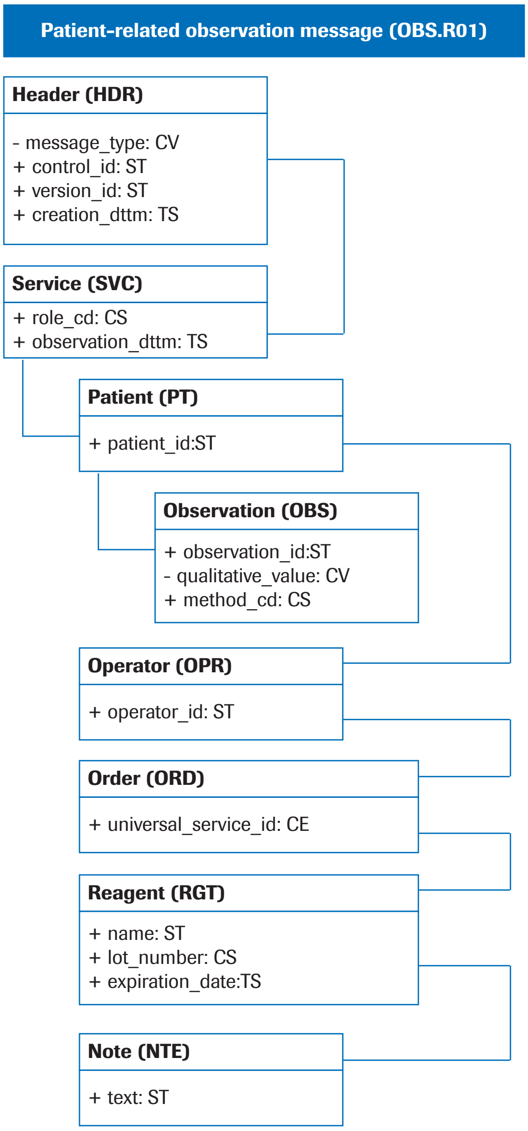

The Observation message OBS.R01 is adapted to the analyzer’s requirements for patient-related observations.

Observations (results) (41)

Observation object (OBS) (75)

Header object (HDR) (75)

Observation object (OBS) (75)

Service Object (SVC) (79)

Order object (ORD) (77)

Patient object (PT) (85)

Operator object (OPR) (78)

Reagent object (RGT) (79)

Note object (NTE) (83)

Example: Observation topic (134)

Non-patient-related observation message (OBS.R02)

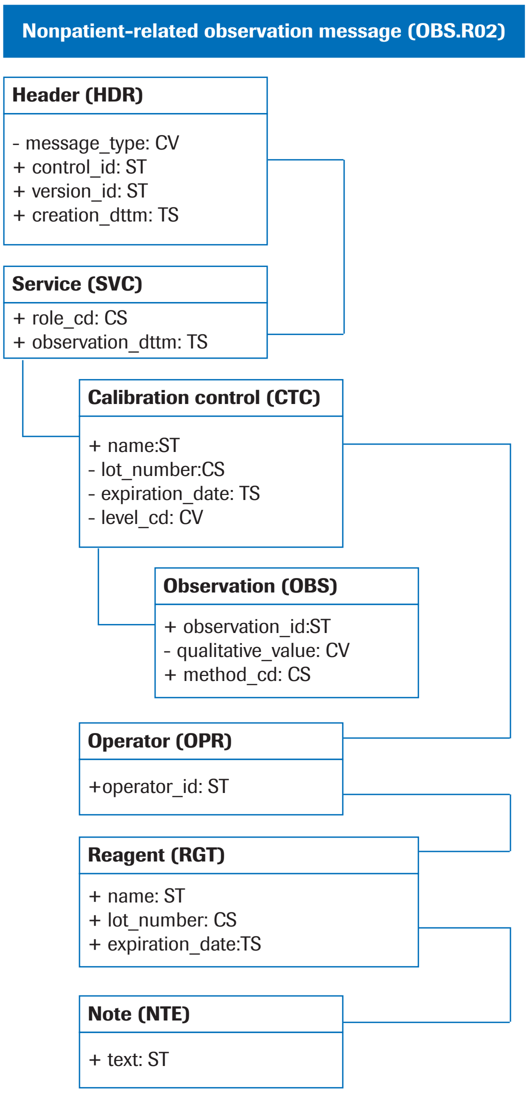

The Observation message OBS.R02 is used to transmit the analyzer's results for liquid quality-control tests.

Observations (results) (41)

Control / Calibration object (CTC) (71)

Observation object (OBS) (75)

Header object (HDR) (75)

Observation object (OBS) (75)

Service Object (SVC) (79)

Order object (ORD) (77)

Operator object (OPR) (78)

Reagent object (RGT) (79)

Note object (NTE) (83)

Example: Observation topic (134)

Operator messages (OPL)

Operator full list message (OPL.R01)

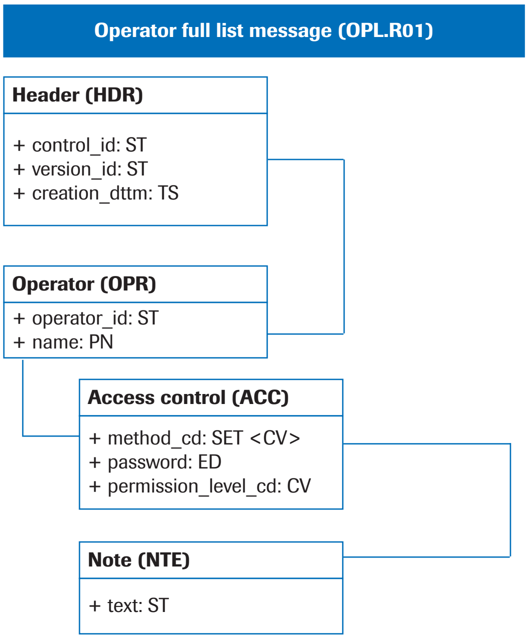

Operator and lot lists (44)
About operators (48)
Access control object (ACC) (69)
Header object (HDR) (75)
Operator object (OPR) (78)
Note object (NTE) (83)
Example: Operators topic (140)

Operator partial list message

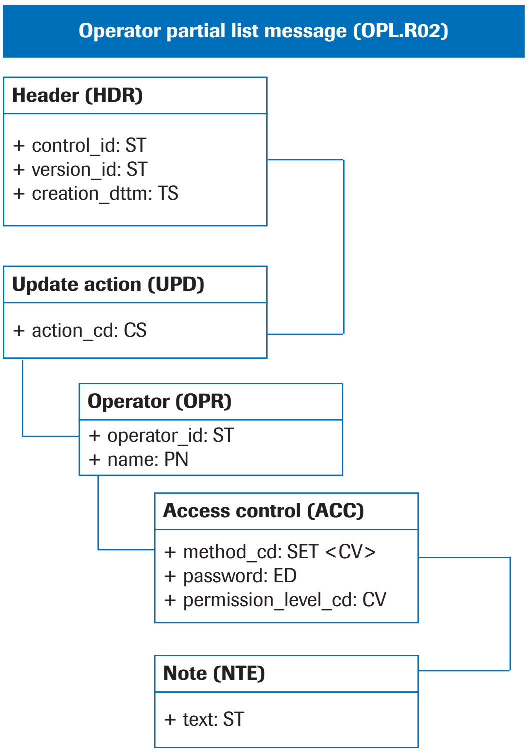

Operator and lot lists (44)
About operators (48)
Access control object (ACC) (69)
Update Action object (UPD) (84)
Header object (HDR) (75)
Operator object (OPR) (78)
Note object (NTE) (83)
Example: Operators topic (140)

Request message (REQ.R01)

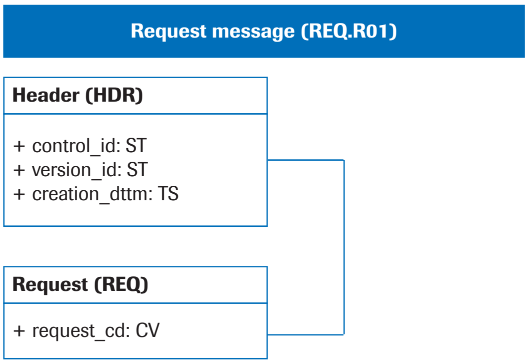

The Request message is used by the DMS to prompt the analyzer to begin transferring data. The type of data requested depends on the Request message.

Related topics

- Workflows (37)
- Header object (HDR) (75)
- Request object (REQ) (80)

Termination message (END.R01)

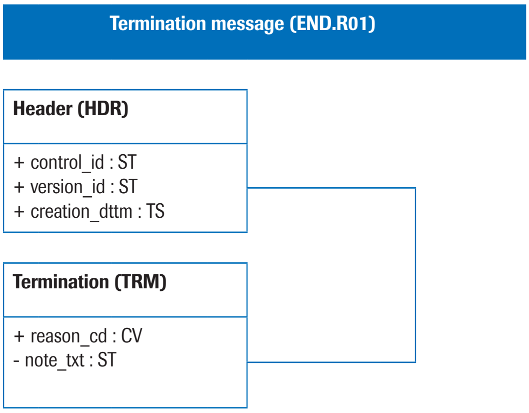

Each conversation is ended by a termination message. Normally the DMS will send the termination but on rare occasions it is acceptable for the analyzer to terminate the conversation.

Related topics

- Communication termination (28)
- Header object (HDR) (75)
- Termination object (TRM) (83)

Custom cobas® Liat® messages

The **cobas**® Liat® System supports several messages that provide custom extensions to the POCT1-A protocol.

Device configuration message (DTV.ROCHE.LIAT.CFG)

DTV.ROCHE.LIAT.CFG message


```
<DTV.ROCHE.LIAT.CFG>
    <HDR>
       <HDR.message_type V="DTV.ROCHE.LIAT.CFG" SN="ROCHE" SV="1.0"/>
       <HDR.control_id V="3"/>
       <HDR.version_id V="POCT1"/>
       <HDR.creation_dttm V="2019-04-26T16:10:00+00:00"/>
    </HDR>
    <DTV>
        <DTV.command_cd V="SET_CONFIG" SN="ROCHE" SV="1.0"/>
    </DTV>
    <GEN_CFG>
        <GEN_CFG.DateTime.sntp V="false"/>
        <GEN_CFG.DateTime.Server V=""/>
        <GEN_CFG.DateTime.TimeZone V="Central European Standard Time"/>
        <GEN_CFG.DateTime.TimeFormat V="12"/>
        <GEN_CFG.DateTime.DateFormat V="yyyy-mm-dd"/>
        <GEN_CFG.Display.brightness V="7"/>
        <GEN_CFG.EnableTilt.EnableCheckTilt V="true"/>
        <GEN_CFG.Sound.sSoundInitialization V="Beep2"/>
        <GEN_CFG.Sound.sSoundBarcodeScan V="Beep3"/>
        <GEN_CFG.Sound.sSoundTubeInsert V="Beep4"/>
        <GEN_CFG.Sound.sSoundAssayFinish V="BUZZER"/>
        <GEN_CFG.Sound.sSoundTouchScreen V="Soft"/>
        <GEN_CFG.Sound.sSoundKeyClicks V="Soft"/>
        <GEN_CFG.Sound.SoundVolume V="1"/>
        <GEN_CFG.TubeInsertTime.iTubeInsertTime V="15"/>
        <GEN_CFG.AutoLock.autolocktime V="7"/>
        <GEN_CFG.Authentication.authenticationType V="User ID &amp; Password"/>
        <GEN_CFG.Languages.Language V="en-US"/>
        <GEN_CFG.AutoReboot.Time V="05:30"/>
        <GEN_CFG.Connectivity.Timeout V="30"/>
        <GEN_CFG.Connectivity.DMLAutoSend V="false"/>
        <GEN_CFG.Connectivity.DataSynchronizationUsers V="true"/>
        <GEN_CFG.Connectivity.DataSynchronizationAssayLots V="true"/>
        <GEN_CFG.Connectivity.DataSynchronizationLogEvents V="true"/>
        <GEN_CFG.Connectivity.DataSynchronizationInformation V="true"/>
        <GEN_CFG.Connectivity.DataSynchronizationWarningErrors V="true"/>
        <GEN_CFG.Connectivity.ConnectionInterval V="5"/>
        <GEN_CFG.BarcodeITF.Enabled V="false"/>
        <GEN_CFG.BarcodeITF.Checksum V="true"/>
        <GEN_CFG.BarcodeITF.FixLength V="One discrete length"/>
        <GEN_CFG.BarcodeITF.BarcodeLength V="10"/>
        <GEN_CFG.BarcodeCodabar.Enabled V="true"/>
        <GEN_CFG.BarcodeCodabar.TransmitStartStopChar V="false"/>
        <GEN_CFG.BarcodeCode39.Enabled V="true"/>
        <GEN_CFG.BarcodeCode39.Checksum V="true"/>
```

```
<GEN_CFG.BarcodeCode93.Enabled V="true"/>
<GEN_CFG.BarcodeEAN8.Enabled V="true"/>
<GEN_CFG.BarcodeEAN13.Enabled V="true"/>
<GEN_CFG.BarcodeGS1Databar14.Enabled V="true"/>
<GEN_CFG.PV.Verification V="prior run"/>
<GEN_CFG.PV.VerificationType V="o"/>
<GEN_CFG.PV.PatientMismatch V="run allowed"/>
<GEN_CFG.PV.DisplayedData V="verbose"/>
<GEN_CFG.PV.ManualConfirmation V="not required"/>
<GEN_CFG.PRContent.CommunicationLog V="included"/>
<GEN_CFG.PRContent.Runlog V="included"/>
<GEN_CFG.PRContent.DataRange V="last30days"/>
<GEN_CFG.PRContent.Sampleresults V="included"/>
<GEN_CFG.PRContent.SampleID V="excluded"/>
<GEN_CFG.PRContent.Users V="excluded"/>
<GEN_CFG.PRSchedule.Creation V="on demand"/>
<GEN_CFG.PRSchedule.DayOfMonth V="firstdayofmonth"/>
<GEN_CFG.PRSchedule.DayOfWeek V="sunday"/>
<GEN_CFG.PRSchedule.Destination V="remoteservicesystem"/>
<GEN_CFG.PRSchedule.Time V="16"/>
<GEN_CFG.PRSchedule.Frequency V="monthly"/>
<GEN_CFG.Printers.InkLaserName V="HP Officejet Pro 8100"/>
<GEN_CFG.Printers.InkLaserDescription V="Officejet Pro 8100 [126E24]"/>
<GEN_CFG.Printers.InkLaserLocation V=""/>
<GEN_CFG.Printers.InkLaserColorMode V="greyscale"/>
<GEN_CFG.Printers.InkLaserConnection V="dnssd://Officejet%20Pro%208100%20%5B126E24%5D._pdlda
 tastream._tcp.local/?uuid=1c852a4d-b800-1f08-abcd-a02bb8126e24"/>
<GEN_CFG.Printers.InkLaserUser V=""/>
<GEN_CFG.Printers.InkLaserPassword V=""/>
<GEN_CFG.Printers.ThermalName V="Brother QL-820NWB"/>
<GEN_CFG.Printers.ThermalDescription V="Brother QL-820NWB"/>
<GEN_CFG.Printers.ThermalLocation V=""/>
<GEN_CFG.Printers.ThermalColorMode V="greyscale"/>
<GEN_CFG.Printers.ThermalConnection V="dnssd://Brother%20QL 820NWB._ipp._tcp.local/?uuid=e32
 48000-80ce-11db-8000-0080775abcab"/>
<GEN_CFG.Printers.ThermalUser V=""/>
<GEN_CFG.Printers.ThermalPassword V=""/>
<GEN_CFG.Printers.ReportPrinting.SelectedPrinter V="inklaser"/>
<GEN_CFG.Printers.ResultPrinting.Autoprinting V="false"/>
<GEN_CFG.Printers.ResultPrinting.SelectedPrinter V="thermal"/>
<GEN_CFG.Printers.ResultPrinting.Manualprinting V="default"/>
<GEN_CFG.SLNetworkShare1.Name V="Network Share 1"/>
<GEN_CFG.SLNetworkShare1.ServerName V="10.138.206.208"/>
<GEN_CFG.SLNetworkShare1.FolderPath V="SharedFolder_1"/>
<GEN_CFG.SLNetworkShare1.UserName V="UserRW"/>
<GEN_CFG.SLNetworkShare1.Password V="617617"/>
<GEN_CFG.SLNetworkShare2.Name V="Network Share 2"/>
<GEN_CFG.SLNetworkShare2.ServerName V="10.138.206.208"/>
<GEN_CFG.SLNetworkShare2.FolderPath V="SharedFolder_2"/>
<GEN_CFG.SLNetworkShare2.UserName V="UserRW"/>
<GEN_CFG.SLNetworkShare2.Password V="617617"/>
<GEN_CFG.SLNetworkShare3.Name V="Network Share 3"/>
<GEN_CFG.SLNetworkShare3.ServerName V="10.138.206.208"/>
<GEN_CFG.SLNetworkShare3.FolderPath V="SharedFolder_3"/>
<GEN_CFG.SLNetworkShare3.UserName V="UserRW"/>
<GEN_CFG.SLNetworkShare3.Password V="617617"/>
<GEN_CFG.SLFTPShare1.Name V="FTP share 1"/>
<GEN_CFG.SLFTPShare1.Type V="FTP"/>
<GEN_CFG.SLFTPShare1.IP V="192.168.222.44"/>
```

```
        <GEN_CFG.SLFTPShare1.Port V="2554"/>
        <GEN_CFG.SLFTPShare1.FolderPath V="c:\testfolder"/>
        <GEN_CFG.SLFTPShare1.UserName V="SI"/>
        <GEN_CFG.SLFTPShare1.Password V="617617"/>
        <GEN_CFG.SLFTPShare2.Name V="FTP share 2"/>
        <GEN_CFG.SLFTPShare2.Type V="FTP"/>
        <GEN_CFG.SLFTPShare2.IP V="192.168.222.44"/>
        <GEN_CFG.SLFTPShare2.Port V="2554"/>
        <GEN_CFG.SLFTPShare2.FolderPath V="c:\testfolder"/>
        <GEN_CFG.SLFTPShare2.UserName V="SI"/>
        <GEN_CFG.SLFTPShare2.Password V="617617"/>
        <GEN_CFG.SLFTPShare3.Name V="FTP share 3"/>
        <GEN_CFG.SLFTPShare3.Type V="FTP"/>
        <GEN_CFG.SLFTPShare3.IP V="192.168.222.44"/>
        <GEN_CFG.SLFTPShare3.Port V="2554"/>
        <GEN_CFG.SLFTPShare3.FolderPath V="c:\testfolder"/>
        <GEN_CFG.SLFTPShare3.UserName V="SI"/>
        <GEN_CFG.SLFTPShare3.Password V="617617"/>
        <GEN_CFG.RemoteService.HTTPproxy V="false"/>
        <GEN_CFG.RemoteService.Server V=""/>
        <GEN_CFG.RemoteService.Port V=""/>
        <GEN_CFG.RemoteService.Authentication V="false"/>
        <GEN_CFG.RemoteService.UserName V=""/>
        <GEN_CFG.RemoteService.Password V=""/>
    </GEN_CFG>
</DTV.ROCHE.LIAT.CFG>
```

Related topics

- Device Configuration (53)
- Header object (HDR) (75)
- Generic configuration object (GEN_CFG) (88)
- Examples: Device configuration directive (143)

Lot full list messages (ROCHE.LIAT.LOTS.R01)

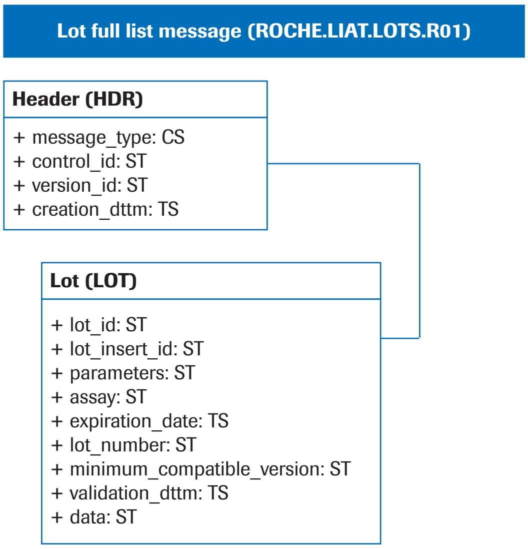

Related topics

- Operator and lot lists (44)
- Header object (HDR) (75)
- Lot object (LOT) (103)
- Examples: Lot topic (130)

Lot partial list messages (ROCHE.LIAT.LOTS.R02)

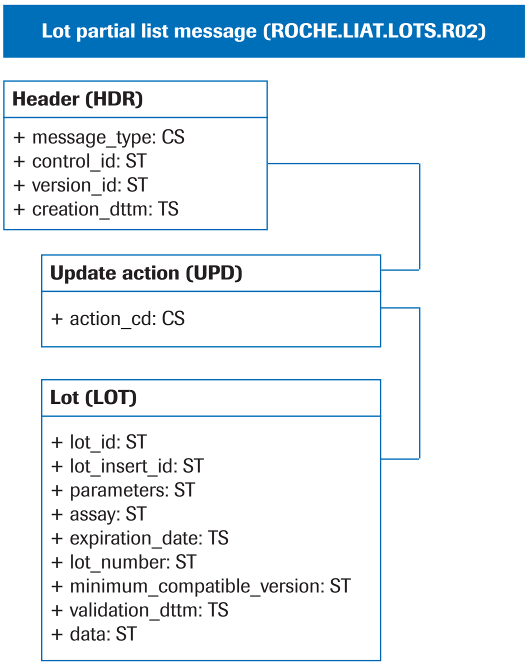

Related topics

- Operator and lot lists (44)
- Header object (HDR) (75)
- Update Action object (UPD) (84)
- Lot object (LOT) (103)
- Examples: Lot topic (130)

Patient verification request message (ROCHE.LIAT.PVI.R01)

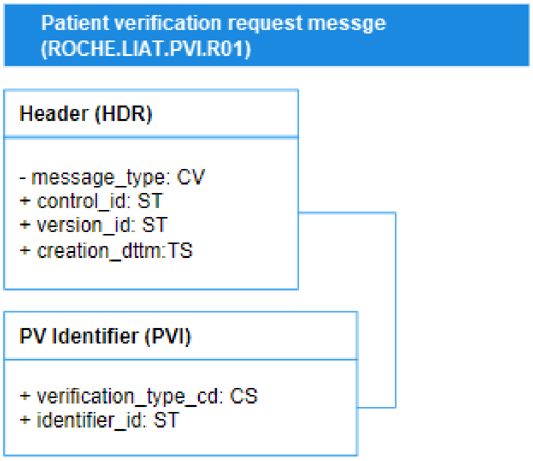


```
<ROCHE.LIAT.PVI.R01>
  <HDR>
    <HDR.control_id V="875"/>
    <HDR.version_id V="POCT1"/>
    <HDR.creation_dttm V="2019-08-13T15:15:08+02:00"/>
  </HDR>
  <PVI>
    <PVI.verification_type_cd V="O"/>
    <PVI.identifier_id V="1234"/>
  </PVI>
```

```
</ROCHE.LIAT.PVI.R01>
```

Related topics

- Patient verification (63)
- Header object (HDR) (75)
- Patient verification identifier object (PVI) (87)
- Example: Communication scenario 9 - Patient verification passed - run performed (197)
- Example: Communication scenario 10 - Patient verification failed - run prevented (205)

Patient verification response message (ROCHE.LIAT.PVR.R01)

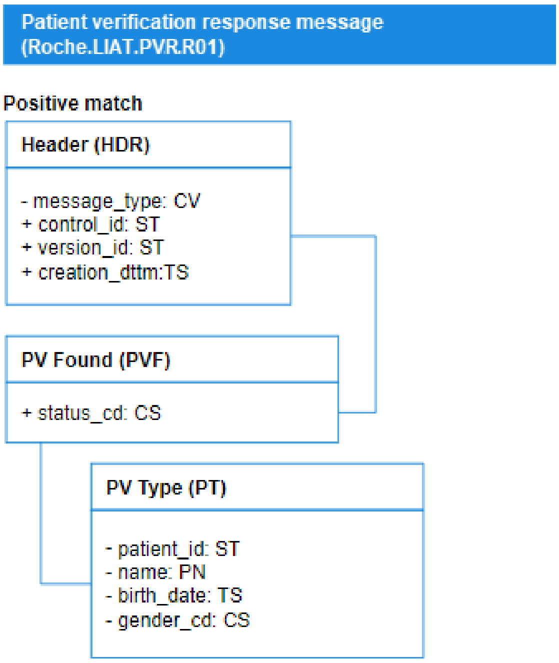

DMS to analyzer (positive answer - matching record found by DMS)

```
<ROCHE.LIAT.PVR.R01>
  <HDR>
    <HDR.control_id V="877"/>
    <HDR.version_id V="POCT1"/>
    <HDR.creation_dttm V="2019-08-13T15:14:54+02:00"/>
  </HDR>
  <PVF.status_cd V="T"/>
  <PT>
    <PT.patient_id V="A-12345678"/>
    <PT.name V="Diego"/>
    <PT.birth_date V="1990-01-01"/>
    <PT.gender_cd V="M"/>
  </PT>
</ROCHE.LIAT.PVR.R01>
```

DMS to analyzer (negative answer - no matching record found by DMS)

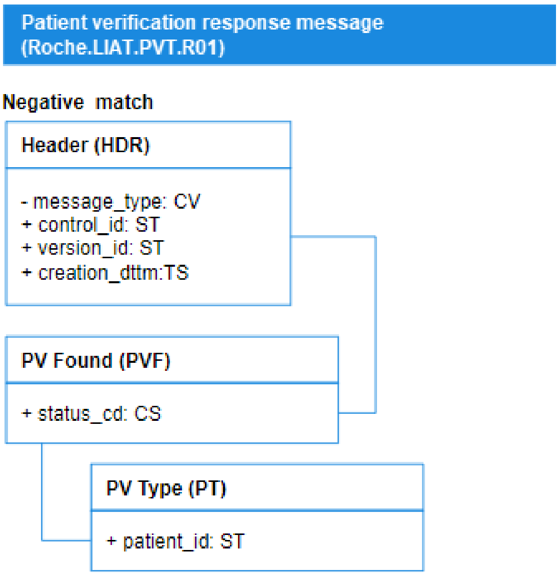


```
<ROCHE.LIAT.PVR.R01>
  <HDR>
    <HDR.control_id V="6"/>
    <HDR.version_id V="POCT1"/>
    <HDR.creation_dttm V="2019-08-13T15:25:38+02:00"/>
  </HDR>
  <PVF.status_cd V="F"/>
  <PT/>
</ROCHE.LIAT.PVR.R01>
```

Related topics

- Patient verification (63)
- Header object (HDR) (75)
- Patient verification found object (PVF) (87)
- Patient object (PT) (85)
- Example: Communication scenario 9 - Patient verification passed - run performed (197)
- Example: Communication scenario 10 - Patient verification failed - run prevented (205)

Communication examples

5 Communication examples 125
6 Example message logs 149
7 Additional examples 163

Communication examples

This section has example messages and scenarios, which show communication between the analyzer and the DMS. All values and data are examples only.
Messages are highlighted on each communication example:
Green for messages sent by the analyzer.
Blue for messages sent by the DMS.

In this chapter 5

Communication start up topics. 127
Lot topic. 130
Observation topic 134
Operators topic 140
Device configuration directive 143
Communication ending 146
Keep alive message 147

Communication start up topics

The protocol strictly defines the sequence of topics and messages required to start a conversation. A device initiates this start up sequence by sending a Hello message to a DMS.

Hello topic

- Hello message sent from device to the DMS.
- Response message is an acknowledgment sent from DMS to the device.

```
<HEL.R01>
    <HDR>
        <HDR.control_id V="987"/>
        <HDR.version_id V="POCT1"/>
        <HDR.creation_dttm V="2019-08-14T08:30:38+02:00"/>
    </HDR>
    <DEV>
        <DEV.device_id V="f8:dc:7a:06:27:0c"/>
        <DEV.vendor_id V="ROCHE"/>
        <DEV.serial_id V="M1-E-10063"/>
        <DEV.manufacturer_name V="Roche Molecular Diagnostics"/>
        <DEV.sw_version V="3.3.0.4027"/>
        <DEV.device_name V="cobasLiat"/>
        <DCP>
            <DCP.application_timeout V="120"/>
            <DCP.vendor_specific>ROCHE.LIAT.LOTS.R01;ROCHE.LIAT.LOTS.R02;DTV.ROCHE.LIAT.CFG
            </DCP.vendor_specific>
        </DCP>
        <DSC>
            <DSC.connection_profile_cd V="SA"/>
            <DSC.topics_supported_cd V="OP_LST"/>
            <DSC.topics_supported_cd V="OP_LST_I"/>
            <DSC.topics_supported_cd V="D_EV"/>
            <DSC.topics_supported_cd V="DTV"/>
            <DSC.max_message_sz V="614400"/>
       </DSC>
    </DEV>
</HEL.R01>
```

Hello Message

```
<ACK.R01>
    <HDR>
        <HDR.control_id V="2"/>
        <HDR.version_id V="POCT1"/>
        <HDR.creation_dttm V="2019-08-14T08:30:21+02:00"/>
    </HDR>
    <ACK>
        <ACK.type_cd V="AA"/>
        <ACK.ack_control_id V="987"/>
    </ACK>
```

```
</ACK.R01>
```

Positive ACK message

Communication initialization (25)
Initialization flow (27)
Conversations and topics (38)
Acknowledgment object (ACK) (70)
Header object (HDR) (75)
Device object (DEV) (80)
Device capabilities object (DCP) (81)
Device static capabilities object (DSC) (82)
Acknowledgment message (ACK.R01) (108)
Hello message (HEL.R01) (110)
Examples: Operators topic (140)

Device status topic

- Device Status message indicates that new observations and new events are available on the device.
- instrument state is "Partial Lock" which means one or more tests are locked on the device.

```
<DST.R01>
    <HDR>
        <HDR.control_id V="988"/>
        <HDR.version_id V="POCT1"/>
        <HDR.creation_dttm V="2019-08-14T08:30:38+02:00"/>
    </HDR>
    <DST>
        <DST.status_dttm V="2019-08-14T08:30:38+02:00"/>
        <DST.new_observations_qty V="0"/>
        <DST.new_events_qty V="25"/>
        <DST.condition_cd V="S"/>
    </DST>
</DST.R01>
```

Device Status Message

```
<ACK.R01>
    <HDR>
        <HDR.control_id V="3"/>
        <HDR.version_id V="POCT1"/>
        <HDR.creation_dttm V="2019-08-14T08:30:22+02:00"/>
    </HDR>
    <ACK>
        <ACK.type_cd V="AA"/>
        <ACK.ack_control_id V="988"/>
    </ACK>
</ACK.R01>
```

Positive ACK message

Communication initialization (25)
Initialization flow (27)
Conversations and topics (38)
Acknowledgment object (ACK) (70)
Device status object (DST) (72)
Header object (HDR) (75)
Acknowledgment message (ACK.R01) (108)
Device status message (DST.R01) (108)

Lot topic

New 4-digit lot with partial list


```
<ROCHE.LIAT.LOTS.R02>
    <HDR>
        <HDR.message_type V="ROCHE.LIAT.LOTS.R02" SN="ROCHE" SV="1.0"/>
        <HDR.control_id V="26"/>
        <HDR.version_id V="POCT1"/>
        <HDR.creation_dttm V="2019-08-14T12:46:55+02:00"/>
    </HDR>
    <UPD>
        <UPD.action_cd V="I"/>
        <LOT>
            <LOT.lot_id V="SASA^A56B^1.26"/>
            <LOT.lot_insert_id V="ISASA3412A56BAAAAAAAAAAAT"/>
            <LOT.parameters V="AAAAAAAAAAA"/>
            <LOT.assay V="SASA"/>
            <LOT.expiration_date V="2034-12-31"/>
            <LOT.lot_number V="A56B"/>
            <LOT.minimum_compatible_version V="1.26"/>
            <LOT.validation_dttm V="2018-07-01T00:00:00+00:00"/>
            <LOT.data V="ORO2TpIanrlO4iOB3SYH/jIrzI7XXaqNcB0xQjalVBZ3GGp2GoxFPNfEYS2g37keDruTNymT6v3
HuJo2VnZhDf5hSxNj8/VSoGLhvBOaxmLqbrVpPC7EGAZO4NGLLrTLBYIX2OjQg/dC4wiRqnUVIIxDQoVbIdJKCorngVxrkl+IMEI
Z+8gxSeLzNpbM7wlX8HTJWniqFxMcEkwp6hJxAt29jTNxlItyt97i6FpEgl495ECV4m+zxR5o/sPXd2lSMzZUYHEPLLmMkyoGfRu
xohgcEjBDPSWSni45Da3oDMZ5cTW82etpyK4M7BqR9/4K9Kzpeo4vkOoK4HtCVLZ44A=="/>
        </LOT>
    </UPD>
</ROCHE.LIAT.LOTS.R02>
```


New 4-digit lot with partial list


```
<ACK.R01>
    <HDR>
        <HDR.control_id V="470"/>
        <HDR.version_id V="POCT1"/>
        <HDR.creation_dttm V="2019-08-14T12:47:15+02:00"/>
    </HDR>
    <ACK>
        <ACK.type_cd V="AA"/>
        <ACK.ack_control_id V="26"/>
        <ACK.note_txt/>
    </ACK>
</ACK.R01>
```


ACK message from partial lot list

New 6-digit lot with partial list


```
<ROCHE.LIAT.LOTS.R02>
    <HDR>
        <HDR.message_type V="ROCHE.LIAT.LOTS.R02" SN="ROCHE" SV="1.0"/>
        <HDR.control_id V="32"/>
        <HDR.version_id V="POCT1"/>
        <HDR.creation_dttm V="2019-08-14T12:52:13+02:00"/>
    </HDR>
    <UPD>
        <UPD.action_cd V="I"/>
        <LOT>
            <LOT.lot_id V="FABA^80101Z^1.31"/>
```

```
            <LOT.lot_insert_id V="IFABA3408A11ZEI84B000000S"/>
            <LOT.parameters V="EI84B000000"/>
            <LOT.assay V="FABA"/>
            <LOT.expiration_date V="2034-08-31"/>
            <LOT.lot_number V="80101Z"/>
            <LOT.minimum_compatible_version V="1.31"/>
            <LOT.validation_dttm V="2018-01-01T00:00:00+00:00"/>
            <LOT.data V="ejhfNH9QDUKEixRu2On5PpctdO0CV2NHvlNEOwqv2JaJ9ZIdjUUISkifoMG/RjFqrz3zEpFPcRd
o2dq3F0yIpATWhAHqRcU782wmKdp0uGmE/c3Qmq2ptXLRRFzuptCYLTpeFqyq/Kc7KvgLFZ8UkEunqkcPRRQvstygEO8yUW7nGRR
P0EK3Q06HXpa0CVnbEmQ93y2zASiqGvG7OBoPJoyyGSh8YH+wPBlqHR/D1F7ghfiRrCQ073CHllP17SKmSV0DDP4tArPO5qQjoVY
3av8qsYTp5kSGpRPUCmvvN/JT/npDgZLl8xFVVyz10jMkJXzDlB28j+2DMGPCoMCj6w=="/>
        </LOT>
    </UPD>
</ROCHE.LIAT.LOTS.R02>
```


New 6-digit lot with partial list

4-digit lot deletion with partial list


```
<ROCHE.LIAT.LOTS.R02>
    <HDR>
        <HDR.message_type V="ROCHE.LIAT.LOTS.R02" SN="ROCHE" SV="1.0"/>
        <HDR.control_id V="39"/>
        <HDR.version_id V="POCT1"/>
        <HDR.creation_dttm V="2019-08-14T12:54:01+02:00"/>
    </HDR>
    <UPD>
        <UPD.action_cd V="D"/>
        <LOT>
            <LOT.lot_id V="SASA^A56B^1.26"/>
            <LOT.lot_insert_id V="ISASA3412A56BAAAAAAAAAAAT"/>
            <LOT.parameters V="AAAAAAAAAAA"/>
            <LOT.assay V="SASA"/>
            <LOT.expiration_date V="2034-12-31"/>
            <LOT.lot_number V="A56B"/>
            <LOT.minimum_compatible_version V="1.26"/>
            <LOT.validation_dttm V="2018-01-01T00:00:00+00:00"/>
            <LOT.data V="ORO2TpIanrlO4iOB3SYH/jIrzI7XXaqNcB0xQjalVBZ3GGp2GoxFPNfEYS2g37keDruTNymT6v3
HuJo2VnZhDf5hSxNj8/VSoGLhvBOaxmLqbrVpPC7EGAZO4NGLLrTLBYIX2OjQg/dC4wiRqnUVIIxDQoVbIdJKCorngVxrkl+IMEI
Z+8gxSeLzNpbM7wlX8HTJWniqFxMcEkwp6hJxAt29jTNxlItyt97i6FpEgl495ECV4m+zxR5o/sPXd2lSMzZUYHEPLLmMkyoGfRu
xohgcEjBDPSWSni45Da3oDMZ5cTW82etpyK4M7BqR9/4K9Kzpeo4vkOoK4HtCVLZ44A=="/>
        </LOT>
    </UPD>
</ROCHE.LIAT.LOTS.R02>
```


4-digit lot deletion with partial list

6-digit lot deletion with partial list


```
<ROCHE.LIAT.LOTS.R02>
    <HDR>
        <HDR.message_type V="ROCHE.LIAT.LOTS.R02" SN="ROCHE" SV="1.0"/>
        <HDR.control_id V="42"/>
        <HDR.version_id V="POCT1"/>
        <HDR.creation_dttm V="2019-08-14T12:55:21+02:00"/>
    </HDR>
    <UPD>
        <UPD.action_cd V="D"/>
        <LOT>
            <LOT.lot_id V="FABA^80101Z^1.31"/>
            <LOT.lot_insert_id V="IFABA3408A11ZEI84B000000S"/>
```

```
            <LOT.parameters V="EI84B000000"/>
            <LOT.assay V="FABA"/>
            <LOT.expiration_date V="2034-08-31"/>
            <LOT.lot_number V="80101Z"/>
            <LOT.minimum_compatible_version V="1.31"/>
            <LOT.validation_dttm V="2018-01-01T00:00:00+00:00"/>
            <LOT.data V="ejhfNH9QDUKEixRu2On5PpctdO0CV2NHvlNEOwqv2JaJ9ZIdjUUISkifoMG/RjFqrz3zEpFPcRd
o2dq3F0yIpATWhAHqRcU782wmKdp0uGmE/c3Qmq2ptXLRRFzuptCYLTpeFqyq/Kc7KvgLFZ8UkEunqkcPRRQvstygEO8yUW7nGRR
P0EK3Q06HXpa0CVnbEmQ93y2zASiqGvG7OBoPJoyyGSh8YH+wPBlqHR/D1F7ghfiRrCQ073CHllP17SKmSV0DDP4tArPO5qQjoVY
3av8qsYTp5kSGpRPUCmvvN/JT/npDgZLl8xFVVyz10jMkJXzDlB28j+2DMGPCoMCj6w=="/>
        </LOT>
    </UPD>
</ROCHE.LIAT.LOTS.R02>
```


6-digit lot deletion with partial list

Full lot list message


```
<ROCHE.LIAT.LOTS.R01>
    <HDR>
        <HDR.control_id V="47"/>
        <HDR.version_id V="POCT1"/>
        <HDR.creation_dttm V="2019-08-14T12:59:08+02:00"/>
    </HDR>
    <LOT>
        <LOT.lot_id V="SASA^A56B^1.26"/>
        <LOT.lot_insert_id V="ISASA3412A56BAAAAAAAAAAAT"/>
        <LOT.parameters V="AAAAAAAAAAA"/>
        <LOT.assay V="SASA"/>
        <LOT.expiration_date V="2034-12-31"/>
        <LOT.lot_number V="A56B"/>
        <LOT.minimum_compatible_version V="1.26"/>
        <LOT.validation_dttm V="2018-01-01T00:00:00+00:00"/>
        <LOT.data ENC="B64"V="ORO2TpIanrlO4iOB3SYH/jIrzI7XXaqNcB0xQjalVBZ3GGp2GoxFPNfEYS2g37keDruTNy
mT6v3HuJo2VnZhDf5hSxNj8/VSoGLhvBOaxmLqbrVpPC7EGAZO4NGLLrTLBYIX2OjQg/dC4wiRqnUVIIxDQoVbIdJKCorngVxrkl
+IMEIZ+8gxSeLzNpbM7wlX8HTJWniqFxMcEkwp6hJxAt29jTNxlItyt97i6FpEgl495ECV4m+zxR5o/sPXd2lSMzZUYHEPLLmMky
oGfRuxohgcEjBDPSWSni45Da3oDMZ5cTW82etpyK4M7BqR9/4K9Kzpeo4vkOoK4HtCVLZ44A=="/>
    </LOT>
    <LOT>
        <LOT.lot_id V="FABA^80101Z^1.31"/>
        <LOT.lot_insert_id V="IFABA3408A11ZEI84B000000S"/>
        <LOT.parameters V="EI84B000000"/>
        <LOT.assay V="FABA"/>
        <LOT.expiration_date V="2034-08-31"/>
        <LOT.lot_number V="80101Z"/>
        <LOT.minimum_compatible_version V="1.31"/>
        <LOT.validation_dttm V="2018-01-01T00:00:00+00:00"/>
        <LOT.data ENC="B64" V="ejhfNH9QDUKEixRu2On5PpctdO0CV2NHvlNEOwqv2JaJ9ZIdjUUISkifoMG/RjFqrz3zE
pFPcRdo2dq3F0yIpATWhAHqRcU782wmKdp0uGmE/c3Qmq2ptXLRRFzuptCYLTpeFqyq/Kc7KvgLFZ8UkEunqkcPRRQvstygEO8yU
W7nGRRP0EK3Q06HXpa0CVnbEmQ93y2zASiqGvG7OBoPJoyyGSh8YH+wPBlqHR/D1F7ghfiRrCQ073CHllP17SKmSV0DDP4tArPO5
qQjoVY3av8qsYTp5kSGpRPUCmvvN/JT/npDgZLl8xFVVyz10jMkJXzDlB28j+2DMGPCoMCj6w=="/>
    </LOT>
```

</ROCHE.LIAT.LOTS.R01>

Full lot list message

- Operator and lot lists (44)
- Lots (51)
- Acknowledgment object (ACK) (70)
- Header object (HDR) (75)
- Update Action object (UPD) (84)
- Lot object (LOT) (103)
- Lot full list messages (ROCHE.LIAT.LOTS.R01) (118)
- Lot partial list messages (ROCHE.LIAT.LOTS.R02) (119)

Observation topic

Patient-related observations


```
<REQ.R01>
    <HDR>
        <HDR.control_id V="4"/>
        <HDR.version_id V="POCT1"/>
        <HDR.creation_dttm V="2019-08-14T14:02:33+02:00"/>
    </HDR>
    <REQ>
        <REQ.request_cd V="ROBS"/>
    </REQ>
</REQ.R01>
```


Request message for observations


```
<OBS.R01>
    <HDR>
        <HDR.message_type V="OBS.R01"/>
        <HDR.control_id V="567"/>
        <HDR.version_id V="POCT1"/>
        <HDR.creation_dttm V="2019-08-14T14:09:38+02:00"/>
    </HDR>
    <SVC>
        <SVC.role_cd V="OBS"/>
        <SVC.observation_dttm V="2019-08-14T14:07:40+02:00"/>
        <PT>
            <PT.patient_id V="A-12398345"/>
            <OBS>
                <OBS.observation_id V="Influenza A (FABA)" SN="ROCHE" SV="1.0"/>
                <OBS.qualitative_value V="Detected" SN="ROCHE" SV="1.0"/>
                <OBS.method_cd V="M"/>
            </OBS>
            <OBS>
                <OBS.observation_id V="Influenza B (FABA)" SN="ROCHE" SV="1.0"/>
                <OBS.qualitative_value V="Detected" SN="ROCHE" SV="1.0"/>
                <OBS.method_cd V="M"/>
            </OBS>
        </PT>
        <OPR>
            <OPR.operator_id V="ADMIN"/>
        </OPR>
        <ORD>
            <ORD.universal_service_id V="Influenza Assay" SN="ROCHE" SV="1.0"/>
        </ORD>
        <RGT>
            <RGT.name V="FABA"/>
            <RGT.lot_number V="FABA^80101Z^1.31"/>
            <RGT.expiration_date V="2034-08-31T00:00:00+00:00"/>
        </RGT>
        <NTE>
            <NTE.text V="LIAT.Use=For In Vitro Diagnostic Use"/>
        </NTE>
        <NTE>
            <NTE.text V="LIAT.Run=00023"/>
        </NTE>
        <NTE>
            <NTE.text V="LIAT.Tube=00002"/>
```

```
        </NTE>
        <NTE>
            <NTE.text V="LIAT.Tube_id=TFABA3408A11Z00002R"/>
        </NTE>
        <NTE>
            <NTE.text V="LIAT.Approver=ADMIN"/>
        </NTE>
        <NTE>
            <NTE.text V="LIAT.Universal_service_id=Liat Influenza Assay"/>
        </NTE>
    </SVC>
</OBS.R01>
```

Patient result observation with patient verification

```
<OBS.R01>
    <HDR>
        <HDR.message_type V="OBS.R01"/>
        <HDR.control_id V="542"/>
        <HDR.version_id V="POCT1"/>
        <HDR.creation_dttm V="2019-08-14T14:02:51+02:00"/>
    </HDR>
    <SVC>
        <SVC.role_cd V="OBS"/>
        <SVC.observation_dttm V="2019-08-14T14:00:51+02:00"/>
        <PT>
            <PT.patient_id V="JAN"/>
            <OBS>
                <OBS.observation_id V="Influenza A (FABA)" SN="ROCHE" SV="1.0"/>
                <OBS.qualitative_value V="Detected" SN="ROCHE" SV="1.0"/>
                <OBS.method_cd V="M"/>
            </OBS>
            <OBS>
                <OBS.observation_id V="Influenza B (FABA)" SN="ROCHE" SV="1.0"/>
                <OBS.qualitative_value V="Not Detected" SN="ROCHE" SV="1.0"/>
                <OBS.method_cd V="M"/>
            </OBS>
        </PT>
        <OPR>
            <OPR.operator_id V="ADMIN"/>
        </OPR>
        <ORD>
            <ORD.universal_service_id V="Influenza Assay" SN="ROCHE" SV="1.0"/>
        </ORD>
        <RGT>
            <RGT.name V="FABA"/>
            <RGT.lot_number V="FABA^80101Z^1.31"/>
            <RGT.expiration_date V="2034-08-31T00:00:00+00:00"/>
        </RGT>
        <NTE>
            <NTE.text V="LIAT.Use=For In Vitro Diagnostic Use"/>
        </NTE>
        <NTE>
            <NTE.text V="LIAT.Run=00022"/>
        </NTE>
        <NTE>
            <NTE.text V="LIAT.Tube=00001"/>
        </NTE>
        <NTE>
            <NTE.text V="LIAT.Tube_id=TFABA3408A11Z00001S"/>
```

```
        </NTE>
        <NTE>
            <NTE.text V="LIAT.Approver=ADMIN"/>
        </NTE>
        <NTE>
            <NTE.text V="LIAT.Universal_service_id=Liat Influenza Assay"/>
        </NTE>
    </SVC>
</OBS.R01>
```


Patient result observation without patient verification


```
<OBS.R01>
    <HDR>
        <HDR.message_type V="OBS.R01"/>
        <HDR.control_id V="581"/>
        <HDR.version_id V="POCT1"/>
        <HDR.creation_dttm V="2019-08-14T14:21:39+02:00"/>
    </HDR>
    <SVC>
        <SVC.role_cd V="OBS"/>
        <SVC.observation_dttm V="2019-08-14T14:21:03+02:00"/>
        <PT>
            <PT.patient_id V="JAN"/>
            <OBS>
                <OBS.observation_id V="Influenza A (FABA)" SN="ROCHE" SV="1.0"/>
                <OBS.qualitative_value V="Not Detected" SN="ROCHE" SV="1.0"/>
                <OBS.method_cd V="M"/>
            </OBS>
            <OBS>
                <OBS.observation_id V="Influenza B (FABA)" SN="ROCHE" SV="1.0"/>
                <OBS.qualitative_value V="Detected" SN="ROCHE" SV="1.0"/>
                <OBS.method_cd V="M"/>
            </OBS>
        </PT>
        <OPR>
            <OPR.operator_id V="ADMIN"/>
        </OPR>
        <ORD>
            <ORD.universal_service_id V="Influenza Assay" SN="ROCHE" SV="1.0"/>
        </ORD>
        <RGT>
            <RGT.name V="FABA"/>
            <RGT.lot_number V="FABA^80101Z^1.31"/>
            <RGT.expiration_date V="2034-08-31T00:00:00+00:00"/>
        </RGT>
        <NTE>
            <NTE.text V="LIAT.Use=For In Vitro Diagnostic Use"/>
        </NTE>
        <NTE>
            <NTE.text V="LIAT.Run=00024"/>
        </NTE>
        <NTE>
            <NTE.text V="LIAT.Tube=00003"/>
        </NTE>
        <NTE>
            <NTE.text V="LIAT.Tube_id=TFABA3408A11Z00003Q"/>
        </NTE>
        <NTE>
            <NTE.text V="LIAT.Approver=ADMIN"/>
```

```
        </NTE>
        <NTE>
            <NTE.text V="LIAT.Universal_service_id=Liat Influenza Assay"/>
        </NTE>
        <NTE>
        <NTE.text V="LIAT.Patient_verification_failure_ignored=Run has been performed without patien
t verification"/>
        </NTE>
    </SVC>
</OBS.R01>
```

Patient result observation when patient verification is enabled but ignored

```
<ACK.R01>
    <HDR>
        <HDR.control_id V="5"/>
        <HDR.version_id V="POCT1"/>
        <HDR.creation_dttm V="2019-08-14T14:21:23+02:00"/>
    </HDR>
    <ACK>
        <ACK.type_cd V="AA"/>
        <ACK.ack_control_id V="581"/>
    </ACK>
</ACK.R01>
```

Positive ACK message

Quality control observation

```
<OBS.R02>
    <HDR>
        <HDR.message_type V="OBS.R02"/>
        <HDR.control_id V="861"/>
        <HDR.version_id V="POCT1"/>
        <HDR.creation_dttm V="2019-08-16T11:20:04+02:00"/>
    </HDR>
    <SVC>
        <SVC.role_cd V="LQC"/>
        <SVC.observation_dttm V="2019-08-15T11:17:37+02:00"/>
        <CTC>
            <CTC.name V="FABA control"/>
            <CTC.lot_number V="80101Z"/>
            <CTC.expiration_date V="2034-08-31T00:00:00+00:00"/>
            <CTC.level_cd V="M" SN="ROCHE" SV="1.0"/>
            <OBS>
                <OBS.observation_id V="Influenza A (FABA)" SN="ROCHE" SV="1.0"/>
                <OBS.qualitative_value V="Detected" SN="ROCHE" SV="1.0"/>
                <OBS.method_cd V="M"/>
            </OBS>
            <OBS>
                <OBS.observation_id V="Influenza B (FABA)" SN="ROCHE" SV="1.0"/>
                <OBS.qualitative_value V="Detected" SN="ROCHE" SV="1.0"/>
                <OBS.method_cd V="M"/>
            </OBS>
        </CTC>
        <OPR>
            <OPR.operator_id V="ADMIN"/>
        </OPR>
        <RGT>
            <RGT.name V="FABA"/>
            <RGT.lot_number V="FABA^80101Z^1.31"/>
```

```
            <RGT.expiration_date V="2034-08-31T00:00:00+00:00"/>
        </RGT>
        <NTE>
            <NTE.text V="LIAT.Use=For In Vitro Diagnostic Use"/>
        </NTE>
        <NTE>
            <NTE.text V="LIAT.Run=00040"/>
        </NTE>
        <NTE>
            <NTE.text V="LIAT.Tube=00002"/>
        </NTE>
        <NTE>
            <NTE.text V="LIAT.Tube_id=TFABA3408A11Z00002R"/>
        </NTE>
        <NTE>
            <NTE.text V="LIAT.Approver=ADMIN"/>
        </NTE>
        <NTE>
            <NTE.text V="LIAT.Universal_service_id=Liat Influenza Assay"/>
        </NTE>
        <NTE>
            <NTE.text V="LIAT.Lot_validation_status=Validated"/>
        </NTE>
    </SVC>
</OBS.R02>
```


QC observations


```
<ACK.R01>
    <HDR>
        <HDR.control_id V="6"/>
        <HDR.version_id V="POCT1"/>
        <HDR.creation_dttm V="2019-08-16T11:19:42+02:00"/>
    </HDR>
    <ACK>
        <ACK.type_cd V="AA"/>
        <ACK.ack_control_id V="861"/>
    </ACK>
</ACK.R01>
```


Positive ACK message


```
<EOT.R01>
    <HDR>
        <HDR.control_id V="862"/>
        <HDR.version_id V="POCT1"/>
        <HDR.creation_dttm V="2019-08-16T11:20:05+02:00"/>
    </HDR>
    <EOT>
        <EOT.topic_cd V="OBS"/>
    </EOT>
```

</EOT.R01>

End of Topic message

- Observations (results) (41)
- Acknowledgment object (ACK) (70)
- End of topic object (EOT) (73)
- Header object (HDR) (75)
- Observation object (OBS) (75)
- Patient object (PT) (85)
- Operator object (OPR) (78)
- Reagent object (RGT) (79)
- Service Object (SVC) (79)
- Request object (REQ) (80)
- Note object (NTE) (83)
- Acknowledgment message (ACK.R01) (108)
- Observation messages (OBS) (112)

Operators topic

Full list with one operator


```
<OPL.R01>
    <HDR>
        <HDR.message_type V="OPL.R01"/>
        <HDR.control_id V="30"/>
        <HDR.version_id V="POCT1"/>
        <HDR.creation_dttm V="2019-08-16T10:15:33+02:00"/>
    </HDR>
    <OPR>
        <OPR.operator_id V="USER4"/>
        <OPR.name V="Amy"/>
        <ACC>
            <ACC.method_cd V="FABA" SN="ROCHE" SV="1.0"/>
            <ACC.method_cd V="SASA" SN="ROCHE" SV="1.0"/>
            <ACC.password>
                10001
            </ACC.password>
            <ACC.permission_level_cd V="Administrator" SN="ROCHE" SV="1.0"/>
        </ACC>
        <NTE>
            <NTE.text V="LIAT.Contact=my contact info"/>
        </NTE>
        <NTE>
            <NTE.text V="LIAT.Department=RMD"/>
        </NTE>
        <NTE>
            <NTE.text V="LIAT.ReadGeneralUserManual=YES"/>
        </NTE>
        <NTE>
            <NTE.text V="LIAT.ChangePasswordOnNextLogin=YES"/>
        </NTE>
        <NTE>
            <NTE.text V="LIAT.Locked=NO"/>
        </NTE>
        <NTE>
            <NTE.text V="LIAT.BadgeBarcode=A45b97xA"/>
        </NTE>
        <NTE>
            <NTE.text V="LIAT.ReadGeneralUserManual=YES"/>
        </NTE>
        <NTE>
            <NTE.text V="LIAT.ReadAssayUserManuals=SASA,FABA"/>
        </NTE>
    </OPR>
</OPL.R01>
```


Full list of operators with one operator

Partial operator list with one insert and one delete


```
<OPL.R02>
    <HDR>
        <HDR.message_type V="OPL.R02"/>
        <HDR.control_id V="53"/>
        <HDR.version_id V="POCT1"/>
```

```
    <HDR.creation_dttm V="2019-08-16T10:24:08+02:00"/>
</HDR>
<UPD>
    <UPD.action_cd V="I"/>
    <OPR>
        <OPR.operator_id V="USER5"/>
        <OPR.name V="John"/>
        <ACC>
            <ACC.method_cd V="FABA" SN="ROCHE" SV="1.0"/>
            <ACC.method_cd V="SASA" SN="ROCHE" SV="1.0"/>
            <ACC.password>
                10001
            </ACC.password>
            <ACC.permission_level_cd V="Administrator" SN="ROCHE" SV="1.0"/>
        </ACC>
        <NTE>
            <NTE.text V="LIAT.Contact=my contact info"/>
        </NTE>
        <NTE>
            <NTE.text V="LIAT.Department=RMD"/>
        </NTE>
        <NTE>
            <NTE.text V="LIAT.ReadGeneralUserManual=YES"/>
        </NTE>
        <NTE>
            <NTE.text V="LIAT.ChangePasswordOnNextLogin=YES"/>
        </NTE>
        <NTE>
            <NTE.text V="LIAT.Locked=NO"/>
        </NTE>
        <NTE>
            <NTE.text V="LIAT.BadgeBarcode=A45v97xA"/>
        </NTE>
        <NTE>
            <NTE.text V="LIAT.ReadGeneralUserManual=YES"/>
        </NTE>
        <NTE>
            <NTE.text V="LIAT.ReadAssayUserManuals=SASA,FABA"/>
        </NTE>
    </OPR>
</UPD>
<UPD>
    <UPD.action_cd V="D"/>
    <OPR>
        <OPR.operator_id V="USER1"/>
    </OPR>
</UPD>
```

```
</OPL.R02>
```

Partial operator list with one insert and one delete

Operator and lot lists (44)
About operators (48)
Access control object (ACC) (69)
Header object (HDR) (75)
Operator object (OPR) (78)
Note object (NTE) (83)
Update Action object (UPD) (84)
Operator messages (OPL) (114)

Device configuration directive

Complete device configuration (all attributes)

```
<DTV.ROCHE.LIAT.CFG>
    <HDR>
        <HDR.message_type V="DTV.ROCHE.LIAT.CFG" SN="ROCHE" SV="1.0"/>
        <HDR.control_id V="3"/>
        <HDR.version_id V="POCT1"/>
        <HDR.creation_dttm V="2019-04-26T16:10:00+00:00"/>
    </HDR>
    <DTV>
        <DTV.command_cd V="SET_CONFIG" SN="ROCHE" SV="1.0"/>
    </DTV>
    <GEN_CFG>
        <GEN_CFG.DateTime.sntp V="false"/>
        <GEN_CFG.DateTime.Server V=""/>
        <GEN_CFG.DateTime.TimeZone V="Central European Standard Time"/>
        <GEN_CFG.DateTime.TimeFormat V="12"/>
        <GEN_CFG.DateTime.DateFormat V="yyyy-mm-dd"/>
        <GEN_CFG.Display.brightness V="7"/>
        <GEN_CFG.EnableTilt.EnableCheckTilt V="true"/>
        <GEN_CFG.Sound.sSoundInitialization V="Beep2"/>
        <GEN_CFG.Sound.sSoundBarcodeScan V="Beep3"/>
        <GEN_CFG.Sound.sSoundTubeInsert V="Beep4"/>
        <GEN_CFG.Sound.sSoundAssayFinish V="BUZZER"/>
        <GEN_CFG.Sound.sSoundTouchScreen V="Soft"/>
        <GEN_CFG.Sound.sSoundKeyClicks V="Soft"/>
        <GEN_CFG.Sound.SoundVolume V="1"/>
        <GEN_CFG.TubeInsertTime.iTubeInsertTime V="15"/>
        <GEN_CFG.AutoLock.autolocktime V="7"/>
        <GEN_CFG.Authentication.authenticationType V="User ID &amp; Password"/>
        <GEN_CFG.Languages.Language V="en-US"/>
        <GEN_CFG.AutoReboot.Time V="05:30"/>
        <GEN_CFG.Connectivity.Timeout V="30"/>
        <GEN_CFG.Connectivity.DMLAutoSend V="false"/>
        <GEN_CFG.Connectivity.DataSynchronizationUsers V="true"/>
        <GEN_CFG.Connectivity.DataSynchronizationAssayLots V="true"/>
        <GEN_CFG.Connectivity.DataSynchronizationLogEvents V="true"/>
        <GEN_CFG.Connectivity.DataSynchronizationInformation V="true"/>
        <GEN_CFG.Connectivity.DataSynchronizationWarningErrors V="true"/>
        <GEN_CFG.Connectivity.ConnectionInterval V="5"/>
        <GEN_CFG.BarcodeITF.Enabled V="false"/>
        <GEN_CFG.BarcodeITF.Checksum V="true"/>
        <GEN_CFG.BarcodeITF.FixLength V="One discrete length"/>
        <GEN_CFG.BarcodeITF.BarcodeLength V="10"/>
        <GEN_CFG.BarcodeCodabar.Enabled V="true"/>
        <GEN_CFG.BarcodeCodabar.TransmitStartStopChar V="false"/>
        <GEN_CFG.BarcodeCode39.Enabled V="true"/>
        <GEN_CFG.BarcodeCode39.Checksum V="true"/>
        <GEN_CFG.BarcodeCode93.Enabled V="true"/>
        <GEN_CFG.BarcodeEAN8.Enabled V="true"/>
        <GEN_CFG.BarcodeEAN13.Enabled V="true"/>
        <GEN_CFG.BarcodeGS1Databar14.Enabled V="true"/>
        <GEN_CFG.MachineName.sMachineName V="NewMachine"/>
        <GEN_CFG.PV.Verification V="prior run"/>
        <GEN_CFG.PV.VerificationType V="o"/>
        <GEN_CFG.PV.PatientMismatch V="run allowed"/>
```

```
        <GEN_CFG.PV.DisplayedData V="verbose"/>
        <GEN_CFG.PV.ManualConfirmation V="not required"/>
        <GEN_CFG.PRContent.CommunicationLog V="included"/>
        <GEN_CFG.PRContent.Runlog V="included"/>
        <GEN_CFG.PRContent.DataRange V="last30days"/>
        <GEN_CFG.PRContent.Sampleresults V="included"/>
        <GEN_CFG.PRContent.SampleID V="excluded"/>
        <GEN_CFG.PRContent.Users V="excluded"/>
        <GEN_CFG.PRSchedule.Creation V="on demand"/>
        <GEN_CFG.PRSchedule.DayOfMonth V="firstdayofmonth"/>
        <GEN_CFG.PRSchedule.DayOfWeek V="sunday"/>
        <GEN_CFG.PRSchedule.Destination V="remoteservicesystem"/>
        <GEN_CFG.PRSchedule.Time V="16"/>
        <GEN_CFG.PRSchedule.Frequency V="monthly"/>
        <GEN_CFG.Printers.InkLaserName V="HP Officejet Pro 8100"/>
        <GEN_CFG.Printers.InkLaserDescription V="Officejet Pro 8100 [126E24]"/>
        <GEN_CFG.Printers.InkLaserLocation V=""/>
        <GEN_CFG.Printers.InkLaserColorMode V="greyscale"/>
        <GEN_CFG.Printers.InkLaserConnection V="dnssd://Officejet%20Pro%208100%20%5B126E24%5D._pdlda
tastream._tcp.local/?uuid=1c852a4d-b800-1f08-abcd-a02bb8126e24"/>
        <GEN_CFG.Printers.ThermalName V="Brother QL-820NWB"/>
        <GEN_CFG.Printers.ThermalDescription V="Brother QL-820NWB"/>
        <GEN_CFG.Printers.ThermalLocation V=""/>
        <GEN_CFG.Printers.ThermalColorMode V="greyscale"/>
        <GEN_CFG.Printers.ThermalConnection V="dnssd://Brother%20QL-820NWB._ipp._tcp.local/?uuid=e32
48000-80ce-11db-8000-0080775abcab"/>
        <GEN_CFG.Printers.ReportPrinting.SelectedPrinter V="inklaser"/>
        <GEN_CFG.Printers.ResultPrinting.Autoprinting V="false"/>
        <GEN_CFG.Printers.ResultPrinting.SelectedPrinter V="thermal"/>
        <GEN_CFG.Printers.ResultPrinting.Manualprinting V="default"/>
        <GEN_CFG.SLNetworkShare1.Name V="Network Share 1"/>
        <GEN_CFG.SLNetworkShare1.ServerName V="10.138.206.208"/>
        <GEN_CFG.SLNetworkShare1.FolderPath V="SharedFolder_1"/>
        <GEN_CFG.SLNetworkShare1.UserName V="UserRW"/>
        <GEN_CFG.SLNetworkShare1.Password V="617617"/>
        <GEN_CFG.SLNetworkShare2.Name V="Network Share 2"/>
        <GEN_CFG.SLNetworkShare2.ServerName V="10.138.206.208"/>
        <GEN_CFG.SLNetworkShare2.FolderPath V="SharedFolder_2"/>
        <GEN_CFG.SLNetworkShare2.UserName V="UserRW"/>
        <GEN_CFG.SLNetworkShare2.Password V="617617"/>
        <GEN_CFG.SLNetworkShare3.Name V="Network Share 3"/>
        <GEN_CFG.SLNetworkShare3.ServerName V="10.138.206.208"/>
        <GEN_CFG.SLNetworkShare3.FolderPath V="SharedFolder_3"/>
        <GEN_CFG.SLNetworkShare3.UserName V="UserRW"/>
        <GEN_CFG.SLNetworkShare3.Password V="617617"/>
        <GEN_CFG.SLFTPShare1.Name V="FTP share 1"/>
        <GEN_CFG.SLFTPShare1.Type V="FTP"/>
        <GEN_CFG.SLFTPShare1.IP V="192.168.222.44"/>
        <GEN_CFG.SLFTPShare1.Port V="2554"/>
        <GEN_CFG.SLFTPShare1.FolderPath V="c:\testfolder"/>
        <GEN_CFG.SLFTPShare1.UserName V="SI"/>
        <GEN_CFG.SLFTPShare1.Password V="617617"/>
        <GEN_CFG.SLFTPShare2.Name V="FTP share 2"/>
        <GEN_CFG.SLFTPShare2.Type V="FTP"/>
        <GEN_CFG.SLFTPShare2.IP V="192.168.222.44"/>
        <GEN_CFG.SLFTPShare2.Port V="2554"/>
        <GEN_CFG.SLFTPShare2.FolderPath V="c:\testfolder"/>
        <GEN_CFG.SLFTPShare2.UserName V="SI"/>
        <GEN_CFG.SLFTPShare2.Password V="617617"/>
```

```
        <GEN_CFG.SLFTPShare3.Name V="FTP share 3"/>
        <GEN_CFG.SLFTPShare3.Type V="FTP"/>
        <GEN_CFG.SLFTPShare3.IP V="192.168.222.44"/>
        <GEN_CFG.SLFTPShare3.Port V="2554"/>
        <GEN_CFG.SLFTPShare3.FolderPath V="c:\testfolder"/>
        <GEN_CFG.SLFTPShare3.UserName V="SI"/>
        <GEN_CFG.SLFTPShare3.Password V="617617"/>
        <GEN_CFG.RemoteService.HTTPproxy V="false"/>
        <GEN_CFG.RemoteService.Server V=""/>
        <GEN_CFG.RemoteService.Port V=""/>
        <GEN_CFG.RemoteService.Authentication V="false"/>
        <GEN_CFG.RemoteService.UserName V=""/>
        <GEN_CFG.RemoteService.Password V=""/>
    </GEN_CFG>
</DTV.ROCHE.LIAT.CFG>
```

Complete device configuration (all attributes)

Partial device configuration (not all attributes)

```
<DTV.ROCHE.LIAT.CFG>
    <HDR>
        <HDR.message_type V="DTV.ROCHE.LIAT.CFG" SN="ROCHE" SV="1.0"/>
        <HDR.control_id V="3"/>
        <HDR.version_id V="POCT1"/>
        <HDR.creation_dttm V="2019-04-26T16:10:00+00:00"/>
    </HDR>
    <DTV>
        <DTV.command_cd V="SET_CONFIG" SN="ROCHE" SV="1.0"/>
    </DTV>
    <GEN_CFG>
        <GEN_CFG.Display.brightness V="7"/>
        <GEN_CFG.TubeInsertTime.iTubeInsertTime V="15"/>
        <GEN_CFG.AutoLock.autolocktime V="7"/>
     </GEN_CFG>
</DTV.ROCHE.LIAT.CFG>
```

Partial device configuration (not all attributes)

Device Configuration (53)

Header object (HDR) (75)

Generic configuration object (GEN_CFG) (88)

Device configuration message (DTV.ROCHE.LIAT.CFG) (116)

Communication ending

Terminate topic

```
<END.R01>
    <HDR>
        <HDR.control_id V="507"/>
        <HDR.version_id V="POCT1"/>
        <HDR.creation_dttm V="2019-08-14T13:21:45+02:00"/>
    </HDR>
    <TRM>
        <TRM.reason_cd V="ABN"/>
        <TRM.note_txt V="Timeout occurred."/>
    </TRM>
</END.R01>
```

Terminate message

```
<ACK.R01>
    <HDR>
        <HDR.control_id V="8"/>
        <HDR.version_id V="POCT1"/>
        <HDR.creation_dttm V="2019-08-14T13:21:28+02:00"/>
    </HDR>
    <ACK>
        <ACK.type_cd V=""/>
        <ACK.ack_control_id V="507"/>
    </ACK>
</ACK.R01>
```

Positive ACK message

Communication termination (28)

Acknowledgment object (ACK) (70)

Header object (HDR) (75)

Termination object (TRM) (83)

Acknowledgment message (ACK.R01) (108)

Termination message (END.R01) (115)

Keep alive message

```
<KPA.R01>
    <HDR>
        <HDR.control_id V="58"/>
        <HDR.version_id V="POCT1"/>
        <HDR.creation_dttm V="2019-08-14T13:13:36+02:00"/>
    </HDR>
</KPA.R01>
```

Keep alive message

Keep alive (33)

Keep alive message (KPA.R01) (111)

Example message logs

Communication logs with DML

This chapter contains logs of example communication between the **cobas**® Liat® System and the DML.
Messages are highlighted on each communication example:
Green for messages sent by the analyzer.
Blue for messages sent by the DMS.

In this chapter

6

FABA and SASA observation message log 151
FRTA observation message log. 156
SCFA observation message log 159

FABA and SASA observation message log

Patient and quality control results

SASA and FABA observation results

This section shows communication sent from and received by a host connected to the **cobas**® Liat® System.

- Hello message received: 11:19:38.058
- ACK sent: 11:19:38.175
- Device status received: 11:19:39.180
- ACK sent: 11:19:39.295
- Request sent: 11:19:41.595
- FABA patient observation and SASA patient observation received: 11:19:42.607
- ACK sent: 11:19:42.719
- FABA non-patient observation received: 11:19:43.729
- ACK sent: 11:19:43.842
- End of topic received: 11:19:44.845


```
<HEL.R01>
    <HDR>
        <HDR.control_id V="858"/>
        <HDR.version_id V="POCT1"/>
        <HDR.creation_dttm V="2019-08-16T11:19:59+02:00"/>
    </HDR>
    <DEV>
        <DEV.device_id V="f8:dc:7a:06:27:0c"/>
        <DEV.vendor_id V="ROCHE"/>
        <DEV.serial_id V="M1-E-10063"/>
        <DEV.manufacturer_name V="Roche Molecular Diagnostics"/>
        <DEV.sw_version V="3.3.0.4027"/>
        <DEV.device_name V="cobasLiat"/>
        <DCP>
            <DCP.application_timeout V="120"/>
        </DCP>
        <DSC>
            <DSC.connection_profile_cd V="SA"/>
            <DSC.topics_supported_cd V="D_EV"/>
            <DSC.max_message_sz V="614400"/>
        </DSC>
    </DEV>
</HEL.R01>

<ACK.R01>
    <HDR>
        <HDR.control_id V="2"/>
        <HDR.version_id V="POCT1"/>
        <HDR.creation_dttm V="2019-08-16T11:19:38+02:00"/>
    </HDR>
    <ACK>
        <ACK.type_cd V="AA"/>
        <ACK.ack_control_id V="858"/>
    </ACK>
</ACK.R01>
```

```
<DST.R01>
    <HDR>
        <HDR.control_id V="859"/>
        <HDR.version_id V="POCT1"/>
        <HDR.creation_dttm V="2019-08-16T11:19:59+02:00"/>
    </HDR>
    <DST>
        <DST.status_dttm V="2019-08-16T11:19:59+02:00"/>
        <DST.new_observations_qty V="3"/>
        <DST.new_events_qty V="321"/>
        <DST.condition_cd V="R"/>
    </DST>
</DST.R01>

<ACK.R01>
    <HDR>
        <HDR.control_id V="3"/>
        <HDR.version_id V="POCT1"/>
        <HDR.creation_dttm V="2019-08-16T11:19:39+02:00"/>
    </HDR>
    <ACK>
        <ACK.type_cd V="AA"/>
        <ACK.ack_control_id V="859"/>
    </ACK>
</ACK.R01>

<REQ.R01>
    <HDR>
        <HDR.control_id V="4"/>
        <HDR.version_id V="POCT1"/>
        <HDR.creation_dttm V="2019-08-16T11:19:41+02:00"/>
    </HDR>
    <REQ>
        <REQ.request_cd V="ROBS"/>
    </REQ>
</REQ.R01>

<OBS.R01>
    <HDR>
        <HDR.message_type V="OBS.R01"/>
        <HDR.control_id V="860"/>
        <HDR.version_id V="POCT1"/>
        <HDR.creation_dttm V="2019-08-16T11:20:03+02:00"/>
    </HDR>
    <SVC>
        <SVC.role_cd V="OBS"/>
        <SVC.observation_dttm V="2019-08-13T11:16:28+02:00"/>
        <PT>
            <PT.patient_id V="JAN"/>
            <OBS>
                <OBS.observation_id V="Influenza A (FABA)" SN="ROCHE" SV="1.0"/>
                <OBS.qualitative_value V="Detected" SN="ROCHE" SV="1.0"/>
                <OBS.method_cd V="M"/>
            </OBS>
            <OBS>
                <OBS.observation_id V="Influenza B (FABA)" SN="ROCHE" SV="1.0"/>
                <OBS.qualitative_value V="Not Detected" SN="ROCHE" SV="1.0"/>
                <OBS.method_cd V="M"/>
```

```
            </OBS>
        </PT>
        <OPR>
            <OPR.operator_id V="ADMIN"/>
        </OPR>
        <ORD>
            <ORD.universal_service_id V="Influenza Assay" SN="ROCHE" SV="1.0"/>
        </ORD>
        <RGT>
            <RGT.name V="FABA"/>
            <RGT.lot_number V="FABA^80101Z^1.31"/>
            <RGT.expiration_date V="2034-08-31T00:00:00+00:00"/>
        </RGT>
        <NTE>
            <NTE.text V="LIAT.Use=For In Vitro Diagnostic Use"/>
        </NTE>
        <NTE>
            <NTE.text V="LIAT.Run=00039"/>
        </NTE>
        <NTE>
            <NTE.text V="LIAT.Tube=00004"/>
        </NTE>
        <NTE>
            <NTE.text V="LIAT.Tube_id=TFABA3408A11Z00004P"/>
        </NTE>
        <NTE>
            <NTE.text V="LIAT.Approver=ADMIN"/>
        </NTE>
        <NTE>
            <NTE.text V="LIAT.Universal_service_id=Liat Influenza Assay"/>
        </NTE>
    </SVC>
    <SVC>
        <SVC.role_cd V="OBS"/>
        <SVC.observation_dttm V="2019-08-14T11:18:50+02:00"/>
        <PT>
            <PT.patient_id V="AMY"/>
            <OBS>
                <OBS.observation_id V="Strep A (SASA)" SN="ROCHE" SV="1.0"/>
                <OBS.qualitative_value V="Detected" SN="ROCHE" SV="1.0"/>
                <OBS.method_cd V="M"/>
            </OBS>
        </PT>
        <OPR>
            <OPR.operator_id V="ADMIN"/>
        </OPR>
        <ORD>
            <ORD.universal_service_id V="Strep A Assay" SN="ROCHE" SV="1.0"/>
        </ORD>
        <RGT>
            <RGT.name V="SASA"/>
            <RGT.lot_number V="SASA^80506B^1.28"/>
            <RGT.expiration_date V="2034-12-31T00:00:00+00:00"/>
        </RGT>
        <NTE>
            <NTE.text V="LIAT.Use=For In Vitro Diagnostic Use"/>
        </NTE>
        <NTE>
            <NTE.text V="LIAT.Run=00041"/>
```

```
        </NTE>
        <NTE>
            <NTE.text V="LIAT.Tube=00009"/>
        </NTE>
        <NTE>
            <NTE.text V="LIAT.Tube_id=TSASA3412A56B00009A"/>
        </NTE>
        <NTE>
            <NTE.text V="LIAT.Approver=ADMIN"/>
        </NTE>
        <NTE>
            <NTE.text V="LIAT.Universal_service_id=Liat Strep A Assay"/>
        </NTE>
    </SVC>
</OBS.R01>

<ACK.R01>
    <HDR>
        <HDR.control_id V="5"/>
        <HDR.version_id V="POCT1"/>
        <HDR.creation_dttm V="2019-08-16T11:19:42+02:00"/>
    </HDR>
    <ACK>
        <ACK.type_cd V="AA"/>
        <ACK.ack_control_id V="860"/>
    </ACK>
</ACK.R01>

<OBS.R02>
    <HDR>
        <HDR.message_type V="OBS.R02"/>
        <HDR.control_id V="861"/>
        <HDR.version_id V="POCT1"/>
        <HDR.creation_dttm V="2019-08-16T11:20:04+02:00"/>
    </HDR>
    <SVC>
        <SVC.role_cd V="LQC"/>
        <SVC.observation_dttm V="2019-08-15T11:17:37+02:00"/>
        <CTC>
            <CTC.name V="FABA control"/>
            <CTC.lot_number V="80101Z"/>
            <CTC.expiration_date V="2034-08-31T00:00:00+00:00"/>
            <CTC.level_cd V="M" SN="ROCHE" SV="1.0"/>
            <OBS>
                <OBS.observation_id V="Influenza A (FABA)" SN="ROCHE" SV="1.0"/>
                <OBS.qualitative_value V="Detected" SN="ROCHE" SV="1.0"/>
                <OBS.method_cd V="M"/>
            </OBS>
            <OBS>
                <OBS.observation_id V="Influenza B (FABA)" SN="ROCHE" SV="1.0"/>
                <OBS.qualitative_value V="Detected" SN="ROCHE" SV="1.0"/>
                <OBS.method_cd V="M"/>
            </OBS>
        </CTC>
        <OPR>
            <OPR.operator_id V="ADMIN"/>
        </OPR>
        <RGT>
            <RGT.name V="FABA"/>
```

```
            <RGT.lot_number V="FABA^80101Z^1.31"/>
            <RGT.expiration_date V="2034-08-31T00:00:00+00:00"/>
        </RGT>
        <NTE>
            <NTE.text V="LIAT.Use=For In Vitro Diagnostic Use"/>
        </NTE>
        <NTE>
            <NTE.text V="LIAT.Run=00040"/>
        </NTE>
        <NTE>
            <NTE.text V="LIAT.Tube=00002"/>
        </NTE>
        <NTE>
            <NTE.text V="LIAT.Tube_id=TFABA3408A11Z00002R"/>
        </NTE>
        <NTE>
            <NTE.text V="LIAT.Approver=ADMIN"/>
        </NTE>
        <NTE>
            <NTE.text V="LIAT.Universal_service_id=Liat Influenza Assay"/>
        </NTE>
        <NTE>
            <NTE.text V="LIAT.Lot_validation_status=Validated"/>
        </NTE>
    </SVC>
</OBS.R02>

<ACK.R01>
    <HDR>
        <HDR.control_id V="6"/>
        <HDR.version_id V="POCT1"/>
        <HDR.creation_dttm V="2019-08-16T11:19:42+02:00"/>
    </HDR>
    <ACK>
        <ACK.type_cd V="AA"/>
        <ACK.ack_control_id V="861"/>
    </ACK>
</ACK.R01>

<EOT.R01>
    <HDR>
        <HDR.control_id V="862"/>
        <HDR.version_id V="POCT1"/>
        <HDR.creation_dttm V="2019-08-16T11:20:05+02:00"/>
    </HDR>
    <EOT>
        <EOT.topic_cd V="OBS"/>
    </EOT>
</EOT.R01>
```

FRTA observation message log

This section shows communication sent from and received by a host connected to the **cobas**® Liat® System.

- Hello message received: 12:04:56.889
- ACK sent: 12:04:57.329
- Device status received: 12:04:58.335
- ACK sent: 12:04:58.448
- Request sent: 12:05:03.987
- FRTA patient observation received: 12:05:04.993
- ACK sent: 12:05:05.113
- End of topic: 12:05:06.114


```
<HEL.R01>
    <HDR>
        <HDR.control_id V="884"/>
        <HDR.version_id V="POCT1"/>
        <HDR.creation_dttm V="2019-08-16T12:05:18+02:00"/>
    </HDR>
    <DEV>
        <DEV.device_id V="f8:dc:7a:06:27:0c"/>
        <DEV.vendor_id V="ROCHE"/>
        <DEV.serial_id V="M1-E-10063"/>
        <DEV.manufacturer_name V="Roche Molecular Diagnostics"/>
        <DEV.sw_version V="3.3.0.4027"/>
        <DEV.device_name V="cobasLiat"/>
        <DCP>
            <DCP.application_timeout V="120"/>
        </DCP>
        <DSC>
            <DSC.connection_profile_cd V="SA"/>
            <DSC.topics_supported_cd V="D_EV"/>
            <DSC.max_message_sz V="614400"/>
        </DSC>
    </DEV>
</HEL.R01>

<ACK.R01>
    <HDR>
        <HDR.control_id V="884"/>
        <HDR.version_id V="POCT1"/>
        <HDR.creation_dttm V="2019-08-16T12:04:57+02:00"/>
    </HDR>
    <ACK>
        <ACK.type_cd V="AA"/>
        <ACK.ack_control_id V="884"/>
    </ACK>
</ACK.R01>

<DST.R01>
    <HDR>
        <HDR.control_id V="885"/>
        <HDR.version_id V="POCT1"/>
        <HDR.creation_dttm V="2019-08-16T12:05:18+02:00"/>
```

```
    </HDR>
    <DST>
        <DST.status_dttm V="2019-08-16T12:05:18+02:00"/>
        <DST.new_observations_qty V="1"/>
        <DST.new_events_qty V="321"/>
        <DST.condition_cd V="R"/>
    </DST>
</DST.R01>

<ACK.R01>
    <HDR>
        <HDR.control_id V="885"/>
        <HDR.version_id V="POCT1"/>
        <HDR.creation_dttm V="2019-08-16T12:04:58+02:00"/>
    </HDR>
    <ACK>
        <ACK.type_cd V="AA"/>
        <ACK.ack_control_id V="885"/>
    </ACK>
</ACK.R01>

<REQ.R01>
    <HDR>
        <HDR.control_id V="886"/>
        <HDR.version_id V="POCT1"/>
        <HDR.creation_dttm V="2019-08-16T12:05:03+02:00"/>
    </HDR>
    <REQ>
        <REQ.request_cd V="ROBS"/>
    </REQ>
</REQ.R01>

<OBS.R01>
    <HDR>
        <HDR.message_type V="OBS.R01"/>
        <HDR.control_id V="886"/>
        <HDR.version_id V="POCT1"/>
        <HDR.creation_dttm V="2019-08-16T12:05:25+02:00"/>
    </HDR>
    <SVC>
        <SVC.role_cd V="OBS"/>
        <SVC.observation_dttm V="2019-08-15T12:02:20+02:00"/>
        <PT>
            <PT.patient_id V="JOHN"/>
            <OBS>
                <OBS.observation_id V="Influenza A (FRTA)" SN="ROCHE" SV="1.0"/>
                <OBS.qualitative_value V="Detected" SN="ROCHE" SV="1.0"/>
                <OBS.method_cd V="M"/>
            </OBS>
            <OBS>
                <OBS.observation_id V="Influenza B (FRTA)" SN="ROCHE" SV="1.0"/>
                <OBS.qualitative_value V="Detected" SN="ROCHE" SV="1.0"/>
                <OBS.method_cd V="M"/>
            </OBS>
            <OBS>
                <OBS.observation_id V="RSV (FRTA)" SN="ROCHE" SV="1.0"/>
                <OBS.qualitative_value V="Detected" SN="ROCHE" SV="1.0"/>
                <OBS.method_cd V="M"/>
            </OBS>
```

```
        </PT>
        <OPR>
            <OPR.operator_id V="ADMIN"/>
        </OPR>
        <ORD>
            <ORD.universal_service_id V="Flu-RSV Assay" SN="ROCHE" SV="1.0"/>
        </ORD>
        <RGT>
            <RGT.name V="FRTA"/>
            <RGT.lot_number V="FRTA^80123X^1.28"/>
            <RGT.expiration_date V="2034-09-30T00:00:00+00:00"/>
        </RGT>
        <NTE>
            <NTE.text V="LIAT.Use=For In Vitro Diagnostic Use"/>
        </NTE>
        <NTE>
            <NTE.text V="LIAT.Run=00042"/>
        </NTE>
        <NTE>
            <NTE.text V="LIAT.Tube=00003"/>
        </NTE>
        <NTE>
            <NTE.text V="LIAT.Tube_id=TFRTA3409A1MX00003A"/>
        </NTE>
        <NTE>
            <NTE.text V="LIAT.Approver=ADMIN"/>
        </NTE>
        <NTE>
            <NTE.text V="LIAT.Universal_service_id=Liat Flu-RSV Assay"/>
        </NTE>
    </SVC>
</OBS.R01>

<ACK.R01>
    <HDR>
        <HDR.control_id V="887"/>
        <HDR.version_id V="POCT1"/>
        <HDR.creation_dttm V="2019-08-16T12:05:05+02:00"/>
    </HDR>
    <ACK>
        <ACK.type_cd V="AA"/>
        <ACK.ack_control_id V="886"/>
    </ACK>
</ACK.R01>

<EOT.R01>
    <HDR>
        <HDR.control_id V="887"/>
        <HDR.version_id V="POCT1"/>
        <HDR.creation_dttm V="2019-08-16T12:05:26+02:00"/>
    </HDR>
    <EOT>
        <EOT.topic_cd V="OBS"/>
    </EOT>
</EOT.R01>
```

SCFA observation message log

This section shows communication sent from and received by a host connected to the **cobas**® Liat® System.

- Hello message received: 19:25:30
- ACK sent: 19:25:32
- Device status received: 19:25:34
- ACK sent: 19:25:36
- Request sent: 19:25:38
- SCFA patient observation received: 19:25:40
- ACK sent: 19:25:42
- End of topic: 19:25:44


```
<HEL.R01>
    <HDR>
        <HDR.control_id V="903" />
        <HDR.version_id V="POCT1" />
        <HDR.creation_dttm V="2020-02-01T19:25:30+01:00" />
    </HDR>
    <DEV>
        <DEV.device_id V="f8:dc:7a:03:3a:6a" />
        <DEV.vendor_id V="ROCHE" />
        <DEV.serial_id V="M1-E-00547" />
        <DEV.manufacturer_name V="Roche Molecular Diagnostics" />
        <DEV.sw_version V="3.3.1.4061" />
        <DEV.device_name V="cobasLiat" />
        <DCP>
            <DCP.application_timeout V="120" />
        </DCP>
        <DSC>
            <DSC.connection_profile_cd V="SA" />
            <DSC.topics_supported_cd V="D_EV" />
            <DSC.max_message_sz V="614400" />
        </DSC>
    </DEV>
</HEL.R01>

<ACK.R01>
    <HDR>
        <HDR.control_id V="2" />
        <HDR.version_id V="POCT1" />
        <HDR.creation_dttm V="2020-02-01T19:25:32+01:00" />
    </HDR>
    <ACK>
        <ACK.type_cd V="AA" />
        <ACK.ack_control_id V="903" />
    </ACK>
</ACK.R01>

<DST.R01>
    <HDR>
        <HDR.control_id V="904" />
        <HDR.version_id V="POCT1" />
        <HDR.creation_dttm V="2020-02-01T19:25:34+01:00" />
```

```
    </HDR>
    <DST>
        <DST.status_dttm V="2020-02-01T19:25:34+01:00" />
        <DST.new_observations_qty V="1" />
        <DST.new_events_qty V="1" />
        <DST.condition_cd V="R" />
    </DST>
</DST.R01>

<ACK.R01>
    <HDR>
        <HDR.control_id V="3" />
        <HDR.version_id V="POCT1" />
        <HDR.creation_dttm V="2020-02-01T19:25:36+01:00" />
    </HDR>
    <ACK>
        <ACK.type_cd V="AA" />
        <ACK.ack_control_id V="904" />
    </ACK>
</ACK.R01>

<REQ.R01>
    <HDR>
        <HDR.control_id V="4" />
        <HDR.version_id V="POCT1" />
        <HDR.creation_dttm V="2020-02-01T19:25:38+01:00" />
    </HDR>
    <REQ>
        <REQ.request_cd V="ROBS" />
    </REQ>
</REQ.R01>

<OBS.R01>
    <HDR>
        <HDR.message_type V="OBS.R01" />
        <HDR.control_id V="905" />
        <HDR.version_id V="POCT1" />
        <HDR.creation_dttm V="2020-02-01T19:25:40+01:00" />
    </HDR>
    <SVC>
        <SVC.role_cd V="OBS" />
        <SVC.observation_dttm V="2020-02-01T19:25:40+01:00" />
        <PT>
            <PT.patient_id V="PAT002" />
            <OBS>
                <OBS.observation_id V="SARS-CoV-2 (SCFA)" SN="ROCHE" SV="1.0" />
                <OBS.qualitative_value V="Detected" SN="ROCHE" SV="1.0" />
                <OBS.method_cd V="M" />
            </OBS>
            <OBS>
                <OBS.observation_id V="Influenza A (SCFA)" SN="ROCHE" SV="1.0" />
                <OBS.qualitative_value V="Not Detected" SN="ROCHE" SV="1.0" />
                <OBS.method_cd V="M" />
            </OBS>
            <OBS>
                <OBS.observation_id V="Influenza B (SCFA)" SN="ROCHE" SV="1.0" />
                <OBS.qualitative_value V="Not Detected" SN="ROCHE" SV="1.0" />
                <OBS.method_cd V="M" />
            </OBS>
```

```
        </PT>
        <OPR>
            <OPR.operator_id V="ADMIN" />
        </OPR>
        <ORD>
            <ORD.universal_service_id V="SARS-CoV-2/Flu" SN="ROCHE" SV="1.0" />
        </ORD>
        <RGT>
            <RGT.name V="SCFA" />
            <RGT.lot_number V="SCFA^20126A^1.0" />
            <RGT.expiration_date V="2030-01-31T00:00:00+00:00" />
        </RGT>
        <NTE>
            <NTE.text V="LIAT.Use=EUA/IVD" />
        </NTE>
        <NTE>
            <NTE.text V="LIAT.Run=00012" />
        </NTE>
        <NTE>
            <NTE.text V="LIAT.Tube=00013" />
        </NTE>
        <NTE>
            <NTE.text V="LIAT.Tube_id=TSCFA3001E1PA013V" />
        </NTE>
        <NTE>
            <NTE.text V="LIAT.Approver=ADMIN" />
        </NTE>
        <NTE>
            <NTE.text V="LIAT.Universal_service_id=Liat SARS-CoV-2/Flu" />
        </NTE>
    </SVC>
</OBS.R01>

<ACK.R01>
    <HDR>
        <HDR.control_id V="5" />
        <HDR.version_id V="POCT1" />
        <HDR.creation_dttm V="2020-02-01T19:25:42+01:00" />
    </HDR>
    <ACK>
        <ACK.type_cd V="AA" />
        <ACK.ack_control_id V="905" />
    </ACK>
</ACK.R01>

<EOT.R01>
    <HDR>
        <HDR.control_id V="906"/>
        <HDR.version_id V="POCT1"/>
        <HDR.creation_dttm V="2020-02-01T19:25:44+01:00"/>
    </HDR>
    <EOT>
        <EOT.topic_cd V="OBS"/>
    </EOT>
</EOT.R01>
```

Additional examples

In this chapter

7

Communication scenarios. 165
Communication scenario 1 – Wrong system setting to receive a device configuration. 166
Communication scenario 2 – Wrong instrument state to receive a device configuration 169
Communication scenario 3 – Device configuration file with an invalid parameter 171
Communication scenario 4– Device configuration successfully synchronized. 174
Communication scenario 5 – Send a validated assay lot to a DMS 178
Communication scenario 6 – Send a validated assay lot from DMS to an instrument 185
Communication scenario 7 – Liat sends automatically a result to a DMS 189
Communication scenario 8 – Activate the patient verification workflow via DMS 193
Communication scenario 9 – Patient verification passed – run performed. 197
Communication scenario 10 – Patient verification failed – run prevented 205
Communication scenario 11 – Wrong instrument state does not accept a user list. 209
Communication scenario 12 – Replace operators list via DMS 213
Communication scenario 13 – Delete and add operators via DMS 218

Communication scenarios

General information and preconditions

- Relevant messages are highlighted on each communication example:
  - Green for messages sent by the analyzer.
  - Blue for messages sent by the DMS.
  - Relevant data for particular scenario is in bold.
- For the current **configuration/preconditions** on each scenario, screen captures are shown.
- Most of the scenarios are sequential, and the provided order is important to show how the data and configuration changes on the analyzer, after the messages are exchanged.

In this section

Communication scenario 1 - Wrong system setting to receive a device configuration (166)

Communication scenario 2 - Wrong instrument state to receive a device configuration (169)

Communication scenario 3 - Device configuration file with an invalid parameter (171)

Communication scenario 4- Device configuration successfully synchronized (174)

Communication scenario 5 - Send a validated assay lot to a DMS (178)

Communication scenario 6 - Send a validated assay lot from DMS to an instrument (185)

Communication scenario 7 - Liat sends automatically a result to a DMS (189)

Communication scenario 8 - Activate the patient verification workflow via DMS (193)

Communication scenario 9 - Patient verification passed - run performed (197)

Communication scenario 10 - Patient verification failed - run prevented (205)

Communication scenario 11 - Wrong instrument state does not accept a user list (209)

Communication scenario 12 - Replace operators list via DMS (213)

Communication scenario 13 - Delete and add operators via DMS (218)

Communication scenario 1 – Wrong system setting to receive a device configuration

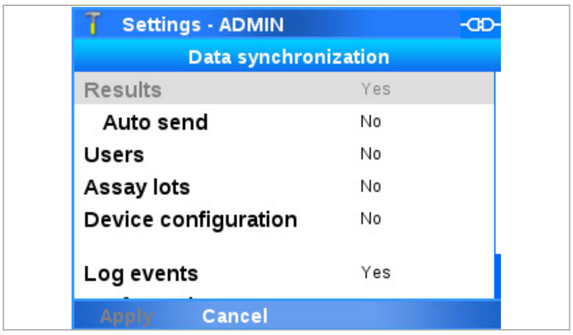

- **Purpose:** to see how the Liat does not accept (escapes) a Device Configuration directive, as it is explicitly not accepting them.
- **Configuration/Preconditions:** Device Config disabled.

Steps

1. DMS: send a DevConf when not logged on.
2. Liat: ESC

Direction	Message type	Comment
Analyzer→DMS	HEL.R01	
DMS→analyzer	ACK.R01	
Analyzer→DMS	DST.R01	
DMS→analyzer	ACK.R01	Handshake successful
DMS→analyzer	DTV.ROCHE.LIAT.CFG	DMS sends DevConf.
Analyzer→DMS	ESC.R01	Liat rejects it because **Device configuration** (on the Data synchronization screen) is “no”
Analyzer→DMS	END.R01	Liat: close connection
DMS→analyzer	ACK.R01	DMS: connection closed


```
<HEL.R01>
    <HDR>
        <HDR.control_id V="985"/>
        <HDR.version_id V="POCT1"/>
        <HDR.creation_dttm V="2019-12-19T08:30:28-05:00"/>
    </HDR>
    <DEV>
        <DEV.device_id V="f8:dc:7a:1c:a3:c9"/>
        <DEV.vendor_id V="ROCHE"/>
        <DEV.serial_id V="M1-E-16036"/>
        <DEV.manufacturer_name V="Roche Molecular Diagnostics"/>
        <DEV.sw_version V="3.3.0.4027"/>
        <DEV.device_name V="cobasLiat"/>
        <DCP>
            <DCP.application_timeout V="30"/>
        </DCP>
        <DSC>
            <DSC.connection_profile_cd V="SA"/>
            <DSC.topics_supported_cd V="D_EV"/>
            <DSC.max_message_sz V="614400"/>
        </DSC>
    </DEV>
</HEL.R01>
<ACK.R01>
    <HDR>
```

```
        <HDR.control_id V="2"/>
        <HDR.version_id V="POCT1"/>
        <HDR.creation_dttm V="2019-12-19T14:30:14+01:00"/>
    </HDR>
    <ACK>
        <ACK.type_cd V="AA"/>
        <ACK.ack_control_id V="985"/>
    </ACK>
</ACK.R01>
<DST.R01>
    <HDR>
        <HDR.control_id V="986"/>
        <HDR.version_id V="POCT1"/>
        <HDR.creation_dttm V="2019-12-19T08:30:28-05:00"/>
    </HDR>
    <DST>
        <DST.status_dttm V="2019-12-19T08:30:28-05:00"/>
        <DST.new_observations_qty V="3"/>
        <DST.new_events_qty V="76"/>
        <DST.condition_cd V="S"/>
    </DST>
</DST.R01>
<ACK.R01>
    <HDR>
        <HDR.control_id V="3"/>
        <HDR.version_id V="POCT1"/>
        <HDR.creation_dttm V="2019-12-19T14:30:15+01:00"/>
    </HDR>
    <ACK>
        <ACK.type_cd V="AA"/>
        <ACK.ack_control_id V="986"/>
    </ACK>
</ACK.R01>
<DTV.ROCHE.LIAT.CFG>
    <HDR>
        <HDR.message_type V="DTV.ROCHE.LIAT.CFG" SN="ROCHE" SV="1.0"/>
        <HDR.control_id V="4"/>
        <HDR.version_id V="POCT1"/>
        <HDR.creation_dttm V="2019-12-19T14:30:17+01:00"/>
    </HDR>
    <DTV>
        <DTV.command_cd V="SET_CONFIG" SN="ROCHE" SV="1.0"/>
    </DTV>
    <GEN_CFG>
       <GEN_CFG.DateTime.sntp V="false"/>
        <GEN_CFG.DateTime.Server V=""/>
        <GEN_CFG.DateTime.TimeZone V="Eastern Standard Time"/>
        <GEN_CFG.DateTime.TimeFormat V="12"/>
        <GEN_CFG.DateTime.DateFormat V="yyyy-mm-dd"/>
        <GEN_CFG.Display.brightness V="7"/>
        <GEN_CFG.EnableTilt.EnableCheckTilt V="true"/>
        <GEN_CFG.TubeInsertTime.iTubeInsertTime V="15"/>
        <GEN_CFG.AutoLock.autolocktime V="7"/>
        <GEN_CFG.Authentication.authenticationType V="User ID & Password"/>
        <GEN_CFG.Languages.Language V="en-US"/>
        <GEN_CFG.AutoReboot.Time V="05:30"/>
        <GEN_CFG.Connectivity.Timeout V="30"/>
        <GEN_CFG.Connectivity.DMLAutoSend V="false"/>
        <GEN_CFG.Connectivity.DataSynchronizationUsers V="false"/>
```

```
        <GEN_CFG.Connectivity.DataSynchronizationAssayLots V="false"/>
        <GEN_CFG.Connectivity.DataSynchronizationLogEvents V="false"/>
        <GEN_CFG.Connectivity.DataSynchronizationInformation V="false"/>
        <GEN_CFG.Connectivity.DataSynchronizationWarningErrors V="false"/>
        <GEN_CFG.Connectivity.ConnectionInterval V="05"/>
    </GEN_CFG>
</DTV.ROCHE.LIAT.CFG>
<ESC.R01>
    <HDR>
        <HDR.control_id V="987"/>
        <HDR.version_id V="POCT1"/>
        <HDR.creation_dttm V="2019-12-19T08:30:31-05:00"/>
    </HDR>
    <ESC>
        <ESC.esc_control_id V="4"/>
        <ESC.detail_cd V="TOP"/>
        <ESC.note_txt V="Message not accepted."/>
   </ESC>
</ESC.R01>
<END.R01>
    <HDR>
        <HDR.control_id V="988"/>
        <HDR.version_id V="POCT1"/>
        <HDR.creation_dttm V="2019-12-19T08:31:01-05:00"/>
    </HDR>
    <TRM>
        <TRM.reason_cd V="ABN"/>
        <TRM.note_txt V="Timeout occurred."/>
    </TRM>
</END.R01>
<ACK.R01>
    <HDR>
        <HDR.control_id V="5"/>
        <HDR.version_id V="POCT1"/>
        <HDR.creation_dttm V="2019-12-19T14:30:48+01:00"/>
    </HDR>
    <ACK>
        <ACK.type_cd V="AA"/>
        <ACK.ack_control_id V="988"/>
    </ACK>
</ACK.R01>
```

Communication scenario 2 – Wrong instrument state to receive a device configuration

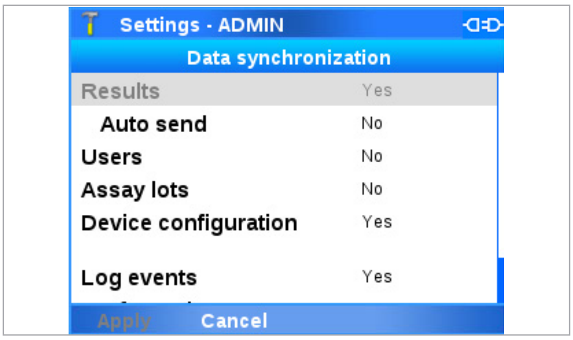

- **Purpose:** to see how the Liat does not accept (escapes) a Device Configuration directive, as this was received when the instrument was not in “stand-by”.
- **Configuration/Preconditions:** Device config enabled.

Steps

1. DMS: send a DevConf when logged on.
2. Liat: ESC

Direction	Message type	Comment
Analyzer➔DMS	HEL.R01	
DMS➔analyzer	ACK.R01	
Analyzer➔DMS	DST.R01	
DMS➔analyzer	ACK.R01	Handshake successful
DMS➔analyzer	DTV.ROCHE.LIAT.CFG	DMS sends Device Configuration directive.
Analyzer➔DMS	ESC.R01	Liat rejects it: instrument state is “ready”, and should be “stand-by”
Analyzer➔DMS	END.R01	Liat: close connection
DMS➔analyzer	ACK.R01	DMS: connection closed


```
<HEL.R01>
    <HDR>
        <HDR.control_id V="993"/>
        <HDR.version_id V="POCT1"/>
        <HDR.creation_dttm V="2019-12-19T08:40:28-05:00"/>
    </HDR>
    <DEV>
        <DEV.device_id V="f8:dc:7a:1c:a3:c9"/>
        <DEV.vendor_id V="ROCHE"/>
        <DEV.serial_id V="M1-E-16036"/>
        <DEV.manufacturer_name V="Roche Molecular Diagnostics"/>
        <DEV.sw_version V="3.3.0.4027"/>
        <DEV.device_name V="cobasLiat"/>
        <DCP>
            <DCP.application_timeout V="30"/>
        </DCP>
        <DSC>
            <DSC.connection_profile_cd V="SA"/>
            <DSC.topics_supported_cd V="D_EV"/>
            <DSC.max_message_sz V="614400"/>
        </DSC>
    </DEV>
</HEL.R01>
<ACK.R01>
    <HDR>
        <HDR.control_id V="2"/>
```

```
        <HDR.version_id V="POCT1"/>
        <HDR.creation_dttm V="2019-12-19T14:40:14+01:00"/>
    </HDR>
    <ACK>
        <ACK.type_cd V="AA"/>
        <ACK.ack_control_id V="993"/>
    </ACK>
</ACK.R01>
<DST.R01>
    <HDR>
        <HDR.control_id V="994"/>
        <HDR.version_id V="POCT1"/>
        <HDR.creation_dttm V="2019-12-19T08:40:28-05:00"/>
    </HDR>
    <DST>
        <DST.status_dttm V="2019-12-19T08:40:28-05:00"/>
        <DST.new_observations_qty V="3"/>
        <DST.new_events_qty V="78"/>
        <DST.condition_cd V="R"/>
    </DST>
</DST.R01>
<ACK.R01>
    <HDR>
        <HDR.control_id V="3"/>
        <HDR.version_id V="POCT1"/>
        <HDR.creation_dttm V="2019-12-19T14:40:15+01:00"/>
    </HDR>
    <ACK>
        <ACK.type_cd V="AA"/>
        <ACK.ack_control_id V="994"/>
    </ACK>
</ACK.R01>
<DTV.ROCHE.LIAT.CFG>
    <HDR>
        <HDR.message_type V="DTV.ROCHE.LIAT.CFG" SN="ROCHE" SV="1.0"/>
        <HDR.control_id V="4"/>
        <HDR.version_id V="POCT1"/>
        <HDR.creation_dttm V="2019-12-19T14:40:17+01:00"/>
    </HDR>
    <DTV>
        <DTV.command_cd V="SET_CONFIG" SN="ROCHE" SV="1.0"/>
    </DTV>
    <GEN_CFG>
        <GEN_CFG.DateTime.sntp V="false"/>
        <GEN_CFG.DateTime.Server V=""/>
        <GEN_CFG.DateTime.TimeZone V="Eastern Standard Time"/>
        <GEN_CFG.DateTime.TimeFormat V="12"/>
        <GEN_CFG.DateTime.DateFormat V="yyyy-mm-dd"/>
        <GEN_CFG.Display.brightness V="7"/>
        <GEN_CFG.EnableTilt.EnableCheckTilt V="true"/>
        <GEN_CFG.TubeInsertTime.iTubeInsertTime V="15"/>
        <GEN_CFG.AutoLock.autolocktime V="7"/>
        <GEN_CFG.Authentication.authenticationType V="User ID & Password"/>
        <GEN_CFG.Languages.Language V="en-US"/>
        <GEN_CFG.AutoReboot.Time V="05:30"/>
        <GEN_CFG.Connectivity.Timeout V="30"/>
        <GEN_CFG.Connectivity.DMLAutoSend V="false"/>
        <GEN_CFG.Connectivity.DataSynchronizationUsers V="false"/>
        <GEN_CFG.Connectivity.DataSynchronizationAssayLots V="false"/>
```

```
        <GEN_CFG.Connectivity.DataSynchronizationLogEvents V="false"/>
        <GEN_CFG.Connectivity.DataSynchronizationInformation V="false"/>
        <GEN_CFG.Connectivity.DataSynchronizationWarningErrors V="false"/>
        <GEN_CFG.Connectivity.ConnectionInterval V="05"/>
    </GEN_CFG>
</DTV.ROCHE.LIAT.CFG>
<ESC.R01>
    <HDR>
        <HDR.control_id V="995"/>
        <HDR.version_id V="POCT1"/>
        <HDR.creation_dttm V="2019-12-19T08:40:31-05:00"/>
    </HDR>
    <ESC>
        <ESC.esc_control_id V="4"/>
        <ESC.detail_cd V="TOP"/>
        <ESC.note_txt V="Message not accepted."/>
    </ESC>
</ESC.R01>
<END.R01>
    <HDR>
        <HDR.control_id V="996"/>
        <HDR.version_id V="POCT1"/>
        <HDR.creation_dttm V="2019-12-19T08:41:01-05:00"/>
    </HDR>
    <TRM>
        <TRM.reason_cd V="NRM"/>
        <TRM.note_txt V="Conversation was terminated by the user."/>
    </TRM>
</END.R01>
<ACK.R01>
   <HDR>
        <HDR.control_id V="5"/>
        <HDR.version_id V="POCT1"/>
        <HDR.creation_dttm V="2019-12-19T14:40:48+01:00"/>
    </HDR>
    <ACK>
        <ACK.type_cd V="AA"/>
        <ACK.ack_control_id V="996"/>
    </ACK>
</ACK.R01>
```


Communication scenario 3 – Device configuration file with an invalid parameter

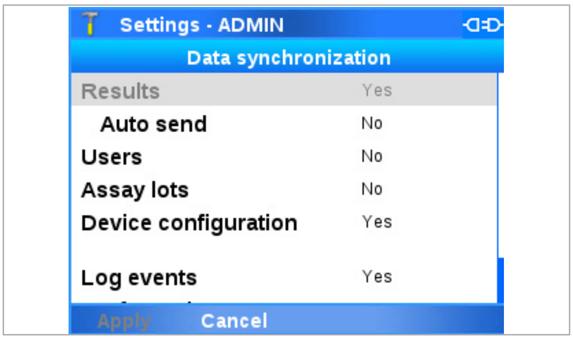

- **Purpose:** to see how the Liat does not accept (rejects with an “Error ACK”) a wrong Device Configuration directive.
- **Configuration/Preconditions:** Device configuration enabled

Steps

1. DMS: send a wrong Device Configuration directive (with Autosend = INT, instead of a BOOLEAN).
2. Liat: error acknowledgment

Direction	Message type	Comment
Analyzer➔DMS	HEL.R01	
DMS➔analyzer	ACK.R01	
Analyzer➔DMS	DST.R01	
DMS➔analyzer	ACK.R01	Handshake successful
DMS➔analyzer	DTV.ROCHE.LIAT.CFG	DMS sends Device Configuration directive with incorrect parameter.
Analyzer➔DMS	ACK.R01	Liat acknowledges with an error (does not accept the data received).
Analyzer➔DMS	END.R01	Liat: close connection
DMS➔analyzer	ACK.R01	DMS: connection closed

```
<HEL.R01>
    <HDR>
        <HDR.control_id V="1005"/>
        <HDR.version_id V="POCT1"/>
        <HDR.creation_dttm V="2019-12-19T08:55:28-05:00"/>
    </HDR>
    <DEV>
        <DEV.device_id V="f8:dc:7a:1c:a3:c9"/>
        <DEV.vendor_id V="ROCHE"/>
        <DEV.serial_id V="M1-E-16036"/>
        <DEV.manufacturer_name V="Roche Molecular Diagnostics"/>
        <DEV.sw_version V="3.3.0.4027"/>
        <DEV.device_name V="cobasLiat"/>
        <DCP>
            <DCP.application_timeout V="30"/>
            <DCP.vendor_specific>
                DTV.ROCHE.LIAT.CFG
            </DCP.vendor_specific>
        </DCP>
        <DSC>
            <DSC.connection_profile_cd V="SA"/>
            <DSC.topics_supported_cd V="D_EV"/>
            <DSC.topics_supported_cd V="DTV"/>
            <DSC.max_message_sz V="614400"/>
        </DSC>
    </DEV>
</HEL.R01>
<ACK.R01>
    <HDR>
        <HDR.control_id V="2"/>
        <HDR.version_id V="POCT1"/>
        <HDR.creation_dttm V="2019-12-19T14:55:14+01:00"/>
    </HDR>
    <ACK>
        <ACK.type_cd V="AA"/>
        <ACK.ack_control_id V="1005"/>
    </ACK>
</ACK.R01>
<DST.R01>
    <HDR>
        <HDR.control_id V="1006"/>
```

```
        <HDR.version_id V="POCT1"/>
        <HDR.creation_dttm V="2019-12-19T08:55:29-05:00"/>
    </HDR>
    <DST>
        <DST.status_dttm V="2019-12-19T08:55:29-05:00"/>
        <DST.new_observations_qty V="3"/>
        <DST.new_events_qty V="82"/>
        <DST.condition_cd V="S"/>
    </DST>
</DST.R01>
<ACK.R01>
    <HDR>
        <HDR.control_id V="3"/>
        <HDR.version_id V="POCT1"/>
        <HDR.creation_dttm V="2019-12-19T14:55:16+01:00"/>
    </HDR>
    <ACK>
        <ACK.type_cd V="AA"/>
        <ACK.ack_control_id V="1006"/>
    </ACK>
</ACK.R01>
<DTV.ROCHE.LIAT.CFG>
    <HDR>
        <HDR.message_type V="DTV.ROCHE.LIAT.CFG" SN="ROCHE" SV="1.0"/>
        <HDR.control_id V="4"/>
        <HDR.version_id V="POCT1"/>
        <HDR.creation_dttm V="2019-12-19T14:55:18+01:00"/>
    </HDR>
    <DTV>
        <DTV.command_cd V="SET_CONFIG" SN="ROCHE" SV="1.0"/>
    </DTV>
    <GEN_CFG>
        <GEN_CFG.DateTime.sntp V="false"/>
        <GEN_CFG.DateTime.Server V=""/>
        <GEN_CFG.DateTime.TimeZone V="Eastern Standard Time"/>
        <GEN_CFG.DateTime.TimeFormat V="12"/>
        <GEN_CFG.DateTime.DateFormat V="yyyy-mm-dd"/>
        <GEN_CFG.Display.brightness V="7"/>
        <GEN_CFG.EnableTilt.EnableCheckTilt V="true"/>
        <GEN_CFG.TubeInsertTime.iTubeInsertTime V="15"/>
        <GEN_CFG.AutoLock.autolocktime V="7"/>
        <GEN_CFG.Authentication.authenticationType V="User ID & Password"/>
        <GEN_CFG.Languages.Language V="en-US"/>
        <GEN_CFG.AutoReboot.Time V="05:30"/>
        <GEN_CFG.Connectivity.Timeout V="30"/>
        <GEN_CFG.Connectivity.DMLAutoSend V="3"/>
        <GEN_CFG.Connectivity.DataSynchronizationUsers V="false"/>
        <GEN_CFG.Connectivity.DataSynchronizationAssayLots V="false"/>
        <GEN_CFG.Connectivity.DataSynchronizationLogEvents V="false"/>
        <GEN_CFG.Connectivity.DataSynchronizationInformation V="false"/>
        <GEN_CFG.Connectivity.DataSynchronizationWarningErrors V="false"/>
        <GEN_CFG.Connectivity.ConnectionInterval V="05"/>
    </GEN_CFG>
</DTV.ROCHE.LIAT.CFG>
<ACK.R01>
    <HDR>
        <HDR.control_id V="1007"/>
        <HDR.version_id V="POCT1"/>
        <HDR.creation_dttm V="2019-12-19T08:55:32-05:00"/>
```

```
    </HDR>
    <ACK>
        <ACK.type_cd V="AE"/>
        <ACK.ack_control_id V="4"/>
        <ACK.note_txt V="Unable to parse POCT1A message."/>
    </ACK>
</ACK.R01>
<END.R01>
    <HDR>
        <HDR.control_id V="1008"/>
        <HDR.version_id V="POCT1"/>
        <HDR.creation_dttm V="2019-12-19T08:56:03-05:00"/>
    </HDR>
    <TRM>
        <TRM.reason_cd V="NRM"/>
        <TRM.note_txt V="There are no more commands to process."/>
    </TRM>
</END.R01>
<ACK.R01>
    <HDR>
        <HDR.control_id V="5"/>
        <HDR.version_id V="POCT1"/>
        <HDR.creation_dttm V="2019-12-19T14:55:50+01:00"/>
    </HDR>
    <ACK>
        <ACK.type_cd V="AA"/>
        <ACK.ack_control_id V="1008"/>
    </ACK>
</ACK.R01>
```

Communication scenario 4- Device configuration successfully synchronized

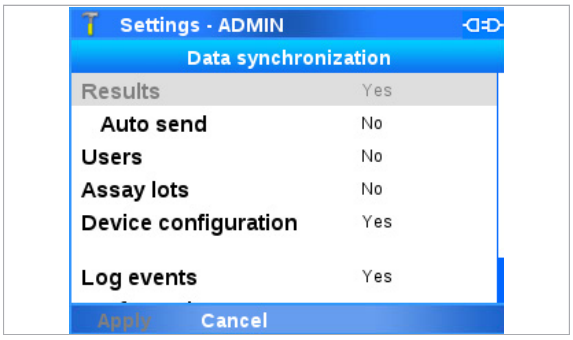

- **Purpose:** to see how the Liat accepts a Device Configuration directive.
- **Configuration/Preconditions:** Device Configuration enabled

Steps

1. DMS: send a correct Device Configuration directive with: Lots enabled, Autosend true.
2. Liat: acknowledgment

Direction	Message type	Comment
Analyzer➔DMS	HEL.R01	
DMS➔analyzer	ACK.R01	
Analyzer➔DMS	DST.R01	

Direction	Message type	Comment
DMS➔analyzer	ACK.R01	Handshake successful
DMS➔analyzer	DTV.ROCHE.LIAT.CFG	DMS sends DevConf.
Analyzer➔DMS	ACK.R01	Liat accepts the DevConf. -> data sync.
Analyzer➔DMS	END.R01	Liat: close connection
DMS➔analyzer	ACK.R01	DMS: connection closed

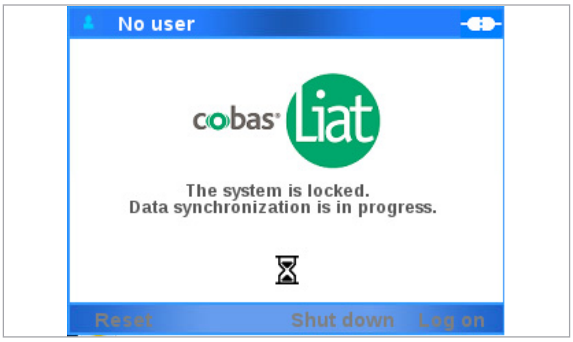

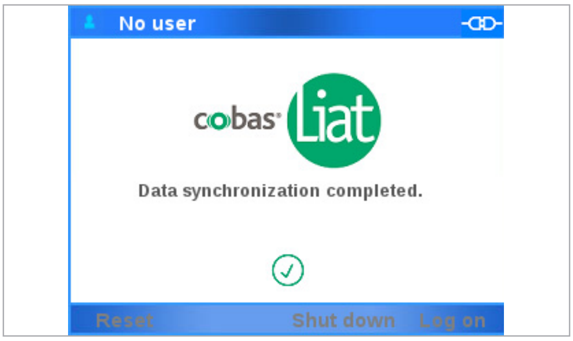


```
<HEL.R01>
    <HDR>
        <HDR.control_id V="401"/>
        <HDR.version_id V="POCT1"/>
        <HDR.creation_dttm V="2020-01-15T09:31:11-05:00"/>
    </HDR>
    <DEV>
        <DEV.device_id V="f8:dc:7a:1c:a3:c9"/>
        <DEV.vendor_id V="ROCHE"/>
        <DEV.serial_id V="M1-E-16036"/>
        <DEV.manufacturer_name V="Roche Molecular Diagnostics"/>
        <DEV.sw_version V="3.3.0.4027"/>
        <DEV.device_name V="cobasLiat"/>
        <DCP>
            <DCP.application_timeout V="30"/>
            <DCP.vendor_specific>
                DTV.ROCHE.LIAT.CFG
            </DCP.vendor_specific>
        </DCP>
        <DSC>
            <DSC.connection_profile_cd V="SA"/>
            <DSC.topics_supported_cd V="D_EV"/>
            <DSC.topics_supported_cd V="DTV"/>
            <DSC.max_message_sz V="614400"/>
        </DSC>
    </DEV>
</HEL.R01>
```

```
<ACK.R01>
    <HDR>
        <HDR.control_id V="2"/>
        <HDR.version_id V="POCT1"/>
        <HDR.creation_dttm V="2020-01-15T15:31:10+01:00"/>
    </HDR>
    <ACK>
        <ACK.type_cd V="AA"/>
        <ACK.ack_control_id V="401"/>
    </ACK>
</ACK.R01>
<DST.R01>
    <HDR>
        <HDR.control_id V="402"/>
        <HDR.version_id V="POCT1"/>
        <HDR.creation_dttm V="2020-01-15T09:31:11-05:00"/>
    </HDR>
    <DST>
        <DST.status_dttm V="2020-01-15T09:31:11-05:00"/>
        <DST.new_observations_qty V="0"/>
        <DST.new_events_qty V="15"/>
        <DST.condition_cd V="S"/>
    </DST>
</DST.R01>
<ACK.R01>
    <HDR>
        <HDR.control_id V="3"/>
        <HDR.version_id V="POCT1"/>
        <HDR.creation_dttm V="2020-01-15T15:31:11+01:00"/>
    </HDR>
    <ACK>
        <ACK.type_cd V="AA"/>
        <ACK.ack_control_id V="402"/>
    </ACK>
</ACK.R01>
<DTV.ROCHE.LIAT.CFG>
    <HDR>
        <HDR.message_type V="DTV.ROCHE.LIAT.CFG" SN="ROCHE" SV="1.0"/>
        <HDR.control_id V="4"/>
        <HDR.version_id V="POCT1"/>
        <HDR.creation_dttm V="2020-01-15T15:31:13+01:00"/>
    </HDR>
    <DTV>
        <DTV.command_cd V="SET_CONFIG" SN="ROCHE" SV="1.0"/>
    </DTV>
    <GEN_CFG>
        <GEN_CFG.DateTime.sntp V="false"/>
        <GEN_CFG.DateTime.Server V=""/>
        <GEN_CFG.DateTime.TimeZone V="Eastern Standard Time"/>
        <GEN_CFG.DateTime.TimeFormat V="12"/>
        <GEN_CFG.DateTime.DateFormat V="yyyy-mm-dd"/>
        <GEN_CFG.Display.brightness V="7"/>
        <GEN_CFG.EnableTilt.EnableCheckTilt V="true"/>
        <GEN_CFG.TubeInsertTime.iTubeInsertTime V="15"/>
        <GEN_CFG.AutoLock.autolocktime V="7"/>
        <GEN_CFG.Authentication.authenticationType V="User ID & Password"/>
        <GEN_CFG.Languages.Language V="en-US"/>
        <GEN_CFG.AutoReboot.Time V="05:30"/>
        <GEN_CFG.Connectivity.Timeout V="30"/>
```

```
        <GEN_CFG.Connectivity.DMLAutoSend V="true"/>
        <GEN_CFG.Connectivity.DataSynchronizationUsers V="false"/>
        <GEN_CFG.Connectivity.DataSynchronizationAssayLots V="true"/>
        <GEN_CFG.Connectivity.ConnectionInterval V="05"/>
     </GEN_CFG>
</DTV.ROCHE.LIAT.CFG>
<ACK.R01>
    <HDR>
        <HDR.control_id V="403"/>
        <HDR.version_id V="POCT1"/>
        <HDR.creation_dttm V="2020-01-15T09:31:14-05:00"/>
    </HDR>
    <ACK>
        <ACK.type_cd V="AA"/>
        <ACK.ack_control_id V="4"/>
        <ACK.note_txt/>
    </ACK>
</ACK.R01>
<END.R01>
    <HDR>
        <HDR.control_id V="404"/>
        <HDR.version_id V="POCT1"/>
        <HDR.creation_dttm V="2020-01-15T09:31:45-05:00"/>
    </HDR>
    <TRM>
        <TRM.reason_cd V="NRM"/>
        <TRM.note_txt V="There are no more commands to process."/>
    </TRM>
</END.R01>
<ACK.R01>
    <HDR>
        <HDR.control_id V="5"/>
        <HDR.version_id V="POCT1"/>
        <HDR.creation_dttm V="2020-01-15T15:31:44+01:00"/>
    </HDR>
    <ACK>
        <ACK.type_cd V="AA"/>
        <ACK.ack_control_id V="404"/>
    </ACK>
</ACK.R01>
```

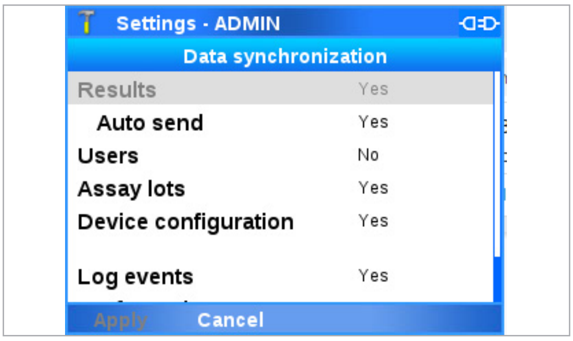

Communication scenario 5 – Send a validated assay lot to a DMS

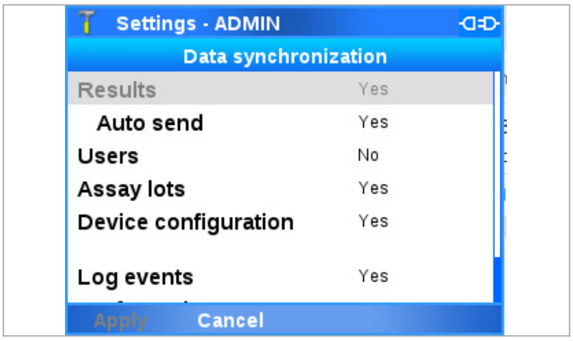

- **Purpose:** to see lot related exchanges, after a lot validation (negative and positive QC's) are performed on the analyzer.
- **Configuration/Preconditions:** Assay Menu with no Lots, Lots enabled, Events enabled, Autosend on (Note that config changed).

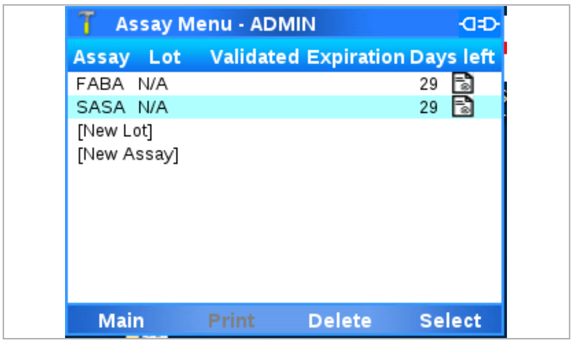

Steps

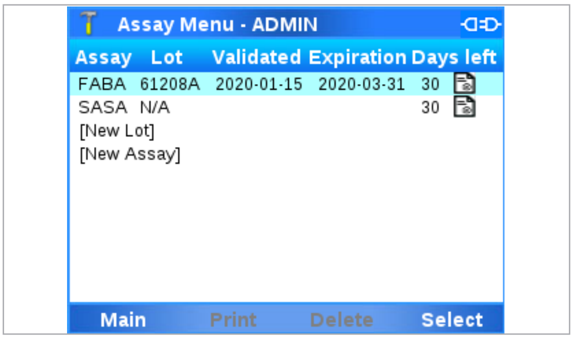

1. Liat: validate FABA Lot.
2. Liat: connect to DMS
3. DMS: request device events
4. Liat: send Events -> Lot added (TR.001)
5. DMS: request Lots
6. Liat: send FABA Lot
7. DMS: request OBS
8. Liat: send OBS (this message contains the QCs for FABA Lot)
9. EOT
10. END

Direction	Message type	Comment
Analyzer➔DMS	HEL.R01	
DMS➔analyzer	ACK.R01	
Analyzer➔DMS	DST.R01	Observe that 2 OBS (the positive and negative QC's) are reported.
DMS➔analyzer	ACK.R01	Handshake successful
DMS➔analyzer	REQ.R01	DMS requests device events.
Analyzer➔DMS	EVS.R01	Device event TR.001 informs the DMS to request lots.
DMS➔analyzer	ACK.R01	
Analyzer➔DMS	EOT.R01	
DMS➔analyzer	REQ.R01	DMS requests assay lots (RRDL_D).
Analyzer➔DMS	ROCHE.LIAT.LOTS.R02	Lots
DMS➔analyzer	ACK.R01	
Analyzer➔DMS	EOT.R01	
DMS➔analyzer	REQ.R01	DMS requests observations / results.
Analyzer➔DMS	OBS.R02	QC lot results (negative and positive)
DMS➔analyzer	ACK.R01	
Analyzer➔DMS	EOT.R01	
Analyzer➔DMS	END.R01	Liat: close connection
DMS➔analyzer	ACK.R01	DMS: connection closed


```
<HEL.R01>
    <HDR>
        <HDR.control_id V="331"/>
        <HDR.version_id V="POCT1"/>
        <HDR.creation_dttm V="2020-01-15T14:34:33-05:00"/>
    </HDR>
    <DEV>
        <DEV.device_id V="f8:dc:7a:1c:a3:c9"/>
        <DEV.vendor_id V="ROCHE"/>
        <DEV.serial_id V="M1-E-16036"/>
        <DEV.manufacturer_name V="Roche Molecular Diagnostics"/>
        <DEV.sw_version V="3.3.0.4027"/>
        <DEV.device_name V="cobasLiat"/>
        <DCP>
            <DCP.application_timeout V="30"/>
            <DCP.vendor_specific>
                ROCHE.LIAT.LOTS.R01;ROCHE.LIAT.LOTS.R02
            </DCP.vendor_specific>
        </DCP>
        <DSC>
            <DSC.connection_profile_cd V="SA"/>
            <DSC.topics_supported_cd V="D_EV"/>
            <DSC.max_message_sz V="614400"/>
        </DSC>
    </DEV>
</HEL.R01>
<ACK.R01>
    <HDR>
        <HDR.control_id V="1"/>
        <HDR.version_id V="POCT1"/>
        <HDR.creation_dttm V="2020-01-15T20:34:33+01:00"/>
    </HDR>
```

```
    <ACK>
        <ACK.type_cd V="AA"/>
        <ACK.ack_control_id V="331"/>
    </ACK>
</ACK.R01>
<DST.R01>
    <HDR>
        <HDR.control_id V="332"/>
        <HDR.version_id V="POCT1"/>
        <HDR.creation_dttm V="2020-01-15T14:34:34-05:00"/>
    </HDR>
    <DST>
        <DST.status_dttm V="2020-01-15T14:34:34-05:00"/>
        <DST.new_observations_qty V="2"/>
        <DST.new_events_qty V="5"/>
        <DST.condition_cd V="S"/>
    </DST>
</DST.R01>
<ACK.R01>
    <HDR>
        <HDR.control_id V="2"/>
        <HDR.version_id V="POCT1"/>
        <HDR.creation_dttm V="2020-01-15T20:34:34+01:00"/>
    </HDR>
    <ACK>
        <ACK.type_cd V="AA"/>
        <ACK.ack_control_id V="332"/>
    </ACK>
</ACK.R01>
<REQ.R01>
    <HDR>
        <HDR.control_id V="3"/>
        <HDR.version_id V="POCT1"/>
        <HDR.creation_dttm V="2020-01-15T20:34:43+01:00"/>
    </HDR>
    <REQ>
        <REQ.request_cd V="RDEV"/>
    </REQ>
</REQ.R01>
<EVS.R01>
    <HDR>
        <HDR.message_type V="EVS.R01"/>
        <HDR.control_id V="333"/>
        <HDR.version_id V="POCT1"/>
        <HDR.creation_dttm V="2020-01-15T14:34:45-05:00"/>
    </HDR>
    <EVT>
        <EVT.description V="AC.001:User [ADMIN] logged on with authentication mode [User ID &
Password]"/>
        <EVT.event_dttm V="2020-01-14T08:54:41-05:00"/>
        <EVT.severity_cd V="N"/>
        <OPR>
            <OPR.operator_id V="ADMIN"/>
        </OPR>
    <EVT>
        <EVT.description V="SC.013:'Host' settings changed by user [ADMIN]"/>
        <EVT.event_dttm V="2020-01-15T09:21:50-05:00"/>
        <EVT.severity_cd V="N"/>
        <OPR>
```

```
            <OPR.operator_id V="ADMIN"/>
        </OPR>
    </EVT>
   <EVT>
        <EVT.description V="TR.001:Trigger notification for lot data upload to DMS"/>
        <EVT.event_dttm V="2020-01-15T14:28:19-05:00"/>
        <EVT.severity_cd V="N"/>
        <OPR>
            <OPR.operator_id V="System"/>
        </OPR>
    </EVT>
    <EVT>
        <EVT.description V="AM.001:Lot(s) [FABA^61208A^1.31] validated by user [ADMIN]"/>
        <EVT.event_dttm V="2020-01-15T14:28:21-05:00"/>
        <EVT.severity_cd V="N"/>
        <OPR>
            <OPR.operator_id V="ADMIN"/>
        </OPR>
    </EVT>
    <EVT>
        <EVT.description V="AC.002:User [ADMIN] logged off"/>
        <EVT.event_dttm V="2020-01-15T14:31:15-05:00"/>
        <EVT.severity_cd V="N"/>
        <OPR>
            <OPR.operator_id V="ADMIN"/>
        </OPR>
    </EVT>
</EVS.R01>
<ACK.R01>
    <HDR>
        <HDR.control_id V="4"/>
        <HDR.version_id V="POCT1"/>
        <HDR.creation_dttm V="2020-01-15T20:34:45+01:00"/>
    </HDR>
    <ACK>
        <ACK.type_cd V="AA"/>
        <ACK.ack_control_id V="333"/>
    </ACK>
</ACK.R01>
<EOT.R01>
    <HDR>
        <HDR.control_id V="334"/>
        <HDR.version_id V="POCT1"/>
        <HDR.creation_dttm V="2020-01-15T14:34:45-05:00"/>
    </HDR>
    <EOT>
        <EOT.topic_cd V="EVS"/>
    </EOT>
</EOT.R01>
<REQ.R01>
    <HDR>
        <HDR.control_id V="5"/>
        <HDR.version_id V="POCT1"/>
        <HDR.creation_dttm V="2020-01-15T20:34:58+01:00"/>
    </HDR>
    <REQ>
        <REQ.request_cd V="RRDL_D"/>
    </REQ>
</REQ.R01>
```

```
<ROCHE.LIAT.LOTS.R02>
    <HDR>
        <HDR.message_type V="ROCHE.LIAT.LOTS.R02" SN="ROCHE" SV="1.0"/>
        <HDR.control_id V="335"/>
        <HDR.version_id V="POCT1"/>
        <HDR.creation_dttm V="2020-01-15T14:35:00-05:00"/>
    </HDR>
<UPD>
    <UPD.action_cd V=”I”/>
    <LOT>
        <LOT.lot_id V="FABA^61208A^1.31"/>
        <LOT.lot_insert_id V="IFABA20036B8AAAAAAAAAAAAH"/>
        <LOT.parameters V="AAAAAAAAAAA"/>
        <LOT.assay V="FABA"/>
        <LOT.expiration_date V="2020-03-31T00:00:00+00:00"/>
        <LOT.lot_number V="61208A"/>
        <LOT.minimum_compatible_version V="1.31"/>
        <LOT.validation_dttm V="2020-01-15T14:28:16-05:00"/>
        <LOT.data ENC="B64"
V="ouWnn4r3ErOJHy22bmsdhLOq3welDVBzrHy6JptBeQ2BuBcFICnTZW49mQIlIypr1/0MpOodM131b13LKAVHxuYIClG1QJIHt
gBbS/LJP+UwTMMqh02zzqeYWvTAnU0DvG77jFtubEC3SLe9i8fsdxi3t4GSpIknhcqWQDFBtpSKlvYPzyYtxhVGMGr4RHonONCjP
7jaZR2SxeT5uf8THYuwxdJzSVGBTXkVea8d1paWOCjHBkanVfLo43JtHBOaH/E1CmBoUeZYsmoQvWQw/yWMAUGHH0Z4bCiYpiTXI
cPDoCHaphrJB25cHtL7TkvVVLojRXAii5l6R4t0C5GM6Q=="/>
    </LOT>
</UPD>
</ROCHE.LIAT.LOTS.R02>
<ACK.R01>
    <HDR>
        <HDR.control_id V="6"/>
        <HDR.version_id V="POCT1"/>
        <HDR.creation_dttm V="2020-01-15T20:34:59+01:00"/>
    </HDR>
    <ACK>
        <ACK.type_cd V="AA"/>
        <ACK.ack_control_id V="335"/>
    </ACK>
</ACK.R01>
<EOT.R01>
    <HDR>
        <HDR.control_id V="336"/>
        <HDR.version_id V="POCT1"/>
        <HDR.creation_dttm V="2020-01-15T14:35:00-05:00"/>
    </HDR>
    <EOT>
        <EOT.topic_cd V="ROCHE.LIAT.LOTS"/>
    </EOT>
</EOT.R01>
<REQ.R01>
    <HDR>
        <HDR.control_id V="7"/>
        <HDR.version_id V="POCT1"/>
        <HDR.creation_dttm V="2020-01-15T20:35:11+01:00"/>
    </HDR>
    <REQ>
        <REQ.request_cd V="ROBS"/>
    </REQ>
</REQ.R01>
<OBS.R02>
    <HDR>
```

```
    <HDR.message_type V="OBS.R02"/>
    <HDR.control_id V="337"/>
    <HDR.version_id V="POCT1"/>
    <HDR.creation_dttm V="2020-01-15T14:35:13-05:00"/>
</HDR>
<SVC>
    <SVC.role_cd V="LQC"/>
    <SVC.observation_dttm V="2020-01-15T14:27:16-05:00"/>
    <CTC>
        <CTC.name V="FABA control"/>
        <CTC.lot_number V="61208A"/>
        <CTC.expiration_date V="2020-03-31T00:00:00+00:00"/>
        <CTC.level_cd V="N" SN="ROCHE" SV="1.0"/>
        <OBS>
            <OBS.observation_id V="Influenza A (FABA)" SN="ROCHE" SV="1.0"/>
            <OBS.qualitative_value V="Not Detected" SN="ROCHE" SV="1.0"/>
            <OBS.method_cd V="M"/>
        </OBS>
        <OBS>
            <OBS.observation_id V="Influenza B (FABA)" SN="ROCHE" SV="1.0"/>
            <OBS.qualitative_value V="Not Detected" SN="ROCHE" SV="1.0"/>
            <OBS.method_cd V="M"/>
        </OBS>
    </CTC>
    <OPR>
        <OPR.operator_id V="ADMIN"/>
    </OPR>
    <RGT>
        <RGT.name V="FABA"/>
        <RGT.lot_number V="FABA^61208A^1.31"/>
        <RGT.expiration_date V="2020-03-31T00:00:00+00:00"/>
    </RGT>
    <NTE>
        <NTE.text V="LIAT.Use=For In Vitro Diagnostic Use"/>
    </NTE>
    <NTE>
        <NTE.text V="LIAT.Run=00009"/>
    </NTE>
    <NTE>
        <NTE.text V="LIAT.Tube=00003"/>
    </NTE>
    <NTE>
        <NTE.text V="LIAT.Tube_id=TFABA20036B8A00003U"/>
    </NTE>
    <NTE>
        <NTE.text V="LIAT.Approver=N/A"/>
    </NTE>
    <NTE>
        <NTE.text V="LIAT.Universal_service_id=Liat Influenza Assay"/>
    </NTE>
    <NTE>
        <NTE.text V="LIAT.Lot_validation_status=Incomplete"/>
    </NTE>
</SVC>
<SVC>
    <SVC.role_cd V="LQC"/>
    <SVC.observation_dttm V="2020-01-15T14:28:07-05:00"/>
    <CTC>
        <CTC.name V="FABA control"/>
```

```
            <CTC.lot_number V="61208A"/>
            <CTC.expiration_date V="2020-03-31T00:00:00+00:00"/>
            <CTC.level_cd V="L" SN="ROCHE" SV="1.0"/>
            <OBS>
                <OBS.observation_id V="Influenza A (FABA)" SN="ROCHE" SV="1.0"/>
                <OBS.qualitative_value V="Detected" SN="ROCHE" SV="1.0"/>
                <OBS.method_cd V="M"/>
            </OBS>
            <OBS>
                <OBS.observation_id V="Influenza B (FABA)" SN="ROCHE" SV="1.0"/>
                <OBS.qualitative_value V="Detected" SN="ROCHE" SV="1.0"/>
                <OBS.method_cd V="M"/>
            </OBS>
        </CTC>
        <OPR>
            <OPR.operator_id V="ADMIN"/>
        </OPR>
        <RGT>
            <RGT.name V="FABA"/>
            <RGT.lot_number V="FABA^61208A^1.31"/>
            <RGT.expiration_date V="2020-03-31T00:00:00+00:00"/>
        </RGT>
        <NTE>
            <NTE.text V="LIAT.Use=For In Vitro Diagnostic Use"/>
        </NTE>
        <NTE>
            <NTE.text V="LIAT.Run=00010"/>
        </NTE>
        <NTE>
            <NTE.text V="LIAT.Tube=00004"/>
        </NTE>
        <NTE>
            <NTE.text V="LIAT.Tube_id=TFABA20036B8A00004T"/>
        </NTE>
        <NTE>
            <NTE.text V="LIAT.Approver=N/A"/>
        </NTE>
        <NTE>
            <NTE.text V="LIAT.Universal_service_id=Liat Influenza Assay"/>
        </NTE>
        <NTE>
            <NTE.text V="LIAT.Lot_validation_status=Validated"/>
        </NTE>
    </SVC>
</OBS.R02>
<ACK.R01>
    <HDR>
        <HDR.control_id V="8"/>
        <HDR.version_id V="POCT1"/>
        <HDR.creation_dttm V="2020-01-15T20:35:12+01:00"/>
    </HDR>
    <ACK>
        <ACK.type_cd V="AA"/>
        <ACK.ack_control_id V="337"/>
    </ACK>
</ACK.R01>
<EOT.R01>
    <HDR>
        <HDR.control_id V="338"/>
```

```
        <HDR.version_id V="POCT1"/>
        <HDR.creation_dttm V="2020-01-15T14:35:13-05:00"/>
    </HDR>
    <EOT>
        <EOT.topic_cd V="OBS"/>
    </EOT>
</EOT.R01>
<END.R01>
    <HDR>
        <HDR.control_id V="339"/>
        <HDR.version_id V="POCT1"/>
        <HDR.creation_dttm V="2020-01-15T14:35:43-05:00"/>
    </HDR>
    <TRM>
        <TRM.reason_cd V="ABN"/>
        <TRM.note_txt V="Timeout occurred."/>
    </TRM>
</END.R01>
<ACK.R01>
    <HDR>
        <HDR.control_id V="9"/>
        <HDR.version_id V="POCT1"/>
        <HDR.creation_dttm V="2020-01-15T20:35:43+01:00"/>
    </HDR>
    <ACK>
        <ACK.type_cd V="AA"/>
        <ACK.ack_control_id V="339"/>
    </ACK>
</ACK.R01>
```

Communication scenario 6 – Send a validated assay lot from DMS to an instrument

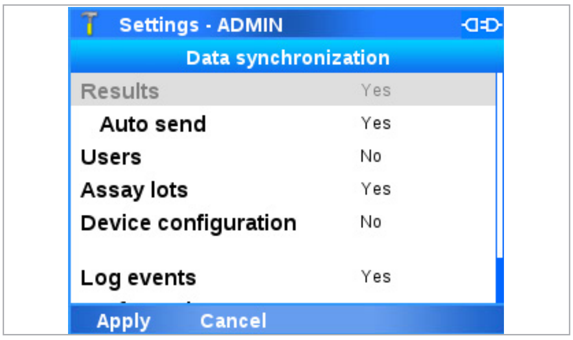

- **Purpose:** to see how the Liat receives/accepts lots from the DMS.
- **Configuration/Preconditions:** Assay menu with FABA lot, Lots enabled, Events enabled, and Autosend on.

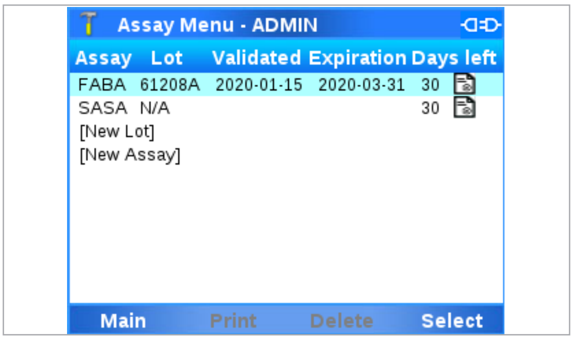

Steps

1. (Info) SASA Lot was added on another instrument and was already successfully sent to the DMS.
2. DMS: send SASA lot.
3. Liat: ACK

Direction	Message type	Comment
Analyzer→DMS	HEL.R01	
DMS→analyzer	ACK.R01	
Analyzer→DMS	DST.R01	
DMS→analyzer	ACK.R01	Handshake successful
DMS→analyzer	ROCHE.LIAT.LOTS.R02	DMS sends SASA lot.
Analyzer→DMS	ACK.R01	Liat accepts SASA lot.
DMS→analyzer	EOT.R01	End of Topic (Lot transmission)
Analyzer→DMS	END.R01	Liat: close connection
DMS→analyzer	ACK.R01	DMS: connection closed


```
<HEL.R01>
    <HDR>
        <HDR.control_id V="350"/>
        <HDR.version_id V="POCT1"/>
        <HDR.creation_dttm V="2020-01-15T15:04:33-05:00"/>
    </HDR>
    <DEV>
        <DEV.device_id V="f8:dc:7a:1c:a3:c9"/>
        <DEV.vendor_id V="ROCHE"/>
        <DEV.serial_id V="M1-E-16036"/>
        <DEV.manufacturer_name V="Roche Molecular Diagnostics"/>
        <DEV.sw_version V="3.3.0.4027"/>
        <DEV.device_name V="cobasLiat"/>
        <DCP>
            <DCP.application_timeout V="30"/>
            <DCP.vendor_specific>
                ROCHE.LIAT.LOTS.R01;ROCHE.LIAT.LOTS.R02
            </DCP.vendor_specific>
        </DCP>
        <DSC>
            <DSC.connection_profile_cd V="SA"/>
            <DSC.topics_supported_cd V="D_EV"/>
            <DSC.max_message_sz V="614400"/>
        </DSC>
    </DEV>
</HEL.R01>
<ACK.R01>
```

```
    <HDR>
        <HDR.control_id V="2"/>
        <HDR.version_id V="POCT1"/>
        <HDR.creation_dttm V="2020-01-15T21:04:33+01:00"/>
    </HDR>
    <ACK>
        <ACK.type_cd V="AA"/>
        <ACK.ack_control_id V="350"/>
    </ACK>
</ACK.R01>
<DST.R01>
    <HDR>
        <HDR.control_id V="351"/>
        <HDR.version_id V="POCT1"/>
        <HDR.creation_dttm V="2020-01-15T15:04:34-05:00"/>
    </HDR>
    <DST>
        <DST.status_dttm V="2020-01-15T15:04:34-05:00"/>
        <DST.new_observations_qty V="0"/>
        <DST.new_events_qty V="2"/>
        <DST.condition_cd V="S"/>
    </DST>
</DST.R01>
<ACK.R01>
    <HDR>
        <HDR.control_id V="3"/>
        <HDR.version_id V="POCT1"/>
        <HDR.creation_dttm V="2020-01-15T21:04:34+01:00"/>
    </HDR>
    <ACK>
        <ACK.type_cd V="AA"/>
        <ACK.ack_control_id V="351"/>
    </ACK>
</ACK.R01>
<ROCHE.LIAT.LOTS.R02>
    <HDR>
        <HDR.message_type V="ROCHE.LIAT.LOTS.R02" SN="ROCHE" SV="1.0"/>
        <HDR.control_id V="4"/>
        <HDR.version_id V="POCT1"/>
        <HDR.creation_dttm V="2020-01-15T21:04:36+01:00"/>
    </HDR>
    <UPD>
        <UPD.action_cd V="I"/>
        <LOT>
            <LOT.lot_id V="SASA^A56B^1.26"/>
            <LOT.lot_insert_id V="ISASA3412A56BAAAAAAAAAAAT"/>
            <LOT.parameters V="AAAAAAAAAAA"/>
            <LOT.assay V="SASA"/>
            <LOT.expiration_date V="2034-12-31T00:00:00+00:00"/>
            <LOT.lot_number V="A56B"/>
            <LOT.minimum_compatible_version V="1.26"/>
            <LOT.validation_dttm V="2018-01-01T00:00:00+00:00"/>
            <LOT.data
V="ORO2TpIanrlO4iOB3SYH/jIrzI7XXaqNcB0xQjalVBZ3GGp2GoxFPNfEYS2g37keDruTNymT6v3HuJo2VnZhDf5hSxNj8/VSo
GLhvBOaxmLqbrVpPC7EGAZO4NGLLrTLBYIX2OjQg/dC4wiRqnUVIIxDQoVbIdJKCorngVxrkl+IMEIZ+8gxSeLzNpbM7wlX8HTJW
niqFxMcEkwp6hJxAt29jTNxlItyt97i6FpEgl495ECV4m+zxR5o/sPXd2lSMzZUYHEPLLmMkyoGfRuxohgcEjBDPSWSni45Da3oD
MZ5cTW82etpyK4M7BqR9/4K9Kzpeo4vkOoK4HtCVLZ44A==" ENC="B64"/>
        </LOT>
    </UPD>
```

```
</ROCHE.LIAT.LOTS.R02>
<ACK.R01>
    <HDR>
        <HDR.control_id V="352"/>
        <HDR.version_id V="POCT1"/>
        <HDR.creation_dttm V="2020-01-15T15:04:37-05:00"/>
    </HDR>
    <ACK>
        <ACK.type_cd V="AA"/>
        <ACK.ack_control_id V="4"/>
        <ACK.note_txt/>
    </ACK>
</ACK.R01>
<EOT.R01>
    <HDR>
        <HDR.control_id V="5"/>
        <HDR.version_id V="POCT1"/>
        <HDR.creation_dttm V="2020-01-27T08:27:23-05:00"/>
    </HDR>
    <EOT>
        <EOT.topic_cd V="ROCHE.LIAT.LOTS"/>
    </EOT>
</EOT.R01>
<END.R01>
    <HDR>
        <HDR.control_id V="354"/>
        <HDR.version_id V="POCT1"/>
        <HDR.creation_dttm V="2020-01-15T15:05:08-05:00"/>
    </HDR>
    <TRM>
        <TRM.reason_cd V="NRM"/>
        <TRM.note_txt V="Conversation was terminated by the user."/>
    </TRM>
</END.R01>
<ACK.R01>
 <HDR>
        <HDR.control_id V="6"/>
        <HDR.version_id V="POCT1"/>
        <HDR.creation_dttm V="2020-01-15T21:05:09+01:00"/>
    </HDR>
    <ACK>
        <ACK.type_cd V="AA"/>
        <ACK.ack_control_id V="354"/>
    </ACK>
</ACK.R01>
```

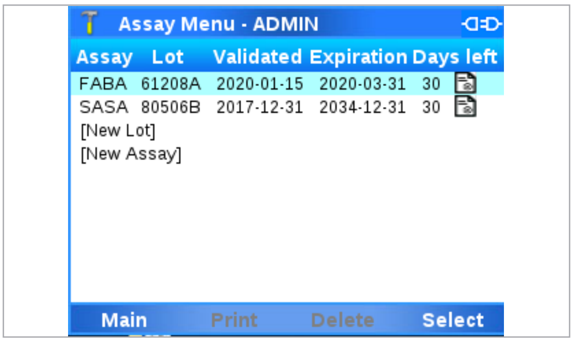

Communication scenario 7 – Liat sends automatically a result to a DMS

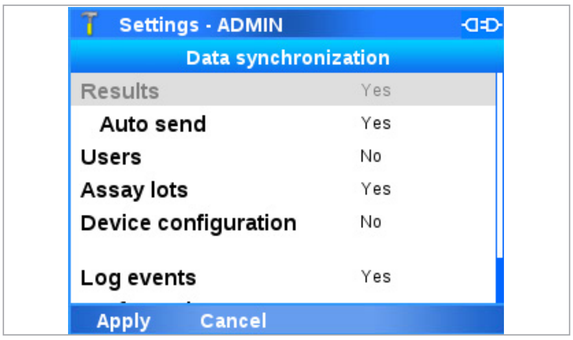

- **Purpose:** to see how the Liat sends observations.
- **Configuration/Preconditions:** Assay menu with FABA & SASA Lots, Lots enabled, Events enabled, Autosend on, and PV disabled.

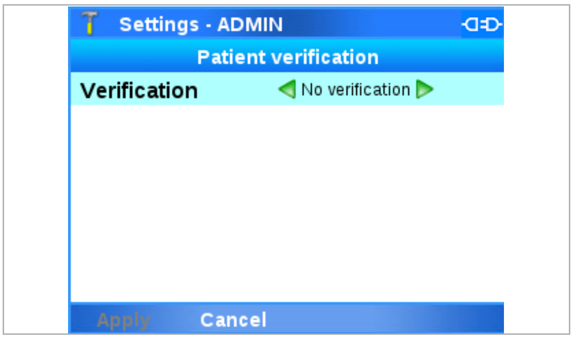

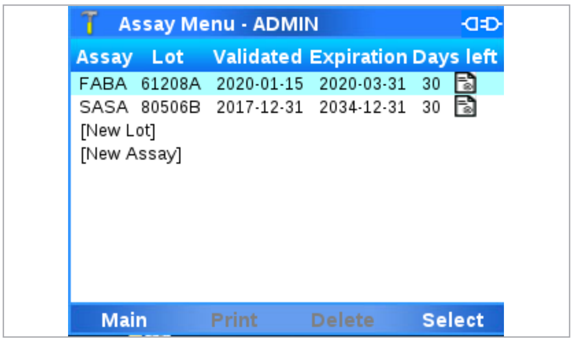

Steps

1. Liat: perform a SASA run, for patient with sample ID 12345 (this is result I).
2. Liat: send result I.
3. DMS: ACK

Direction	Message type	Comment
Analyzer➔DMS	HEL.R01	
DMS➔analyzer	ACK.R01	
Analyzer➔DMS	DST.R01	
DMS➔analyzer	ACK.R01	Handshake successful
DMS➔analyzer	REQ.R01	DMS requests observations / results
Analyzer➔DMS	OBS.R01	
DMS➔analyzer	ACK.R01	
Analyzer➔DMS	EOT.R01	
Analyzer➔DMS	END.R01	Liat: connection closed
DMS➔analyzer	ACK.R01	DMS: connection closed


```
<HEL.R01>
    <HDR>
        <HDR.control_id V="365"/>
        <HDR.version_id V="POCT1"/>
        <HDR.creation_dttm V="2020-01-15T15:16:24-05:00"/>
    </HDR>
    <DEV>
        <DEV.device_id V="f8:dc:7a:1c:a3:c9"/>
        <DEV.vendor_id V="ROCHE"/>
        <DEV.serial_id V="M1-E-16036"/>
        <DEV.manufacturer_name V="Roche Molecular Diagnostics"/>
        <DEV.sw_version V="3.3.0.4027"/>
        <DEV.device_name V="cobasLiat"/>
        <DCP>
            <DCP.application_timeout V="30"/>
            <DCP.vendor_specific>
                ROCHE.LIAT.LOTS.R01;ROCHE.LIAT.LOTS.R02
            </DCP.vendor_specific>
        </DCP>
        <DSC>
            <DSC.connection_profile_cd V="SA"/>
            <DSC.topics_supported_cd V="D_EV"/>
            <DSC.max_message_sz V="614400"/>
        </DSC>
    </DEV>
</HEL.R01>
<ACK.R01>
    <HDR>
        <HDR.control_id V="2"/>
        <HDR.version_id V="POCT1"/>
        <HDR.creation_dttm V="2020-01-15T21:16:24+01:00"/>
    </HDR>
    <ACK>
        <ACK.type_cd V="AA"/>
        <ACK.ack_control_id V="365"/>
    </ACK>
</ACK.R01>
<DST.R01>
    <HDR>
        <HDR.control_id V="366"/>
        <HDR.version_id V="POCT1"/>
        <HDR.creation_dttm V="2020-01-15T15:16:25-05:00"/>
```

```
    </HDR>
    <DST>
        <DST.status_dttm V="2020-01-15T15:16:25-05:00"/>
        <DST.new_observations_qty V="1"/>
        <DST.new_events_qty V="10"/>
        <DST.condition_cd V="S"/>
    </DST>
</DST.R01>
<ACK.R01>
    <HDR>
        <HDR.control_id V="3"/>
        <HDR.version_id V="POCT1"/>
        <HDR.creation_dttm V="2020-01-15T21:16:25+01:00"/>
    </HDR>
    <ACK>
        <ACK.type_cd V="AA"/>
        <ACK.ack_control_id V="366"/>
    </ACK>
</ACK.R01>
<REQ.R01>
    <HDR>
        <HDR.control_id V="4"/>
        <HDR.version_id V="POCT1"/>
        <HDR.creation_dttm V="2020-01-15T21:16:35+01:00"/>
    </HDR>
    <REQ>
        <REQ.request_cd V="ROBS"/>
    </REQ>
</REQ.R01>
<OBS.R01>
    <HDR>
        <HDR.message_type V="OBS.R01"/>
        <HDR.control_id V="367"/>
        <HDR.version_id V="POCT1"/>
        <HDR.creation_dttm V="2020-01-15T15:16:36-05:00"/>
    </HDR>
    <SVC>
        <SVC.role_cd V="OBS"/>
        <SVC.observation_dttm V="2020-01-15T15:10:53-05:00"/>
        <PT>
            <PT.patient_id V="12345"/>
            <OBS>
                <OBS.observation_id V="Strep A (SASA)" SN="ROCHE" SV="1.0"/>
                <OBS.qualitative_value V="Detected" SN="ROCHE" SV="1.0"/>
                <OBS.method_cd V="M"/>
            </OBS>
        </PT>
        <OPR>
            <OPR.operator_id V="ADMIN"/>
        </OPR>
        <ORD>
            <ORD.universal_service_id V="Strep A Assay" SN="ROCHE" SV="1.0"/>
        </ORD>
        <RGT>
            <RGT.name V="SASA"/>
            <RGT.lot_number V="SASA^A56B^1.26"/>
            <RGT.expiration_date V="2034-12-31T00:00:00+00:00"/>
        </RGT>
        <NTE>
```

```
            <NTE.text V="LIAT.Use=For In Vitro Diagnostic Use"/>
        </NTE>
        <NTE>
            <NTE.text V="LIAT.Run=00011"/>
        </NTE>
        <NTE>
            <NTE.text V="LIAT.Tube=00005"/>
        </NTE>
        <NTE>
            <NTE.text V="LIAT.Tube_id=TSASA3412A56B00005E"/>
        </NTE>
        <NTE>
            <NTE.text V="LIAT.Approver=N/A"/>
        </NTE>
        <NTE>
            <NTE.text V="LIAT.Universal_service_id=Liat Strep A Assay"/>
        </NTE>
    </SVC>
</OBS.R01>
<ACK.R01>
    <HDR>
        <HDR.control_id V="5"/>
        <HDR.version_id V="POCT1"/>
        <HDR.creation_dttm V="2020-01-15T21:16:36+01:00"/>
    </HDR>
    <ACK>
        <ACK.type_cd V="AA"/>
        <ACK.ack_control_id V="367"/>
    </ACK>
</ACK.R01>
<EOT.R01>
    <HDR>
        <HDR.control_id V="368"/>
        <HDR.version_id V="POCT1"/>
        <HDR.creation_dttm V="2020-01-15T15:16:38-05:00"/>
    </HDR>
    <EOT>
        <EOT.topic_cd V="OBS"/>
    </EOT>
</EOT.R01>
<END.R01>
    <HDR>
        <HDR.control_id V="369"/>
        <HDR.version_id V="POCT1"/>
        <HDR.creation_dttm V="2020-01-15T15:17:08-05:00"/>
    </HDR>
    <TRM>
        <TRM.reason_cd V="NRM"/>
        <TRM.note_txt V="Conversation was terminated by the user."/>
    </TRM>
</END.R01>
<ACK.R01>
    <HDR>
        <HDR.control_id V="6"/>
        <HDR.version_id V="POCT1"/>
        <HDR.creation_dttm V="2020-01-15T21:17:08+01:00"/>
    </HDR>
    <ACK>
        <ACK.type_cd V="AA"/>
```

```
        <ACK.ack_control_id V="369"/>
    </ACK>
</ACK.R01>
```

Communication scenario 8 – Activate the patient verification workflow via DMS

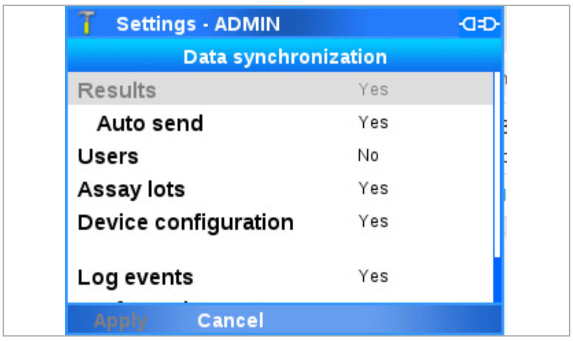

- **Purpose:** to see how the DMS enables the patient verification workflow on a Liat.
- **Configuration/Preconditions:** Assay menu with FABA & SASA Lots, Lots enabled, Events enabled, Autosend on, PV disabled, and Result I is visible.

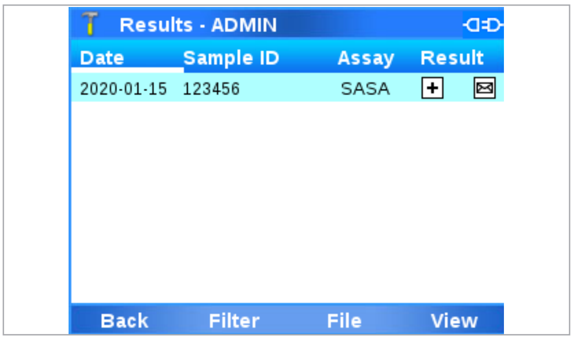

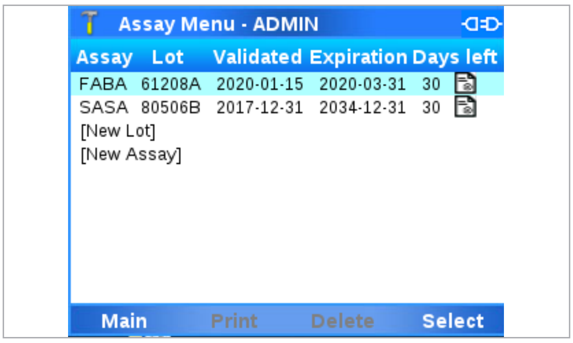

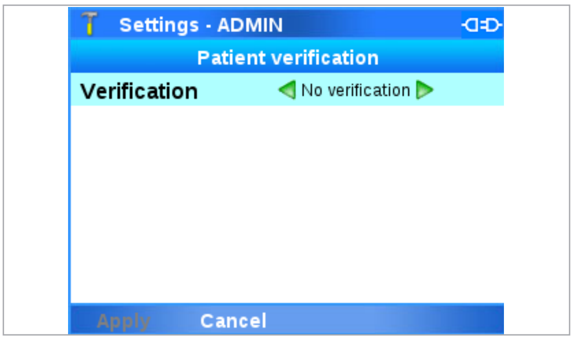

Steps

1. DMS: send DevConf with PV enabled.
2. Liat: ACK

Direction	Message type	Comment
Analyzer➔DMS	HEL.R01	
DMS➔analyzer	ACK.R01	
Analyzer➔DMS	DST.R01	
DMS➔analyzer	ACK.R01	Handshake successful
DMS➔analyzer	DTV.ROCHE.LIAT.CFG	DMS sends DevConf.
Analyzer➔DMS	ACK.R01	Liat accepts DevConf. -> data sync.
Analyzer➔DMS	END.R01	Liat: connection closed
DMS➔analyzer	ACK.R01	DMS: connection closed

```
<HEL.R01>
    <HDR>
        <HDR.control_id V="225"/>
        <HDR.version_id V="POCT1"/>
        <HDR.creation_dttm V="2020-01-15T13:43:17-05:00"/>
    </HDR>
    <DEV>
```

```
        <DEV.device_id V="f8:dc:7a:1c:a3:c9"/>
        <DEV.vendor_id V="ROCHE"/>
        <DEV.serial_id V="M1-E-16036"/>
        <DEV.manufacturer_name V="Roche Molecular Diagnostics"/>
        <DEV.sw_version V="3.3.0.4027"/>
        <DEV.device_name V="cobasLiat"/>
        <DCP>
            <DCP.application_timeout V="30"/>
            <DCP.vendor_specific>
                ROCHE.LIAT.LOTS.R01;ROCHE.LIAT.LOTS.R02;DTV.ROCHE.LIAT.CFG
            </DCP.vendor_specific>
        </DCP>
        <DSC>
            <DSC.connection_profile_cd V="SA"/>
            <DSC.topics_supported_cd V="D_EV"/>
            <DSC.topics_supported_cd V="DTV"/>
            <DSC.max_message_sz V="614400"/>
        </DSC>
    </DEV>
</HEL.R01>
<ACK.R01>
    <HDR>
        <HDR.control_id V="5"/>
        <HDR.version_id V="POCT1"/>
        <HDR.creation_dttm V="2020-01-15T19:43:17+01:00"/>
    </HDR>
    <ACK>
        <ACK.type_cd V="AA"/>
        <ACK.ack_control_id V="225"/>
    </ACK>
</ACK.R01>
<DST.R01>
    <HDR>
        <HDR.control_id V="226"/>
        <HDR.version_id V="POCT1"/>
        <HDR.creation_dttm V="2020-01-15T13:43:18-05:00"/>
    </HDR>
    <DST>
        <DST.status_dttm V="2020-01-15T13:43:18-05:00"/>
        <DST.new_observations_qty V="0"/>
        <DST.new_events_qty V="71"/>
        <DST.condition_cd V="S"/>
    </DST>
</DST.R01>
<ACK.R01>
    <HDR>
        <HDR.control_id V="6"/>
        <HDR.version_id V="POCT1"/>
        <HDR.creation_dttm V="2020-01-15T19:43:18+01:00"/>
    </HDR>
    <ACK>
        <ACK.type_cd V="AA"/>
        <ACK.ack_control_id V="226"/>
    </ACK>
</ACK.R01>
<DTV.ROCHE.LIAT.CFG>
    <HDR>
        <HDR.message_type V="DTV.ROCHE.LIAT.CFG" SN="ROCHE" SV="1.0"/>
        <HDR.control_id V="7"/>
```

```
        <HDR.version_id V="POCT1"/>
        <HDR.creation_dttm V="2020-01-15T19:43:20+01:00"/>
    </HDR>
    <DTV>
        <DTV.command_cd V="SET_CONFIG" SN="ROCHE" SV="1.0"/>
    </DTV>
    <GEN_CFG>
        <GEN_CFG.PV.Verification V="prior run"/>
        <GEN_CFG.PV.VerificationType V="s"/>
        <GEN_CFG.PV.PatientMismatch V="run allowed"/>
        <GEN_CFG.PV.DisplayedData V="verbose"/>
        <GEN_CFG.PV.ManualConfirmation V="not required"/>
    </GEN_CFG>
</DTV.ROCHE.LIAT.CFG>
<ACK.R01>
    <HDR>
        <HDR.control_id V="227"/>
        <HDR.version_id V="POCT1"/>
        <HDR.creation_dttm V="2020-01-15T13:43:23-05:00"/>
    </HDR>
    <ACK>
        <ACK.type_cd V="AA"/>
        <ACK.ack_control_id V="7"/>
        <ACK.note_txt/>
    </ACK>
</ACK.R01>
<END.R01>
    <HDR>
        <HDR.control_id V="228"/>
        <HDR.version_id V="POCT1"/>
        <HDR.creation_dttm V="2020-01-15T13:43:54-05:00"/>
    </HDR>
    <TRM>
        <TRM.reason_cd V="NRM"/>
        <TRM.note_txt V="There are no more commands to process."/>
    </TRM>
</END.R01>
<ACK.R01>
    <HDR>
        <HDR.control_id V="8"/>
        <HDR.version_id V="POCT1"/>
        <HDR.creation_dttm V="2020-01-15T19:43:54+01:00"/>
    </HDR>
    <ACK>
        <ACK.type_cd V="AA"/>
        <ACK.ack_control_id V="228"/>
    </ACK>
</ACK.R01>
```

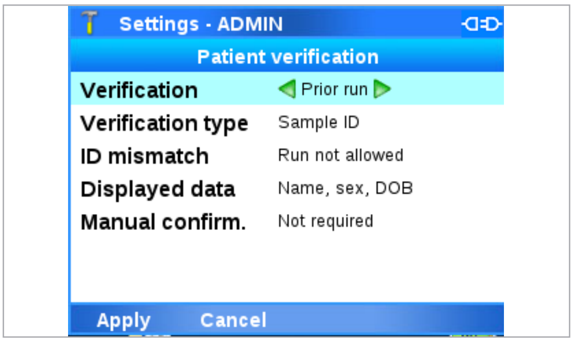

Communication scenario 9 – Patient verification passed – run performed

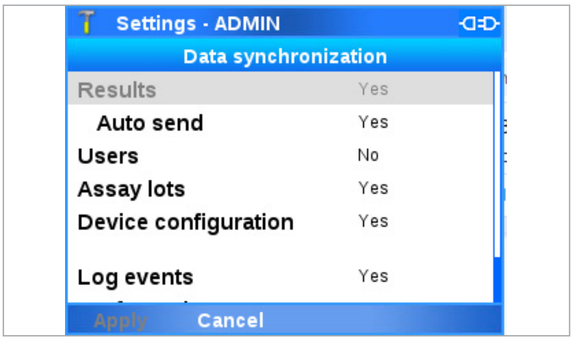

- **Purpose:** to see how during a run, a Liat performs a patient verification (by sample ID) workflow.
- **Configuration/Preconditions:** Assay Menu with FABA & SASA Lots, Lots enabled, Events enabled, Autosend on, PV enabled, and Result I is visible.

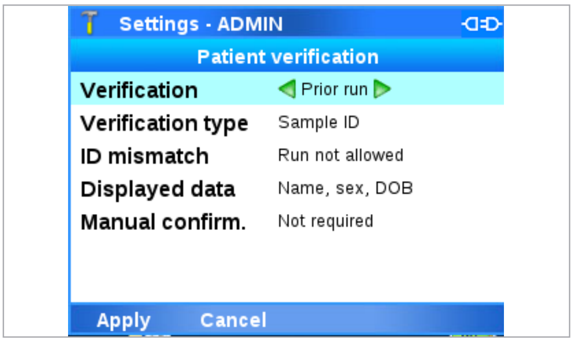

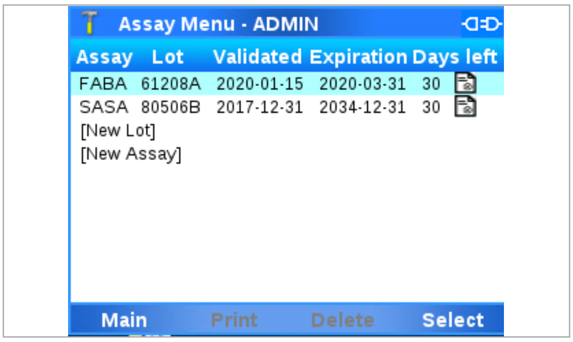

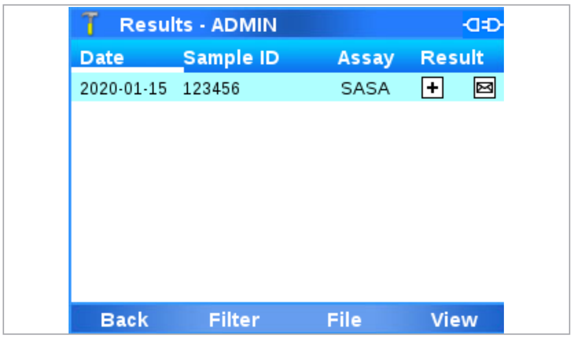

Steps

1. Liat: start a run using FABA, for patient with sample ID 67890 (result II).
2. Liat: send HEL (notice that it reports patient verification support)
3. Liat: PV workflow starts -> ID 67890 is known (DMS side) -> DMS: sends the patient data (Peter, 1960-08-29, ‘M’).

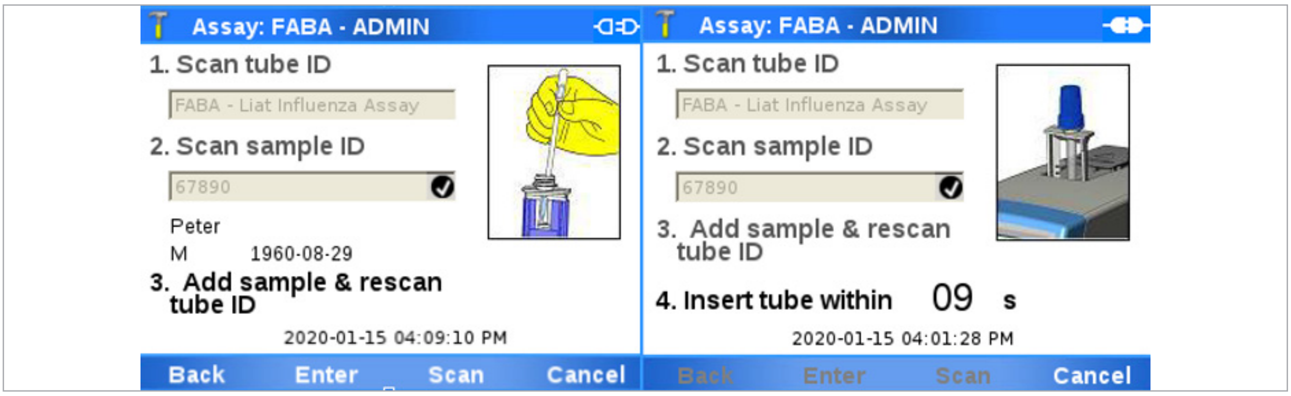

4. Liat: sends END. connection is closed.
5. Liat: run finishes.

6. Liat: wait for the next Liat - DMS connection
7. Liat: send result (II)

Direction	Message type	Comment
Analyzer→DMS	HEL.R01	Reports that patient verification is supported.
DMS→analyzer	ACK.R01	
Analyzer→DMS	DST.R01	
DMS→analyzer	ACK.R01	Handshake successful
DMS→analyzer	REQ.R01	DMS requests to send sample ID for PV.
Analyzer→DMS	ROCHE.LIAT.PVI.R01	Liat sends sample ID for PV check.
DMS→analyzer	ACK.R01	
Analyzer→DMS	EOT.R01	
DMS→analyzer	ROCHE.LIAT.PVR.R01	DMS sends PV data (“Peter”)
Analyzer→DMS	ACK.R01	Liat: acknowledges patient verification.
DMS→analyzer	EOT.R01	
Analyzer→DMS	END.R01	Liat: close connection.
DMS→analyzer	ACK.R01	DMS: connection closed.
Analyzer→DMS	HEL.R01	
DMS→analyzer	ACK.R01	
Analyzer→DMS	DST.R01	
DMS→analyzer	ACK.R01	Handshake successful
DMS→analyzer	REQ.R01	DMS requests for observations / results.
Analyzer→DMS	OBS.R01	
DMS→analyzer	ACK.R01	
Analyzer→DMS	EOT.R01	
Analyzer→DMS	END.R01	Liat: close connection.
DMS→analyzer	ACK.R01	DMS: connection closed.


```
<HEL.R01>
    <HDR>
        <HDR.control_id V="328"/>
        <HDR.version_id V="POCT1"/>
        <HDR.creation_dttm V="2021-01-08T04:31:53-10:00"/>
    </HDR>
    <DEV>
        <DEV.device_id V="f8:dc:7a:03:3b:74"/>
        <DEV.vendor_id V="ROCHE"/>
        <DEV.serial_id V="M1-E-09967"/>
        <DEV.manufacturer_name V="Roche Molecular Diagnostics"/>
        <DEV.sw_version V="5.0.0.4056"/>
        <DEV.device_name V="cobasLiat"/>
        <DCP>
            <DCP.application_timeout V="120"/>
            <DCP.vendor_specific>ROCHE.LIAT.PVI;ROCHE.LIAT.PVR</DCP.vendor_specific>
        </DCP>
        <DSC>
            <DSC.connection_profile_cd V="SA"/>
            <DSC.topics_supported_cd V="ROCHE.LIAT.PVI"/>
            <DSC.topics_supported_cd V="ROCHE.LIAT.PVR"/>
            <DSC.max_message_sz V="612345"/>
```

```
        </DSC>
    </DEV>
</HEL.R01>
<ACK.R01>
    <HDR>
        <HDR.control_id V="2"/>
        <HDR.version_id V="POCT1"/>
        <HDR.creation_dttm V="2021-01-08T15:30:46+01:00"/>
    </HDR>
    <ACK>
        <ACK.type_cd V="AA"/>
        <ACK.ack_control_id V="328"/>
    </ACK>
</ACK.R01>
<DST.R01>
    <HDR>
        <HDR.control_id V="329"/>
        <HDR.version_id V="POCT1"/>
        <HDR.creation_dttm V="2021-01-08T04:31:55-10:00"/>
    </HDR>
    <DST>
        <DST.status_dttm V="2021-01-08T04:31:55-10:00"/>
        <DST.new_observations_qty V="0"/>
        <DST.new_events_qty V="3"/>
        <DST.condition_cd V="R"/>
    </DST>
</DST.R01>
<ACK.R01>
    <HDR>
        <HDR.control_id V="914"/>
        <HDR.version_id V="POCT1"/>
        <HDR.creation_dttm V="2021-01-08T15:30:47+01:00"/>
    </HDR>
    <ACK>
        <ACK.type_cd V="AA"/>
        <ACK.ack_control_id V="329"/>
    </ACK>
</ACK.R01>
<REQ.R01>
    <HDR>
        <HDR.control_id V="915"/>
        <HDR.version_id V="POCT1"/>
        <HDR.creation_dttm V="2021-01-08T15:30:47+01:00"/>
    </HDR>
    <REQ>
        <REQ.request_cd V="RPVI"/>
    </REQ>
</REQ.R01>
<ROCHE.LIAT.PVI.R01>
    <HDR>
        <HDR.control_id V="330"/>
        <HDR.version_id V="POCT1"/>
        <HDR.creation_dttm V="2021-01-08T04:31:57-10:00"/>
    </HDR>
    <PVI>
        <PVI.verification_type_cd V="S"/>
        <PVI.identifier_id V="PTN001""/>
    </PVI>
</ROCHE.LIAT.PVI.R01>
```

```
<ACK.R01>
    <HDR>
        <HDR.control_id V="916"/>
        <HDR.version_id V="POCT1"/>
        <HDR.creation_dttm V="2021-01-08T15:30:50+01:00"/>
    </HDR>
    <ACK>
        <ACK.type_cd V="AA"/>
        <ACK.ack_control_id V="330"/>
    </ACK>
</ACK.R01>
<EOT.R01>
    <HDR>
        <HDR.control_id V="331"/>
        <HDR.version_id V="POCT1"/>
        <HDR.creation_dttm V="2021-01-08T04:31:57-10:00"/>
    </HDR>
    <EOT>
        <EOT.topic_cd V="ROCHE.LIAT.PVI"/>
    </EOT>
</EOT.R01>
<ROCHE.LIAT.PVR.R01>
    <HDR>
        <HDR.message_type V="PVR.R01"/>
        <HDR.control_id V="917"/>
        <HDR.version_id V="POCT1"/>
        <HDR.creation_dttm V="2021-01-08T15:30:50+01:00"/>
    </HDR>
    <PVF.status_cd V="T"/>
    <PT>
        <PT.patient_id V="PTN001"/>
        <PT.name V="Hansj....rg Feldmann"/>
        <PT.birth_date V="1901-01-01"/>
        <PT.gender_cd V="M"/>
    </PT>
</ROCHE.LIAT.PVR.R01>
<ACK.R01>
    <HDR>
        <HDR.control_id V="332"/>
        <HDR.version_id V="POCT1"/>
        <HDR.creation_dttm V="2021-01-08T04:31:59-10:00"/>
    </HDR>
    <ACK>
        <ACK.type_cd V="AA"/>
        <ACK.ack_control_id V="917"/>
        <ACK.note_txt/>
    </ACK>
</ACK.R01>
<EOT.R01>
    <HDR>
        <HDR.control_id V="918"/>
        <HDR.version_id V="POCT1"/>
        <HDR.creation_dttm V="2021-01-08T15:30:51+01:00"/>
    </HDR>
    <EOT>
        <EOT.topic_cd V="RPVI"/>
    </EOT>
</EOT.R01>
<END.R01>
```

```
    <HDR>
        <HDR.control_id V="333"/>
        <HDR.version_id V="POCT1"/>
        <HDR.creation_dttm V="2021-01-08T04:32:01-10:00"/>
    </HDR>
    <TRM>
        <TRM.reason_cd V="NRM"/>
        <TRM.note_txt V="Conversation was terminated by the user."/>
    </TRM>
</END.R01>
<ACK.R01>
    <HDR>
        <HDR.control_id V="919"/>
        <HDR.version_id V="POCT1"/>
        <HDR.creation_dttm V="2021-01-08T15:30:53+01:00"/>
    </HDR>
    <ACK>
        <ACK.type_cd V="AA"/>
        <ACK.ack_control_id V="438"/>
    </ACK>
</ACK.R01>


<HEL.R01>
    <HDR>
        <HDR.control_id V="443"/>
        <HDR.version_id V="POCT1"/>
        <HDR.creation_dttm V="2020-01-15T16:12:52-05:00"/>
    </HDR>
    <DEV>
        <DEV.device_id V="f8:dc:7a:1c:a3:c9"/>
        <DEV.vendor_id V="ROCHE"/>
        <DEV.serial_id V="M1-E-16036"/>
        <DEV.manufacturer_name V="Roche Molecular Diagnostics"/>
        <DEV.sw_version V="3.3.0.4027"/>
        <DEV.device_name V="cobasLiat"/>
        <DCP>
            <DCP.application_timeout V="30"/>
            <DCP.vendor_specific>
                ROCHE.LIAT.LOTS.R01;ROCHE.LIAT.LOTS.R02
            </DCP.vendor_specific>
        </DCP>
        <DSC>
            <DSC.connection_profile_cd V="SA"/>
            <DSC.topics_supported_cd V="D_EV"/>
            <DSC.max_message_sz V="614400"/>
        </DSC>
    </DEV>
</HEL.R01>
<ACK.R01>
    <HDR>
        <HDR.control_id V="2"/>
        <HDR.version_id V="POCT1"/>
        <HDR.creation_dttm V="2020-01-15T22:12:52+01:00"/>
    </HDR>
    <ACK>
        <ACK.type_cd V="AA"/>
        <ACK.ack_control_id V="443"/>
    </ACK>
```

```
</ACK.R01>
<DST.R01>
    <HDR>
        <HDR.control_id V="444"/>
        <HDR.version_id V="POCT1"/>
        <HDR.creation_dttm V="2020-01-15T16:12:53-05:00"/>
    </HDR>
    <DST>
        <DST.status_dttm V="2020-01-15T16:12:53-05:00"/>
        <DST.new_observations_qty V="1"/>
        <DST.new_events_qty V="53"/>
        <DST.condition_cd V="R"/>
    </DST>
</DST.R01>
<ACK.R01>
    <HDR>
        <HDR.control_id V="3"/>
        <HDR.version_id V="POCT1"/>
        <HDR.creation_dttm V="2020-01-15T22:12:53+01:00"/>
    </HDR>
    <ACK>
        <ACK.type_cd V="AA"/>
        <ACK.ack_control_id V="444"/>
    </ACK>
</ACK.R01>
<REQ.R01>
    <HDR>
        <HDR.control_id V="4"/>
        <HDR.version_id V="POCT1"/>
        <HDR.creation_dttm V="2020-01-15T22:13:13+01:00"/>
    </HDR>
    <REQ>
        <REQ.request_cd V="ROBS"/>
    </REQ>
</REQ.R01>
<OBS.R01>
    <HDR>
        <HDR.message_type V="OBS.R01"/>
        <HDR.control_id V="445"/>
        <HDR.version_id V="POCT1"/>
        <HDR.creation_dttm V="2020-01-15T16:13:14-05:00"/>
    </HDR>
    <SVC>
        <SVC.role_cd V="OBS"/>
        <SVC.observation_dttm V="2020-01-15T16:01:37-05:00"/>
        <PT>
            <PT.patient_id V="777"/>
            <OBS>
                <OBS.observation_id V="Influenza A (FABA)" SN="ROCHE" SV="1.0"/>
                <OBS.qualitative_value V="Detected" SN="ROCHE" SV="1.0"/>
                <OBS.method_cd V="M"/>
            </OBS>
            <OBS>
                <OBS.observation_id V="Influenza B (FABA)" SN="ROCHE" SV="1.0"/>
                <OBS.qualitative_value V="Detected" SN="ROCHE" SV="1.0"/>
                <OBS.method_cd V="M"/>
            </OBS>
        </PT>
        <OPR>
```

```
            <OPR.operator_id V="ADMIN"/>
        </OPR>
        <ORD>
            <ORD.universal_service_id V="Influenza Assay" SN="ROCHE" SV="1.0"/>
        </ORD>
        <RGT>
            <RGT.name V="FABA"/>
            <RGT.lot_number V="FABA^61208A^1.31"/>
            <RGT.expiration_date V="2020-03-31T00:00:00+00:00"/>
        </RGT>
        <NTE>
            <NTE.text V="LIAT.Use=For In Vitro Diagnostic Use"/>
        </NTE>
        <NTE>
            <NTE.text V="LIAT.Run=00012"/>
        </NTE>
        <NTE>
            <NTE.text V="LIAT.Tube=00005"/>
        </NTE>
        <NTE>
            <NTE.text V="LIAT.Tube_id=TFABA20036B8A00005S"/>
        </NTE>
        <NTE>
            <NTE.text V="LIAT.Approver=N/A"/>
        </NTE>
        <NTE>
            <NTE.text V="LIAT.Universal_service_id=Liat Influenza Assay"/>
        </NTE>
       </SVC>
</OBS.R01>
<ACK.R01>
    <HDR>
        <HDR.control_id V="5"/>
        <HDR.version_id V="POCT1"/>
        <HDR.creation_dttm V="2020-01-15T22:13:14+01:00"/>
    </HDR>
    <ACK>
        <ACK.type_cd V="AA"/>
        <ACK.ack_control_id V="445"/>
    </ACK>
</ACK.R01>
<EOT.R01>
    <HDR>
        <HDR.control_id V="446"/>
        <HDR.version_id V="POCT1"/>
        <HDR.creation_dttm V="2020-01-15T16:13:15-05:00"/>
    </HDR>
    <EOT>
        <EOT.topic_cd V="OBS"/>
    </EOT>
</EOT.R01>
<END.R01>
    <HDR>
        <HDR.control_id V="447"/>
        <HDR.version_id V="POCT1"/>
        <HDR.creation_dttm V="2020-01-15T16:13:45-05:00"/>
    </HDR>
    <TRM>
        <TRM.reason_cd V="ABN"/>
```

```
        <TRM.note_txt V="Timeout occurred."/>
    </TRM>
</END.R01>
<ACK.R01>
    <HDR>
        <HDR.control_id V="6"/>
        <HDR.version_id V="POCT1"/>
        <HDR.creation_dttm V="2020-01-15T22:13:45+01:00"/>
    </HDR>
    <ACK>
        <ACK.type_cd V="AA"/>
        <ACK.ack_control_id V="447"/>
    </ACK>
</ACK.R01>
```

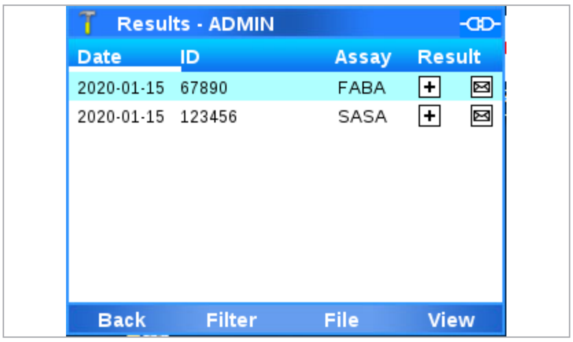

Communication scenario 10 – Patient verification failed – run prevented

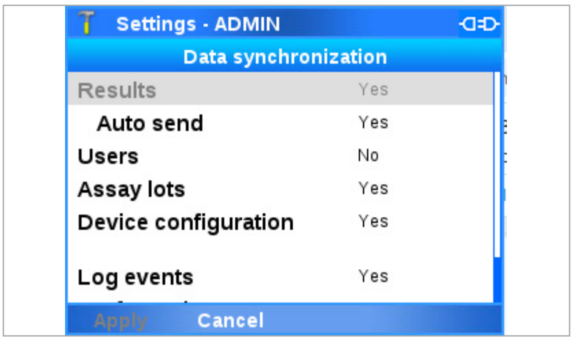

- **Purpose:** to show how a run is prevented due to a mismatch during Patient Verification workflow.
- **Configuration/Preconditions:** Assay menu with FABA & SASA Lots, Lots enabled, Events enabled, Autosend on, PV enabled but if mismatch, "run not allowed", and Result I and II are visible.

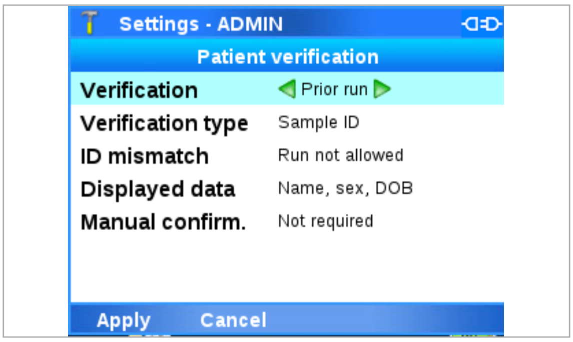

Steps

1. Liat: send HEL (highlight PV support).
2. Liat: start a run using FABA, for patient with sample ID 34567 (III).
3. Liat: PV starts, ID 34567 is unknown (DMS side).
4. DMS: send: "I could not find 34567".
5. Liat: conn closed.

Direction	Message type	Comment
Analyzer→DMS	HEL.R01	Reports that patient verification is supported.
DMS→analyzer	ACK.R01	
Analyzer→DMS	DST.R01	
DMS→analyzer	ACK.R01	Handshake successful
DMS→analyzer	REQ.R01	DMS: request for PV information
Analyzer→DMS	ROCHE.LIAT.PVI.R01	Liat: sends sample ID.
DMS→analyzer	ROCHE.LIAT.PVR.R01	DMS: sample ID unknown -> PV failed.
Analyzer→DMS	ACK.R01	Liat: acknowledges issue during patient verification. Run cannot be started.
Analyzer→DMS	END.R01	Liat: close connection.
DMS→analyzer	ACK.R01	DMS: connection closed.

```
<HEL.R01>
    <HDR>
        <HDR.control_id V="205"/>
        <HDR.version_id V="POCT1"/>
        <HDR.creation_dttm V="2020-01-16T10:39:35-05:00"/>
    </HDR>
    <DEV>
        <DEV.device_id V="f8:dc:7a:1c:a3:c9"/>
        <DEV.vendor_id V="ROCHE"/>
        <DEV.serial_id V="M1-E-16036"/>
        <DEV.manufacturer_name V="Roche Molecular Diagnostics"/>
        <DEV.sw_version V="3.3.0.4027"/>
        <DEV.device_name V="cobasLiat"/>
        <DCP>
            <DCP.application_timeout V="30"/>
            <DCP.vendor_specific>
                ROCHE.LIAT.PVI;ROCHE.LIAT.PVR
            </DCP.vendor_specific>
        </DCP>
        <DSC>
            <DSC.connection_profile_cd V="SA"/>
            <DSC.topics_supported_cd V="ROCHE.LIAT.PVI"/>
```

```
            <DSC.topics_supported_cd V="ROCHE.LIAT.PVR"/>
            <DSC.max_message_sz V="614400"/>
        </DSC>
    </DEV>
</HEL.R01>
<ACK.R01>
    <HDR>
        <HDR.control_id V="2"/>
        <HDR.version_id V="POCT1"/>
        <HDR.creation_dttm V="2020-01-16T16:39:35+01:00"/>
    </HDR>
    <ACK>
        <ACK.type_cd V="AA"/>
        <ACK.ack_control_id V="205"/>
    </ACK>
</ACK.R01>
<DST.R01>
    <HDR>
        <HDR.control_id V="206"/>
        <HDR.version_id V="POCT1"/>
        <HDR.creation_dttm V="2020-01-16T10:39:35-05:00"/>
    </HDR>
    <DST>
        <DST.status_dttm V="2020-01-16T10:39:35-05:00"/>
        <DST.new_observations_qty V="0"/>
        <DST.new_events_qty V="89"/>
        <DST.condition_cd V="R"/>
    </DST>
</DST.R01>
<ACK.R01>
    <HDR>
        <HDR.control_id V="3"/>
        <HDR.version_id V="POCT1"/>
        <HDR.creation_dttm V="2020-01-16T16:39:36+01:00"/>
    </HDR>
    <ACK>
        <ACK.type_cd V="AA"/>
        <ACK.ack_control_id V="206"/>
    </ACK>
</ACK.R01>
<REQ.R01>
    <HDR>
        <HDR.control_id V="4"/>
        <HDR.version_id V="POCT1"/>
        <HDR.creation_dttm V="2020-01-16T16:39:42+01:00"/>
    </HDR>
    <REQ>
        <REQ.request_cd V="RPVI"/>
    </REQ>
</REQ.R01>
<ROCHE.LIAT.PVI.R01>
    <HDR>
        <HDR.control_id V="207"/>
        <HDR.version_id V="POCT1"/>
        <HDR.creation_dttm V="2020-01-16T10:39:42-05:00"/>
    </HDR>
    <PVI>
        <PVI.verification_type_cd V="P"/>
        <PVI.identifier_id V="34567"/>
```

```
    </PVI>
</ROCHE.LIAT.PVI.R01>
<ROCHE.LIAT.PVR.R01>
    <HDR>
        <HDR.control_id V="5"/>
        <HDR.version_id V="POCT1"/>
        <HDR.creation_dttm V="2020-01-16T16:39:48+01:00"/>
    </HDR>
    <PVF.status_cd V="F"/>
</ROCHE.LIAT.PVR.R01>
<ACK.R01>
    <HDR>
        <HDR.control_id V="208"/>
        <HDR.version_id V="POCT1"/>
        <HDR.creation_dttm V="2020-01-16T10:39:48-05:00"/>
    </HDR>
    <ACK>
        <ACK.type_cd V="AA"/>
        <ACK.ack_control_id V="5"/>
        <ACK.note_txt/>
    </ACK>
</ACK.R01>
<END.R01>
    <HDR>
        <HDR.control_id V="209"/>
        <HDR.version_id V="POCT1"/>
        <HDR.creation_dttm V="2020-01-16T10:40:19-05:00"/>
    </HDR>
    <TRM>
        <TRM.reason_cd V="NRM"/>
        <TRM.note_txt V="Conversation was terminated by the user."/>
    </TRM>
</END.R01>
<ACK.R01>
    <HDR>
        <HDR.control_id V="6"/>
        <HDR.version_id V="POCT1"/>
        <HDR.creation_dttm V="2020-01-16T16:40:20+01:00"/>
    </HDR>
    <ACK>
        <ACK.type_cd V="AA"/>
        <ACK.ack_control_id V="209"/>
    </ACK>
</ACK.R01>
```


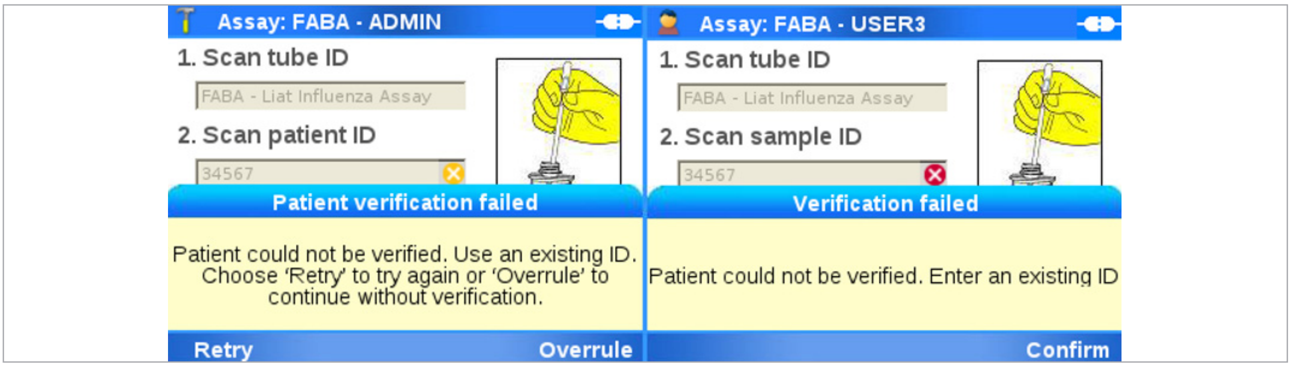

Note: whether you have the permission to overrule a failed patient verification depends on the analyzer configuration and your user role. To overrule a failed patient verification, you need a user role with a higher level of permission than the role “USER”.

- Left screenshot: the DMS protocol of Stage 10 shows the communication workflow for the user role “ADMIN” which permits overruling the failed patient verification.
- Right screenshot: example for the user role “USER” which does not permit overruling the failed patient verification.

Communication scenario 11 – Wrong instrument state does not accept a user list

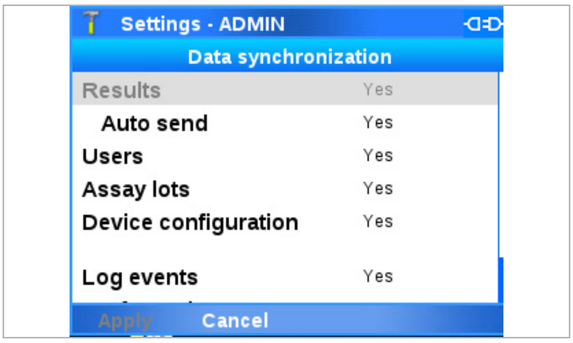

- **Purpose:** to see how the Liat does not accept (escapes) an Operator list, as it is not on “stand-by” state.
- **Configuration/Preconditions:** Assay menu with FABA & SASA Lots, Lots enabled, Events enabled, Autosend on, Users enabled, PV enabled but if mismatch, "run not allowed", Result I and II are visible, and Default users (ADMIN, SUPERVISOR, USER1, USER2) are available (but not accessible, as the users are managed by the DMS, in this configuration scenario).

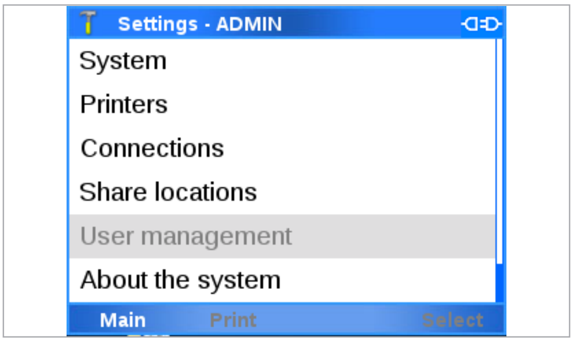

Steps

1. Info: we are still logged on.
2. DMS: send full list with (USER3, USER4, USER5).
3. Liat: ESC

Direction	Message type	Comment
Analyzer➔DMS	HEL.R01	
DMS➔analyzer	ACK.R01	
Analyzer➔DMS	DST.R01	
DMS➔analyzer	ACK.R01	Handshake successful
DMS➔analyzer	OPL.R01	DMS sends operator list.

Direction	Message type	Comment
Analyzer➔DMS	ESC.R01	Liat rejects operator list because the instrument status is "ready".
Analyzer➔DMS	END.R01	Liat: close connection.
DMS➔analyzer	ACK.R01	DMS: connection closed.


```
<HEL.R01>
    <HDR>
        <HDR.control_id V="253"/>
        <HDR.version_id V="POCT1"/>
        <HDR.creation_dttm V="2020-01-16T12:44:35-05:00"/>
    </HDR>
    <DEV>
        <DEV.device_id V="f8:dc:7a:1c:a3:c9"/>
        <DEV.vendor_id V="ROCHE"/>
        <DEV.serial_id V="M1-E-16036"/>
        <DEV.manufacturer_name V="Roche Molecular Diagnostics"/>
        <DEV.sw_version V="3.3.0.4027"/>
        <DEV.device_name V="cobasLiat"/>
        <DCP>
            <DCP.application_timeout V="30"/>
            <DCP.vendor_specific>
                ROCHE.LIAT.LOTS.R01;ROCHE.LIAT.LOTS.R02
            </DCP.vendor_specific>
        </DCP>
        <DSC>
            <DSC.connection_profile_cd V="SA"/>
            <DSC.topics_supported_cd V="OP_LST"/>
            <DSC.topics_supported_cd V="OP_LST_I"/>
            <DSC.topics_supported_cd V="D_EV"/>
            <DSC.max_message_sz V="614400"/>
        </DSC>
    </DEV>
</HEL.R01>
<ACK.R01>
    <HDR>
        <HDR.control_id V="2"/>
        <HDR.version_id V="POCT1"/>
        <HDR.creation_dttm V="2020-01-16T18:44:35+01:00"/>
    </HDR>
    <ACK>
        <ACK.type_cd V="AA"/>
        <ACK.ack_control_id V="253"/>
    </ACK>
</ACK.R01>
<DST.R01>
    <HDR>
        <HDR.control_id V="254"/>
        <HDR.version_id V="POCT1"/>
        <HDR.creation_dttm V="2020-01-16T12:44:35-05:00"/>
    </HDR>
    <DST>
        <DST.status_dttm V="2020-01-16T12:44:35-05:00"/>
        <DST.new_observations_qty V="0"/>
        <DST.new_events_qty V="97"/>
        <DST.condition_cd V="R"/>
    </DST>
```

```
</DST.R01>
<ACK.R01>
    <HDR>
        <HDR.control_id V="3"/>
        <HDR.version_id V="POCT1"/>
        <HDR.creation_dttm V="2020-01-16T18:44:36+01:00"/>
    </HDR>
    <ACK>
        <ACK.type_cd V="AA"/>
        <ACK.ack_control_id V="254"/>
    </ACK>
</ACK.R01>
<OPL.R01>
    <HDR>
        <HDR.control_id V="4"/>
        <HDR.version_id V="POCT1"/>
        <HDR.creation_dttm V="2020-01-16T18:44:42+01:00"/>
    </HDR>
    <OPR>
        <OPR.operator_id V="USER3"/>
        <ACC>
            <ACC.method_cd V="SASA"/>
            <ACC.method_cd V="FABA"/>
            <ACC.password>
                Maxi
            </ACC.password>
            <ACC.permission_level_cd V="User"/>
        </ACC>
        <NTE>
            <NTE.text V="LIAT.Contact="/>
        </NTE>
        <NTE>
            <NTE.text V="LIAT.Department="/>
        </NTE>
        <NTE>
            <NTE.text V="LIAT.ReadGeneralUserManual=NO"/>
        </NTE>
        <NTE>
            <NTE.text V="LIAT.ChangePasswordOnNextLogin=YES"/>
        </NTE>
        <NTE>
            <NTE.text V="LIAT.ReadAssayUserManuals="/>
        </NTE>
        <NTE>
            <NTE.text V="LIAT.Locked=NO"/>
        </NTE>
        <NTE>
            <NTE.text V="LIAT.BadgeBarcode="/>
        </NTE>
    </OPR>
    <OPR>
        <OPR.operator_id V="USER4"/>
        <ACC>
            <ACC.method_cd V="SASA"/>
            <ACC.method_cd V="FABA"/>
            <ACC.password>
                Maxi
            </ACC.password>
            <ACC.permission_level_cd V="User"/>
```

```
        </ACC>
        <NTE>
            <NTE.text V="LIAT.Contact=34141411 321"/>
        </NTE>
        <NTE>
            <NTE.text V="LIAT.Department="/>
        </NTE>
        <NTE>
            <NTE.text V="LIAT.ReadGeneralUserManual=NO"/>
        </NTE>
        <NTE>
            <NTE.text V="LIAT.ChangePasswordOnNextLogin=YES"/>
        </NTE>
        <NTE>
            <NTE.text V="LIAT.ReadAssayUserManuals="/>
        </NTE>
        <NTE>
            <NTE.text V="LIAT.Locked=NO"/>
        </NTE>
        <NTE>
            <NTE.text V="LIAT.BadgeBarcode="/>
        </NTE>
    </OPR>
    <OPR>
        <OPR.operator_id V="USER5"/>
        <ACC>
            <ACC.method_cd V="SASA"/>
            <ACC.method_cd V="FABA"/>
            <ACC.password>
                Maxi
            </ACC.password>
            <ACC.permission_level_cd V="User"/>
        </ACC>
        <NTE>
            <NTE.text V="LIAT.Contact="/>
        </NTE>
        <NTE>
            <NTE.text V="LIAT.Department="/>
        </NTE>
        <NTE>
            <NTE.text V="LIAT.ReadGeneralUserManual=NO"/>
        </NTE>
        <NTE>
            <NTE.text V="LIAT.ChangePasswordOnNextLogin=YES"/>
        </NTE>
        <NTE>
            <NTE.text V="LIAT.ReadAssayUserManuals="/>
        </NTE>
        <NTE>
            <NTE.text V="LIAT.Locked=NO"/>
        </NTE>
        <NTE>
            <NTE.text V="LIAT.BadgeBarcode="/>
        </NTE>
    </OPR>
</OPL.R01>
<ESC.R01>
    <HDR>
        <HDR.control_id V="255"/>
```

```
        <HDR.version_id V="POCT1"/>
        <HDR.creation_dttm V="2020-01-16T12:44:42-05:00"/>
    </HDR>
    <ESC>
        <ESC.esc_control_id V="4"/>
        <ESC.detail_cd V="TOP"/>
        <ESC.note_txt V="Message not accepted."/>
    </ESC>
</ESC.R01>
<END.R01>
    <HDR>
        <HDR.control_id V="256"/>
        <HDR.version_id V="POCT1"/>
        <HDR.creation_dttm V="2020-01-16T12:45:13-05:00"/>
    </HDR>
    <TRM>
        <TRM.reason_cd V="ABN"/>
        <TRM.note_txt V="Timeout occurred."/>
    </TRM>
</END.R01>
<ACK.R01>
    <HDR>
        <HDR.control_id V="5"/>
        <HDR.version_id V="POCT1"/>
        <HDR.creation_dttm V="2020-01-16T18:45:13+01:00"/>
    </HDR>
    <ACK>
        <ACK.type_cd V="AA"/>
        <ACK.ack_control_id V="256"/>
    </ACK>
</ACK.R01>
```

Communication scenario 12 - Replace operators list via DMS

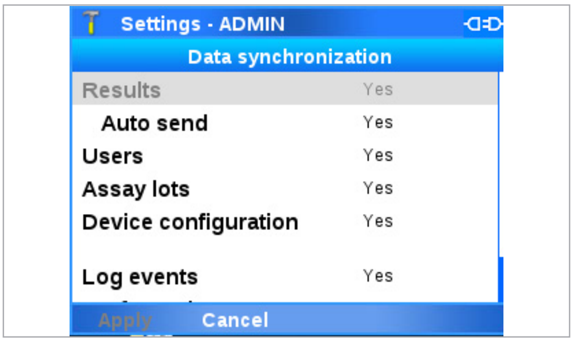

- **Purpose:** to see how the Liat accepts an operator list.
- **Configuration/Preconditions:** Assay menu with FABA & SASA Lots, Lots enabled, Events enabled, Autosend on, User enabled, PV enabled but if mismatch, "run not allowed", Result I and II are visible, and default users (ADMIN, SUPERVISOR, USER1, USER2) available but not accessible.

Steps

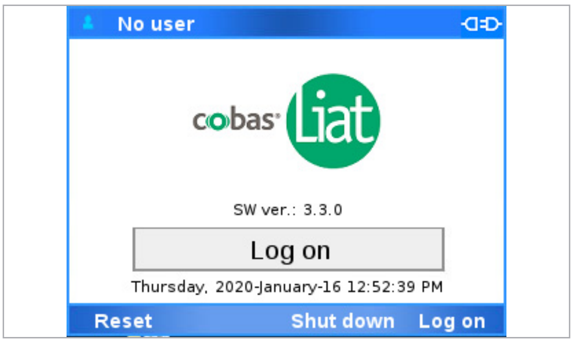

1. Info: we are logged off.
2. DMS: send full list with (USER3, USER4, USER5).
3. Liat: ACK

Direction	Message type	Comment
Analyzer→DMS	HEL.R01	
DMS→analyzer	ACK.R01	
Analyzer→DMS	DST.R01	
DMS→analyzer	ACK.R01	Handshake successful
DMS→analyzer	OPL.R01	DMS sends operator list for full replacement.
Analyzer→DMS	ACK.R01	Liat accepts operator list -> default users replaced (except ADMIN)
DMS→analyzer	EOT.R01	
Analyzer→DMS	END.R01	Liat: close connection.
DMS→analyzer	ACK.R01	DMS: connection closed.


```
<HEL.R01>
    <HDR>
        <HDR.control_id V="257"/>
        <HDR.version_id V="POCT1"/>
        <HDR.creation_dttm V="2020-01-16T12:49:35-05:00"/>
    </HDR>
    <DEV>
        <DEV.device_id V="f8:dc:7a:1c:a3:c9"/>
        <DEV.vendor_id V="ROCHE"/>
        <DEV.serial_id V="M1-E-16036"/>
        <DEV.manufacturer_name V="Roche Molecular Diagnostics"/>
        <DEV.sw_version V="3.3.0.4027"/>
        <DEV.device_name V="cobasLiat"/>
        <DCP>
            <DCP.application_timeout V="30"/>
            <DCP.vendor_specific>
                ROCHE.LIAT.LOTS.R01;ROCHE.LIAT.LOTS.R02;DTV.ROCHE.LIAT.CFG
            </DCP.vendor_specific>
        </DCP>
        <DSC>
            <DSC.connection_profile_cd V="SA"/>
            <DSC.topics_supported_cd V="OP_LST"/>
            <DSC.topics_supported_cd V="OP_LST_I"/>
            <DSC.topics_supported_cd V="D_EV"/>
            <DSC.topics_supported_cd V="DTV"/>
            <DSC.max_message_sz V="614400"/>
        </DSC>
    </DEV>
</HEL.R01>
<ACK.R01>
```

```
    <HDR>
        <HDR.control_id V="2"/>
        <HDR.version_id V="POCT1"/>
        <HDR.creation_dttm V="2020-01-16T18:49:35+01:00"/>
    </HDR>
    <ACK>
        <ACK.type_cd V="AA"/>
        <ACK.ack_control_id V="257"/>
    </ACK>
</ACK.R01>
<DST.R01>
    <HDR>
        <HDR.control_id V="258"/>
        <HDR.version_id V="POCT1"/>
        <HDR.creation_dttm V="2020-01-16T12:49:35-05:00"/>
    </HDR>
    <DST>
        <DST.status_dttm V="2020-01-16T12:49:35-05:00"/>
        <DST.new_observations_qty V="0"/>
        <DST.new_events_qty V="98"/>
        <DST.condition_cd V="S"/>
    </DST>
</DST.R01>
<ACK.R01>
    <HDR>
        <HDR.control_id V="3"/>
        <HDR.version_id V="POCT1"/>
        <HDR.creation_dttm V="2020-01-16T18:49:36+01:00"/>
    </HDR>
    <ACK>
        <ACK.type_cd V="AA"/>
        <ACK.ack_control_id V="258"/>
    </ACK>
</ACK.R01>
<OPL.R01>
    <HDR>
        <HDR.control_id V="4"/>
        <HDR.version_id V="POCT1"/>
        <HDR.creation_dttm V="2020-01-16T18:49:40+01:00"/>
    </HDR>
    <OPR>
        <OPR.operator_id V="USER3"/>
        <ACC>
            <ACC.method_cd V="SASA"/>
            <ACC.method_cd V="FABA"/>
            <ACC.password>
                Maxi
            </ACC.password>
            <ACC.permission_level_cd V="User"/>
        </ACC>
        <NTE>
            <NTE.text V="LIAT.Contact="/>
        </NTE>
        <NTE>
            <NTE.text V="LIAT.Department="/>
        </NTE>
        <NTE>
            <NTE.text V="LIAT.ReadGeneralUserManual=NO"/>
        </NTE>
```

```
        <NTE>
            <NTE.text V="LIAT.ChangePasswordOnNextLogin=YES"/>
        </NTE>
        <NTE>
            <NTE.text V="LIAT.ReadAssayUserManuals="/>
        </NTE>
        <NTE>
            <NTE.text V="LIAT.Locked=NO"/>
        </NTE>
        <NTE>
            <NTE.text V="LIAT.BadgeBarcode="/>
        </NTE>
    </OPR>
    <OPR>
        <OPR.operator_id V="USER4"/>
        <ACC>
            <ACC.method_cd V="SASA"/>
            <ACC.method_cd V="FABA"/>
            <ACC.password>
                Maxi
            </ACC.password>
            <ACC.permission_level_cd V="User"/>
        </ACC>
        <NTE>
            <NTE.text V="LIAT.Contact=34141411 321"/>
        </NTE>
        <NTE>
            <NTE.text V="LIAT.Department="/>
        </NTE>
        <NTE>
            <NTE.text V="LIAT.ReadGeneralUserManual=NO"/>
        </NTE>
        <NTE>
            <NTE.text V="LIAT.ChangePasswordOnNextLogin=YES"/>
        </NTE>
        <NTE>
            <NTE.text V="LIAT.ReadAssayUserManuals="/>
        </NTE>
        <NTE>
            <NTE.text V="LIAT.Locked=NO"/>
        </NTE>
        <NTE>
            <NTE.text V="LIAT.BadgeBarcode="/>
        </NTE>
    </OPR>
    <OPR>
        <OPR.operator_id V="USER5"/>
        <ACC>
            <ACC.method_cd V="SASA"/>
            <ACC.method_cd V="FABA"/>
            <ACC.password>
                Maxi
            </ACC.password>
            <ACC.permission_level_cd V="User"/>
        </ACC>
        <NTE>
            <NTE.text V="LIAT.Contact="/>
        </NTE>
        <NTE>
```

```
            <NTE.text V="LIAT.Department="/>
        </NTE>
        <NTE>
            <NTE.text V="LIAT.ReadGeneralUserManual=NO"/>
        </NTE>
        <NTE>
            <NTE.text V="LIAT.ChangePasswordOnNextLogin=YES"/>
        </NTE>
        <NTE>
            <NTE.text V="LIAT.ReadAssayUserManuals="/>
        </NTE>
        <NTE>
            <NTE.text V="LIAT.Locked=NO"/>
        </NTE>
        <NTE>
            <NTE.text V="LIAT.BadgeBarcode="/>
        </NTE>
    </OPR>
</OPL.R01>
<ACK.R01>
    <HDR>
        <HDR.control_id V="259"/>
        <HDR.version_id V="POCT1"/>
        <HDR.creation_dttm V="2020-01-16T12:49:44-05:00"/>
    </HDR>
    <ACK>
        <ACK.type_cd V="AA"/>
        <ACK.ack_control_id V="4"/>
        <ACK.note_txt/>
    </ACK>
</ACK.R01>
<EOT.R01>
    <HDR>
        <HDR.control_id V="5"/>
        <HDR.version_id V="POCT1"/>
        <HDR.creation_dttm V="2020-01-16T18:49:44+01:00"/>
    </HDR>
    <EOT>
        <EOT.topic_cd V="OPL"/>
    </EOT>
</EOT.R01>
<END.R01>
    <HDR>
        <HDR.control_id V="260"/>
        <HDR.version_id V="POCT1"/>
        <HDR.creation_dttm V="2020-01-16T12:50:15-05:00"/>
    </HDR>
    <TRM>
        <TRM.reason_cd V="NRM"/>
        <TRM.note_txt V="Conversation was terminated by the user."/>
    </TRM>
</END.R01>
<ACK.R01>
    <HDR>
        <HDR.control_id V="6"/>
        <HDR.version_id V="POCT1"/>
        <HDR.creation_dttm V="2020-01-16T18:50:15+01:00"/>
    </HDR>
    <ACK>
```

```
        <ACK.type_cd V="AA"/>
        <ACK.ack_control_id V="260"/>
    </ACK>
</ACK.R01>
```

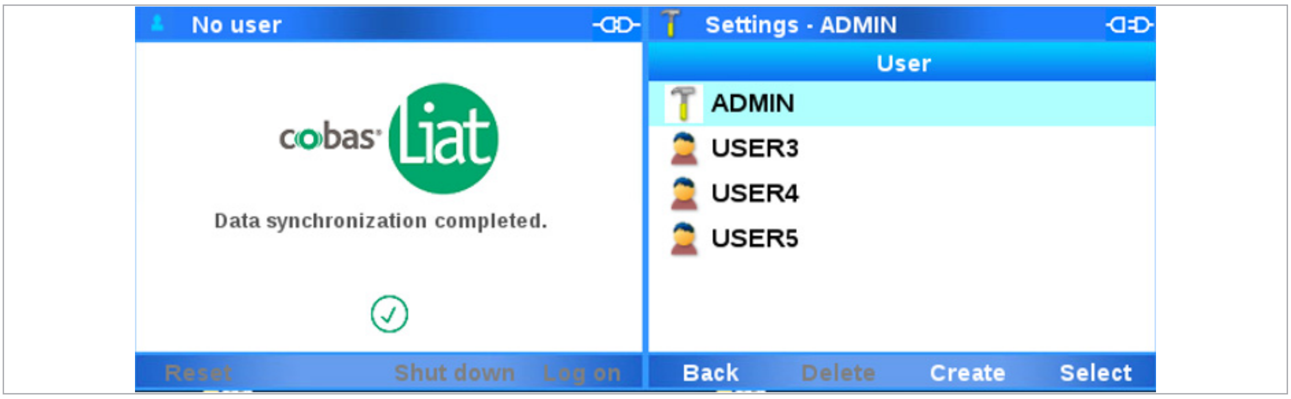

Communication scenario 13 – Delete and add operators via DMS

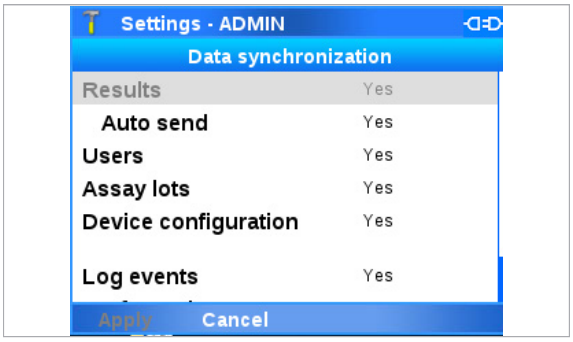

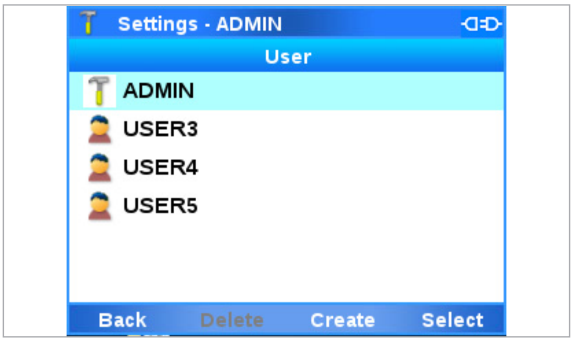

- **Purpose:** to see how the Liat processes a partial operator list, where USER5 is deleted and SUPERVISOR is added. All other users remain the same.
- **Configuration/Preconditions:** Assay menu with FABA & SASA Lots, Lots enabled, Events enabled, Autosend on, PV enabled but if mismatch, "run not allowed", Result I and II are visible, and Users (ADMIN, USER3, USER4, USER5).

Steps

1. Info: we are still logged on.
2. DMS: send delete for USER5, and add SUPERVISOR back (with the role "SUPERVISOR").
3. Liat: ACK

Direction	Message type	Comment
Analyzer➔DMS	HEL.R01	
DMS➔analyzer	ACK.R01	
Analyzer➔DMS	DST.R01	
DMS➔analyzer	ACK.R01	Handshake successful
DMS➔analyzer	OPL.R02	DMS: indicates that “USER5” must be deleted and “SUPERVISOR” must be added.
Analyzer➔DMS	ACK.R01	Liat: “User5” deleted and “SUPERVISOR” added.
DMS➔analyzer	EOT.R01	
Analyzer➔DMS	END.R01	Liat: close connection.
DMS➔analyzer	ACK.R01	DMS: connection closed.


```
<HEL.R01>
    <HDR>
        <HDR.control_id V="282"/>
        <HDR.version_id V="POCT1"/>
        <HDR.creation_dttm V="2020-01-16T13:54:35-05:00"/>
    </HDR>
    <DEV>
        <DEV.device_id V="f8:dc:7a:1c:a3:c9"/>
        <DEV.vendor_id V="ROCHE"/>
        <DEV.serial_id V="M1-E-16036"/>
        <DEV.manufacturer_name V="Roche Molecular Diagnostics"/>
        <DEV.sw_version V="3.3.0.4027"/>
        <DEV.device_name V="cobasLiat"/>
        <DCP>
            <DCP.application_timeout V="30"/>
            <DCP.vendor_specific>
                ROCHE.LIAT.LOTS.R01;ROCHE.LIAT.LOTS.R02;DTV.ROCHE.LIAT.CFG
            </DCP.vendor_specific>
        </DCP>
        <DSC>
            <DSC.connection_profile_cd V="SA"/>
            <DSC.topics_supported_cd V="OP_LST"/>
            <DSC.topics_supported_cd V="OP_LST_I"/>
            <DSC.topics_supported_cd V="D_EV"/>
            <DSC.topics_supported_cd V="DTV"/>
            <DSC.max_message_sz V="614400"/>
        </DSC>
    </DEV>
</HEL.R01>
<ACK.R01>
    <HDR>
        <HDR.control_id V="2"/>
        <HDR.version_id V="POCT1"/>
        <HDR.creation_dttm V="2020-01-16T19:54:35+01:00"/>
    </HDR>
    <ACK>
        <ACK.type_cd V="AA"/>
        <ACK.ack_control_id V="282"/>
    </ACK>
</ACK.R01>
<DST.R01>
    <HDR>
        <HDR.control_id V="283"/>
```

```
        <HDR.version_id V="POCT1"/>
        <HDR.creation_dttm V="2020-01-16T13:54:35-05:00"/>
    </HDR>
    <DST>
        <DST.status_dttm V="2020-01-16T13:54:35-05:00"/>
        <DST.new_observations_qty V="0"/>
        <DST.new_events_qty V="135"/>
        <DST.condition_cd V="S"/>
    </DST>
</DST.R01>
<ACK.R01>
    <HDR>
        <HDR.control_id V="3"/>
        <HDR.version_id V="POCT1"/>
        <HDR.creation_dttm V="2020-01-16T19:54:36+01:00"/>
    </HDR>
    <ACK>
        <ACK.type_cd V="AA"/>
        <ACK.ack_control_id V="283"/>
    </ACK>
</ACK.R01>
<OPL.R02>
    <HDR>
        <HDR.control_id V="4"/>
        <HDR.version_id V="POCT1"/>
        <HDR.creation_dttm V="2020-01-16T19:54:56+01:00"/>
    </HDR>
    <UPD>
        <UPD.action_cd V="D"/>
        <OPR>
            <OPR.operator_id V="USER5"/>
        </OPR>
    </UPD>
    <UPD>
        <UPD.action_cd V="I"/>
        <OPR>
            <OPR.operator_id V="SUPERVISOR"/>
            <ACC>
                <ACC.method_cd V="SASA"/>
                <ACC.method_cd V="FABA"/>
                <ACC.password>
                    1234
                </ACC.password>
                <ACC.permission_level_cd V="SUPERVISOR"/>
            </ACC>
            <NTE>
                <NTE.text V="LIAT.Contact="/>
            </NTE>
            <NTE>
                <NTE.text V="LIAT.Department="/>
            </NTE>
            <NTE>
                <NTE.text V="LIAT.ReadGeneralUserManual=NO"/>
            </NTE>
            <NTE>
                <NTE.text V="LIAT.ChangePasswordOnNextLogin=YES"/>
            </NTE>
            <NTE>
                <NTE.text V="LIAT.ReadAssayUserManuals="/>
```

```
            </NTE>
            <NTE>
                <NTE.text V="LIAT.Locked=NO"/>
            </NTE>
            <NTE>
                <NTE.text V="LIAT.BadgeBarcode="/>
            </NTE>
        </OPR>
    </UPD>
</OPL.R02>
<ACK.R01>
    <HDR>
        <HDR.control_id V="284"/>
        <HDR.version_id V="POCT1"/>
        <HDR.creation_dttm V="2020-01-16T13:54:44-05:00"/>
    </HDR>
    <ACK>
        <ACK.type_cd V="AA"/>
        <ACK.ack_control_id V="4"/>
        <ACK.note_txt/>
    </ACK>
</ACK.R01>
<EOT.R01>
    <HDR>
        <HDR.control_id V="5"/>
        <HDR.version_id V="POCT1"/>
        <HDR.creation_dttm V="2020-01-16T19:54:44+01:00"/>
    </HDR>
    <EOT>
        <EOT.topic_cd V="OPL"/>
    </EOT>
</EOT.R01>
<END.R01>
    <HDR>
        <HDR.control_id V="285"/>
        <HDR.version_id V="POCT1"/>
        <HDR.creation_dttm V="2020-01-16T13:55:47-05:00"/>
    </HDR>
    <TRM>
        <TRM.reason_cd V="NRM"/>
        <TRM.note_txt V="Conversation was terminated by the user."/>
    </TRM>
</END.R01>
<ACK.R01>
    <HDR>
        <HDR.control_id V="6"/>
        <HDR.version_id V="POCT1"/>
        <HDR.creation_dttm V="2020-01-16T19:55:47+01:00"/>
    </HDR>
    <ACK>
        <ACK.type_cd V="AA"/>
        <ACK.ack_control_id V="285"/>
    </ACK>
</ACK.R01>
```

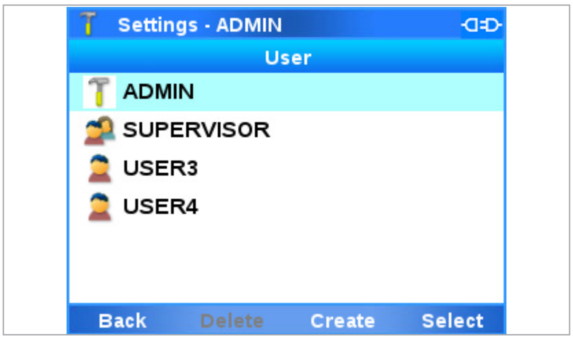
Settings - ADMIN
User
ADMIN
SUPERVISOR
USER3
USER4
Back
Delete
Create
Select
